# Synthesis, Antimicrobial Activities, and Model of Action of Indolyl Derivatives Containing Amino-Guanidinium Moieties

**DOI:** 10.3390/molecules30040887

**Published:** 2025-02-14

**Authors:** Yu-Xi Li, Xiang Geng, Qi Tao, Ruo-Chen Hao, Ya-Jun Yang, Xi-Wang Liu, Jian-Yong Li

**Affiliations:** 1 Key Lab of New Animal Drug of Gansu Province, Key Lab of Veterinary Pharmaceutical Development of Ministry of Agriculture and Rural Affairs, Lanzhou Institute of Husbandry and Pharmaceutical Sciences of Chinese Academy of Agricultural Sciences, Lanzhou 730050, China; 2 School of Health Nursing, Fuyang Vocational Technical College, Fuyang 236000, China

**Keywords:** aminoguanidine, indole, synthesis, antibacterial activity, *K. pneumoniae*

## Abstract

The objectives of the study were to design, synthesize, and evaluate the antibacterial activity of a series of novel aminoguanidine-indole derivatives. Thirty-seven new compounds were effectively synthesized through nucleophilic substitution reaction and guanidinylation reaction. Chemical structures of all the desired compounds were identified by NMR and HR-MS spectroscopy. Most of the synthesized compounds showed significant antibacterial activity against ESKAPE pathogens and clinical resistant *Klebsiella pneumoniae* (*K. pneumoniae*) isolates. *K. pneumoniae* is an important opportunistic pathogen that often threatens the health of immunocompromised people such as the elderly, children, and ICU patients. The most active compound **4P** showed rapid bactericidal activity against resistant *K. pneumoniae* 2108 with MIC and MBC values that were 4 and 8 µg/mL, respectively. The hemolytic activity of **4P** was low, with an HC_50_ value of 123.6 µg/mL. Compound **4P** induced the depolarization of the bacterial membrane and disrupted bacterial membrane integrity and was not prone to antibiotic resistance. The dihydrofolate reductase (DHFR) activity was also notably inhibited by **4P** in vitro. Molecular docking revealed that the aminoguanidine moiety and indole structure of **4P** played an important role in binding to the target site of the *K. pneumoniae* dihydrofolate reductase (DHFR) receptor. In the mouse pneumonia model caused by *K. pneumoniae*, **4P** improved the survival rate of mice, reduced bacterial loads, and alleviated tissues’ pathological injuries at a dosage of 4 mg/kg. Therefore, compound **4P** may be a promising lead compound or drug candidate for antibacterial purposes against *K. pneumoniae*.

## 1. Introduction

The emergence of drug-resistant pathogenic bacteria has become an increasing threat to global health in recent years [1,2]. In particular, the ESKAPE pathogens (*Enterococcus faecalis*, *Staphylococcus aureus*, *Klebsiella pneumoniae*, *Acinetobacter baumannii*, *Pseudomonas aeruginosa*, and *Escherichia coli*), released by the WHO in 2017, draw much attention due to their rapid development of resistance to conventional antibiotics [3]. ESKAPE pathogens have been associated with a variety of infections, including urinary tract infections, pneumonia, and bloodstream infections, which can be fatal in severe cases [4]. As an important opportunistic pathogen, *K. pneumoniae* can colonize on the surface of the respiratory tract, the digestive tract, and medical devices [5,6], threatening the health of immunocompromised people such as the elderly [7], children [8], and ICU patients [9]. Healthy adults can also be infected by hypervirulent *K. pneumoniae* [10]. The WHO has recently emphasized the importance of *K. pneumoniae* in clinical practice [11]. The inappropriate use of antibiotics has led to the development of a frightening level of resistance, in particular the emergence of multidrug-resistant (MDR) bacteria and even ‘superbugs’, contributing to a significant economic burden [12,13]. The escalating threat posed by these pathogens requires not only the rational use of available antibiotics, but also the development of new classes of drugs to reduce deaths from infectious diseases.

Indole and its derivatives, as important biological signaling molecules, are widely distributed in nature and affect various aspects of bacterial physiology, including spore formation, cell division, plasmid stability, drug resistance, biofilm formation and virulence [14,15]. Recently, indole derivatives have received particular attention in the pharmaceutical field as molecules with antimicrobial activity [16,17]. Indole derivatives have great potential for treating methicillin-resistant *Staphylococcus aureus* (MRSA) [18]. A series of indole derivatives have been synthesized and some compounds have shown promising antimicrobial activity against Gram-positive and Gram-negative bacteria, and fungi [19,20]. This makes indole an excellent backbone for the synthesis of new antimicrobial compounds.

Antimicrobial activity depends on the penetration of the antimicrobial agent through the multilayered bacterial envelope and its interaction with the target pathogen. [21]. For Gram-negative bacteria, this process is more complicated due to the presence of an outer membrane, which requires additional attention in the design of novel antibacterial agents [22,23]. The accumulation abilities of 180 compounds were investigated in *E. coli*, with results showing that amphiphilic and rigid small molecules containing amines with low sphericity were most likely to accumulate [24]. The importance of ionizable nitrogen, low three-dimensionality, and rigidity for small molecules entering Gram-negative bacteria was also reviewed [25]. Guanidine was useful for enhancing the activity of compounds and was widely used in many drug molecules. N-Alkyl guanidiniums promoted the uptake of compounds in bacteria, and aminoguanidines were discovered with better activity [19,26,27].

Colistin is regarded as the final line of defense in the treatment of Gram-negative bacterial infections [28]. Consequently, the development of a novel antibiotic with comparable bactericidal efficacy to colistin and low resistance is therefore of considerable practical importance. In this study, a series of aminoguanidyl indole derivatives (**3A-3V**, **4L-4P**, **5L-5P** and **6L-6P**) were first designed and synthesized. These included indoles and 7-azaindoles with various substituted benzyl bromides and aminoguanidine to produce the target compound, then their structures were characterized. Antibacterial activity against both Gram-positive and Gram-negative bacteria was assessed using colistin as a positive control. The role of the active molecule **4P** in disrupting cell membrane integrity was investigated. A molecular docking study was performed between **4P** and *K. pneumoniae* dihydrofolate reductase (DHFR) to confirm the target specificity and elucidate the binding mechanism. Furthermore, the hemolytic activity of **4P** was also examined. This study provides a class of compounds for the development of novel antibacterial agents.

## 2. Result and Discussion

### 2.1. Chemistry

**Scheme 1:**
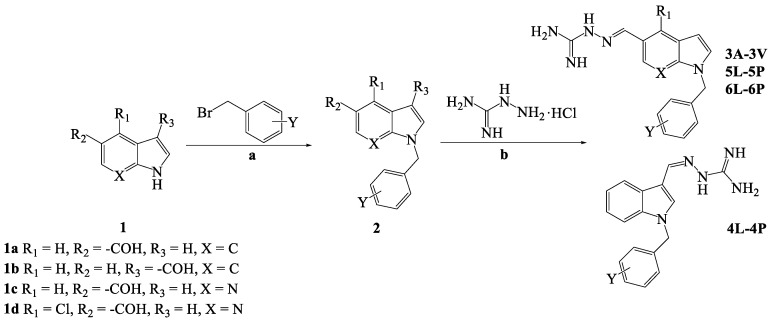
The synthesis route of compounds **3A-3V**, **4L-4P**, **5L-5P** and **6L-6P**. Reagents and Conditions: (a) dry DMF, KOH, RT, 0.75–4 h; (b) ethanol absolute, 10 drops of concentrated HCl, 50 °C, 2–6 h.

The synthetic route for the preparation of the guanidyl indole derivatives (**3A-3V**, **4L-4P**, **5L-5P**, and **6L-6P**) and intermediates **is** shown in Scheme 1. Compounds (**2**) were prepared from starting material **1** and substituted benzyl bromides under alkaline conditions. With the key intermediate in hand, all the target derivatives (Table 1) were prepared directly in the presence of aminoguanidine hydrochloride, and concentrated hydrochloric acid by stirring in an oil-bath for 2–6 h at 50 °C [29]. Finally, the structures of the desired compounds were characterized by ^1^H NMR, ^13^C NMR, **^19^F NMR** and high-resolution mass spectrometry (HR-MS) (Supplementary Material S1). The purity of all the compounds was 100% using the area normalization method by HPLC (Supplementary Material S2).

### 2.2. Evaluation of In Vitro Antimicrobial Activity

The in vitro antimicrobial activity of the synthesized compounds was evaluated to obtain the minimum inhibitory concentrations (MICs) and minimum bactericidal concentrations (MBCs) using a serial two-fold dilution method against ESKAPE strains, MRSA and clinical *K. pneumoniae* isolates. Colistin was selected as the positive control and DMSO (0.1%) was selected as the negative control.

Initial screening results are presented in Table 2. In general, ESKAPE strains and MRSA strains were susceptible to most of the compounds with MICs in the range of 2–64 µg/mL. Compounds **3I-3U** and **4L-4P** based on indole and compounds **5N-5P** based on 7-azaindole showed potent antibacterial activities with MICs in the range of 2–16 µg/mL (except for *Pseudomonas aeruginosa* ATCC27853). It was also found that both Gram-positive and Gram-negative bacteria had approximately the same susceptibility to the test compounds. N-benzyl indole derivatives containing trifluoromethyl (CF_3_) groups, Cl atoms, Br atoms, or both Cl and F atoms showed higher antibacterial activity. The Cl atom on the 7-azaindole ring significantly reduced the activity of the compounds **6L-6P**. This suggests that the presence and the position of halogen atoms are critical for the efficacy of these aminoguanidyl indole derivatives. Comparing **3L-3P** and **4L-4P**, the activity was slightly influenced by the position of the aminoguanidine. Comparing **3L-3P** and **5L-5P**, the activity was declined by the 7-azaindole ring. Among these compounds, the bacteriostatic activities of 16 selected compounds (**3I-3U, 4O**, **4P** and **5P**) were tested against clinical *K. pneumoniae* isolates (Table 3). All 16 compounds showed antibacterial activity (4–16 µg/mL) against clinical *K. pneumoniae* isolates (including susceptible and drug-resistant bacteria). **3O**, **3P**, **4O** and **4P** showed the best antibacterial effect with MICs ranging from 4 to 8 µg/mL. The antibacterial activity of **3O**, **3P**, **4O** and **4P** was generally comparable to that of colistin. Moreover, the MBCs for compounds with MIC values below 64 µg/mL were evaluated. Most compounds displayed MBCs that were 1–4 times higher than their MICs against the tested strains.

**Figure 1:**
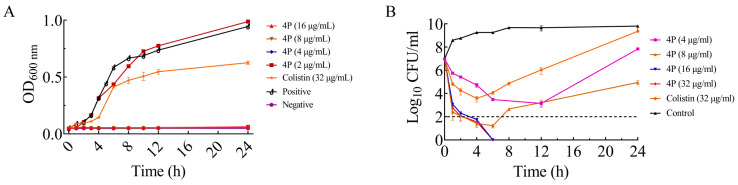
The result of the growth curve and bactericidal time-kill kinetics assay. (**A**) Growth curve of *K.P.* 2108 in the presence of **4P** (2, 4, 8, and 16 µg/mL) and colistin (32 µg/mL). (**B**) Time-kill curve of *K.P.* 2108 in the presence of **4P** (4, 8, 16, and 32 µg/mL) and colistin (32 µg/mL).

As the most effective compound, the bacteriostatic and time-kill assay of **4P** was investigated in vitro. **4P** was able to suppress the growth of MDR strain *K.P.* 2108 at concentrations of 1MIC, 2MIC and 4MIC. Colistin at 32 µg/mL did not completely inhibit the growth of *K.P.* 2108 (Figure 1A). The time-kill assay showed that different concentrations of **4P** exhibited a concentration-dependent and time-dependent bactericidal response (Figure 1B).

### 2.3. **4P** Disrupted Bacteria Viability and Morphology

**Figure 2:**
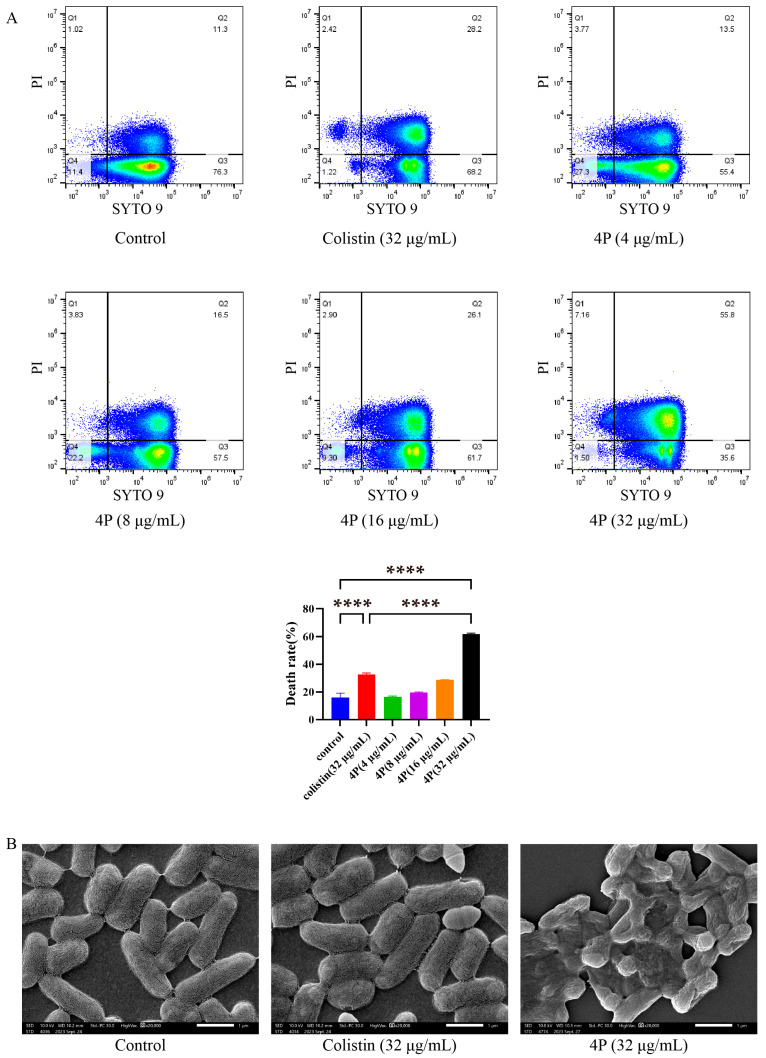
The result of FC and SEM. (**A**) Quantified results of the flow cytometry analysis after the treatment of *K.P.* 2108 with colistin (32 µg/mL) and **4P** (4, 8, 16, 32 µg/mL). (**B**) Scanning electron microscope images of *K.P.* 2108 under the treatment with colistin (32 µg/mL) and **4P** (32 µg/mL). The asterisk denotes statistically significant differences: **** *p* < 0.0001.

Flow cytometry (FC) analysis showed that the fluorescence intensity of the **4P** (32 µg/mL) group was approximately twice that of the colistin (32 µg/mL) group, although the fluorescence intensity of two groups increased to some extent after drug treatment (Figure 2A). Moreover, the effect of **4P** was concentration dependent. The effect of **4P** and colistin on the bacterial structure was further visualized by scanning electron microscopy (SEM) analysis (Figure 2B). Bacteria in the control group and colistin (32 µg/mL) were normal, rod-shaped, and full with no breaks in morphology. However, most of the bacteria treated with **4P** (32 µg/mL) were severely damaged and lost the normal bacterial morphology, were atrophic, and completely collapsed.

### 2.4. **4P** Exerts Its Antibacterial Action on the Bacterial Membrane

**Figure 3:**
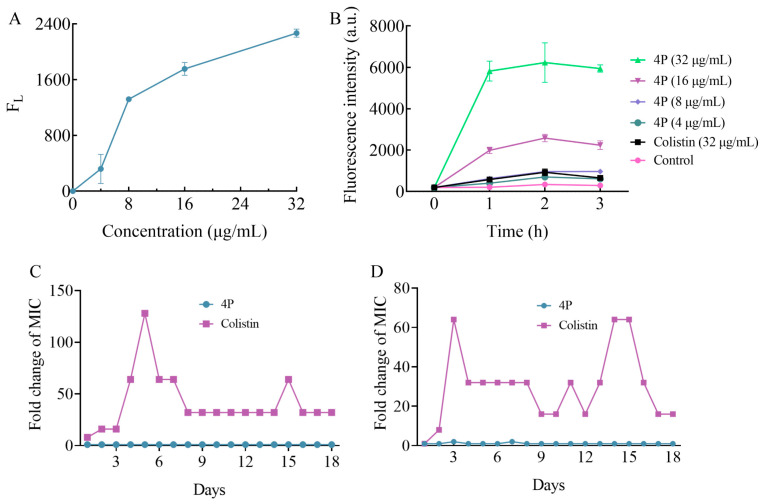
The effect of **4P** on bacterial membrane and bacterial resistance development. (**A**) Increased membrane permeability after treatment with different concentrations of **4P**. (**B**) The dynamic curves of the integrity of the inner membrane probed with PI for *K.P.* 2108, under the treatment of **4P** (4, 8, 16, and 32 µg/mL) and colistin (32 µg/mL). (**C**,**D**) Propensity of the development of *K. pneumoniae* resistance to **4P** and colistin after repetitive treatments for 18 days (C for ATCC 700603, D for *K.P.* 2112).

The effect of **4P** on bacterial membrane perturbation was evaluated using the fluorescent dye DiSC_3_(5). DiSC_3_(5) localizes in the bacterial membrane depending on an intact the membrane potential gradient, where self-quenching occurs [30]. **4P** was able to depolarize the membrane potential, releasing membrane-bound DiSC_3_(5) into the detection medium, where the fluorescence intensity was measured. The fluorescence intensity of DiSC_3_(5) was detected 30 min after treatment with **4P**. As shown in Figure 3A, a dose-dependent increase in the level of membrane depolarization was observed after treatment. This result indicated that the bacterial membrane was disrupted after treatment with **4P**. The membrane integrity of the bacteria was examined by measuring the fluorescence values of propidium iodide (PI). The fluorescence intensity increased when treated with the concentrations above 1MIC, and fluorescence intensities increased with increasing **4P** concentrations (Figure 3B). These findings demonstrated that **4P** caused damage to the plasma membrane.

### 2.5. **4P** Was Not Prone to Antibiotic Resistance

The development of bacterial resistance to conventional antibiotics has become a major public health concern [31]. Resistance is commonly characterized as a greater than 4-fold increase from the initial MIC measurement [32]. Therefore, the tendency to inhibit drug resistance is one of the most important properties of an antibacterial agent [33]. The ability of **4P** to inhibit the development of antibacterial resistance was examined in *K. pneumoniae* ATCC700603 and *K.P.* 2112. As shown in Figure 3C,D, after 18 days of serial challenge, two strains exhibited an increase in colistin resistance, with an 8- to 128-fold increase in colistin MICs, and the MIC value of **4P** showed minimal change. Therefore, **4P** was less susceptible, promoting antibacterial resistance. The results for colistin resistance induced to *K. pneumoniae* under laboratory conditions were consistent with related studies [34,35].

### 2.6. Docking Analysis

**Figure 4:**
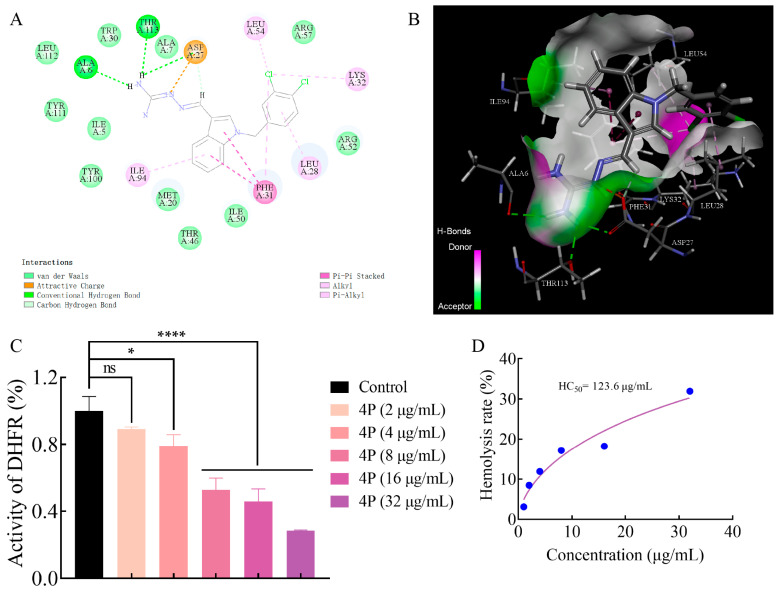
The docking result and enzymatic activity of **4P** and DHFR, and hemolysis. (**A**,**B**) Interaction of **4P** with the DHFR protein ((**A**) for two-dimensional, (**B**) for three-dimensional). (**C**) The inhibition rate of **4P** on the in vitro activity of the DHFR protein. (**D**) Hemolysis rate of **4P**. The value with “ns” is not significant; the asterisk denotes statistically significant differences: * *p* < 0.05; **** *p* < 0.0001.

It was previously shown that carbazole derivatives with an aminoguanidine group have strong binding capabilities with *E. coli* dihydrofolate reductase (DHFR) [36]. Therefore, the binding capacity of the interaction between **4P** and the *K. pneumoniae* DHFR protein was investigated. DHFR has been identified as one of the important antimicrobial targets because it plays a crucial role in the regulation of bacterial metabolism [37,38]. As shown in the molecular docking results (Figure 4A), the docked pose of **4P** with DHFR had a CDOCKER ENERGY of 30.6912, suggesting that **4P** can bind to potential binding sites in the DHFR. Eight active site residues (ALA6, ASP27, LEU28, PHE31, LYS32, LEU54, ILE94 and THR113) were involved in recognition by **4P** (Figure 4B). In DHFR, the ALA6 and THR113 side chains participated in conventional hydrogen bond interaction with the amino group of guanidium of **4P**, the PHE31 side chain participated in Pi-Pi stacked with **4P**, the ASP27 side chain participated in an attractive charge with **4P**, and the LEU54, LYS32, LEU28, and ILE94 side chains were involved in alkyl stacking with **4P**. In addition to these, **4P** can interact with acid residues of DHFR through a carbon-hydrogen bond. Indole and aryl groups enhanced the hydrophobic interactions between **4P** and the residues in the DHFR active pocket. These docking results indicated that guanidium and indole groups played an important role in binding with the *K. pneumoniae* DHFR protein.

### 2.7. **4P** Inhibited DHFR Activities In Vitro

An in vitro enzyme activity assay was performed to investigate whether **4P** can bind and inhibit the DHFR protein. Different concentrations of **4P** (1/2MIC, 1MIC, 2MIC, 4MIC, and 8MIC) were used to test their inhibitory effects on the DHFR protein. The results showed that the enzyme activity was significantly inhibited at 1MIC when compared to the untreated group. Furthermore, when bacteria were treated with **4P** at different concentrations, the enzyme activity of DHFR activity was significantly inhibited in a concentration-dependent manner (Figure 4C). These results suggested that **4P** exerted its antibacterial activity by interfering with DHFR. Certainly, this was probably not the only antibacterial mechanism for **4P**.

### 2.8. Hemolytic Activity

The antibacterial mechanism of guanidine compounds is considered to interfere with the bacterial cell membrane [39], which is consistent with our experimental results. To investigate the potential clinical applicability of compounds **3P**, **4P** and **5P**, hemolysis assays were performed (Table 4). The results showed that **4P** had a lower hemolysis against sheep blood red cells, with an HC_50_ value of 123.6 µg/mL (Figure 4D). **4P** exhibited excellent selectivity for bacterial and mammalian red blood cells (selectivity index = 30.90).

Considering the MIC and hemolysis of the compounds, **4P** was selected for subsequent experiments.

### 2.9. In Vivo Efficacy

**Figure 5:**
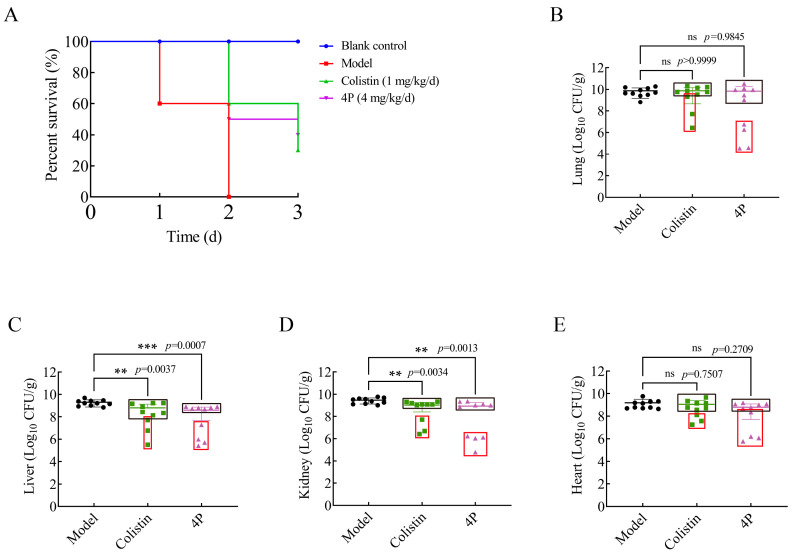
**4P** can treat *K. pneumoniae* infection in mice. (**A**) Survival rates of female BALB/c mice (*n* = 10) infected with *K. pneumoniae*. (**B**–**E**) Bacterial loads in infected lungs, livers, kidneys, and hearts of mice (*n* = 10). The black box represents the bacterial load of mice that died due to infection; the red box represents the bacterial load of the surviving mice. Each symbol represents a mouse. The value with “ns” is not significant; the asterisk denotes statistically significant differences: ** *p* < 0.01; *** *p* < 0.001.

**Figure 6:**
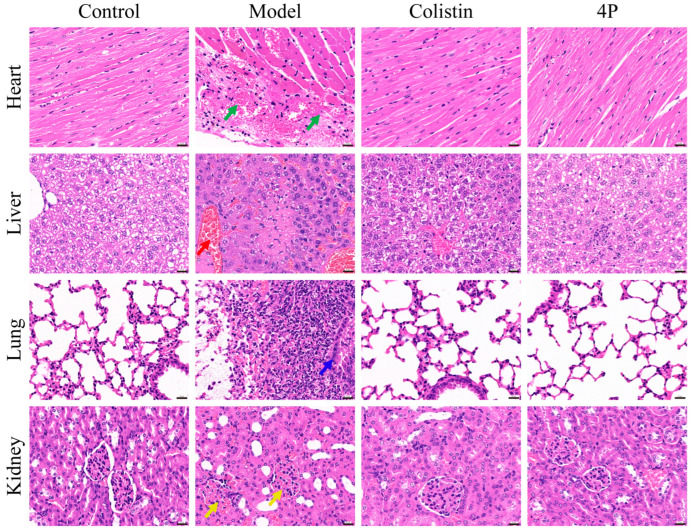
Histopathological analysis of different tissues using hematoxylin-eosin (HE) staining. The heart, liver, lung, and kidney were histologically analyzed in the mouse pneumoniae model. The green arrow indicates the necrosis of cardiac myofibers, the congestion and dilatation of the blood vessels in heart tissue. The red arrow indicates hepatocellular degeneration and hepatic sinusoids in liver tissue. The blue arrow indicates the necrosis and edema of alveolar epithelial cells, alveolar edema, and inflammatory cell infiltration in lung tissue. The yellow arrow indicates extensive tubular necrosis in the kidney. Scale bar, 20 µm.

As **4P** showed good bacteriostatic and bactericidal capabilities in vitro, its in vivo efficacy was further evaluated by measuring the survival rate of mice after a single lethal challenge of *K. pneumoniae*. The lethality rate of mice in the untreated group was 100% by day 2 post-infection. There was a significant improvement in survival under **4P,** with slightly higher survival rates than colistin treatment during this observation period (Figure 5A). Four tissues (heart, liver, lung, and kidney) were harvested at the time of death for bacterial load determination and histopathological observation. The results of the bacterial load analysis showed that there were reductions in bacterial load after **4P** treatment compared to the untreated group, although no significant differences were detected in the lung and heart tissues. In contrast, the tissues from surviving mice exhibited a lower bacterial load (black box). Moreover, the *p*-values in the **4P** treatment group were lower than those in the colistin group, indicating that **4P** displayed a lower bacterial load compared with the colistin group (Figure 5B–E). The histopathological changes were examined by light microscopy to investigate whether **4P** reduced the tissue injury caused by *K. pneumoniae*. Compared with the blank group, there were significant pathological changes in different tissues of the model group. The heart tissue showed the necrosis of cardiac myofibers, along with the congestion and dilatation of the blood vessels. Hepatocellular degeneration and hepatic sinusoids were observed in the liver, and the kidney showed extensive tubular necrosis. The most severe lesions were observed in the lung with the necrosis and edema of alveolar epithelial cells, alveolar edema, and inflammatory cell infiltration. After treatment with **4P**, all the different tissues were normal, with no significant lesions (Figure 6).

## 3. Materials and Methods

### 3.1. Chemistry

#### 3.1.1. General

All chemicals used in this study were of analytical reagents and solvents were not specially treated before use. The reaction process was monitored by Thin Layer Chromatography (TLC), and the products were purified by silica gel column chromatography. The ^1^H, ^13^C and ^19^F NMR spectra were recorded on an Agilent DD2-600MHz NMR spectrometer at room temperature, operating at 600 MHz for ^1^H NMR, 151 MHz for ^13^C NMR and 376 MHz for ^19^F NMR by using *DMSO-d6* as solvent. The mass spectra were obtained at Agilent 6530 QTOF. The purity of all the title compounds was obtained with the area normalization method by HPLC (Agilent Technologies 1290 Infinity).

#### 3.1.2. Synthesis of Intermediates (**2**)

A 50 mL round-bottom flask equipped with a magnetic stirring bar was filled with 5 mL of DMF and 224 mg (4 mmol) of potassium hydroxide. The mixture was stirred at room temperature for 5 min before the addition of 1 mmol of compound **1**. We continued stirring for 45 min, then 1 mmol of benzyl bromide with a substituent on the benzene ring was added. It was stirred again for 0.75–4 h. When the reaction was complete, 40 mL of water was added. The mixture was filtered or extracted with ethyl acetate (the ethyl acetate layer was washed twice with saturated salt water, anhydrous sodium sulfate was used to remove water, and ethyl acetate was removed by vacuum distillation). The obtained crude products were recrystallized in ethanol or purified by silica gel column chromatography to yield the desired compounds.

#### 3.1.3. General Procedure for the Synthesis of Compounds **3A-3V**, **4L-4P**, **5L-5P** and **6L-6P**

One mmol of compound **2** and 110 mg (1 mmol) of aminoguanidine hydrochloride were stirred in 10 mL of ethanol. Then, 10 drops of concentrated hydrochloric acid and 2 mL of water were added. The mixture was stirred in an oil bath at 50 °C for 2–6 h. Subsequently, the solution was evaporated to dryness under reduced pressure, and the residue was purified by silica gel column chromatography (CH_2_Cl_2_:CH_3_OH = 15:1~10:1) to yield the desired compounds.

The spectral data of the novel compounds are listed below.

(*E*)-2-((1-(2-fluorobenzyl)-1*H*-indol-5-yl) methylene) hydrazine-1-carboximidamide (**3A**). Yield: 65.5%; ^1^H NMR (600 MHz, *DMSO-d6*) δ 11.89 (br s, 1H), 8.21 (s, 1H), 7.95 (d, *J* = 1.1 Hz, 1H), 7.84 (br s, 3H), 7.73 (dd, *J* = 8.7, 1.5 Hz, 1H), 7.53 (d, *J* = 8.7 Hz, 1H), 7.49 (d, *J* = 3.1 Hz, 1H), 7.30 (ddd, *J* = 15.4, 5.5, 1.9 Hz, 1H), 7.22−7.17 (m, 1H), 7.11–7.02 (m, 2H), 6.54 (dd, *J* = 3.2, 0.5 Hz, 1H), 5.49 (s, 2H); ^13^C NMR (151 MHz, *DMSO-d6*) δ160.36 (d, *J*_CF_ = 245.2 Hz), 155.74, 148.74, 137.40, 130.72, 130.27 (d, *J*_CF_ = 8.2 Hz), 129.96 (d, *J*_CF_ = 4.0 Hz), 128.49, 125.47, 125.13 (d, *J*_CF_ = 12.0 Hz), 125.07, 122.16, 120.83, 115.90 (d, *J*_CF_ = 20.9 Hz), 110.86, 102.69, 43.74 (d, *J*_CF_ = 4.0 Hz); ^19^F NMR (376 MHz, *DMSO-d6*) δ −117.72–117.85 (m, 1F); HRMS (ESI^+^): *m*/*z* calcd for C_17_H_16_FN_5_ [M + H]^+^ 310.1463, found 310.1477.

(*E*)-2-((1-(3-fluorobenzyl)-1*H*-indol-5-yl) methylene) hydrazine-1-carboximidamide (**3B**). Yield: 91.8%; ^1^H NMR (600 MHz, *DMSO-d6*) 11.90 (br s, 1H), 8.20 (s, 1H), 7.96 (d, J = 1.0 Hz, 1H), 7.80 (br s, 3H), 7.71 (dd, J = 8.7, 1.4 Hz, 1H), 7.57 (d, J = 3.2 Hz, 1H), 7.52 (d, J = 8.7 Hz, 1H), 7.32 (td, J = 8.1, 6.3 Hz, 1H), 7.07–6.98 (m, 3H), 6.55 (d, J = 3.2 Hz, 1H), 5.46 (s, 2H); ^13^C NMR (151 MHz, *DMSO-d6*) δ162.62 (d, *J*_CF_ = 244.0 Hz), 155.75, 148.75, 141.37 (d, *J*_CF_ = 7.1 Hz), 137.37, 131.04 (d, *J*_CF_ = 8.4 Hz), 130.82, 128.60, 125.48, 123.52 (d, *J*_CF_ = 2.7 Hz), 122.16, 120.84, 114.67 (d, *J*_CF_ = 20.9 Hz), 114.28 (d, *J*_CF_ = 21.8 Hz), 111.03, 102.67, 49.03; ^19^F NMR (376 MHz, *DMSO-d6*) δ −113.06 (td, *J* = 9.3, 6.2 Hz, 1F); HRMS (ESI^+^): *m*/*z* calcd for C_17_H_16_FN_5_ [M + H]^+^ 310.1463, found 310.1460.

(*E*)-2-((1-(4-fluorobenzyl)-1*H*-indol-5-yl) methylene) hydrazine-1-carboximidamide (**3C**). Yield: 98.0%; ^1^H NMR (600 MHz, *DMSO-d6*) δ 11.92 (br s, 1H), 8.20 (s, 1H), 7.94 (d, J = 1.0 Hz, 1H), 7.78 (br s, 3H), 7.71 (dd, J = 8.7, 1.4 Hz, 1H), 7.55 (d, J = 3.2 Hz, 1H), 7.52 (d, J = 8.7 Hz, 1H), 7.28–7.22 (m, 2H), 7.14–7.08 (m, 2H), 6.53 (d, J = 3.1 Hz, 1H), 5.42 (s, 2H); ^13^C NMR (151 MHz, *DMSO-d6*) δ161.88 (d, *J*_CF_ = 243.2 Hz), 155.76, 148.76, 137.30, 134.65 (d, *J*_CF_ = 3.0 Hz), 130.68, 129.63 (d, *J*_CF_ = 8.3 Hz, 2C), 128.61, 125.40, 122.14, 120.75, 115.78 (d, *J*_CF_ = 21.4 Hz, 2C), 111.05, 102.56, 48.86; ^19^F NMR (376 MHz, *DMSO-d6*) δ −115.11–115.21 (m, 1F); HRMS (ESI^+^): *m*/*z* calcd for C_17_H_16_FN_5_ [M + H]^+^ 310.1463, found 310.1460.

(*E*)-2-((1-(2,4-difluorobenzyl)-1*H*-indol-5-yl) methylene) hydrazine-1-carboximidamide (**3D**): Yield: 25.4%; ^1^H NMR (600 MHz, *DMSO-d6*) δ 11.91 (br s, 1H), 8.20 (s, 1H), 7.95 (d, J = 1.2 Hz, 1H), 7.83 (br s, 3H), 7.73 (dd, J = 8.7, 1.4 Hz, 1H), 7.54 (d, J = 8.7 Hz, 1H), 7.48 (d, J = 3.2 Hz, 1H), 7.28–7.21 (m, 1H), 7.16 (td, J = 8.6, 6.8 Hz, 1H), 7.00 (td, J = 8.6, 2.6 Hz, 1H), 6.54 (d, J = 3.2 Hz, 1H), 5.46 (s, 2H); ^13^C NMR (151 MHz, *DMSO-d6*) δ162.27 (dd, *J*_CF_ = 246.6, 12.0 Hz), 160.46 (dd, *J*_CF_ = 248.2, 12.5 Hz), 155.76, 148.69, 137.32, 131.29 (dd, *J*_CF_ = 9.9, 5.6 Hz), 130.60, 128.50, 125.53, 122.17, 121.58 (dd, *J*_CF_ = 15.2, 3.7 Hz), 120.87, 112.12 (dd, *J*_CF_ = 21.3, 3.6 Hz), 110.83, 104.56 (t, *J*_CF_ = 25.9 Hz), 102.77, 43.30 (d, *J*_CF_ = 3.2 Hz); ^19^F NMR (376 MHz, *DMSO-d6*) δ −110.60–110.79 (m, 1F), −113.14 (dd, *J* = 18.0, 8.7 Hz, 1F); HRMS (ESI^+^): *m*/*z* calcd for C_17_H_15_F_2_N_5_ [M + H]^+^ 328.1368, found 328.1352.

(*E*)-2-((1-(2,5-difluorobenzyl)-1*H*-indol-5-yl) methylene) hydrazine-1-carboximidamide (**3E**): Yield: 91.4%; ^1^H NMR (600 MHz, *DMSO-d6*) δ 11.88 (br s, 1H), 8.20 (s, 1H), 7.95 (d, J = 1.2 Hz, 1H), 7.74 (dd, J = 8.7, 1.5 Hz, 1H), 7.55 (d, J = 8.7 Hz, 1H), 7.64 (br s, 3H), 7.51 (d, J = 3.2 Hz, 1H), 7.26 (td, J = 9.3, 4.5 Hz, 1H), 7.15 (ddd, J = 12.2, 8.3, 3.6 Hz, 1H), 6.88 (ddd, J = 8.9, 5.7, 3.2 Hz, 1H), 6.55 (d, J = 3.2 Hz, 1H), 5.49 (s, 2H); ^13^C NMR (151 MHz, *DMSO-d6*) δ158.50 (dd, *J*_CF_ = 240.6, 2.0 Hz), 156.51 (dd, *J*_CF_ = 241.6, 2.2 Hz), 155.88, 148.58, 137.30, 130.66, 128.50, 127.19 (dd, *J*_CF_ = 17.9, 7.5 Hz), 125.71, 122.12, 120.95, 117.59 (dd, *J*_CF_ = 24.3, 8.8 Hz), 116.60 (d, *J*_CF_ = 24.1 Hz), 116.27 (dd, *J*_CF_ = 24.9, 4.4 Hz), 110.82, 102.89, 43.61; ^19^F NMR (376 MHz, *DMSO-d6*) δ −118.35 (dtd, *J* = 13.0, 8.4, 4.5 Hz, 1F), −122.96 (ddt, *J* = 18.5, 9.3, 4.7 Hz, 1F); HRMS (ESI^+^): *m*/*z* calcd for C_17_H_15_F_2_N_5_ [M + H]^+^ 328.1368, found 328.1360.

(*E*)-2-((1-(2,6-difluorobenzyl)-1*H*-indol-5-yl) methylene) hydrazine-1-carboximidamide (**3F**): Yield: 98.7%; ^1^H NMR (600 MHz, *DMSO-d6*) δ 11.93 (br s, 1H), 8.19 (s, 1H), 7.93 (d, J = 1.1 Hz, 1H), 7.81 (br s, 3H), 7.77 (dd, J = 8.7, 1.4 Hz, 1H), 7.54 (d, J = 8.7 Hz, 1H), 7.42 (td, J = 8.4, 4.2 Hz, 1H), 7.38 (d, J = 3.1 Hz, 1H), 7.15–7.09 (m, 2H), 6.50 (dd, J = 3.2, 0.4 Hz, 1H), 5.46 (s, 2H); ^13^C NMR (151 MHz, *DMSO-d6*) δ161.23 (dd, *J*_CF_ = 248.1, 7.9 Hz, 2C), 155.80, 148.56, 137.12, 131.49 (t, *J*_CF_ = 10.6 Hz), 130.41, 128.37, 125.55, 122.11, 120.93, 113.27 (t, *J*_CF_ = 19.5 Hz), 112.40 (dd, *J*_CF_ = 20.8, 4.6 Hz, 2C), 110.34, 102.81, 37.71; ^19^F NMR (376 MHz, *DMSO-d6*) δ −114.07 (t, *J* = 7.0 Hz, 2F); HRMS (ESI^+^): *m*/*z* calcd for C_17_H_15_F_2_N_5_ [M + H]^+^ 328.1368, found 328.1361.

(*E*)-2-((1-(3,4-difluorobenzyl)-1*H*-indol-5-yl) methylene) hydrazine-1-carboximidamide (**3G**): Yield: 98.0%; ^1^H NMR (600 MHz, *DMSO-d6*) δ 11.93 (br s, 1H), 8.20 (s, 1H), 7.95 (d, J = 1.0 Hz, 1H), 7.78 (br s, 3H), 7.72 (dd, J = 8.7, 1.4 Hz, 1H), 7.58 (d, J = 3.2 Hz, 1H), 7.55 (d, J = 8.7 Hz, 1H), 7.33 (ddd, J = 11.6, 9.3, 5.2 Hz, 2H), 7.05–7.00 (m, 1H), 6.54 (d, J = 3.1 Hz, 1H), 5.43 (s, 2H); ^13^C NMR (151 MHz, *DMSO-d6*) δ 155.81, 149.71 (dd, *J*_CF_ = 246.3, 12.9 Hz), 149.15 (dd, *J*_CF_ = 245.4, 12.4 Hz), 148.67, 137.25, 136.20 (dd, *J*_CF_ = 5.2, 3.8 Hz), 130.68, 128.63, 125.55, 124.44 (dd, *J*_CF_ = 6.6, 3.3 Hz), 122.14, 120.87, 118.10 (d, *J*_CF_ = 17.2 Hz), 116.78 (d, *J*_CF_ = 17.4 Hz), 111.02, 102.75, 48.52; ^19^F NMR (376 MHz, *DMSO-d6*) δ −138.31–138.48 (m, 1F), −140.33–140.51 (m, 1F); HRMS (ESI^+^): *m*/*z* calcd for C_17_H_15_F_2_N_5_ [M + H]^+^ 328.1368, found 328.1359.

(*E*)-2-((1-(3,5-difluorobenzyl)-1*H*-indol-5-yl) methylene) hydrazine-1-carboximidamide (**3H**): Yield: 97.0%; ^1^H NMR (600 MHz, *DMSO-d6*) δ 11.89 (br s, 1H), 8.20 (s, 1H), 7.96 (s, 1H), 7.77 (br s, 3H), 7.72 (dd, J = 8.7, 1.2 Hz, 1H), 7.59 (d, J = 3.2 Hz, 1H), 7.54 (d, J = 8.7 Hz, 1H), 7.10 (tt, J = 9.3, 2.2 Hz, 1H), 6.92–6.86 (m, 2H), 6.56 (d, J = 3.1 Hz, 1H), 5.48 (s, 2H); ^13^C NMR (151 MHz, *DMSO-d6*) δ162.85 (dd, *J*_CF_ = 246.8, 13.3 Hz, 2C), 155.92, 148.55, 143.11 (t, *J*_CF_ = 8.7 Hz), 137.29, 130.79, 128.62, 125.73, 122.11, 120.96, 110.98, 110.68 (dd, *J*_CF_ = 20.2, 5.1 Hz, 2C), 103.37 (t, *J*_CF_ = 25.8 Hz), 102.86, 48.73; ^19^F NMR (376 MHz, *DMSO-d6*) δ −109.43–109.52 (m, 2F); HRMS (ESI^+^): *m*/*z* calcd for C_17_H_15_F_2_N_5_ [M + H]^+^ 328.1368, found 328.1355.

(*E*)-2-((1-(3-(trifluoromethyl) benzyl)-1*H*-indol-5-yl) methylene) hydrazine-1-carboximidamide (**3I**): Yield: 92.2%; ^1^H NMR (600 MHz, *DMSO-d6*) δ 11.94 (br s, 1H), 8.19 (s, 1H), 7.95 (s, 1H), 7.72 (d, J = 8.7 Hz, 1H), 7.64 (br s, 3H), 7.60 (t, J = 5.9 Hz, 3H), 7.56–7.50 (m, 2H), 7.44 (d, J = 7.7 Hz, 1H), 6.56 (d, J = 3.0 Hz, 1H), 5.56 (s, 2H); ^13^C NMR (151 MHz, *DMSO-d6*) δ 155.98, 148.52, 140.00, 137.29, 131.58, 130.72, 130.16, 129.67 (q, *J*_CF_ = 31.4 Hz), 128.61, 125.72, 124.64 (d, *J*_CF_ = 3.6 Hz), 124.51 (d, *J*_CF_ = 272.4 Hz), 124.06 (q, *J*_CF_ = 3.6 Hz), 122.14, 120.83, 110.96, 102.80, 48.98; ^19^F NMR (376 MHz, *DMSO-d6*) δ −61.15 (s, 3F); HRMS (ESI^+^): *m*/*z* calcd for C_18_H_16_F_3_N_5_ [M + H]^+^360.1431, found 360.1415.

(*E*)-2-((1-(4-(trifluoromethyl) benzyl)-1*H*-indol-5-yl) methylene) hydrazine-1-carboximidamide (**3J**): Yield: 57.9%; ^1^H NMR (600 MHz, *DMSO-d6*) δ 11.91 (br s, 1H), 8.20 (s, 1H), 7.97 (d, J = 1.0 Hz, 1H), 7.71 (dd, J = 8.7, 1.4 Hz, 1H), 7.68 (br s, 3H), 7.65 (d, J = 8.2 Hz, 2H), 7.58 (d, J = 3.1 Hz, 1H), 7.49 (d, J = 8.7 Hz, 1H), 7.34 (d, J = 8.1 Hz, 2H), 6.57 (d, J = 3.0 Hz, 1H), 5.56 (s, 2H); ^13^C NMR (151 MHz, *DMSO-d6*) δ 155.80, 148.68, 143.30, 137.39, 130.86, 128.63, 128.46 (d, *J*_CF_ = 31.7 Hz), 128.06 (2C), 125.93 (q, *J*_CF_ = 3.7 Hz,2C), 125.58, 124.60 (d, *J*_CF_ = 272.1 Hz), 122.17, 120.89, 110.99, 102.80, 49.08; ^19^F NMR (376 MHz, *DMSO-d6*) δ −60.97 (s, 3F); HRMS (ESI^+^): *m*/*z* calcd for C_18_H_16_F_3_N_5_ [M + H]^+^ 360.1431, found 360.1443.

(*E*)-2-((1-(3,5-bis(trifluoromethyl) benzyl)-1*H*-indol-5-yl) methylene) hydrazine-1-carboximidamide (**3K**): Yield: 93.0%; ^1^H NMR (600 MHz, *DMSO-d6*) δ 11.88 (br s, 1H), 8.19 (s, 1H), 7.98 (s, 1H), 7.95 (d, J = 0.9 Hz, 1H), 7.91 (s, 2H), 7.75 (dd, J = 8.7, 1.4 Hz, 1H), 7.70 (br s, 3H), 7.68 (d, J = 3.2 Hz, 1H), 7.61 (d, J = 8.7 Hz, 1H), 6.58 (d, J = 3.1 Hz, 1H), 5.67 (s, 2H); ^13^C NMR (151 MHz, *DMSO-d6*) δ 155.93, 148.49, 142.02, 137.22, 130.86 (q, *J*_CF_ = 32.9 Hz,2C), 130.63, 128.64, 128.42 (d, *J*_CF_ = 3.7 Hz,2C), 125.88, 123.60 (d, *J*_CF_ = 272.9 Hz,2C), 122.28, 121.88–121.68 (m), 120.96, 110.89, 103.17, 48.48; ^19^F NMR (376 MHz, *DMSO-d6*) δ −61.44 (s, 6F); HRMS (ESI^+^): *m*/*z* calcd for C_19_H_15_F_6_N_5_ [M + H]^+^ 428.1304, found 428.1286.

(*E*)-2-((1-(2-chlorobenzyl)-1*H*-indol-5-yl) methylene) hydrazine-1-carboximidamide (**3L**): Yield: 84.0%; ^1^H NMR (600 MHz, *DMSO-d6*) δ 8.19 (s, 1H), 7.96 (d, J = 0.9 Hz, 1H), 7.70 (dd, J = 8.7, 1.4 Hz, 1H), 7.59 (br s, 4H), 7.50–7.46 (m, 2H), 7.44 (d, J = 8.7 Hz, 1H), 7.28 (td, J = 7.8, 1.5 Hz, 1H), 7.20 (td, J = 7.6, 1.0 Hz, 1H), 6.70–6.67 (m, 1H), 6.57 (d, J = 3.2 Hz, 1H), 5.52 (s, 2H); ^13^C NMR (151 MHz, *DMSO-d6*) δ 156.26, 148.26, 137.45, 135.63, 132.25, 130.82, 129.94, 129.74, 128.88, 128.53, 127.95, 125.97, 121.97, 120.85, 110.89, 102.76, 47.48; HRMS (ESI^+^): *m*/*z* calcd for C_17_H_16_ClN_5_ [M + H]^+^ 326.1167, found 326.1181.

(*E*)-2-((1-(3-chlorobenzyl)-1*H*-indol-5-yl) methylene) hydrazine-1-carboximidamide (**3M**): Yield: 34.7%; ^1^H NMR (600 MHz, *DMSO-d6*) δ 11.92 (br s, 1H), 8.20 (s, 1H), 7.96 (d, J = 1.0 Hz, 1H), 7.77 (br s, 3H), 7.72 (dd, J = 8.7, 1.4 Hz, 1H), 7.58 (d, J = 3.2 Hz, 1H), 7.53 (d, J= 8.7 Hz, 1H), 7.33–7.24 (m, 3H), 7.14 (d, J = 7.3 Hz, 1H), 6.55 (d, J = 3.1 Hz, 1H), 5.46 (s, 2H); ^13^C NMR (151 MHz, *DMSO-d6*) δ 155.77, 148.71, 141.01, 137.34, 133.61, 130.93, 130.80, 128.60, 127.84, 127.32, 126.19, 125.51, 122.18, 120.86, 111.03, 102.73, 48.93; HRMS (ESI^+^): *m*/*z* calcd for C_17_H_16_ClN_5_ [M + H]^+^ 326.1167, found 326.1162.

(*E*)-2-((1-(4-chlorobenzyl)-1*H*-indol-5-yl) methylene) hydrazine-1-carboximidamide (**3N**): Yield: 70%; ^1^H NMR (600 MHz, *DMSO-d6*) δ 11.92 (br s, 1H), 8.20 (s, 1H), 7.95 (d, J = 1.0 Hz, 1H), 7.78 (br s, 3H), 7.70 (dd, J = 8.7, 1.4 Hz, 1H), 7.55 (d, J = 3.2 Hz, 1H), 7.50 (d, J = 8.7 Hz, 1H), 7.36–7.32 (m, 2H), 7.20 (d, J = 8.5 Hz, 2H), 6.54 (d, J = 3.1 Hz, 1H), 5.44 (s, 2H); ^13^C NMR (151 MHz, *DMSO-d6*) δ 155.78, 148.73, 137.48, 137.33, 132.47,130.75,129.36(2C), 128.97(2C),128.62,125.45,122.15, 120.80, 111.03, 102.64, 48.88; HRMS (ESI^+^): *m*/*z* calcd for C_17_H_16_ClN_5_ [M + H]^+^ 326.1167, found 326.1168.

(*E*)-2-((1-(2,4-dichlorobenzyl)-1*H*-indol-5-yl) methylene) hydrazine-1-carboximidamide (**3O**): Yield: 67.2%; ^1^H NMR (600 MHz, *DMSO-d6*) δ 11.92 (br s, 1H), 8.21 (s, 1H), 7.98 (d, J = 1.0 Hz, 1H), 7.72 (dd, J = 8.7, 1.4 Hz, 1H), 7.69 (br s, 3H), 7.65 (d, J = 2.1 Hz, 1H), 7.47 (d, J = 3.2 Hz, 1H), 7.45 (d, J = 8.7 Hz, 1H), 7.30 (dd, J = 8.4, 2.1 Hz, 1H), 6.66 (d, J = 8.4 Hz, 1H), 6.58 (d, J = 3.1 Hz, 1H), 5.51 (s, 2H); ^13^C NMR (151 MHz, *DMSO-d6*) δ 155.77, 148.67, 137.51, 134.89, 133.36, 133.20, 130.84, 130.11, 129.42, 128.53, 128.14, 125.68, 122.25, 120.99, 110.92, 102.98, 47.06; HRMS (ESI^+^): *m*/*z* calcd for C_17_H_15_Cl_2_N_5_ [M + H]^+^360.0777, found 360.0763.

(*E*)-2-((1-(3,4-dichlorobenzyl)-1*H*-indol-5-yl) methylene) hydrazine-1-carboximidamide (**3P**): Yield: 31.5%; ^1^H NMR (600 MHz, *DMSO-d6*) δ 8.19 (s, 1H), 7.95 (d, J = 1.0 Hz, 1H), 7.72 (dd, J = 8.7, 1.4 Hz, 1H), 7.64 (br s, 4H), 7.58 (d, J = 3.2 Hz, 1H), 7.54 (d, J = 8.3 Hz, 2H), 7.49 (d, J = 1.9 Hz, 1H), 7.13 (dd, J = 8.3, 2.0 Hz, 1H), 6.55 (d, J = 3.2 Hz, 1H), 5.45 (s, 2H); ^13^C NMR (151 MHz, *DMSO-d6*) δ 155.91, 148.56, 139.64, 137.24, 131.57, 131.24, 130.71, 130.50, 129.56, 128.63, 127.85, 125.69, 122.13, 120.91, 110.99, 102.84, 48.36; HRMS (ESI^+^): *m*/*z* calcd for C_17_H_15_Cl_2_N_5_ [M + H]^+^ 360.0777, found 360.0775.

(E)-2-((1-(2-bromobenzyl)-1*H*-indol-5-yl) methylene) hydrazine-1-carboximidamide (**3Q**): Yield: 95.0%; ^1^H NMR (600 MHz, *DMSO-d6*) δ 11.93 (br s, 1H), 8.21 (s, 1H), 7.99 (d, J = 1.1 Hz, 1H), 7.72 (dd, J = 8.7, 1.4 Hz, 1H), 7.69 (br s, 3H), 7.65 (dd, J = 7.7, 1.4 Hz, 1H), 7.47 (d, J = 3.2 Hz, 1H), 7.43 (d, J = 8.7 Hz, 1H), 7.25–7.18 (m, 2H), 6.58 (dd, J = 6.5, 2.5 Hz, 2H), 5.49 (s, 2H); ^13^C NMR (151 MHz, *DMSO-d6*) δ 155.78, 148.70, 137.57, 137.15, 133.20, 130.88, 129.98, 128.77, 128.53, 128.51, 125.62, 122.40, 122.25, 120.94, 110.95, 102.86, 49.90; HRMS (ESI^+^): *m*/*z* calcd for C_17_H_16_BrN_5_ [M + H]^+^370.0662, found 370.0645.

(*E*)-2-((1-(3-bromobenzyl)-1*H*-indol-5-yl) methylene) hydrazine-1-carboximidamide (**3R**): Yield: 17.8%; ^1^H NMR (600 MHz, *DMSO-d6*) δ 11.87 (br s, 1H), 8.20 (s, 1H), 7.95 (d, J = 1.1 Hz, 1H), 7.72 (dd, J = 8.7, 1.4 Hz, 1H), 7.64 (br s, 3H), 7.58 (d, J = 3.2 Hz, 1H), 7.53 (d, J = 8.7 Hz, 1H), 7.44–7.39 (m, 2H), 7.25 (t, J = 7.8 Hz, 1H), 7.17 (d, J = 7.8 Hz, 1H), 6.55 (d, J = 3.1 Hz, 1H), 5.45 (s, 2H); ^13^C NMR (151 MHz, *DMSO-d6*) δ 155.76, 148.72, 141.26, 137.33, 131.22, 130.79, 130.74, 130.21, 128.59, 126.58, 125.51, 122.23, 122.19, 120.85, 111.03, 102.73, 48.87; HRMS (ESI^+^): *m*/*z* calcd for C_17_H_16_BrN_5_ [M + H]^+^ 370.0662, found 370.0645.

(*E*)-2-((1-(4-bromobenzyl)-1*H*-indol-5-yl) methylene) hydrazine-1-carboximidamide (**3S**): Yield: 37.35%; ^1^H NMR (600 MHz, *DMSO-d6*) δ 11.91 (br s, 1H), 8.20 (s, 1H), 7.95 (d, J = 1.0 Hz, 1H), 7.70 (dd, J = 8.7, 1.4 Hz, 1H), 7.66 (br s, 3H), 7.54 (d, J = 3.2 Hz, 1H), 7.51–7.46 (m, 3H), 7.13 (d, J = 8.4 Hz, 2H), 6.54 (d, J = 3.1 Hz, 1H), 5.42 (s, 2H); ^13^C NMR (151 MHz, *DMSO-d6*) δ 155.76, 148.74, 137.91, 137.33, 131.90(2C), 130.77, 129.70(2C), 128.61, 125.45, 122.15, 120.98, 120.81, 111.03, 102.64, 48.94; HRMS (ESI^+^): *m*/*z* calcd for C_17_H_16_BrN_5_ [M + H]^+^ 370.0662, found 370.0644.

(*E*)-2-((1-(2-chloro-4-fluorobenzyl)-1*H*-indol-5-yl) methylene) hydrazine-1-carboximidamide (**3T**): Yield: 69.8%; ^1^H NMR (600 MHz, *DMSO-d6*) δ 11.92 (br s, 1H), 8.21 (s, 1H), 7.97 (d, J = 1.1 Hz, 1H), 7.72 (dd, J = 8.7, 1.4 Hz, 1H), 7.65 (br s, 3H), 7.50–7.45 (m, 3H), 7.11 (td, J = 8.5, 2.6 Hz, 1H), 6.78 (dd, J = 8.7, 6.1 Hz, 1H), 6.57 (d, J = 3.1 Hz, 1H), 5.50 (s, 2H); ^13^C NMR (151 MHz, *DMSO-d6*) δ161.69 (d, *J*_CF_ = 247.4 Hz), 155.90, 148.57, 137.46, 133.12 (d, *J*_CF_ = 10.8 Hz), 132.04 (d, *J*_CF_ = 3.4 Hz), 130.75, 130.52 (d, *J*_CF_ = 9.1 Hz), 128.53, 125.73, 122.17, 120.93, 117.32 (d, *J*_CF_ = 25.2 Hz), 115.12 (d, *J*_CF_ = 21.2 Hz), 110.92, 102.88, 46.96; ^19^F NMR (376 MHz, *DMSO-d6*) δ −112.63 (dd, *J* = 14.8, 8.4 Hz); HRMS (ESI^+^): *m*/*z* calcd for C_17_H_15_ClFN_5_ [M + H]^+^344.1073, found 344.1061.

(*E*)-2-((1-(3-chloro-4-fluorobenzyl)-1*H*-indol-5-yl) methylene) hydrazine-1-carboximidamide (**3U**): Yield: 47.4%; ^1^H NMR (600 MHz, *DMSO-d6*) δ 11.98 (br s, 1H), 8.19 (s, 1H), 7.94 (d, J = 0.8 Hz, 1H), 7.72 (dd, J = 8.7, 1.4 Hz, 1H), 7.66 (br s, 3H), 7.58 (d, J = 3.1 Hz, 1H), 7.56 (d, J = 8.7 Hz, 1H), 7.47 (dd, J = 7.1, 2.1 Hz, 1H), 7.33 (t, J = 9.0 Hz, 1H), 7.20 (ddd, J = 8.4, 4.6, 2.2 Hz, 1H), 6.54 (d, J = 3.2 Hz, 1H), 5.43 (s, 2H); ^13^C NMR (151 MHz, *DMSO-d6*) δ156.93 (d, *J*_CF_ = 246.4 Hz), 155.86, 148.62, 137.21, 136.38 (d, *J*_CF_ = 3.6 Hz), 130.65, 129.72, 128.63, 128.34 (d, *J*_CF_ = 7.5 Hz), 125.63, 122.13, 120.87, 119.89 (d, *J*_CF_ = 17.8 Hz), 117.50 (d, *J*_CF_ = 21.1 Hz), 111.01, 102.78, 48.34; ^19^F NMR (376 MHz, *DMSO-d6*) δ −118.17 (ddd, *J* = 9.3, 7.3, 4.9 Hz); HRMS (ESI^+^): *m*/*z* calcd for C_17_H_15_ClFN_5_ [M + H]^+^ 344.1073, found 344.1059.

(*E*)-2-((1-(4-cyanobenzyl)-1*H*-indol-5-yl) methylene) hydrazine-1-carboximidamide (**3V**): Yield: 34.6%; ^1^H NMR (600 MHz, *DMSO-d6*) δ 8.06 (s, 1H), 7.77–7.73 (m, 3H), 7.56 (dd, J = 8.6, 1.4 Hz, 1H), 7.47 (d, J = 3.1 Hz, 1H), 7.34 (d, J = 8.6 Hz, 1H), 7.28 (d, J = 8.3 Hz, 2H), 6.50–6.48 (m, 1H), 5.51 (s, 2H); ^13^C NMR (151 MHz, *DMSO-d6*) δ 160.10, 145.58, 144.46, 136.26, 132.96 (2C), 130.09, 129.19, 128.77, 128.13 (2C), 120.48, 119.97, 119.12, 110.55, 110.54, 102.38, 49.15; HRMS (ESI^+^): *m*/*z* calcd for C_18_H_16_N_6_ [M + H]^+^ 317.1509, found 317.1506.

(*Z*)-2-((1-(2-chlorobenzyl)-1*H*-indol-3-yl) methylene) hydrazine-1-carboximidamide (**4L**). Yield: 73.6%; ^1^H NMR (600 MHz, *DMSO-d6*) δ 11.74 (br s, 1H), 8.34 (d, *J* = 5.9 Hz, 2H), 7.96 (s, 1H), 7.50 (dd, *J* = 8.0, 0.9 Hz, 1H), 7.44 (d, *J* = 8.2 Hz, 1H),7.42 (br s, 3H), 7.31 (td, *J* = 7.8, 1.5 Hz, 1H), 7.22 (ddd, *J* = 13.1, 7.2, 0.9 Hz, 2H), 7.19–7.14 (m, 1H), 6.85–6.80 (m, 1H), 5.54 (s, 2H); ^13^C NMR (151 MHz, *DMSO-d6*) δ 155.29, 144.62, 137.49, 135.26, 134.93, 132.46, 130.05, 129.98, 129.21, 128.05, 124.86, 123.66, 123.24, 121.71, 111.02, 110.89, 47.71; HRMS(ESI^+^): *m*/*z* calcd for C_17_H_16_ClN_5_[M+H]^+^ 326.1167, found 326.1183.

(*Z*)-2-((1-(3-chlorobenzyl)-1*H*-indol-3-yl) methylene) hydrazine-1-carboximidamide (**4M**). Yield: 97.1%; ^1^H NMR (600 MHz, *DMSO-d6*) δ 11.83 (br s, 1H), 8.35 (s, 1H), 8.32 (d, J = 7.9 Hz, 1H), 8.07 (s, 1H), 8.00–7.10 (br s, 3H), 7.51 (d, J = 8.2 Hz, 1H), 7.34–7.29 (m, 3H), 7.24–7.20 (m, 1H), 7.19–7.14 (m, 2H), 5.47 (s, 2H); ^13^C NMR (151 MHz, *DMSO-d6*) δ 155.31, 144.57, 140.38, 137.27, 135.14, 133.69, 131.02, 128.03, 127.42, 126.28, 124.94, 123.59, 123.23, 121.67, 111.01, 110.95, 49.12; HRMS(ESI^+^): *m*/*z* calcd for C_17_H_16_ClN_5_[M+H]^+^ 326.1167, found 326.1170.

(*Z*)-2-((1-(4-chlorobenzyl)-1*H*-indol-3-yl) methylene) hydrazine-1-carboximidamide (**4N**). Yield: 76.9%; ^1^H NMR (600 MHz, *DMSO-d6*) δ 11.75 (br s, 1H), 8.33 (d, *J* = 1.7 Hz, 1H), 8.30 (d, *J* = 8.9 Hz, 1H), 8.05 (d, *J* = 1.7 Hz, 1H), 7.40 (br s, 3H), 7.49 (d, *J* = 8.2 Hz, 1H), 7.38–7.34 (m, 2H), 7.26–7.23 (m, 2H), 7.23–7.18 (m, 1H), 7.17–7.13 (m, 1H), 5.45 (s, 2H); ^13^C NMR (151 MHz, *DMSO-d6*) δ 155.26, 144.61, 137.27, 136.85, 135.11, 132.66, 129.49 (2C), 129.07 (2C), 124.97, 123.53, 123.18, 121.62, 111.04, 110.88, 49.08; HRMS(ESI^+^): *m*/*z* calcd for C_17_H_16_ClN_5_[M+H]^+^ 326.1167, found 326.1168.

(*Z*)-2-((1-(2,4-dichlorobenzyl)-1*H*-indol-3-yl) methylene) hydrazine-1-carboximidamide (**4O**). Yield: 82.0%; ^1^H NMR (600 MHz, *DMSO-d6*) δ 11.74 (s, 1H), 8.33 (d, *J* = 9.1 Hz, 2H), 7.95 (s, 1H), 7.68 (d, *J* = 2.1 Hz, 1H), 7.47 (br s, 3H), 7.44 (d, *J* = 8.2 Hz, 1H), 7.34 (dd, *J* = 8.4, 2.1 Hz, 1H), 7.23 (t, *J* = 8.0 Hz, 1H), 7.17 (t, *J* = 7.4 Hz, 1H), 6.82 (d, *J* = 8.4 Hz, 1H), 5.52 (s, 2H); ^13^C NMR (151 MHz, *DMSO-d6*) δ 155.27, 144.59, 137.42, 135.17, 134.17, 133.61, 133.46, 130.53, 129.54, 128.24, 124.87, 123.73, 123.28, 121.79, 111.14, 110.86, 47.27; HRMS(ESI^+^): *m*/*z* calcd for C_17_H_15_Cl_2_N_5_[M+H]^+^ 360.0777, found 360.0781.

(*Z*)-2-((1-(3,4-dichlorobenzyl)-1*H*-indol-3-yl) methylene) hydrazine-1-carboximidamide (**4P**). Yield: 78.0%; ^1^H NMR (600 MHz, *DMSO-d6*) δ 11.75 (br s, 1H), 8.33 (s, 1H), 8.31 (d, *J* = 7.9 Hz, 1H), 8.07 (s, 1H), 7.57–7.50 (m, 3H), 7.41 (br s, 3H), 7.25–7.20 (m, 1H), 7.19–7.13 (m, 2H), 5.47 (s, 2H); ^13^C NMR (151 MHz, *DMSO-d6*) δ 155.27, 144.57, 139.00, 137.21, 135.06, 131.65, 131.33, 130.71, 129.71, 127.97, 124.95, 123.65, 123.25, 121.72, 111.07, 110.98, 48.55; HRMS(ESI^+^): *m*/*z* calcd for C_17_H_15_Cl_2_N_5_[M+H]^+^ 360.0777, found 360.0774.

(*Z*)-2-((1-(2-chlorobenzyl)-1*H*-pyrrolo[2,3-*b*]pyridin-5-yl) methylene) hydrazine-1-carboximidamide (**5L**). Yield: 94.9%; ^1^H NMR (600 MHz, *DMSO-d6*) δ 11.98 (br s, 1H), 8.69 (d, *J* = 1.8 Hz, 1H), 8.52 (d, *J* = 1.8 Hz, 1H), 8.28 (s, 1H), 7.82 (br s, 1H), 7.64 (d, *J* = 3.5 Hz, 1H), 7.48 (d, *J* = 7.9 Hz, 1H), 7.29 (td, *J* = 7.9, 1.3 Hz, 1H), 7.21 (t, *J* = 7.6 Hz, 1H), 6.76 (d, *J* = 7.7 Hz, 1H), 6.61 (d, *J* = 3.5 Hz, 1H), 5.57 (s, 2H); ^13^C NMR (151 MHz, *DMSO-d6*) δ 155.88, 148.40, 146.21, 143.69, 135.67, 132.21, 131.27, 129.87, 129.72, 129.10, 127.97, 127.91, 122.78, 120.26, 101.13, 45.73; HRMS(ESI^+^): *m*/*z* calcd for C_16_H_15_ClN_6_[M+H]^+^ 327.1119, found 326.1133.

(*Z*)-2-((1-(3-chlorobenzyl)-1*H*-pyrrolo[2,3-*b*]pyridin-5-yl) methylene) hydrazine-1-carboximidamide (**5M**). Yield: 91.7%; ^1^H NMR (600 MHz, *DMSO-d6*) δ 12.20 (s, 1H), 8.74 (s, 1H), 8.52 (s, 1H), 8.29 (s, 1H), 8.3–7.4 (br s, 3H), 7.74 (s, 1H), 7.30 (d, J = 11.8 Hz, 3H), 7.19 (d, J = 6.3 Hz, 1H), 6.58 (s, 1H), 5.50 (s, 2H); ^13^C NMR (151 MHz, *DMSO-d6*) δ 155.96, 147.82, 145.99, 143.25, 140.94, 133.54, 131.27, 130.95, 128.34, 127.89, 127.67, 126.53, 122.71, 120.54, 101.22, 47.35; HRMS(ESI^+^): *m*/*z* calcd for C_16_H_15_ClN_6_[M+H]^+^ 327.1119, found 326.1131.

(*Z*)-2-((1-(4-chlorobenzyl)-1*H*-pyrrolo[2,3-*b*] pyridin-5-yl) methylene) hydrazine-1-carboximidamide (**5N**). Yield: 92.5%; ^1^H NMR (600 MHz, *DMSO-d_6_*) δ 12.02 (s, 1H), 8.72 (d, *J* = 1.7 Hz, 1H), 8.49 (d, *J* = 1.7 Hz, 1H), 8.28 (s, 1H), 7.89 (br s, 3H), 7.70 (d, *J* = 3.5 Hz, 1H), 7.34 (d, *J* = 8.4 Hz, 2H), 7.25 (d, *J* = 8.4 Hz, 2H), 6.57 (d, *J* = 3.5 Hz, 1H), 5.47 (s, 2H); ^13^C NMR (151 MHz, *DMSO-d_6_*) δ 155.84, 148.20, 146.25, 143.60, 137.54, 132.51, 131.10, 129.69 (2C), 128.96 (2C), 127.97, 122.60, 120.33, 101.01, 47.17; HRMS(ESI^+^): *m*/*z* calcd for C_16_H_15_ClN_6_[M+H]^+^ 327.1119, found 326.1129.

(*Z*)-2-((1-(2,4-dichlorobenzyl)-1*H*-pyrrolo[2,3-*b*] pyridin-5-yl) methylene) hydrazine-1-carboximidamide (**5O**). Yield: 93.6%; ^1^H NMR (600 MHz, *DMSO-d6*) δ 12.08 (s, 1H), 8.69 (d, *J* = 1.8 Hz, 1H), 8.53 (d, *J* = 1.9 Hz, 1H), 8.28 (s, 1H), 7.77 (br s, 3H), 7.65 (t, *J* = 2.6 Hz, 2H), 7.31 (dd, *J* = 8.4, 2.1 Hz, 1H), 6.79 (d, *J* = 8.4 Hz, 1H), 6.62 (d, *J* = 3.5 Hz, 1H), 5.55 (s, 2H); ^13^C NMR (151 MHz, *DMSO-d6*) δ 155.87, 148.25, 146.12, 143.63, 134.85, 133.36, 133.20, 131.27, 130.51, 129.35, 128.12, 128.09, 122.83, 120.35, 101.29, 45.38; HRMS(ESI^+^): *m*/*z* calcd for C_16_H_14_Cl_2_N_6_[M+H]^+^ 361.0730, found 361.0737.

(*Z*)-2-((1-(3,4-dichlorobenzyl)-1*H*-pyrrolo[2,3-*b*] pyridin-5-yl) methylene) hydrazine-1-carboximidamide (**5P**). Yield: 94.6%; ^1^H NMR (600 MHz, *DMSO-d6*) δ 12.14 (s, 1H), 8.74 (d, *J* = 1.7 Hz, 1H), 8.51 (d, *J* = 1.8 Hz, 1H), 8.28 (s, 1H), 7.92 (br s, 3H), 7.75 (d, *J* = 3.5 Hz, 1H), 7.54 (dd, *J* = 5.1, 3.2 Hz, 2H), 7.20 (dd, *J* = 8.4, 1.8 Hz, 1H), 6.59 (d, *J* = 3.5 Hz, 1H), 5.49 (s, 2H); ^13^C NMR (151 MHz, *DMSO-d6*) δ 155.92, 147.90, 146.03, 143.39, 139.59, 131.49, 131.25, 131.17, 130.56, 129.92, 128.28, 128.22, 122.77, 120.51, 101.26, 46.80; HRMS(ESI^+^): *m*/*z* calcd for C_16_H_14_Cl_2_N_6_[M+H]^+^ 361.0730, found 361.0737.

(*Z*)-2-((4-chloro-1-(2-chlorobenzyl)-1*H*-pyrrolo[2,3-*b*] pyridin-5-yl) methylene) hydrazine-1-carboximidamide (**6L**). Yield: 57.9%; ^1^H NMR (600 MHz, *DMSO-d6*) δ 12.26 (br s, 1H), 9.09 (s, 1H), 8.59 (s, 1H), 7.80 (br s,3H), 7.72 (d, *J* = 3.5 Hz, 1H), 7.48 (dd, *J* = 8.0, 1.0 Hz, 1H), 7.30 (td, *J* = 7.8, 1.6 Hz, 1H), 7.22 (td, *J* = 7.6, 1.1 Hz, 1H), 6.84 (dd, *J* = 7.7, 1.2 Hz, 1H), 6.67 (d, *J* = 3.5 Hz, 1H), 5.59 (s, 2H); ^13^C NMR (151 MHz, *DMSO-d6*) δ 155.82, 148.04, 143.00, 142.30, 135.25, 134.97, 132.30, 131.67, 129.91, 129.88, 129.38, 127.95, 119.77, 118.95, 99.46, 46.16; HRMS(ESI^+^): *m*/*z* calcd for C_16_H_14_Cl_2_N_6_[M+H]^+^ 361.0730, found 361.0745.

(*Z*)-2-((4-chloro-1-(3-chlorobenzyl)-1*H*-pyrrolo[2,3-*b*] pyridin-5-yl) methylene) hydrazine-1-carboximidamide (**6M**). Yield: 65.6%; ^1^H NMR (600 MHz, *DMSO-d6*) δ 12.22 (br s, 1H), 9.12 (s, 1H), 8.58 (s, 1H), 7.83 (d, *J* = 3.5 Hz, 1H), 7.76 (br s, 3H), 7.36–7.28 (m, 3H), 7.19 (d, *J* = 6.3 Hz, 1H), 6.63 (d, *J* = 3.5 Hz, 1H), 5.50 (s, 2H); ^13^C NMR (151 MHz, *DMSO-d6*) δ 155.98, 147.82, 142.99, 142.18, 140.61, 134.89, 133.56, 131.47, 130.97, 128.00, 127.78, 126.60, 119.79, 119.02, 99.40, 47.69; HRMS(ESI^+^): *m*/*z* calcd for C_16_H_14_Cl_2_N_6_[M+H]^+^361.0730, found 361.0739.

(*Z*)-2-((4-chloro-1-(4-chlorobenzyl)-1*H*-pyrrolo[2,3-*b*] pyridin-5-yl) methylene) hydrazine-1-carboximidamide (**6N**). Yield: 78.1%; ^1^H NMR (600 MHz, *DMSO-d6*) δ 12.25 (br s, 1H), 9.11 (s, 1H), 8.58 (s, 1H), 7.79 (d, *J* = 7.0 Hz, 1H), 7.75 (br s, 3H), 7.35 (d, *J* = 8.4 Hz, 2H), 7.26 (d, *J* = 8.4 Hz, 2H), 6.62 (d, *J* = 7.1 Hz, 1H), 5.49 (s, 2H); ^13^C NMR (151 MHz, *DMSO-d6*) δ 155.94, 147.84, 142.94, 142.22, 137.16, 134.86, 132.64, 131.44, 129.75 (2C), 128.99 (2C), 119.71, 119.00, 99.35, 47.59; HRMS(ESI^+^): *m*/*z* calcd for C_16_H_14_Cl_2_N_6_[M+H]^+^ 361.0730, found 361.0740.

(*Z*)-2-((4-chloro-1-(2,4-dichlorobenzyl)-1*H*-pyrrolo[2,3-*b*] pyridin-5-yl) methylene) hydrazine-1-carboximidamide (**6O**). Yield: 78.0%; ^1^H NMR (600 MHz, *DMSO-d6*) δ 12.23 (br s, 1H), 9.08 (s, 1H), 8.57 (s, 1H), 7.72 (d, *J* = 3.5 Hz, 1H), 7.68 (br s, 3H), 7.65 (d, *J* = 2.1 Hz, 1H), 7.32 (dd, *J* = 8.4, 2.1 Hz, 1H), 6.86 (d, *J* = 8.4 Hz, 1H), 6.66 (d, *J* = 3.5 Hz, 1H), 5.57 (s, 2H); ^13^C NMR (151 MHz, *DMSO-d6*) δ 156.16, 147.91, 143.02, 141.98, 134.85, 134.47, 133.51, 133.29, 131.60, 130.77, 129.38, 128.11, 120.05, 119.00, 99.53, 45.77; HRMS(ESI^+^): *m*/*z* calcd for C_16_H_13_Cl_3_N_6_[M+H]^+^ 395.0340, found 395.0348.

(*Z*)-2-((4-chloro-1-(3,4-dichlorobenzyl)-1*H*-pyrrolo[2,3-*b*] pyridin-5-yl) methylene) hydrazine-1-carboximidamide (**6P**). Yield: 61.3%; ^1^H NMR (600 MHz, *DMSO-d6*) δ 12.23 (br s, 1H), 9.12 (s, 1H), 8.57 (s, 1H), 7.83 (d, *J* = 3.5 Hz, 1H), 7.70 (br s, 3H), 7.57 (d, *J* = 2.0 Hz, 1H), 7.55 (d, *J* = 8.3 Hz, 1H), 7.20 (dd, *J* = 8.3, 2.0 Hz, 1H), 6.63 (d, *J* = 3.5 Hz, 1H), 5.50 (s, 2H); ^13^C NMR (151 MHz, *DMSO-d6*) δ 156.10, 147.76, 143.01, 142.05, 139.22, 134.87, 131.52, 131.40, 131.27, 130.69, 130.04, 128.28, 119.93, 119.07, 99.48, 47.17; HRMS(ESI^+^): *m*/*z* calcd for C_16_H_13_Cl_3_N_6_[M+H]^+^ 395.0340, found 395.0347.

### 3.2. Antibacterial Evaluation

#### 3.2.1. MIC and MBC Testing

The standard microdilution test was used for determining the MICs of novel compounds [40]. Briefly, all compounds were dissolved in DMSO and mixed with Mueller–Hinton (MH) broth. Subsequently, the compounds were two-fold serially diluted in 100 µL in 96-well plates. The ESKAPE pathogens (*E. coli* ATCC25922, *S. aureus* ATCC29223, *K. pneumoniae* ATCC700603, *A. baumannii* ATCC19606, *P. aeruginosa* ATCC27853, and *E. faecium* ATCC35667), methicillin-resistant *Staphylococcus aureus* (MRSA) and clinical *K. pneumoniae* isolates (*K.P.* 2102, *K.P.* 2105, *K.P.* 2107, *K.P.* 2108, *K.P.* 2109, *K.P.* 2112, *K.P.* 2118, *K.P.* 2125, *K.P.* 2134, *K.P.* 2135, and *K.P.* 2138) were grown in MH broth overnight at 37 °C with shaking until the mid-logarithmic growth phase. The bacteria were diluted to 10^6^ CFU/mL in MH broth media. A volume of 100 µL of the bacterial solution was added in triplicate to the wells of a 96-well plate containing different concentrations of the test compound. The MIC values were recorded after 18 h of incubation at 37 °C. The MIC of the compounds was recorded as the lowest concentration at which no visible growth was observed by optical density (OD) measurement at 600 nm. Subsequently, the MBC values were also determined (only for the compounds with MIC values of <64 µg/mL). Colistin was used as a positive control and DMSO (0.1%) was used as a negative control.

#### 3.2.2. Growth Curve and Bactericidal Time-Kill Kinetics Assay

The growth and time-dependent killing abilities of **4P** were evaluated for the MDR clinical *K. pneumoniae* isolate *K.P.* 2108. *K.P.* 2108 was grown in MH broth overnight until the mid-logarithmic growth phase and diluted to 10^6^ CFU/mL in MH broth media. Different concentrations of **4P** were added to the diluted bacterial solution in assay plates (final concentration of 2, 4, 8, and 16 µg/mL). Colistin (32 µg/mL) was used as a drug control. Blank medium and untreated bacterial solutions were used as negative and positive controls, respectively. The OD_600_ values of the solutions at different incubation times (0, 1, 2, 3, 4, 6, 8, 10, 12 and 24 h) were measured.

*K.P.* 2108 was grown in MH broth until the mid-logarithmic growth phase and diluted to 10^7^ CFU/mL in MH broth media. Different concentrations of **4P** (4, 8, 16, and 32 µg/mL) and colistin (32 µg/mL) were inoculated with the aliquoted bacteria resuspended in fresh medium. After specified time intervals (0, 1, 2, 3, 4, 6, 8, 10, 12, and 24 h), 100 µL aliquots were subjected to serial dilution to concentrations ranging from 10^−1^ to 10^−9^ in 0.9% saline solution. The resultant dilutions were plated on sterile MH agar plates and incubated at 37 °C for 24 h. The viable colonies were counted and represented as log_10_ (CFU/mL).

### 3.3. Mode of Action Study

#### 3.3.1. FC Analysis

The LIVE/DEAD Baclight Bacterial Viability kit (Invitrogen, Carlsbad, CA, USA) was used to evaluate the bacterial viability after treatment with **4P**. *K.P.* 2108 was incubated until the mid-logarithmic phase, after which it was washed thrice with PBS and resuspended to an OD_600_ of 0.5. The bacteria were treated with **4P** (4, 8, 16, 32 µg/mL) and colistin (32 µg/mL), and were harvested for staining after the completion of the treatment. After an additional incubation for 10 min at 37 °C, protected from light, the stained bacteria were harvested and immediately analyzed using a flow cytometer (BD), with the acquisition of 300,000 events. The data were processed with FlowJo software v10.8.1(FlowJo, LLC, Ashland, OR, USA).

#### 3.3.2. Scanning Electron Microscopy (SEM) Analysis

The bacteria were treated in the same manner as the FC samples. After incubation with **4P** (32 µg/mL) and colistin (32 µg/mL), the bacteria were washed thrice with PBS and fixed with 2.5% glutaraldehyde fixation buffer. SEM observation was performed after dehydration with gradient alcohol and gold spraying.

#### 3.3.3. Cytoplasmic Membrane Depolarization Assay

The ability of **4P** to depolarize cytoplasmic membranes was evaluated by using the membrane potential-sensitive fluorescent dye 3,3′-dipropylthiadicarbocyanine iodide (DiSC_3_(5)), following the previous method [41]. In brief, *K.P.* 2108 was cultured overnight at 37 °C until the mid-logarithmic phase, after which it was washed thrice with PBS and resuspended to an OD_600_ of 0.5. Then, DiSC_3_(5) (1 µM) was added to the suspension. After incubation for 20 min at 37 °C, an aliquot (190 µL) of dilution was transferred to a 96-well microtiter plate, and different concentrations of **4P** (10 µL) were added to each well to achieve final concentrations ranging from 4 to 32 µg/mL. Wells containing only the suspension served as a blank control. The fluorescence leakage (F_L_) was defined by the following equation:

F_L_ = (F_F_ − F_B_) − (F_I_ − F_B_)

F_F_: the final fluorescence intensity in the assay medium after 30 minutes of treatment with **4P**;

F_I_: the initial fluorescence intensity of the bacterial suspension;

F_B_: the fluorescence intensity of the blank.

#### 3.3.4. Membrane Integrity Assay

An overnight culture of *K.P.* 2108 was washed and resuspended to obtain an OD_600_ of 0.5 with PBS, and different concentrations of **4P** (4, 8, 16, and 32 µg/mL) were added for treatment. Samples were collected at 1, 2 and 3 h. After washing with PBS, 5 µM pf PI was added and incubated at 37 °C while protected from light. After 20 min, the unbound PI was removed with PBS. The excitation wavelength was 535 nm, and fluorescence detection was at 615 nm.

#### 3.3.5. Propensity of Bacterial Resistance Development

According to the methods used in previous studies [42], the propensity for the development of bacterial resistance in *K. pneumoniae* ATCC700603 and *K.P.* 2112 towards **4P** and colistin was investigated. Firstly, the initial MIC values of **4P** and colistin were determined, and the compounds were challenged repeatedly at the 0.5MIC level. Then, serial passage was initiated by transferring a microbial suspension that had been grown at a sub-MIC of **4P** or colistin (at 0.5MIC) for another MIC assay. After a 20 h incubation period, the bacteria were transferred and assayed for MIC once more. The process was repeated for 18 passages, and the MICs for test compounds were assayed at every passage as described above.

#### 3.3.6. Predicted Binding Mode of **4P** in DHFR

The possible mechanism underlying the bactericidal effect of **4P** was investigated through a preliminary docking study. Molecular docking of **4P** into the *K. pneumoniae* dihydrofolate reductase (DHFR, PDB: 4OR7) was carried out using the Discovery Studio (version 4.0) via the graphical user interface DS-CDOCKER protocol. The 3D structure of 4OR7 used in docking study was downloaded from the Protein Data Bank (https://www.rcsb.org/structure/4OR7, accessed on 17 February 2024.). The 3D structure of **4P** was constructed using Chem3D 20.0 software (Chemical Structure Drawing Standard; Cambridge Soft corporation, Cambridge, MA, USA (2019)). Hydrogen atoms were added to the DHFR structure, and water molecules and bound ligands were manually removed. During the docking simulation, the 3D conformer of **4P** was placed within the binding pocket of DHFR. Types of interactions between the docked protein and **4P** were analyzed after the end of molecular docking and were sorted by—CDOCKER ENERGY.

#### 3.3.7. Inhibition of DHFR Activities In Vitro

The inhibition of DHFR activity was measured using enzyme-linked immunosorbent assay (ELISA) kits (mlbio, Shanghai, China) under different concentrations (2, 4, 8, 16 and 32 μg/mL) of **4P**, according to the manufacturer′s instructions. The procedure is as follows: The diluted standard solution and prepared samples should be added to the ELISA plate in accordance with the protocol set out in the instruction manual. This should be followed for the incubation, washing, coloration and measurement of the absorbance (OD value) at 450nm within the specified time frame. Calculate the DHFR activity based on the OD value readings.

### 3.4. Hemolysis Assay

The hemolysis assay was carried out according to the procedure of the reported method with some modifications [43]. Sterile sheep red blood cells were centrifuged and resuspended in a saline solution. The test compounds were added in a range from 0.25 to 64 µg/mL and allowed to incubate at 37 °C for 1 h. The mixture was centrifuged, and the supernatant was collected. The optical density of the supernatant was detected at 570 nm. Triton-X100 (0.1%) was used as a positive control, and untreated cells were used as a negative control. The hemolysis rate was calculated according to the following formula:

Hemolysis rate (%) = (OD_Treatment_ − OD_Negative_)/(OD_Positive_ − OD_Negative_) × 100%

### 3.5. In Vivo Infection Model

The mouse model of *K. pneumoniae*-induced pneumonia was used to assess the in vivo antibacterial effects of **4P**. Eight-week-old female BALB/c mice weighing 20 ± 2 g were purchased from Beijing SiPeiFu Biotechnology Co., Ltd., Beijing, China. The animal experiments were approved by the Ethics Committee of Research Involving Animals of the Lanzhou Institute of Husbandry and Pharmaceutical Science of CAAS and performed in accordance with the guidelines of the Ethics Committee for Animal Experiments. Prior to the formal experiment, all mice were acclimated for 5 days. The mice were kept in a pathogen-free environment with a 12 h light/12 h dark cycle, 50% ± 10% humidity, and a temperature of 24 °C ± 2 °C. Following growth in MH broth to an OD_600 nm_ of 0.4, the mice were semi-anesthetized by intraperitoneal injection with sodium Ulatan (TCI, 750 mg/kg). Then, 50 µL of *K. pneumoniae* suspension (approximately 7 × 10^7^ CFU) was delivered by nasal instillation. The mice in the model group exhibited mortality rates of approximately 50% on the first day following the bacterial attack and 100% mortality within two days, indicating that the model was successful. The mice were divided into four groups (10 mice per group), three of which received the control solvent, **4P**, and colistin which were delivered via intraperitoneal injection at 1 h after infection, respectively. An additional group was injected with saline as a blank control group. Drug and solvent injections were performed once every day for 3 days. The dosages of **4P** and colistin were 4 and 1 mg/kg/d, respectively. After the death or sacrifice of the mice, various tissues were immediately harvested, and subjected to the detection of the bacterial load or a histopathology assay, respectively.

### 3.6. Statistical Analysis

The results of all experiments were presented as the mean ± SD. The statistical significance of the data was analyzed by GraphPad Prism 8.0 software (GraphPad Software, San Diego, CA, USA) with an unpaired Student’s t-test or a non-parametric one-way ANOVA. Significant values are represented by an asterisk: * p < 0.05, ** p < 0.01, *** p < 0.001, **** p < 0.0001.

## 4. Conclusions

A total of 37 novel aminoguanidine indole derivatives were designed, synthesized and identified. The synthesized compounds showed similar activity against both Gram-positive and Gram-negative bacteria. Compounds **3I-3U**, **4O**, **4P**, and **5P** demonstrated strong antibacterial activity against the tested strains, including multidrug-resistant ones, with MIC values ranging from 2 to 16 μg/mL. Compound **4P**, with the most antibacterial activity, had a lower risk of inducing resistance in *K. pneumoniae* and a higher in vivo protection rate than colistin. The main mechanisms of **4P** might be the destruction of the inner membrane integrity and the inhibition of DHFR. Furthermore, the hemolytic effect of **4P** was insignificant on sheep red blood cells. Therefore, compound **4P**, as an aminoguanidine derivative with an indole ring, will be a promising candidate for a novel broad-spectrum antibacterial agent.

## Figures and Tables

**Table 1:** Chemical structures of target aminoguanidine indole derivatives.

Structure	Compound	R1	X	Y	MW
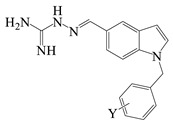	**3A**	H	C	2-F	309.35
	**3B**	H	C	3-F	309.35
	**3C**	H	C	4-F	309.35
	**3D**	H	C	2-F, 4-F	327.34
	**3E**	H	C	2-F,5-F	327.34
	**3F**	H	C	2-F, 6-F	327.34
	**3G**	H	C	3-F, 4-F	327.34
	**3H**	H	C	3-F, 5-F	327.34
	**3I**	H	C	3-CF_3_	359.36
	**3J**	H	C	4-CF_3_	359.36
	**3K**	H	C	3-CF_3_, 5-CF_3_	427.35
	**3L**	H	C	2-Cl	325.80
	**3M**	H	C	3-Cl	325.80
	**3N**	H	C	4-Cl	325.80
	**3O**	H	C	2-Cl, 4-Cl	360.24
	**3P**	H	C	3-Cl, 4-Cl	360.24
	**3Q**	H	C	2-Br	370.25
	**3R**	H	C	3-Br	370.25
	**3S**	H	C	4-Br	370.25
	**3T**	H	C	2-Cl, 4-F	343.79
	**3U**	H	C	3-Cl, 4-F	343.79
	**3V**	H	C	4-CN	316.37
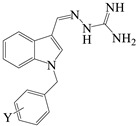	**4L**	H	C	2-Cl	325.80
	**4M**	H	C	3-Cl	325.80
	**4N**	H	C	4-Cl	325.80
	**4O**	H	C	2-Cl, 4-Cl	360.24
	**4P**	H	C	3-Cl, 4-Cl	360.24
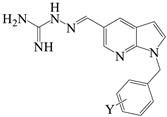	**5L**	H	N	2-Cl	326.79
	**5M**	H	N	3-Cl	326.79
	**5N**	H	N	4-Cl	326.79
	**5O**	H	N	2-Cl, 4-Cl	361.23
	**5P**	H	N	3-Cl, 4-Cl	361.23
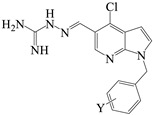	**6L**	Cl	N	2-Cl	361.23
	**6M**	Cl	N	3-Cl	361.23
	**6N**	Cl	N	4-Cl	361.23
	**6O**	Cl	N	2-Cl, 4-Cl	395.67
	**6P**	Cl	N	3-Cl, 4-Cl	395.67

**Table 2:** The MICs and MBCs of target compounds against ESKAPE and MRSA strains.

Compounds	Strains (MIC/MBC, µg/mL)						
	*E. faecium* ATCC35667	*S. aureus* ATCC29223	MRSA	*K. pneumoniae* ATCC700603	*A. baumannii* ATCC19606	*P. aeruginosa* ATCC27853	*E. coli* ATCC25922
**3A**	8/16	8/32	16/16	32/64	8/64	16/16	32/64
**3B**	8/16	8/32	16/-	32/32	8/64	16/32	32/32
**3C**	8/16	4/16	16/32	32/64	16/16	16/32	16/32
**3D**	8/16	8/32	16/16	32/32	8/16	16/64	32/-
**3E**	8/16	8/16	16/16	32/64	8/64	32/64	32/32
**3F**	8/16	4/64	>64/-	>64/-	8/32	>64/-	64/-
**3G**	8/16	8/32	16/64	32/32	8/16	16/32	32/32
**3H**	8/16	4/16	16/16	32/32	8/16	16/16	16/32
**3I**	4/8	4/8	8/8	16/64	4/16	16/64	8/16
**3J**	4/16	2/32	8/8	8/8	4/32	16/16	8/16
**3K**	4/4	2/8	8/8	8/16	4/4	32/64	8/32
**3L**	4/8	2/8	8/8	16/16	8/8	16/32	16/64
**3M**	4/8	4/8	16/32	16/64	4/8	16/16	16/16
**3N**	4/8	4/8	8/8	8/64	4/8	16/32	16/16
**3O**	4/8	4/8	4/8	8/32	4/8	16/64	4/32
**3P**	4/4	2/8	4/4	8/32	2/4	16/32	4/16
**3Q**	4/16	2/8	8/64	8/32	4/16	32/64	8/16
**3R**	4/8	2/32	8/8	16/16	4/4	16/32	8/16
**3S**	4/32	8/32	16/32	16/64	4/16	32/-	16/32
**3T**	8/16	4/8	8/32	8/64	4/8	16/32	16/16
**3U**	4/8	4/8	8/64	16/32	4/4	16/16	8/8
**3V**	32/32	8/32	64/64	>64/-	32/32	64/64	64/64
**4L**	8/16	2/8	2/8	16/32	8/8	16/16	8/16
**4M**	8/16	2/8	2/8	16/32	8/16	16/16	8/16
**4N**	4/16	2/4	2/8	16/16	8/16	8/16	8/8
**4O**	2/16	2/8	2/4	4/16	4/8	8/16	4/8
**4P**	2/8	2/4	2/4	4/16	4/32	8/16	2/4
**5L**	32/32	8/32	8/32	32/32	32/32	16/16	16/16
**5M**	>64/-	16/64	16/32	>64/-	>64/-	>64/-	>64/-
**5N**	16/32	4/32	8/16	16/32	16/32	16/32	16/16
**5O**	16/16	2/8	4/32	16/64	16/16	16/32	8/16
**5P**	16/16	4/8	4/32	8/32	16/16	16/32	8/8
**6L**	>64/-	16/64	16/64	>64/-	>64/-	>64/-	>64/-
**6M**	8/64	8/16	4/32	>64/-	8/8	>64/-	16/16
**6N**	32/-	8/32	8/16	>64/-	16/32	>64/-	16/64
**6O**	>64/-	>64/-	>64/-	>64/-	>64/-	>64/-	>64/-
**6P**	16/32	8/64	8/64	>64/-	32/32	>64/-	>64/-
Colistin	>64/-	>64/-	64/-	2/8	0.5/0.5	1/2	1/2

-: not detected.

**Table 3:** The MICs and MBCs of active compounds against clinical *K. pneumoniae* isolates.

Compounds	Strains (MIC/MBC, µg/mL)										
	*K.P.* 2102	*K.P.* 2105	*K.P.* 2107	*K.P.* 2108	*K.P.* 2109	*K.P.* 2112	*K.P.* 2118	*K.P.* 2125	*K.P.* 2134	*K.P.* 2135	*K.P.* 2138
**3I**	8/8	8/32	8/16	8/16	8/8	8/64	8/8	8/16	8/32	8/32	8/8
**3J**	8/32	8/16	8/8	8/8	8/16	8/8	8/8	8/8	8/8	8/8	8/8
**3K**	8/64	16/64	8/16	8/32	16/32	8/32	8/16	8/16	16/32	8/16	8/32
**3L**	8/16	16/16	8/8	8/8	8/16	8/8	8/8	8/16	8/8	8/8	8/8
**3M**	8/16	16/16	8/16	8/8	8/8	8/8	8/64	16/16	8/16	8/32	8/8
**3N**	8/8	16/32	8/16	8/8	8/16	8/32	8/64	16/16	8/16	8/8	8/8
**3O**	8/8	8/32	4/16	8/16	8/64	4/64	4/4	8/32	8/64	4/8	4/16
**3P**	4/16	8/16	4/8	4/16	4/64	4/8	4/4	8/16	8/8	4/8	4/8
**3Q**	8/8	8/-	8/8	8/8	8/8	8/8	8/32	8/8	8/8	8/16	8/16
**3R**	8/8	16/32	8/8	8/8	8/8	8/8	8/64	16/16	8/8	8/32	8/8
**3S**	8/16	16/32	8/16	8/64	8/16	8/16	8/8	8/16	8/8	8/8	8/16
**3T**	8/8	16/16	8/8	8/8	8/8	8/8	8/8	8/8	8/8	8/8	8/16
**3U**	8/8	8/8	8//32	8/8	816	8/8	8/8	8/8	8/8	8/8	8/8
**4O**	4/8	4/8	4/8	4/8	8/8	4/4	4/8	4/4	8/8	4/16	4/16
**4P**	4/8	4/8	4/4	4/8	4/16	4/4	4/8	4/8	4/8	4/4	4/4
**5P**	8/16	8/8	16/16	8/32	8/32	8/8	8/8	8/8	8/8	8/18	8/16
Colistin	2/4	>64/-	8/8	>64/-	>64/-	4/4	8/8	8/16	8/32	0.5/4	4/8

-: not detected.

**Table 4:** The hemolysis values of **3P**, **4P** and **5P**.

	Compounds (µg/mL)		
	3P	4P	5P
HC_50_	245.0	123.6	243.7
SI *	30.63	30.90	30.46

* Selectivity index (SI), SI = HC_50_/MIC_ATCC 700603_.

## Data Availability

The raw data supporting the conclusion of this study will be made available by the authors.

## Supplementary Materials

The following supporting information can be downloaded at: https://www.mdpi.com/article/10.3390/molecules30040887/s1, Supplementary Materials S1 and S2: HRMS, ^1^H NMR, ^13^C NMR and ^19^F NMR spectrum of the compounds; HPLC profiles of all compounds.

## Abbreviations

*K. pneumoniae*	*Klebsiella pneumoniae*
DMF	*N,N*-Dimethylformamide
MDR	Multidrug-resistant
*MCR-1*	Mobile colistin resistance gene
MIC	Minimum inhibitory concentrations
CLSI	Clinical and Laboratory Standards Institute
PBS	Phosphate-buffered saline
CLSM	Confocal laser scanning microscope
SEM	Scanning electron microscopy
PI	Propidium iodide
DiOC_3_(5)	3,3′-dipropylthiadicarbocyanine iodides
