# Supplementary material for: Synthesis, Antimicrobial Activities, and Model of Action of Indolyl Derivatives Containing Amino-Guanidinium Moieties
Source: Molecules. 2025 Feb 14;30(4):887. doi: 10.3390/molecules30040887 (PMC11858076; doi:10.3390/molecules30040887)
Supplement: Supplementary file 1 [file molecules-30-00887-s001.zip › Supplementary Materials S1.pdf]

Supplementary Information

Synthesis, Antimicrobial Activities, and Model of Action of Indolyl Derivatives Containing Amino-Guanidinium Moieties

Yu-Xi Li 1,†, Xiang Geng 1,2,†, Qi Tao 1, Ruo-Chen Hao 1, Ya-Jun Yang 1, Xi-Wang Liu 1,* and Jian-Yong Li 1,*

1 Key Lab of New Animal Drug of Gansu Province, Key Lab of Veterinary Pharmaceutical Development of Ministry of Agriculture and Rural Affairs, Lanzhou Institute of Husbandry and Pharmaceutical Sciences of Chi-nese Academy of Agricultural Sciences, Lanzhou 730050, China
2 School of Health Nursing, Fuyang Vocational Technical College, Fuyang 236000, China
* Correspondence: xiwangliu@126.com (X.-W.L.); lijy1971@163.com (J.-Y.L.)
† The authors contributed equally to this work.

Supplemental Table of Contents

HRMS, ^{1}H NMR, ^{13}C NMR and ^{19}F NMR spectrum of the compounds

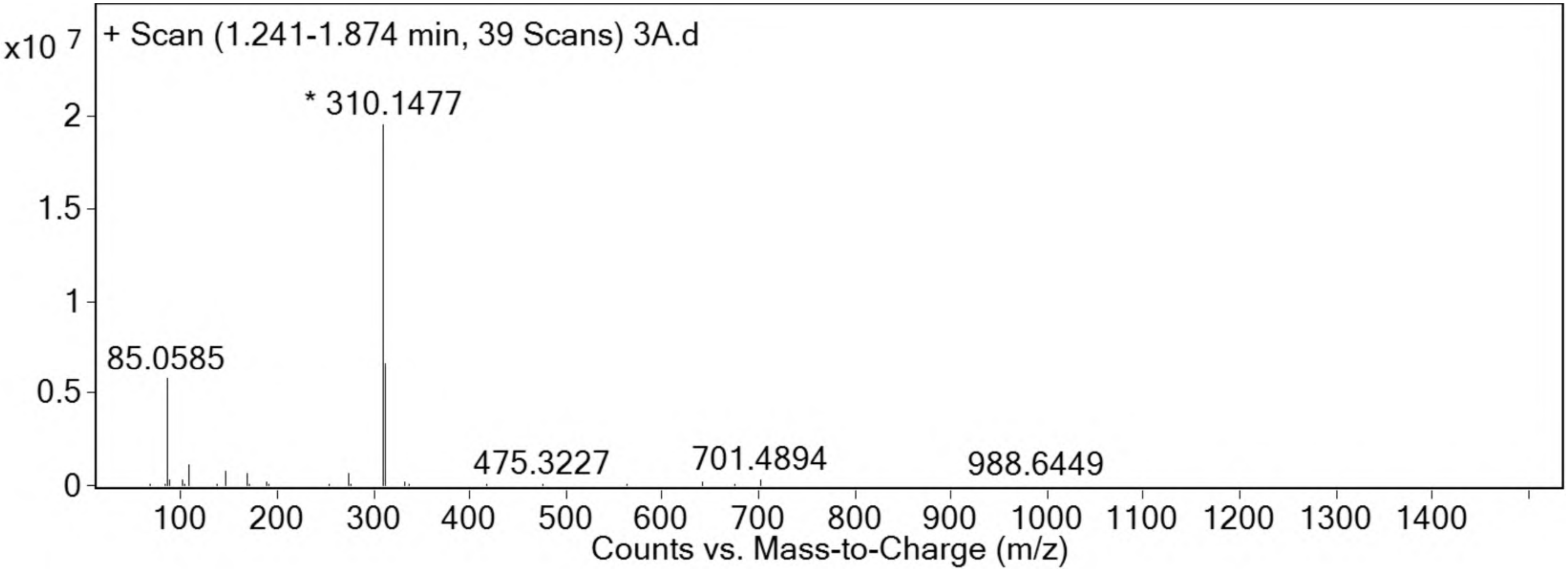
x10 7
+ Scan (1.241-1.874 min, 39 Scans) 3A.d
* 310.1477
85.0585
475.3227
701.4894
988.6449
2
1.5
1
0.5
0
100 200 300 400 500 600 700 800 900 1000 1100 1200 1300 1400
Counts vs. Mass-to-Charge (m/z)

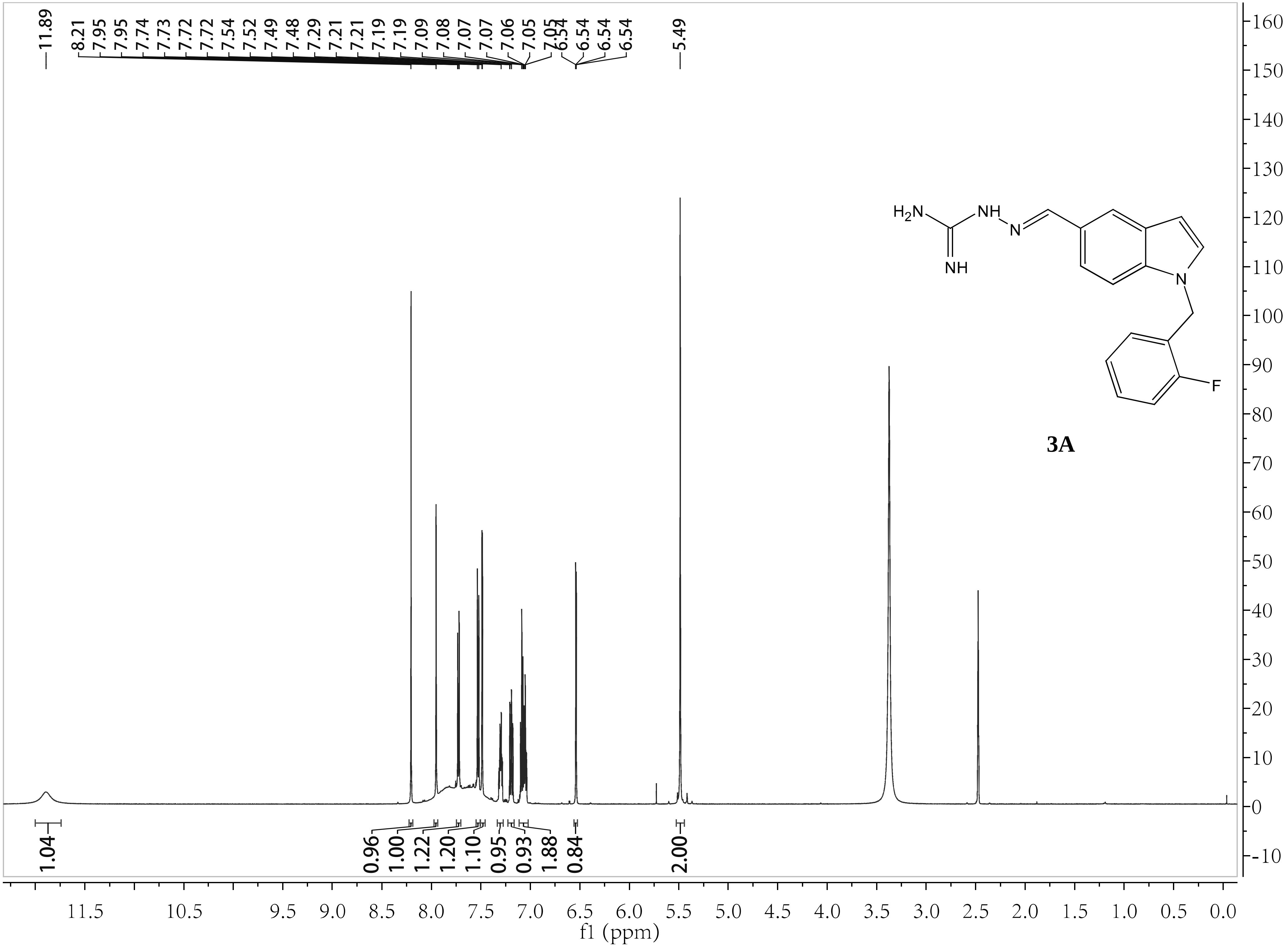

11.89
8.21
7.95
7.95
7.74
7.73
7.72
7.72
7.54
7.52
7.49
7.48
7.29
7.21
7.21
7.19
7.19
7.09
7.08
7.07
7.07
7.06
7.05
7.05
6.54
6.54
6.54
6.54
5.49
1.04
0.96
1.00
1.22
1.20
1.10
0.95
0.93
1.88
0.84
2.00
H2N
NH
N
NH
N
F
3A
f1 (ppm)

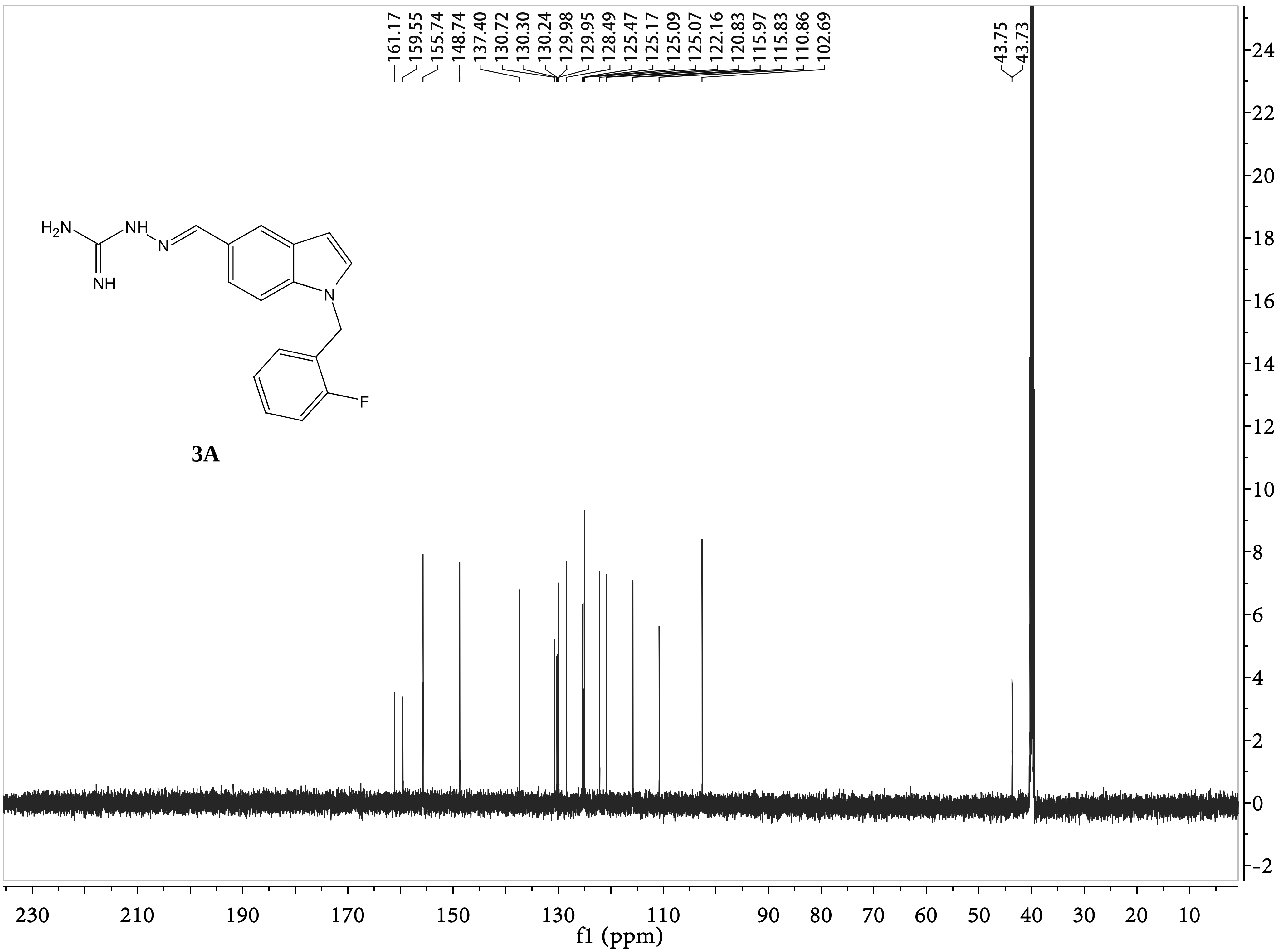

161.17
159.55
155.74
148.74
137.40
130.72
130.30
130.24
129.98
129.95
128.49
125.47
125.17
125.09
125.07
122.16
120.83
115.97
115.83
110.86
102.69
43.75
43.73
H2N
NH
N
NH
N
F
3A
24
22
20
18
16
14
12
10
8
6
4
2
0
-2
230
210
190
170
150
130
110
90
80
70
60
50
40
30
20
10
f1 (ppm)

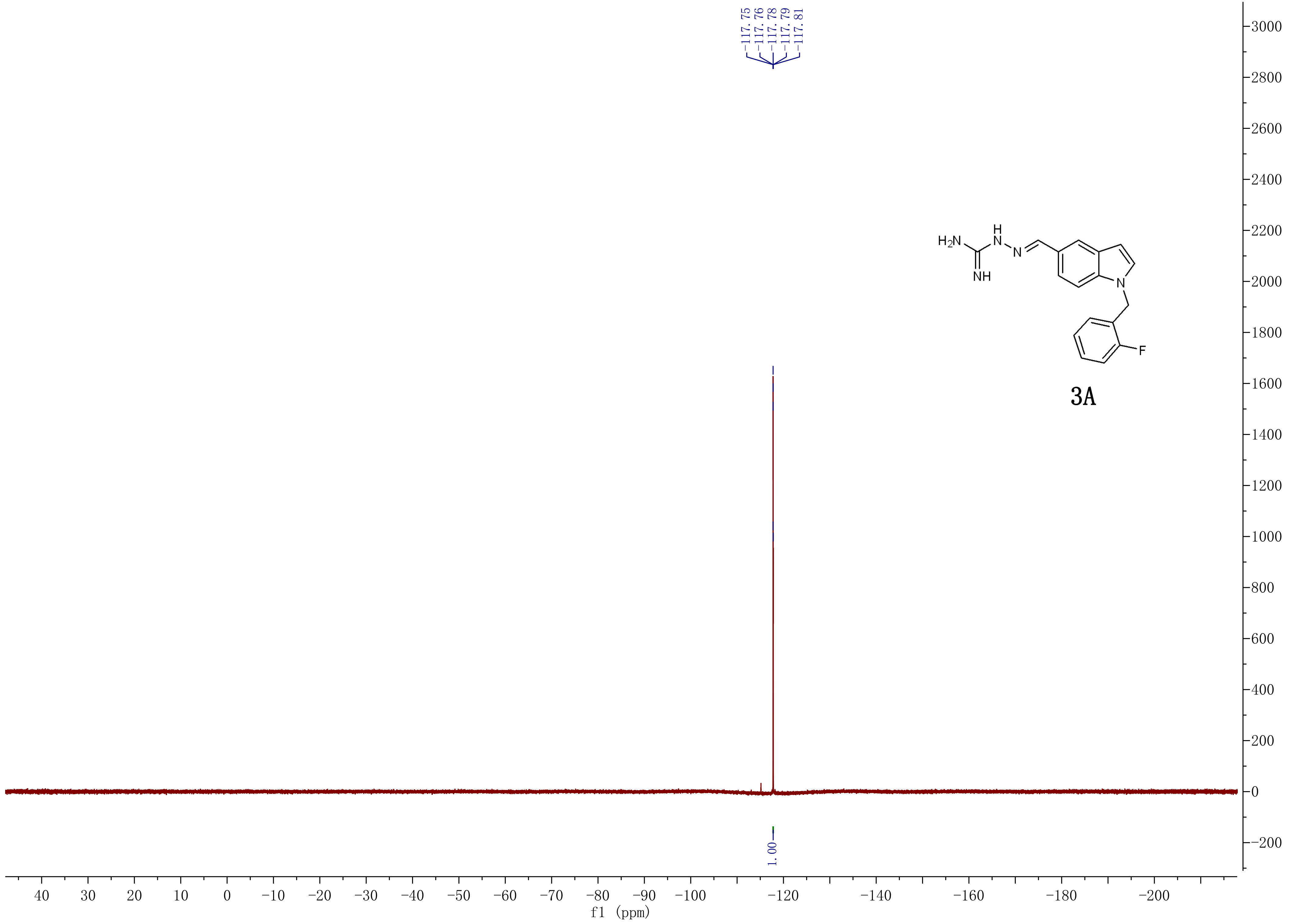

-117.75
-117.76
-117.78
-117.79
-117.81
H2N
H
N
N
NH
N
F
3A
1.00
40 30 20 10 0 −10 −20 −30 −40 −50 −60 −70 −80 −90 −100 −120 −140 −160 −180 −200
f1 (ppm)
3000 2800 2600 2400 2200 2000 1800 1600 1400 1200 1000 800 600 400 200 0 −200

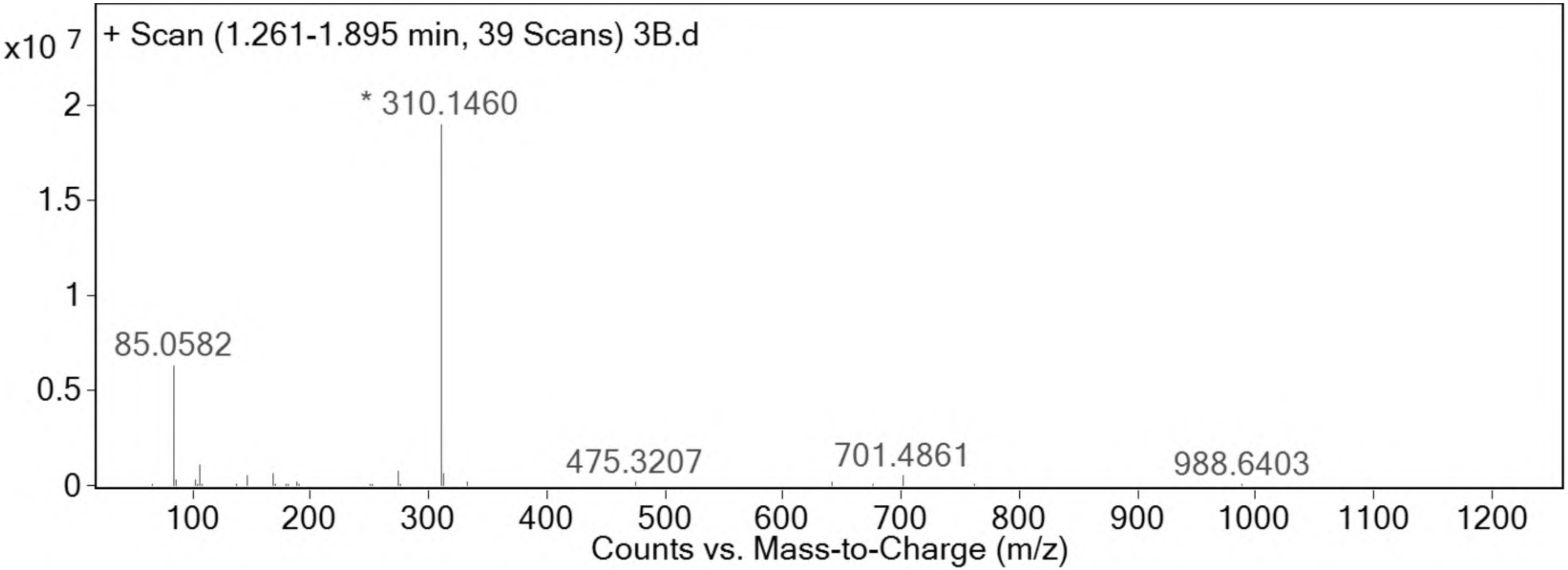
+ Scan (1.261-1.895 min, 39 Scans) 3B.d
x10 7
2
1.5
1
0.5
0
* 310.1460
85.0582
475.3207
701.4861
988.6403
100
200
300
400
500
600
700
800
900
1000
1100
1200
Counts vs. Mass-to-Charge (m/z)

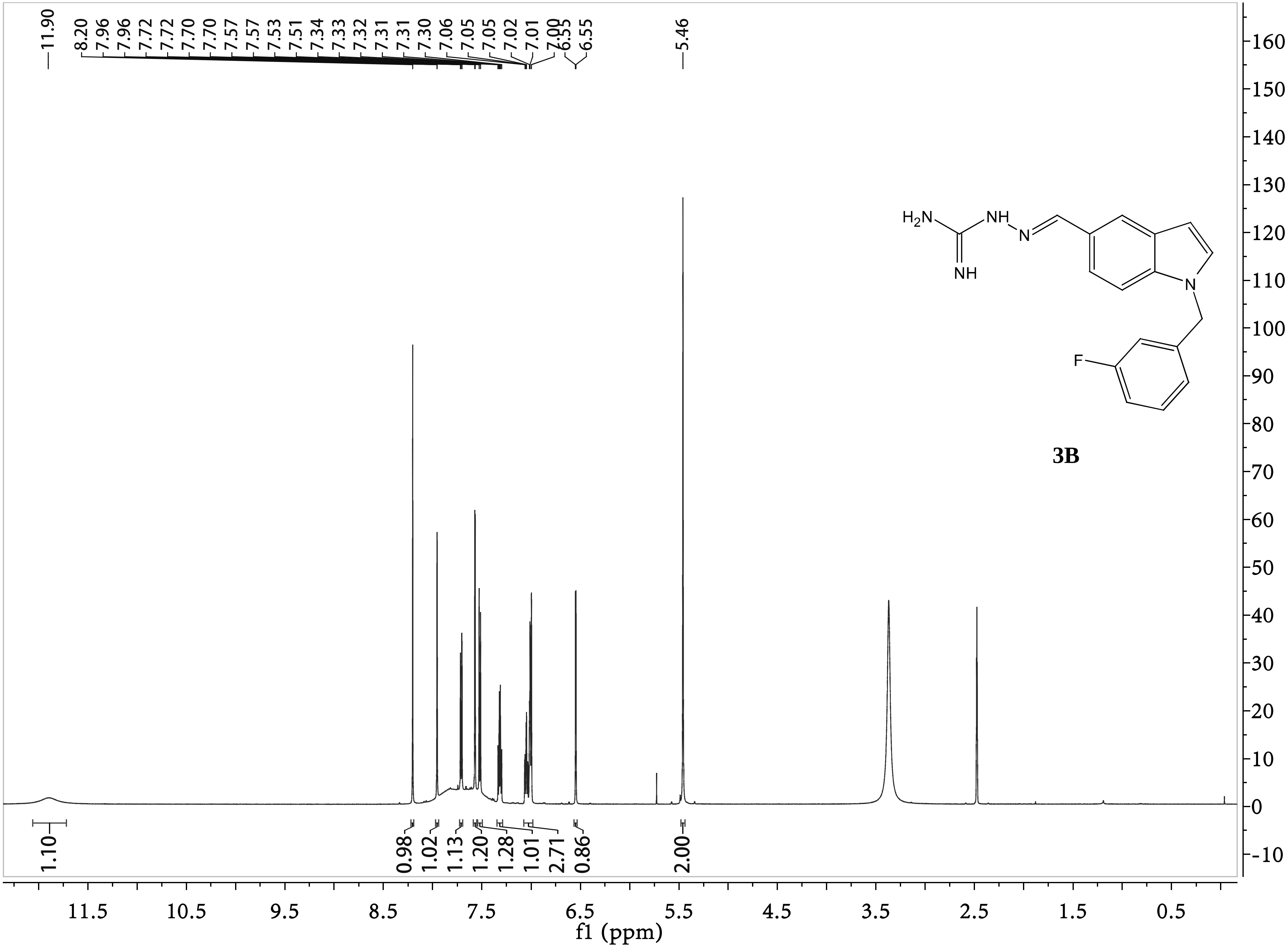
11.90
8.20
7.96
7.96
7.72
7.72
7.70
7.70
7.57
7.57
7.53
7.51
7.34
7.33
7.32
7.31
7.31
7.30
7.06
7.05
7.05
7.02
7.01
7.00
6.55
6.55
5.46
1.10
0.98
1.02
1.13
1.20
1.28
1.01
2.71
0.86
2.00
f1 (ppm)
H2N
NH
N
NH
N
F
3B

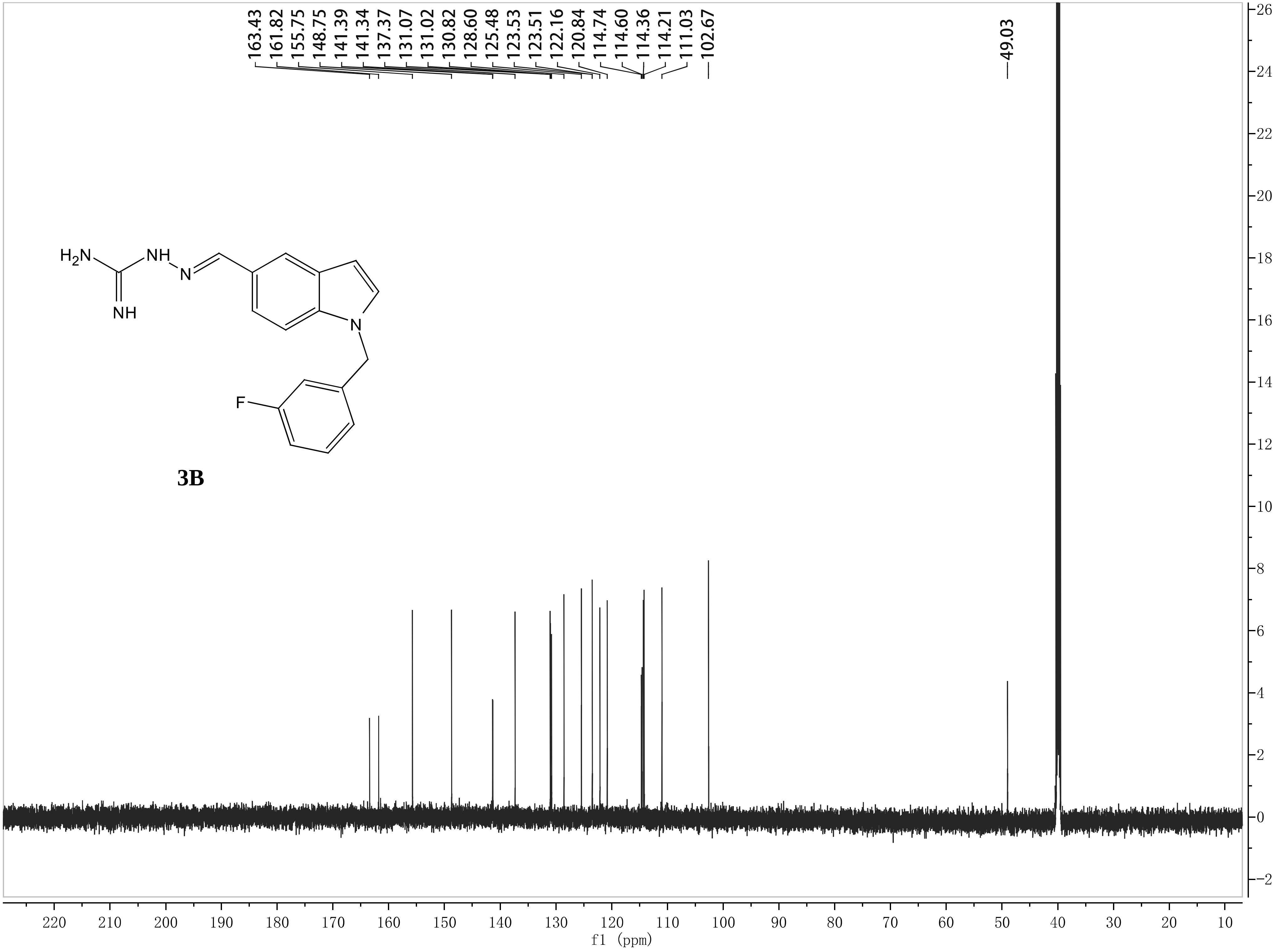
163.43
161.82
155.75
148.75
141.39
141.34
137.37
131.07
131.02
130.82
128.60
125.48
123.53
123.51
122.16
120.84
114.74
114.60
114.36
114.21
111.03
102.67
49.03
H_2N
NH
N
NH
N
F
3B
f1 (ppm)

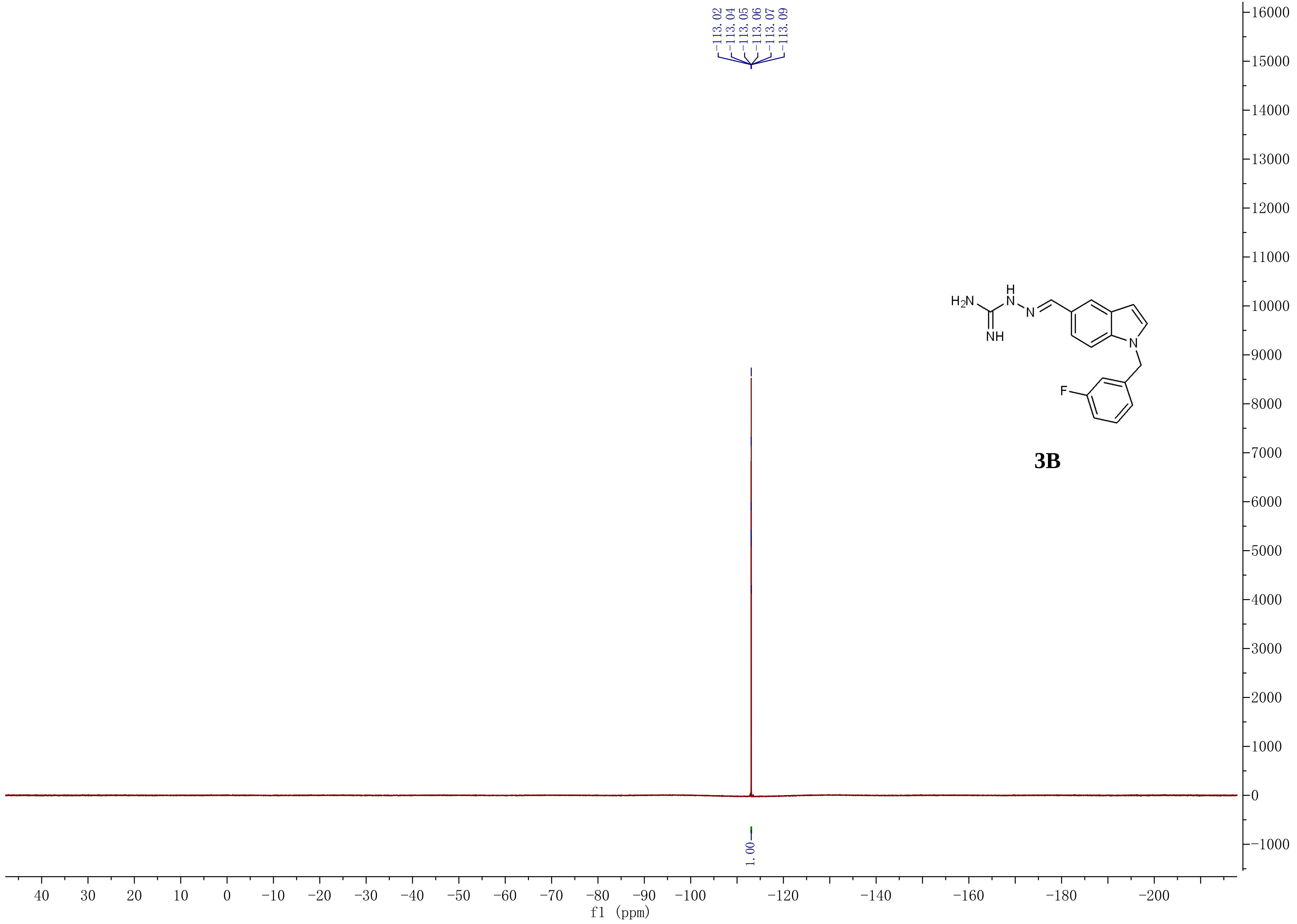

−113.02
−113.04
−113.05
−113.06
−113.07
−113.09
1.00
40 30 20 10 0 −10 −20 −30 −40 −50 −60 −70 −80 −90 −100 −120 −140 −160 −180 −200
f1 (ppm)
16000 15000 14000 13000 12000 11000 10000 9000 8000 7000 6000 5000 4000 3000 2000 1000 0 −1000
H2N
H
N
N
NH
N
F
3B

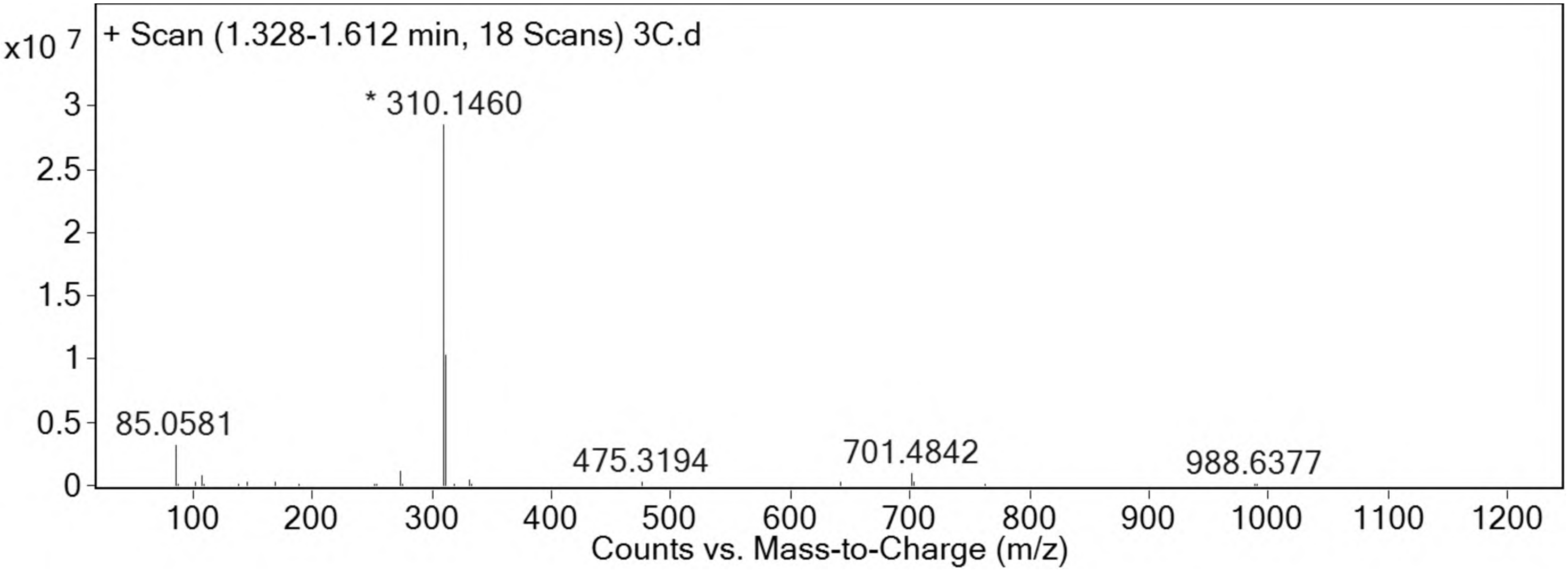
x10 7
+ Scan (1.328-1.612 min, 18 Scans) 3C.d
* 310.1460
85.0581
475.3194
701.4842
988.6377
3
2.5
2
1.5
1
0.5
0
100
200
300
400
500
600
700
800
900
1000
1100
1200
Counts vs. Mass-to-Charge (m/z)

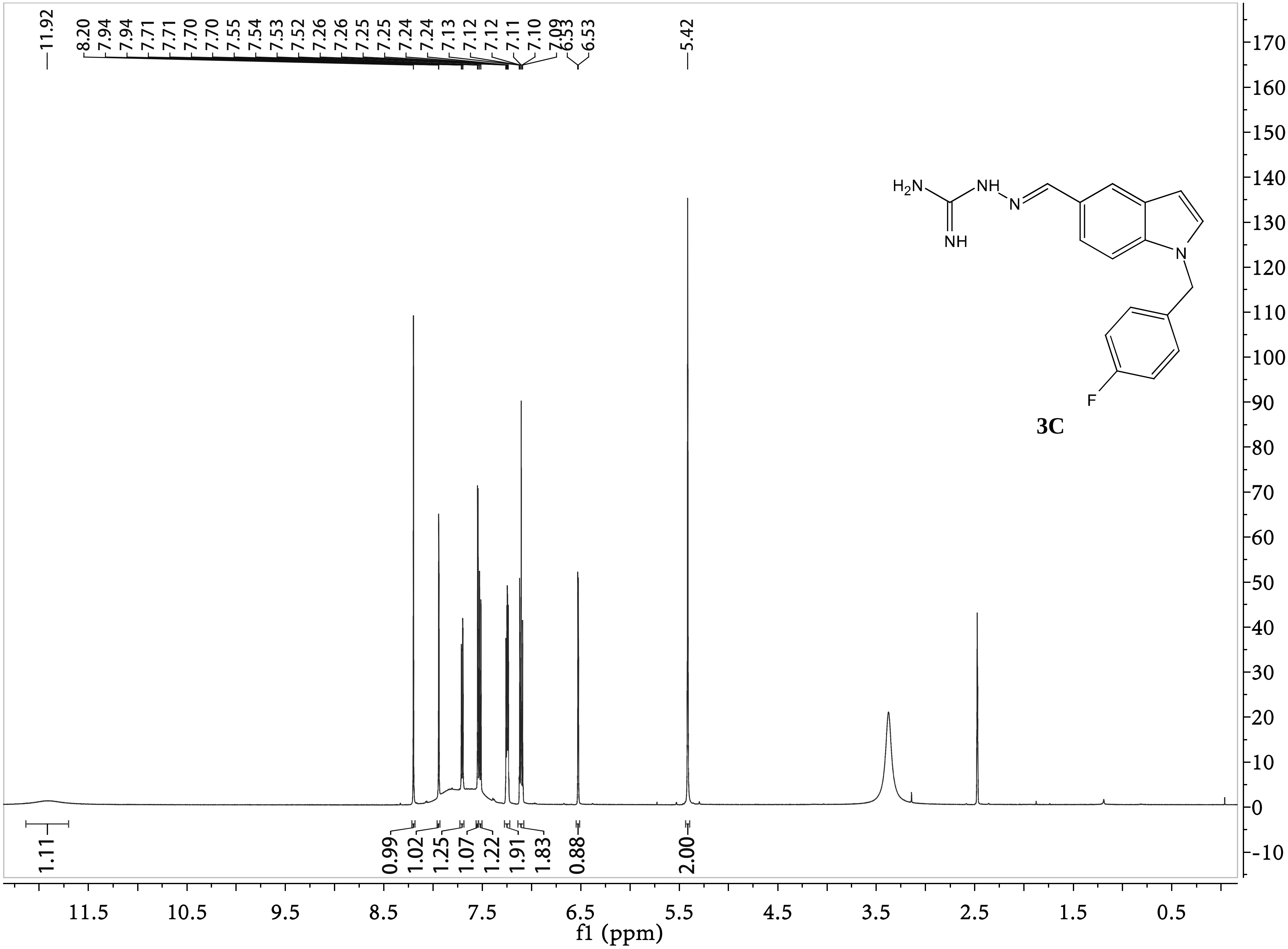

11.92
8.20
7.94
7.94
7.71
7.71
7.70
7.70
7.55
7.54
7.53
7.52
7.26
7.26
7.25
7.25
7.24
7.24
7.13
7.12
7.12
7.11
7.10
7.09
6.53
6.53
5.42
H_2N
NH
N
NH
N
F
1.11
0.99
1.02
1.25
1.07
1.22
1.91
1.83
0.88
2.00
11.5
10.5
9.5
8.5
7.5
6.5
5.5
4.5
3.5
2.5
1.5
0.5
f1 (ppm)
170
160
150
140
130
120
110
100
90
80
70
60
50
40
30
20
10
0
-10

3C

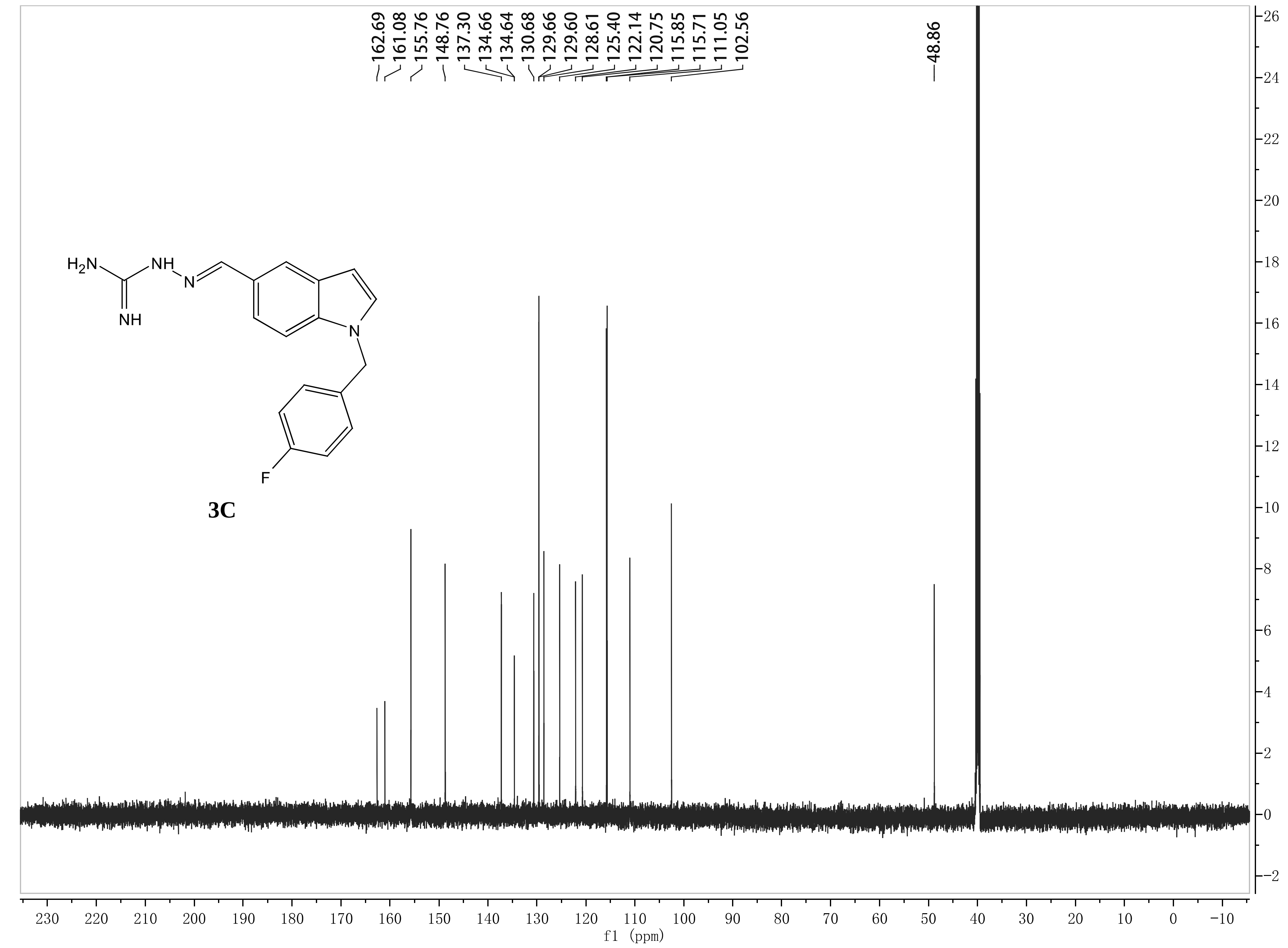

162.69
161.08
155.76
148.76
137.30
134.66
134.64
130.68
129.66
129.60
128.61
125.40
122.14
120.75
115.85
115.71
111.05
102.56
48.86
H2N
NH
N
NH
N
F
3C
f1 (ppm)

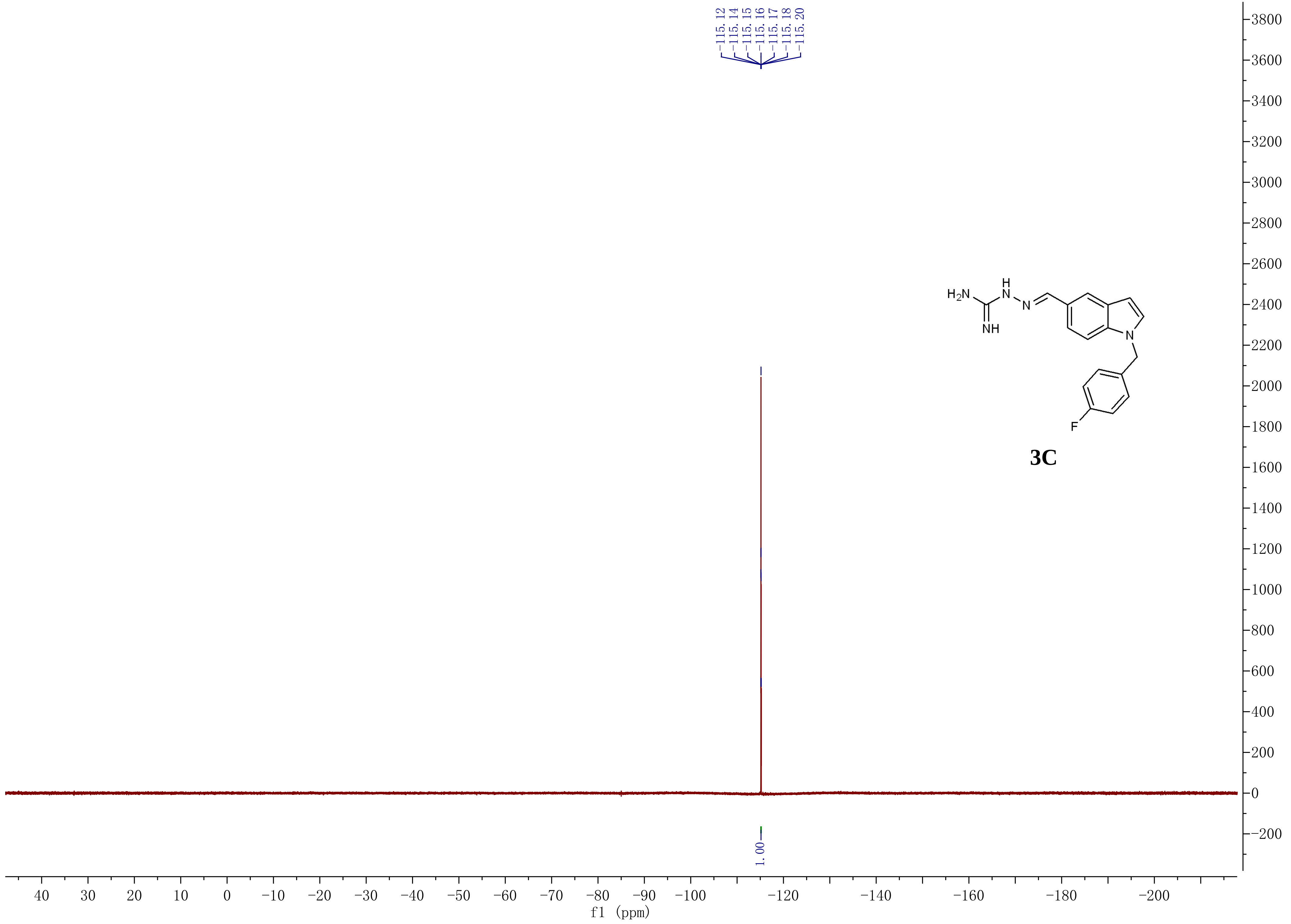

-115.12
-115.14
-115.15
-115.16
-115.17
-115.18
-115.20
H2N
H
N
N
NH
N
F
3C
1.00
f1 (ppm)

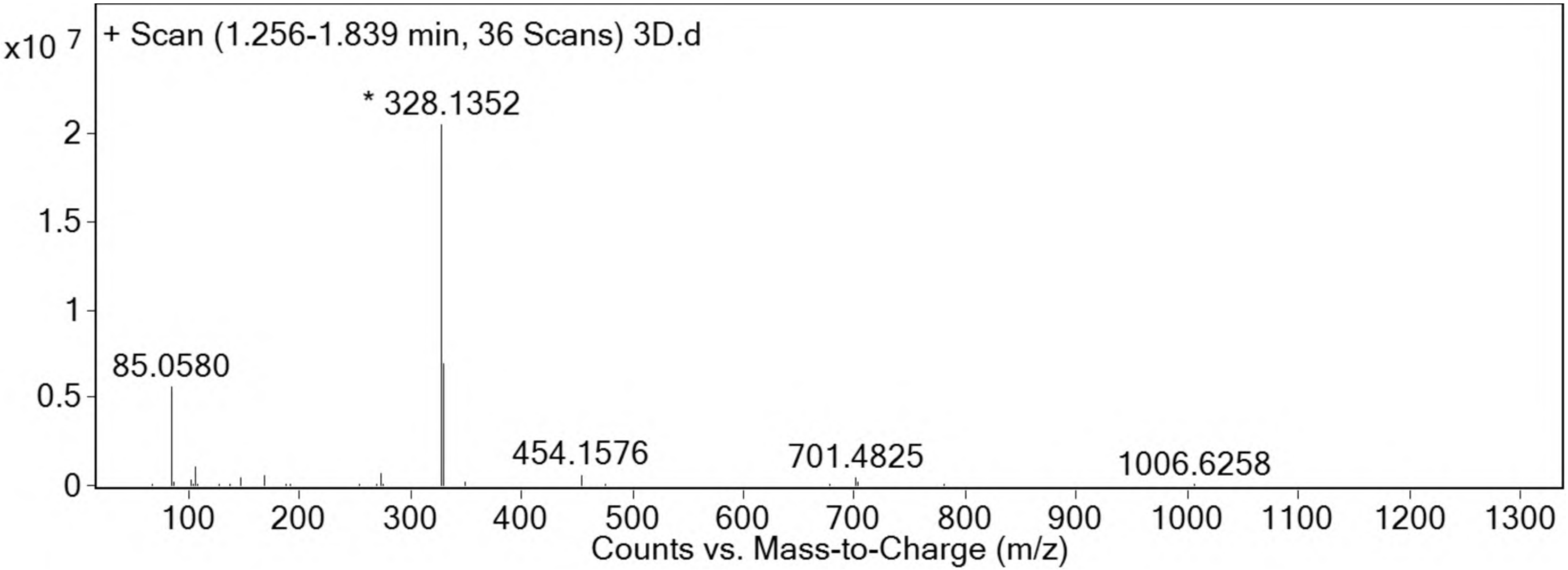

x10 7
+ Scan (1.256-1.839 min, 36 Scans) 3D.d
* 328.1352
85.0580
454.1576
701.4825
1006.6258
0
0.5
1
1.5
2
100
200
300
400
500
600
700
800
900
1000
1100
1200
1300
Counts vs. Mass-to-Charge (m/z)

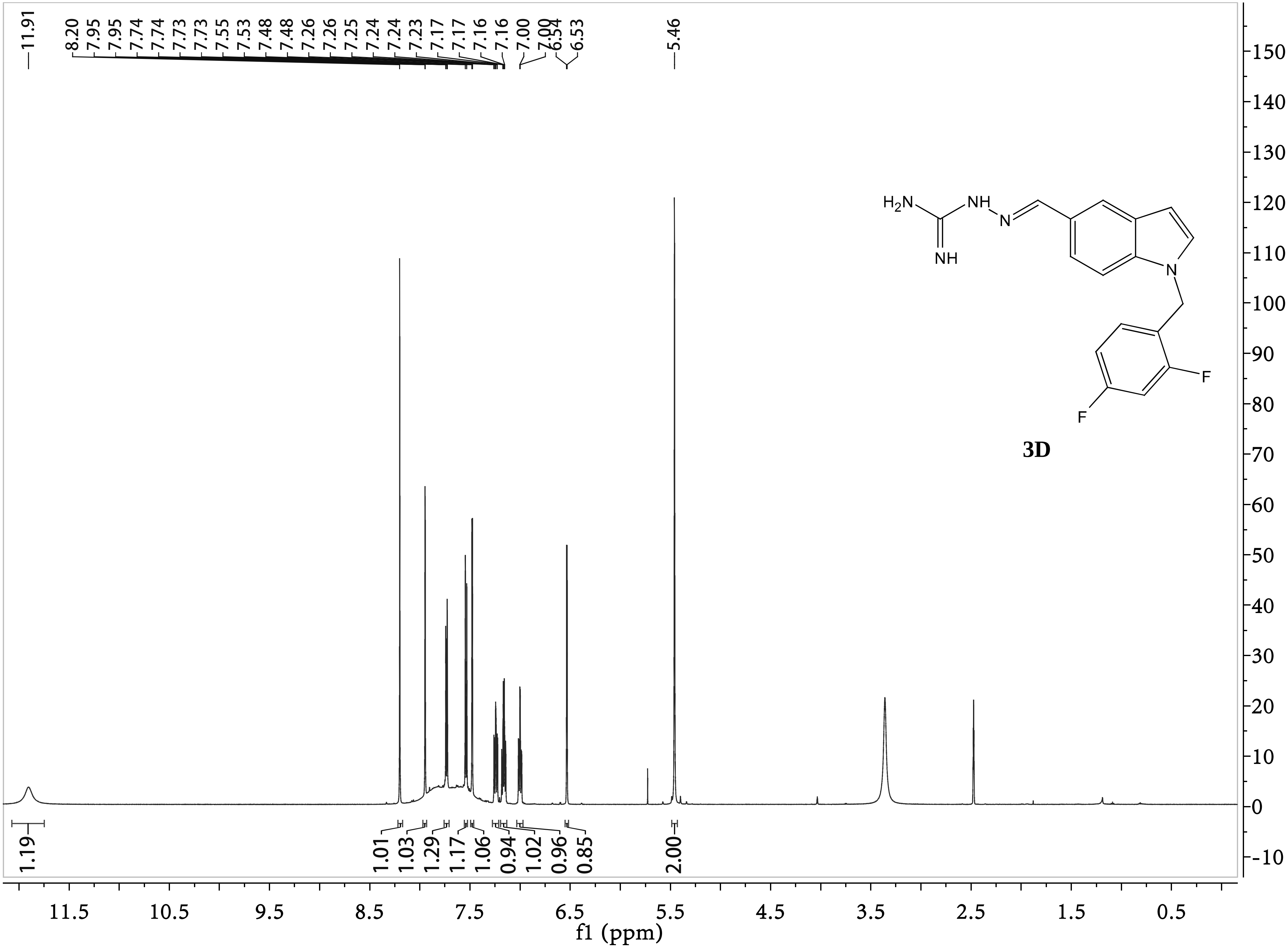

11.91
8.20
7.95
7.95
7.74
7.74
7.73
7.73
7.55
7.53
7.48
7.48
7.26
7.26
7.25
7.24
7.24
7.23
7.17
7.17
7.16
7.16
7.00
7.00
6.54
6.53
5.46
1.19
1.01
1.03
1.29
1.17
1.06
0.94
1.02
0.96
0.85
2.00
f1 (ppm)
H_2N
NH
N
NH
N
F
F
3D

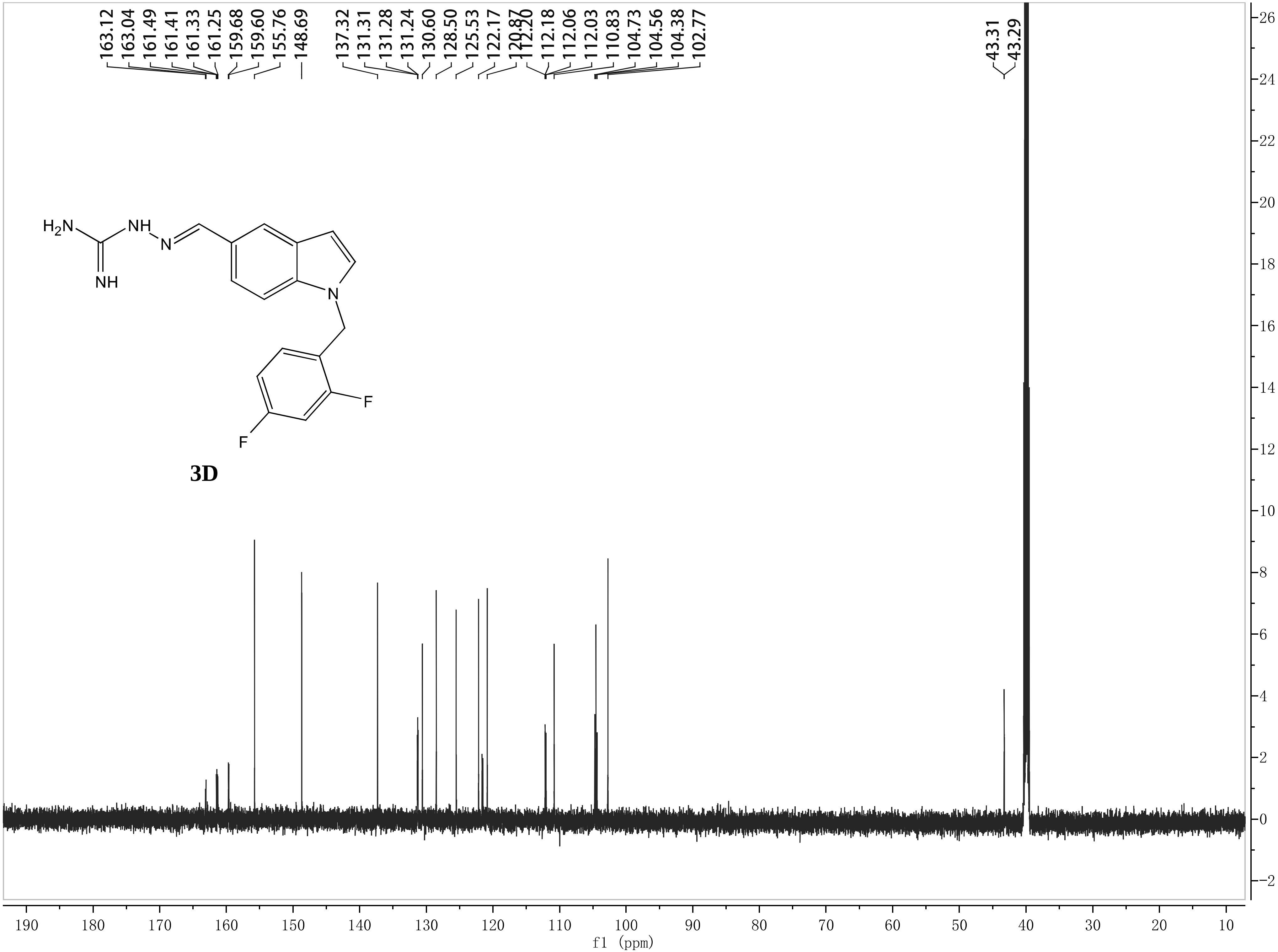
163.12
163.04
161.49
161.41
161.33
161.25
159.68
159.60
155.76
148.69
137.32
131.31
131.28
131.24
130.60
128.50
125.53
122.17
120.87
112.20
112.18
112.06
112.03
110.83
104.73
104.56
104.38
102.77
43.31
43.29
H2N
NH
N
NH
N
F
F
3D
f1 (ppm)

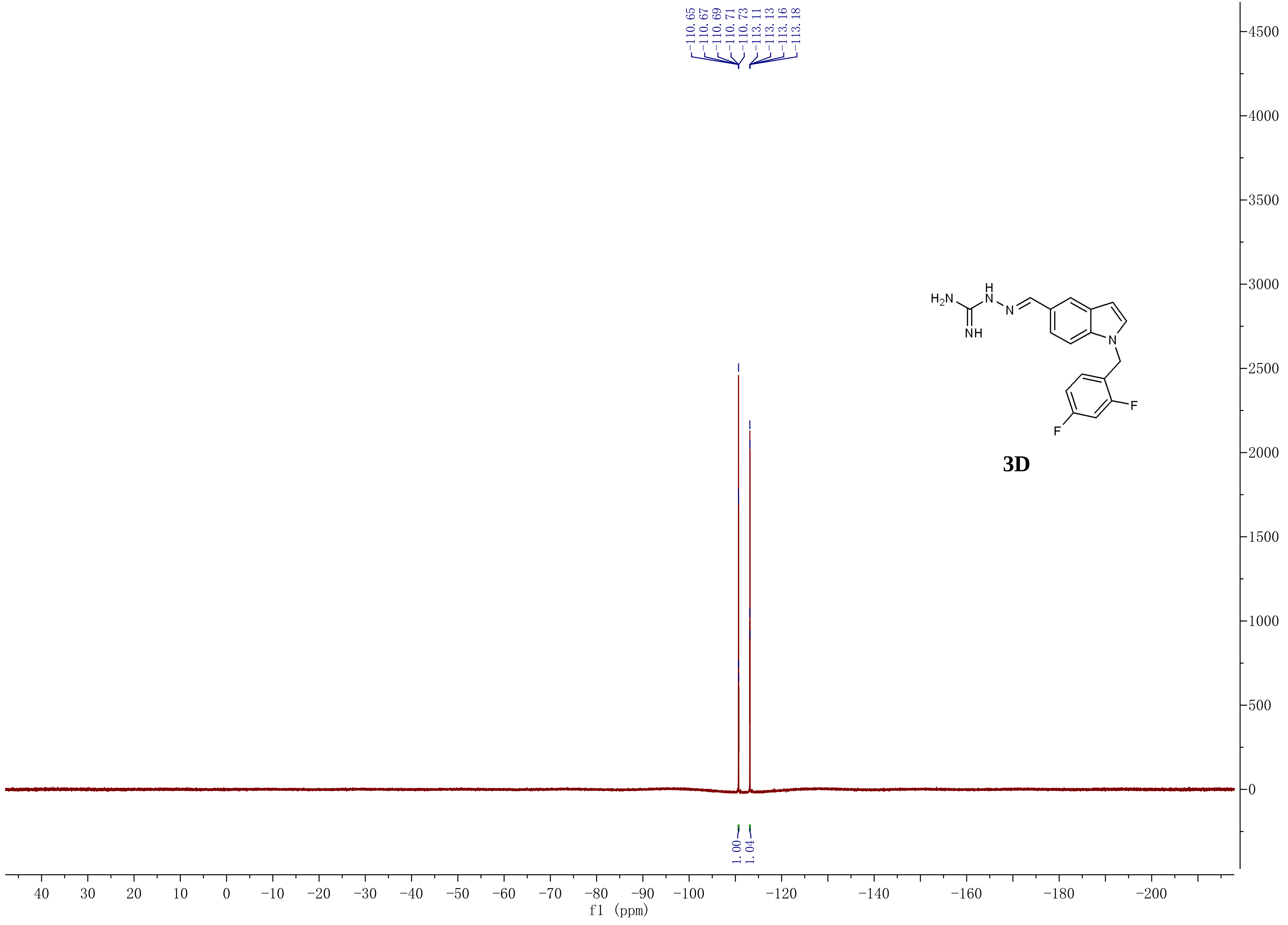

-110.65
-110.67
-110.69
-110.71
-110.73
-113.11
-113.13
-113.16
-113.18
H2N
H
N
N
NH
N
F
F
3D
1.00
1.04
4500
4000
3500
3000
2500
2000
1500
1000
500
0
40 30 20 10 0 −10 −20 −30 −40 −50 −60 −70 −80 −90 −100 −120 −140 −160 −180 −200
f1 (ppm)

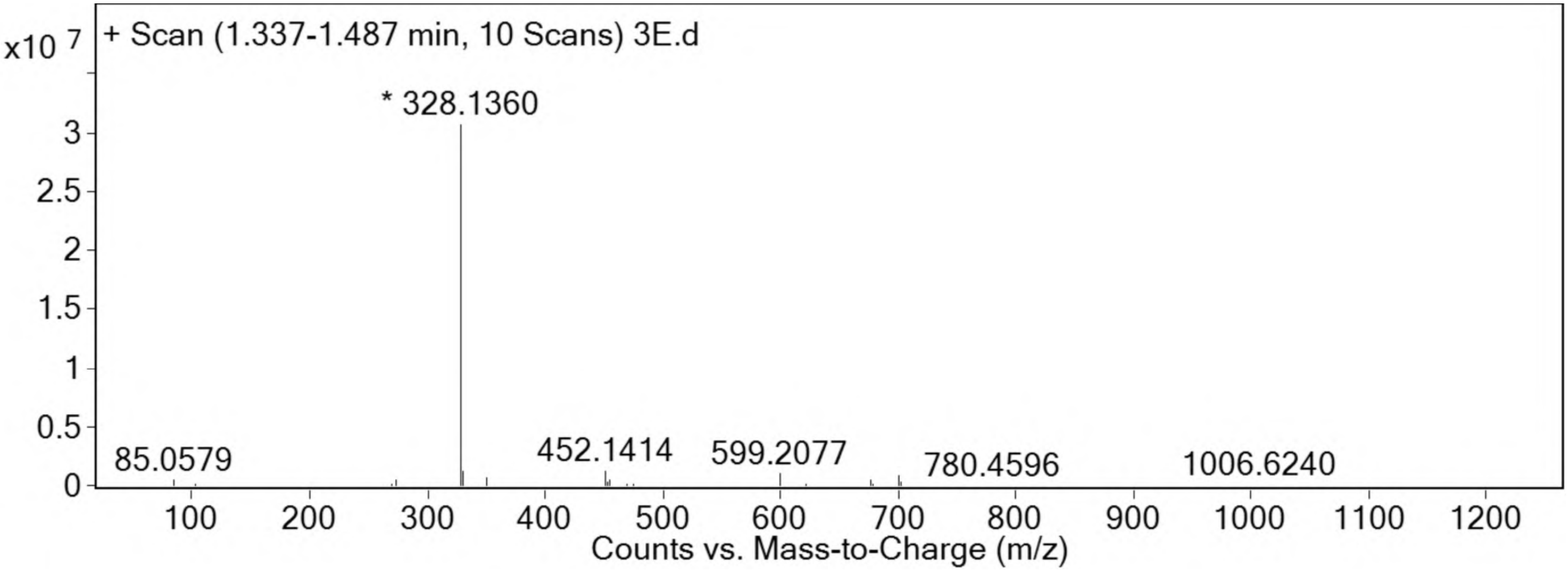

x10 7
+ Scan (1.337-1.487 min, 10 Scans) 3E.d
* 328.1360
85.0579
452.1414
599.2077
780.4596
1006.6240
3
2.5
2
1.5
1
0.5
0
100
200
300
400
500
600
700
800
900
1000
1100
1200
Counts vs. Mass-to-Charge (m/z)

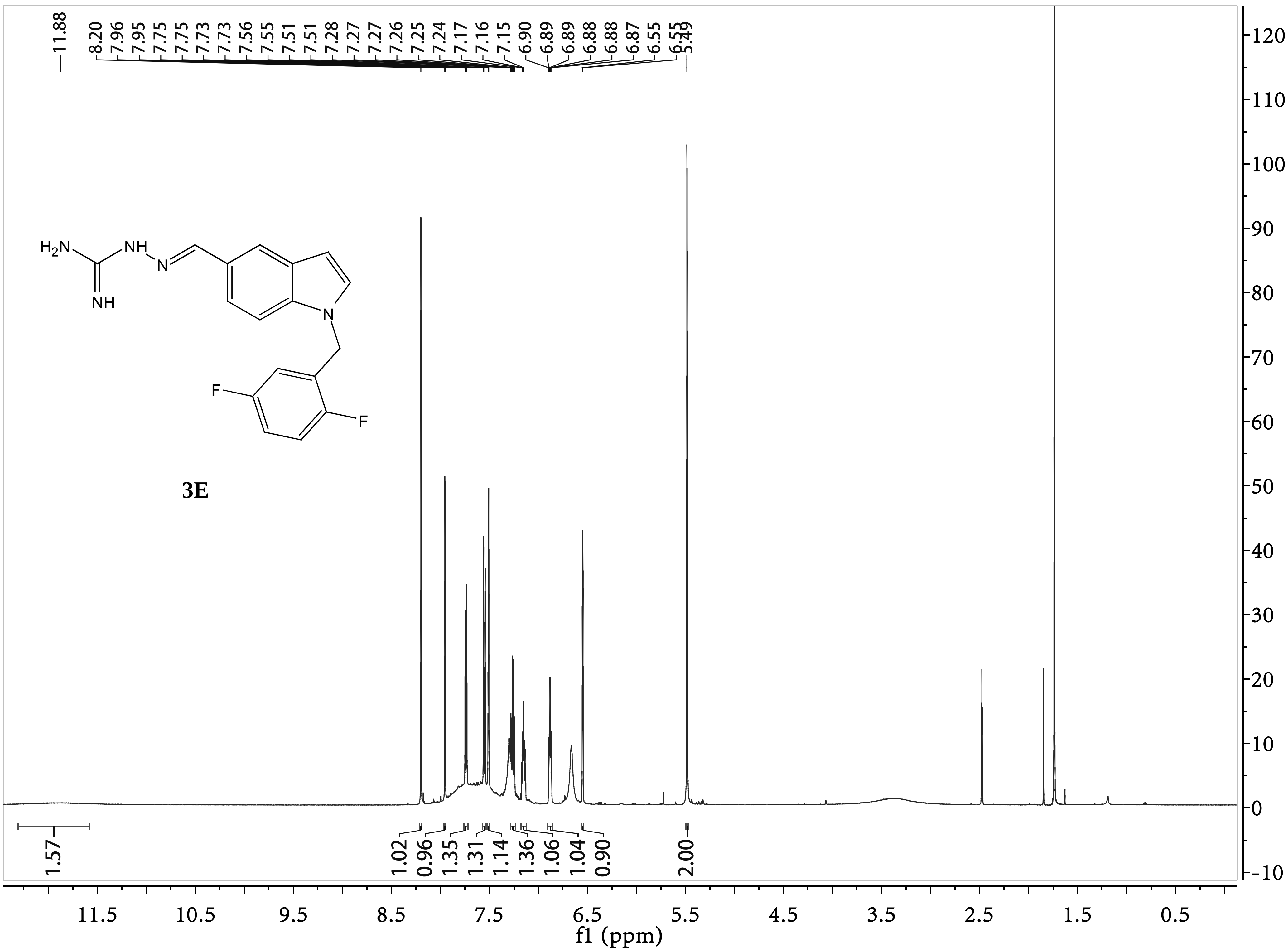
11.88
8.20 7.96 7.95 7.75 7.75 7.73 7.73 7.56 7.55 7.51 7.51 7.28 7.27 7.27 7.26 7.25 7.24 7.17 7.16 7.15 6.90 6.89 6.89 6.88 6.88 6.87 6.55 6.55 5.49
H2N
NH
N
NH
N
F
F
1.57
1.02 0.96 1.35 1.31 1.14 1.36 1.06 1.04 0.90
2.00
f1 (ppm)

3E

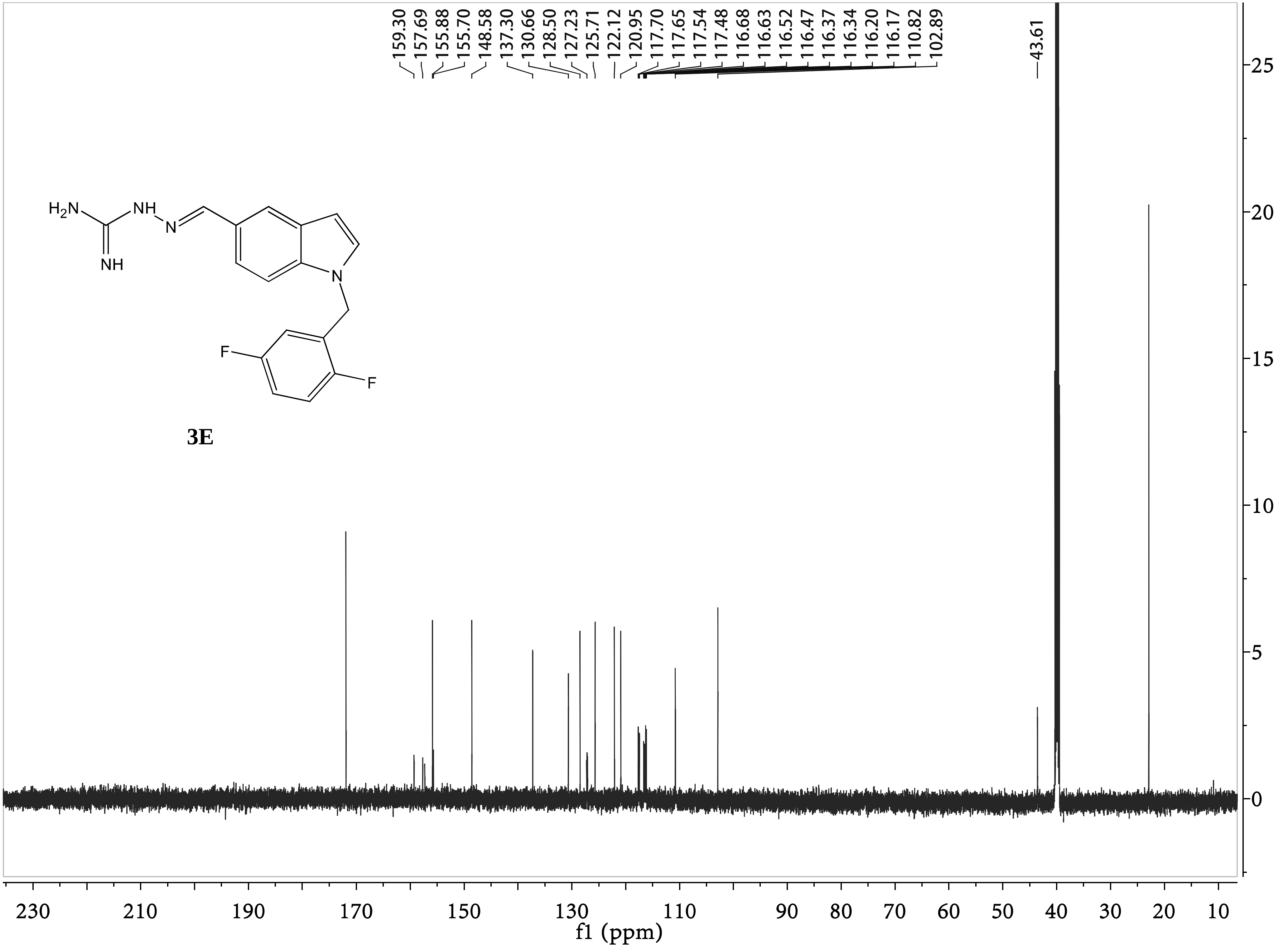

3E
159.30
157.69
155.88
155.70
148.58
137.30
130.66
128.50
127.23
125.71
122.12
120.95
117.70
117.65
117.54
117.48
116.68
116.63
116.52
116.47
116.37
116.34
116.20
116.17
110.82
102.89
43.61
f1 (ppm)

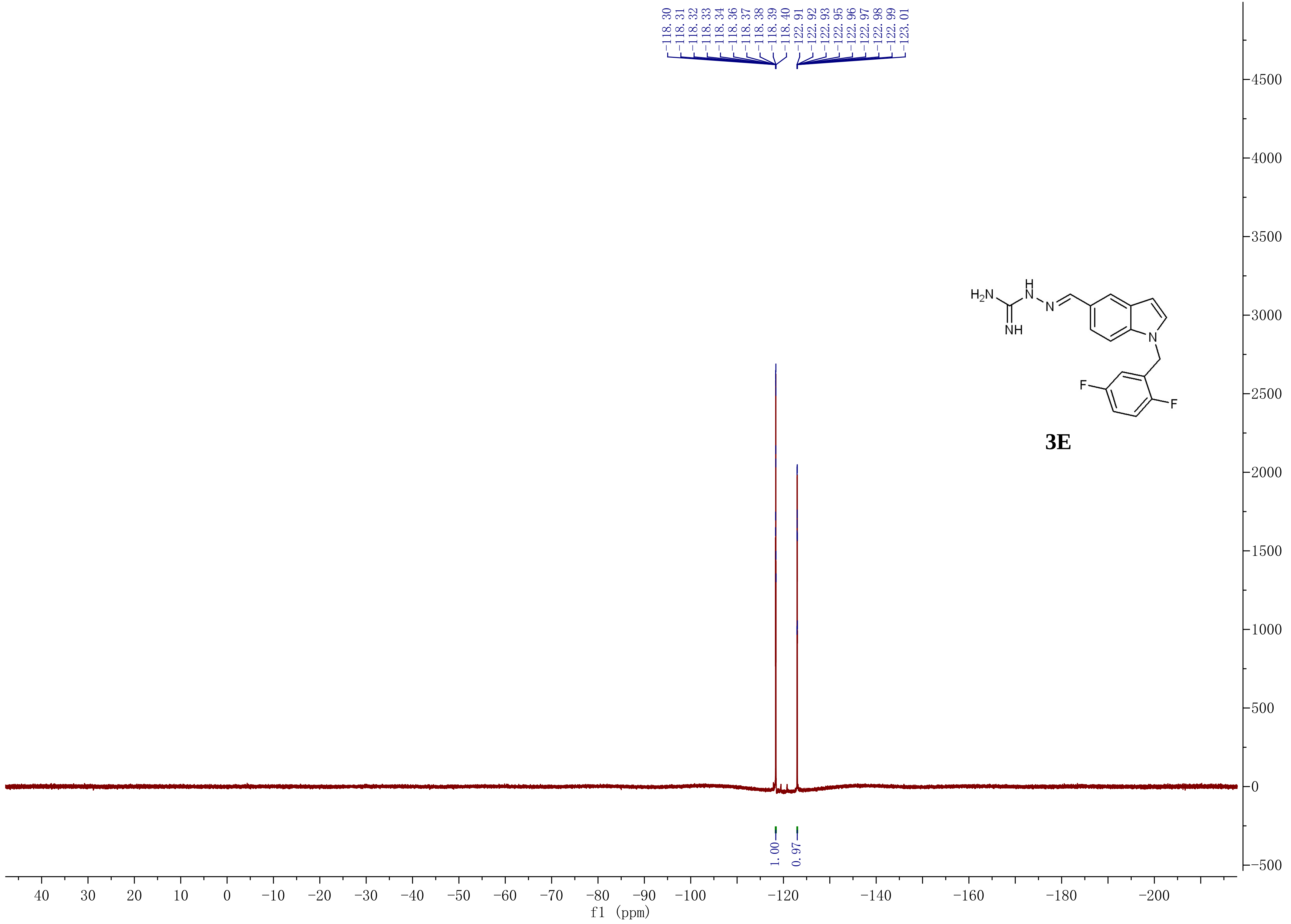

-118.30
-118.31
-118.32
-118.33
-118.34
-118.36
-118.37
-118.38
-118.39
-118.40
-122.91
-122.92
-122.93
-122.95
-122.96
-122.97
-122.98
-122.99
-123.01
H2N
H
N
N
NH
N
F
F
3E
1.00
0.97
40 30 20 10 0 −10 −20 −30 −40 −50 −60 −70 −80 −90 −100 −120 −140 −160 −180 −200
f1 (ppm)
4500 4000 3500 3000 2500 2000 1500 1000 500 0 −500

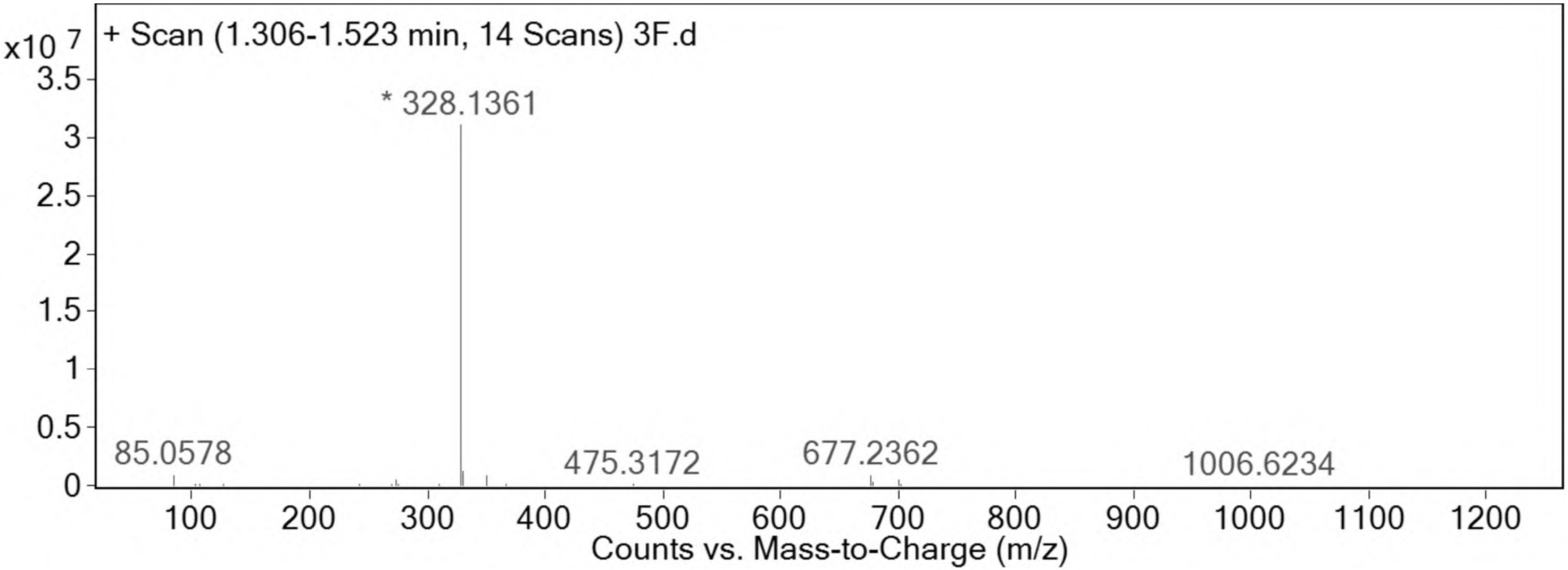

x10 7
+ Scan (1.306-1.523 min, 14 Scans) 3F.d
* 328.1361
85.0578
475.3172
677.2362
1006.6234
3.5
3
2.5
2
1.5
1
0.5
0
100
200
300
400
500
600
700
800
900
1000
1100
1200
Counts vs. Mass-to-Charge (m/z)

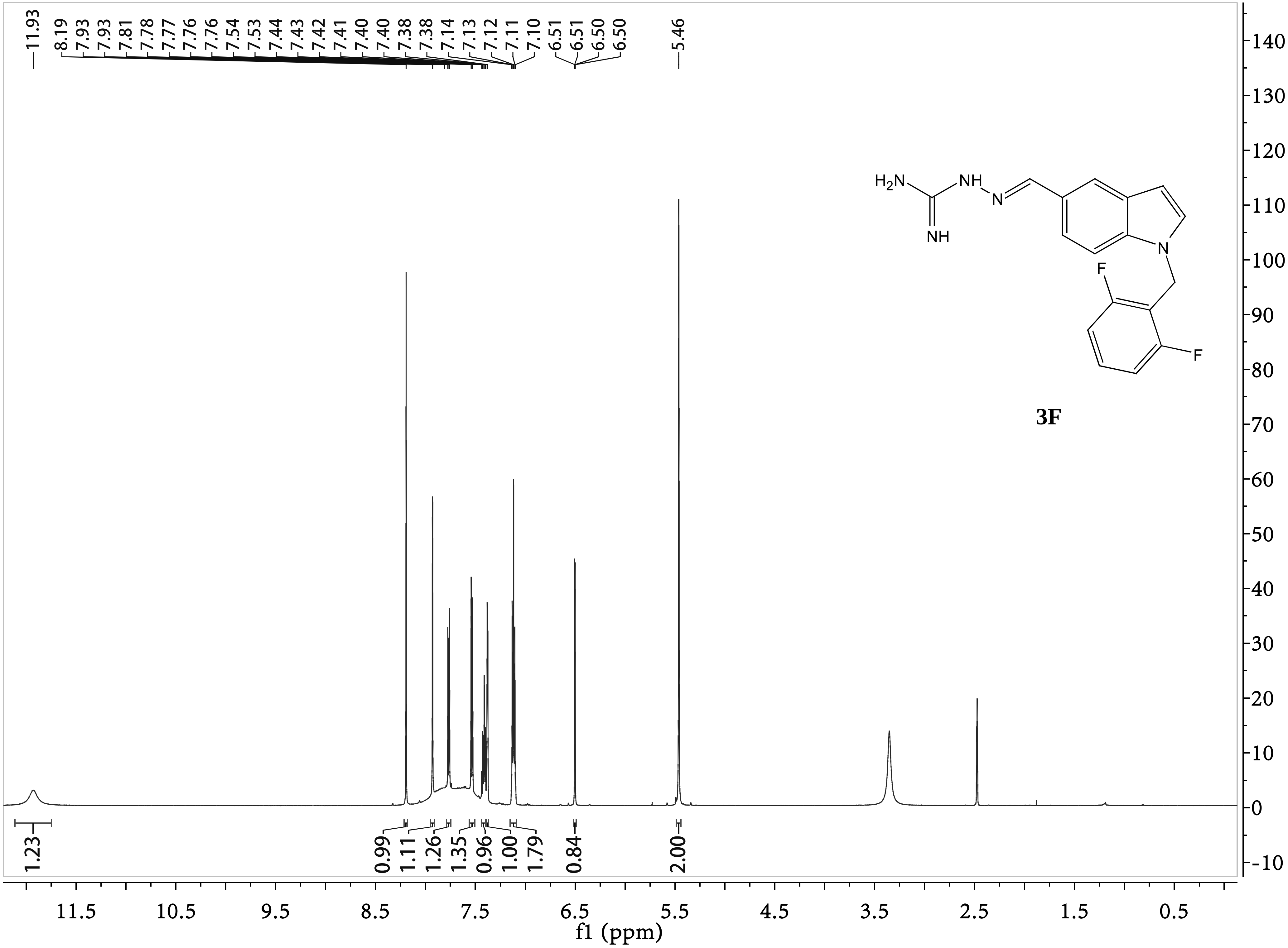
11.93
8.19
7.93
7.93
7.81
7.78
7.77
7.76
7.76
7.54
7.53
7.44
7.43
7.42
7.41
7.40
7.40
7.38
7.38
7.14
7.13
7.12
7.11
7.10
6.51
6.51
6.50
6.50
5.46
H2N
NH
N
NH
N
F
F
1.23
0.99
1.11
1.26
1.35
0.96
1.00
1.79
0.84
2.00
f1 (ppm)

3F

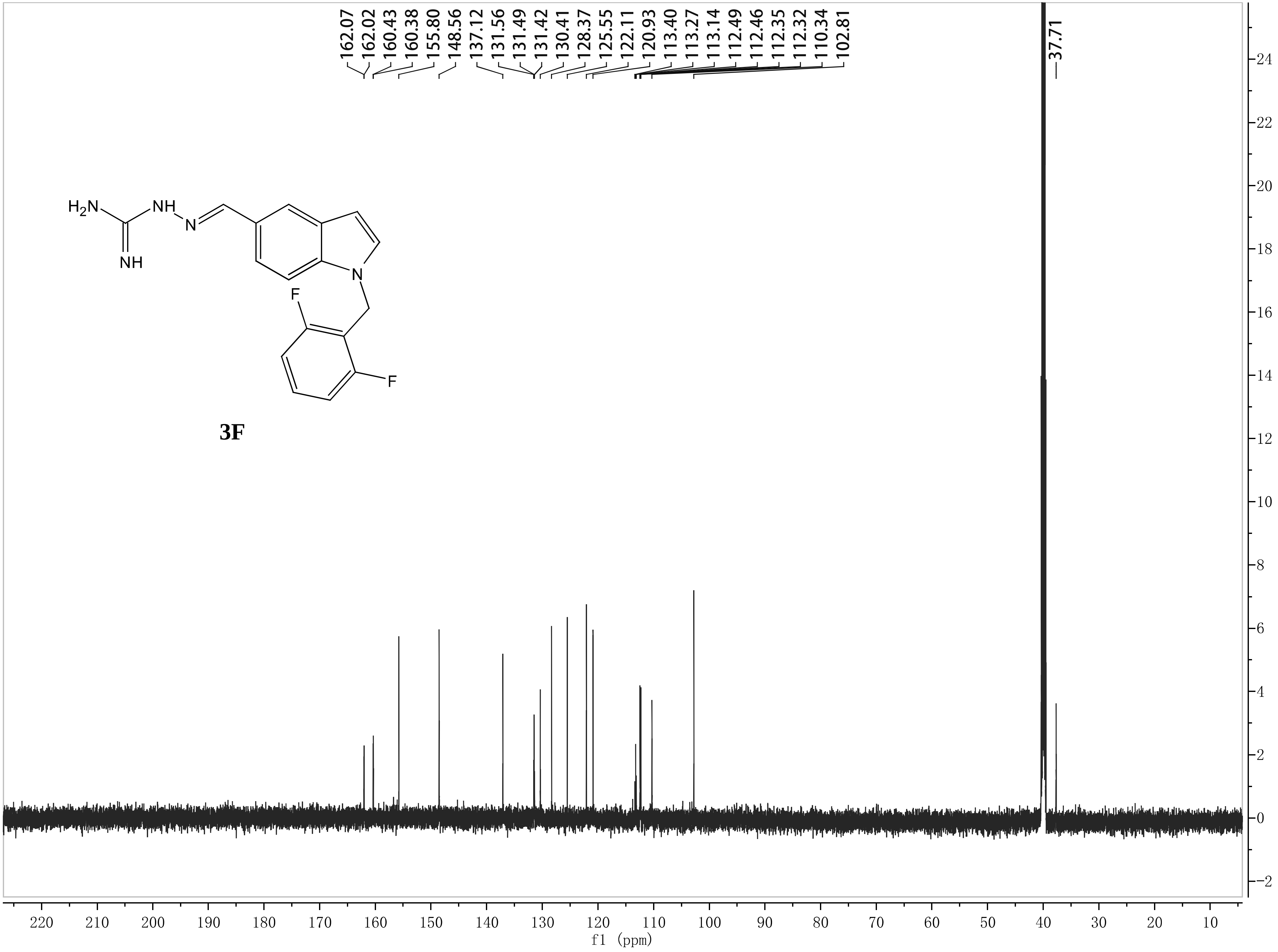
162.07
162.02
160.43
160.38
155.80
148.56
137.12
131.56
131.49
131.42
130.41
128.37
125.55
122.11
120.93
113.40
113.27
113.14
112.49
112.46
112.35
112.32
110.34
102.81
37.71
H2N
NH
N
NH
N
F
F
3F
f1 (ppm)

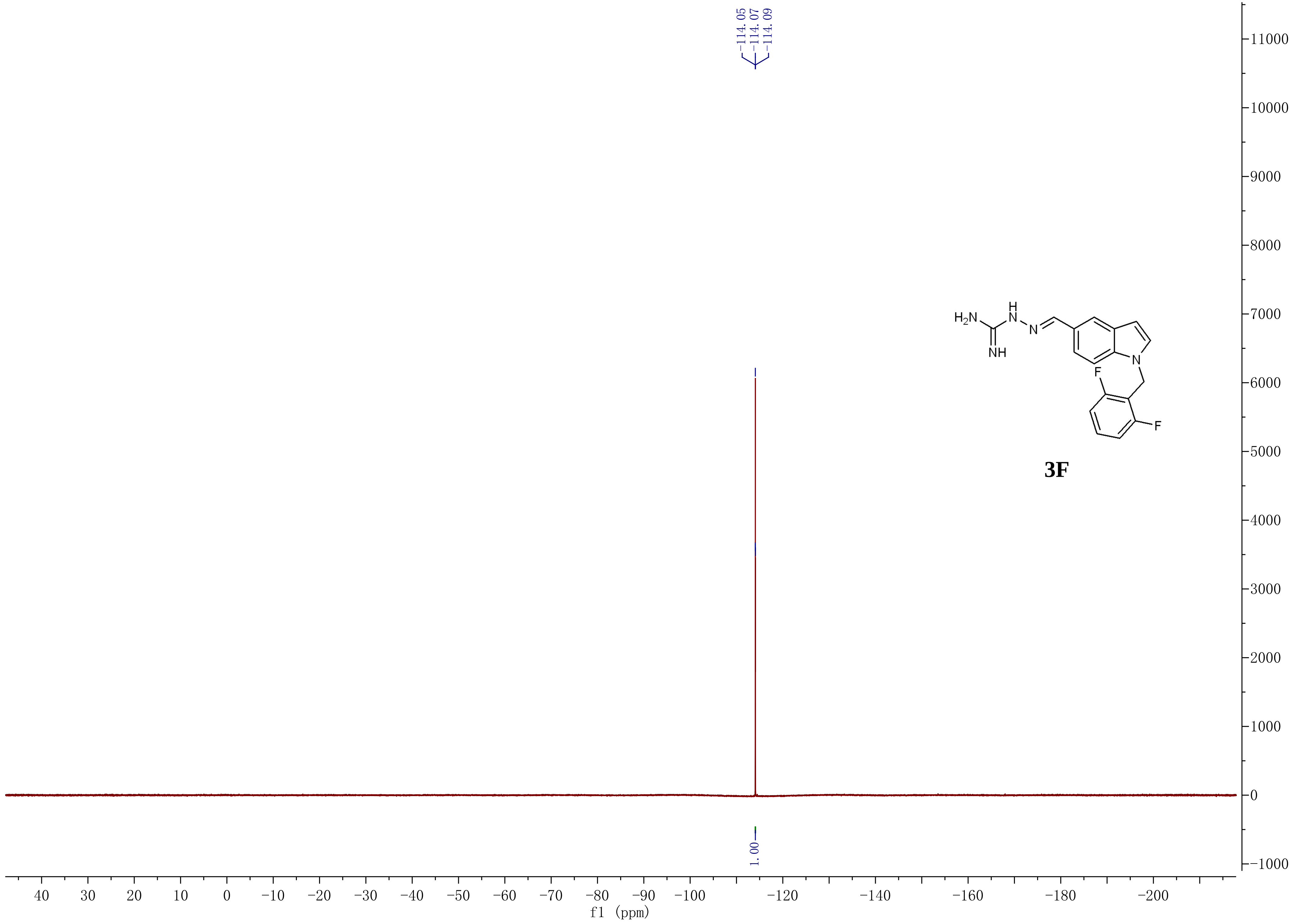
-114.05
-114.07
-114.09
H2N
H
N
N
NH
N
F
F
3F
1.00
40 30 20 10 0 −10 −20 −30 −40 −50 −60 −70 −80 −90 −100 −120 −140 −160 −180 −200
f1 (ppm)
11000
10000
9000
8000
7000
6000
5000
4000
3000
2000
1000
0
−1000

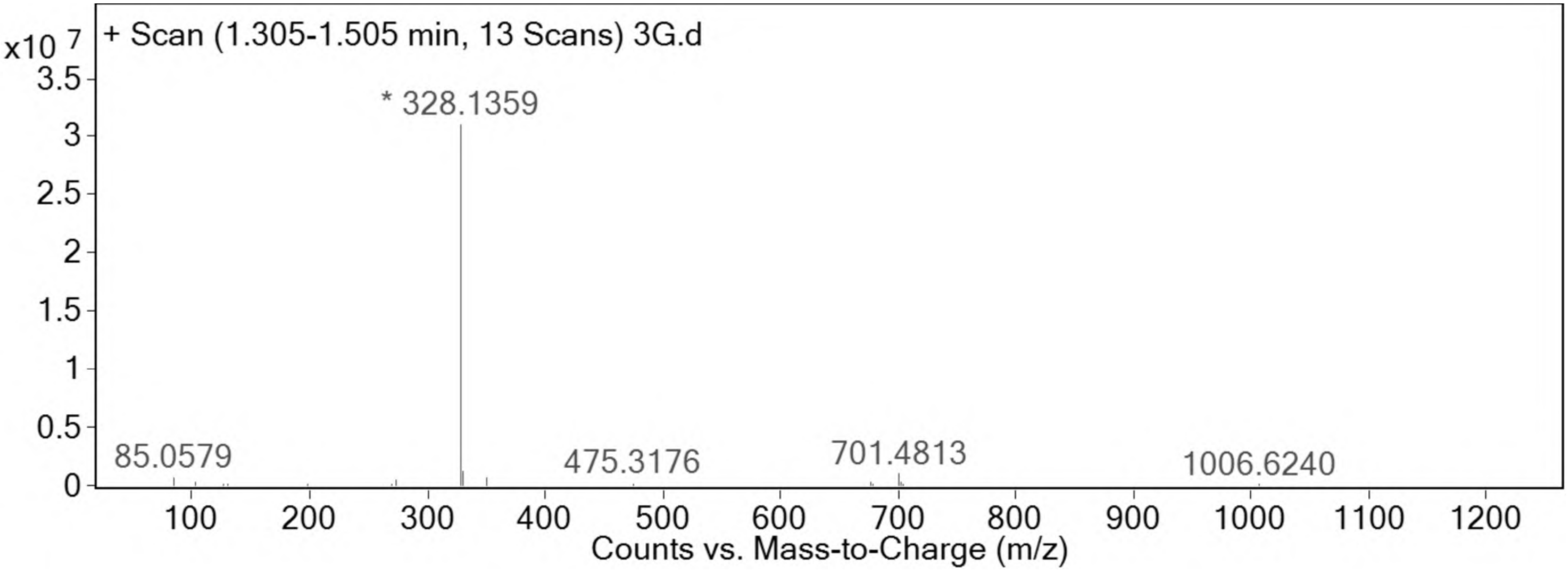
x10 7
+ Scan (1.305-1.505 min, 13 Scans) 3G.d
3.5
3
2.5
2
1.5
1
0.5
0
* 328.1359
85.0579
475.3176
701.4813
1006.6240
100
200
300
400
500
600
700
800
900
1000
1100
1200
Counts vs. Mass-to-Charge (m/z)

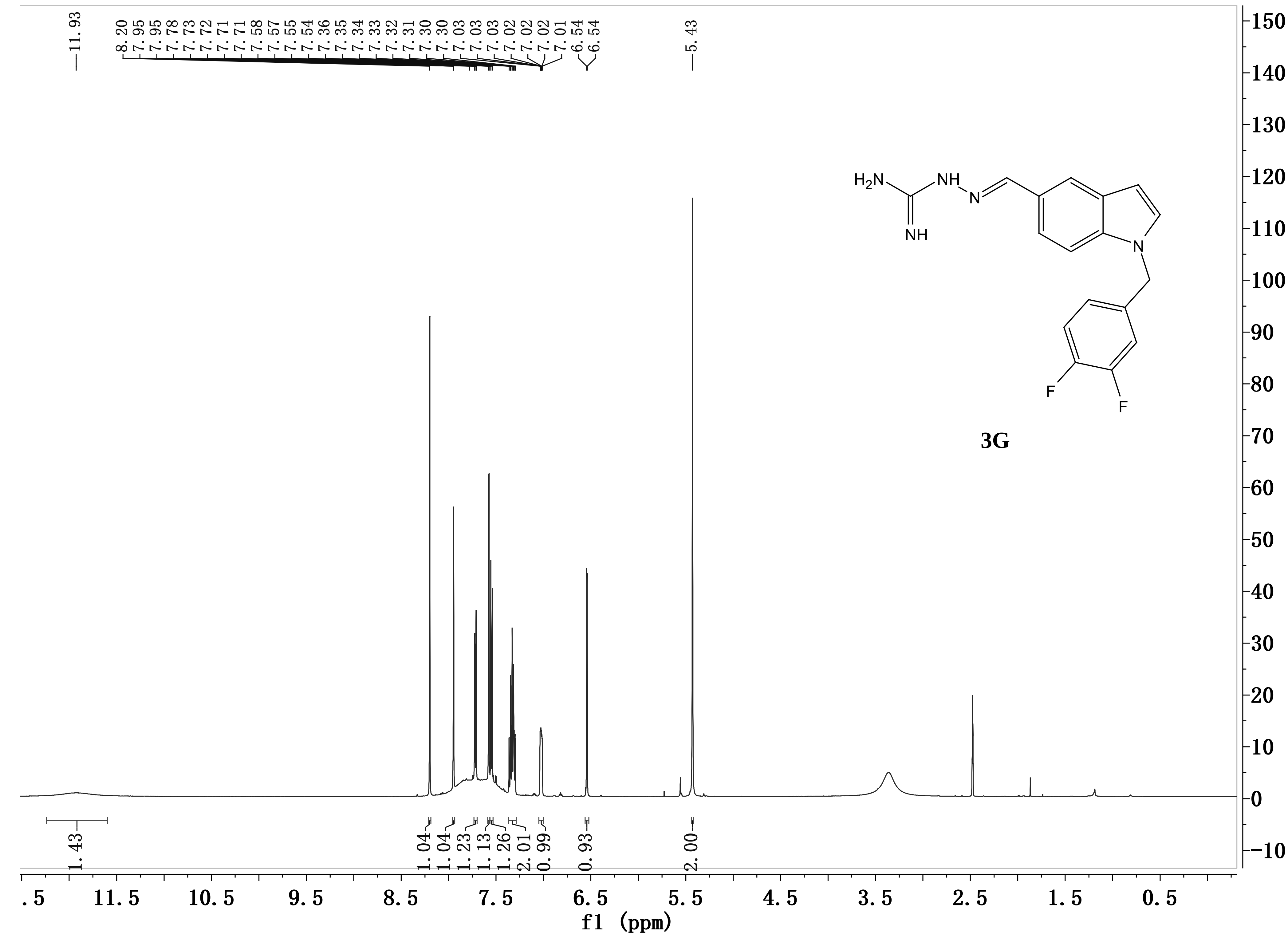
11.93
8.20
7.95
7.95
7.78
7.73
7.72
7.71
7.71
7.58
7.57
7.55
7.54
7.36
7.35
7.34
7.33
7.32
7.31
7.30
7.30
7.03
7.03
7.03
7.02
7.02
7.02
7.01
6.54
6.54
5.43
H_2N
NH
N
NH
N
F
F
3G
1.43
1.04
1.04
1.23
1.13
1.26
2.01
0.99
0.93
2.00
f1 (ppm)

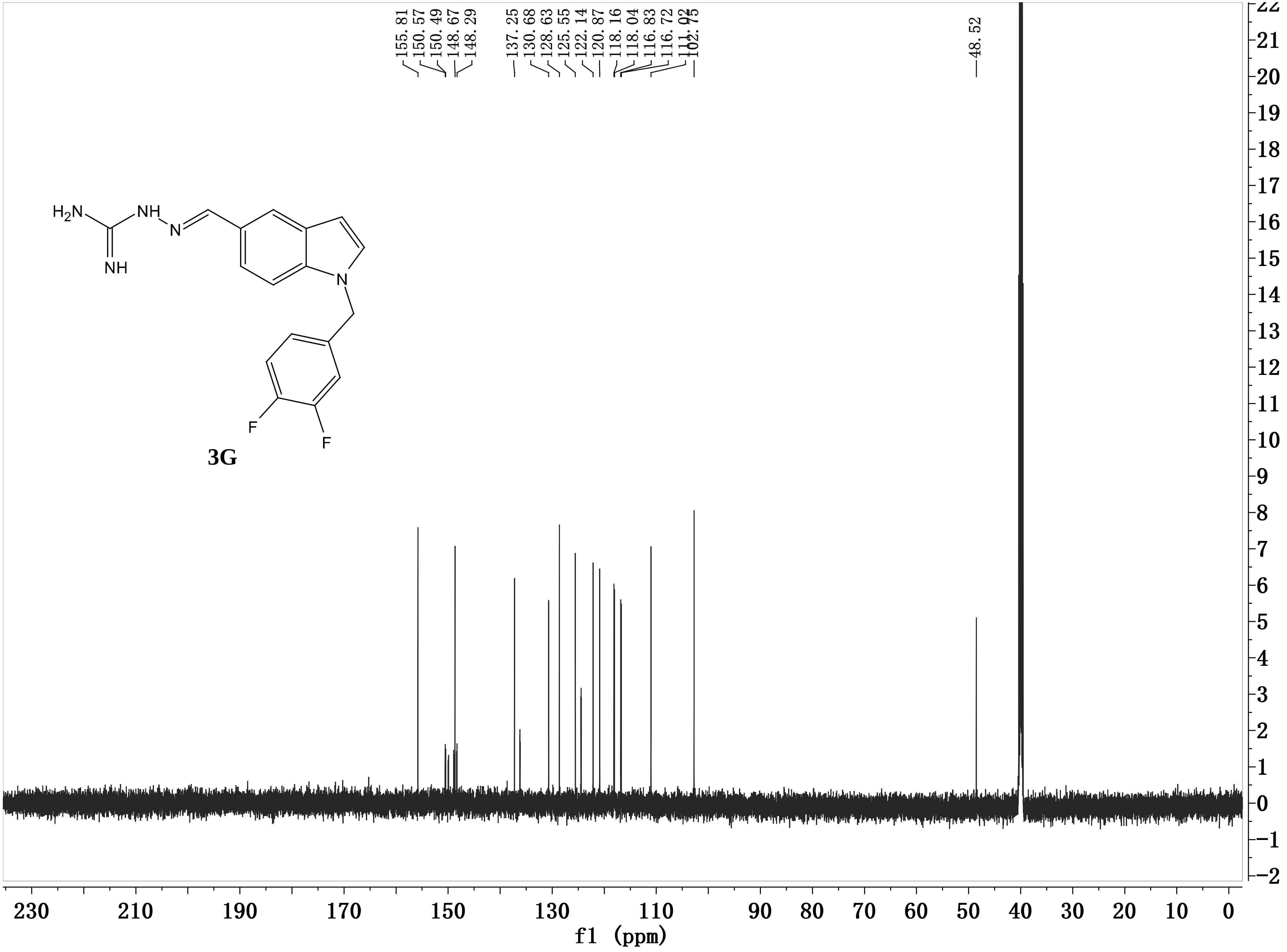

155.81
150.57
150.49
148.67
148.29
137.25
130.68
128.63
125.55
122.14
120.87
118.16
118.04
116.83
116.72
102.75
48.52
H2N
NH
N
NH
N
F
F
3G
f1 (ppm)

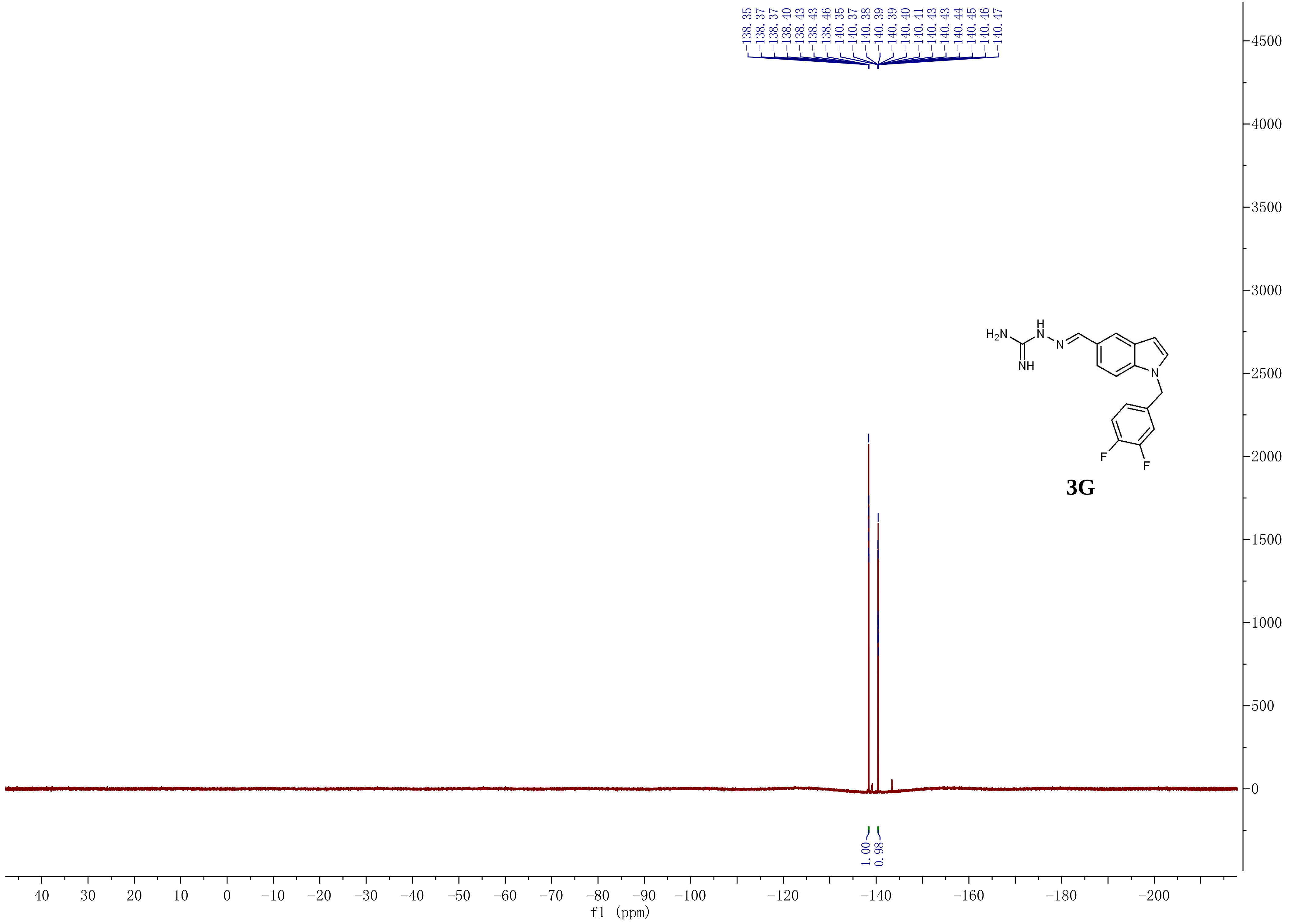

-138.35
-138.37
-138.37
-138.40
-138.43
-138.43
-138.46
-140.35
-140.37
-140.38
-140.39
-140.39
-140.40
-140.41
-140.43
-140.43
-140.44
-140.45
-140.46
-140.47
H2N
H
N
N
NH
N
F
F
3G
1.00
0.98
4500
4000
3500
3000
2500
2000
1500
1000
500
0
40 30 20 10 0 −10 −20 −30 −40 −50 −60 −70 −80 −90 −100 −120 −140 −160 −180 −200
f1 (ppm)

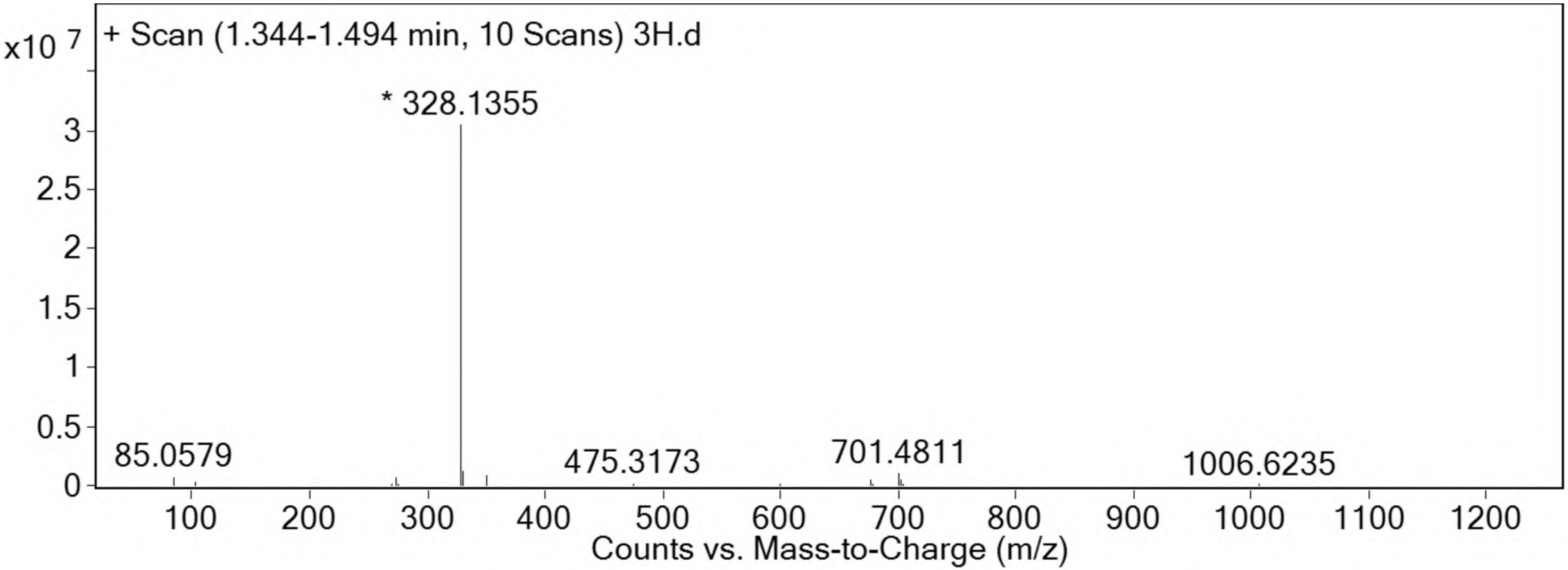

x10 7
+ Scan (1.344-1.494 min, 10 Scans) 3H.d
* 328.1355
3
2.5
2
1.5
1
0.5
0
85.0579
475.3173
701.4811
1006.6235
100
200
300
400
500
600
700
800
900
1000
1100
1200
Counts vs. Mass-to-Charge (m/z)

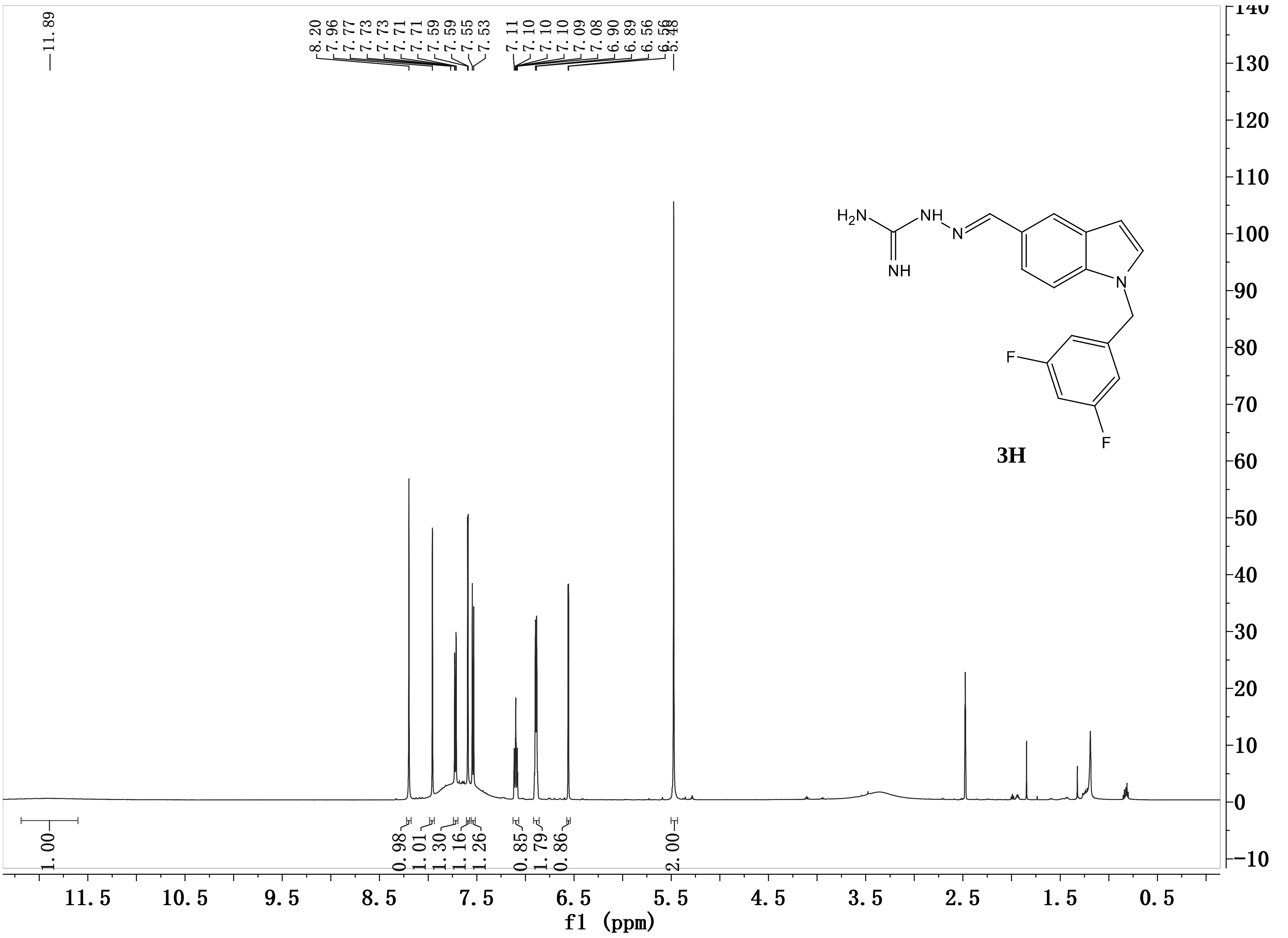

11.89
8.20
7.96
7.77
7.73
7.73
7.71
7.71
7.59
7.59
7.55
7.53
7.11
7.10
7.10
7.10
7.09
7.08
6.90
6.89
6.56
6.56
5.48
1.00
0.98
1.01
1.30
1.16
1.26
0.85
1.79
0.86
2.00
f1 (ppm)
3H

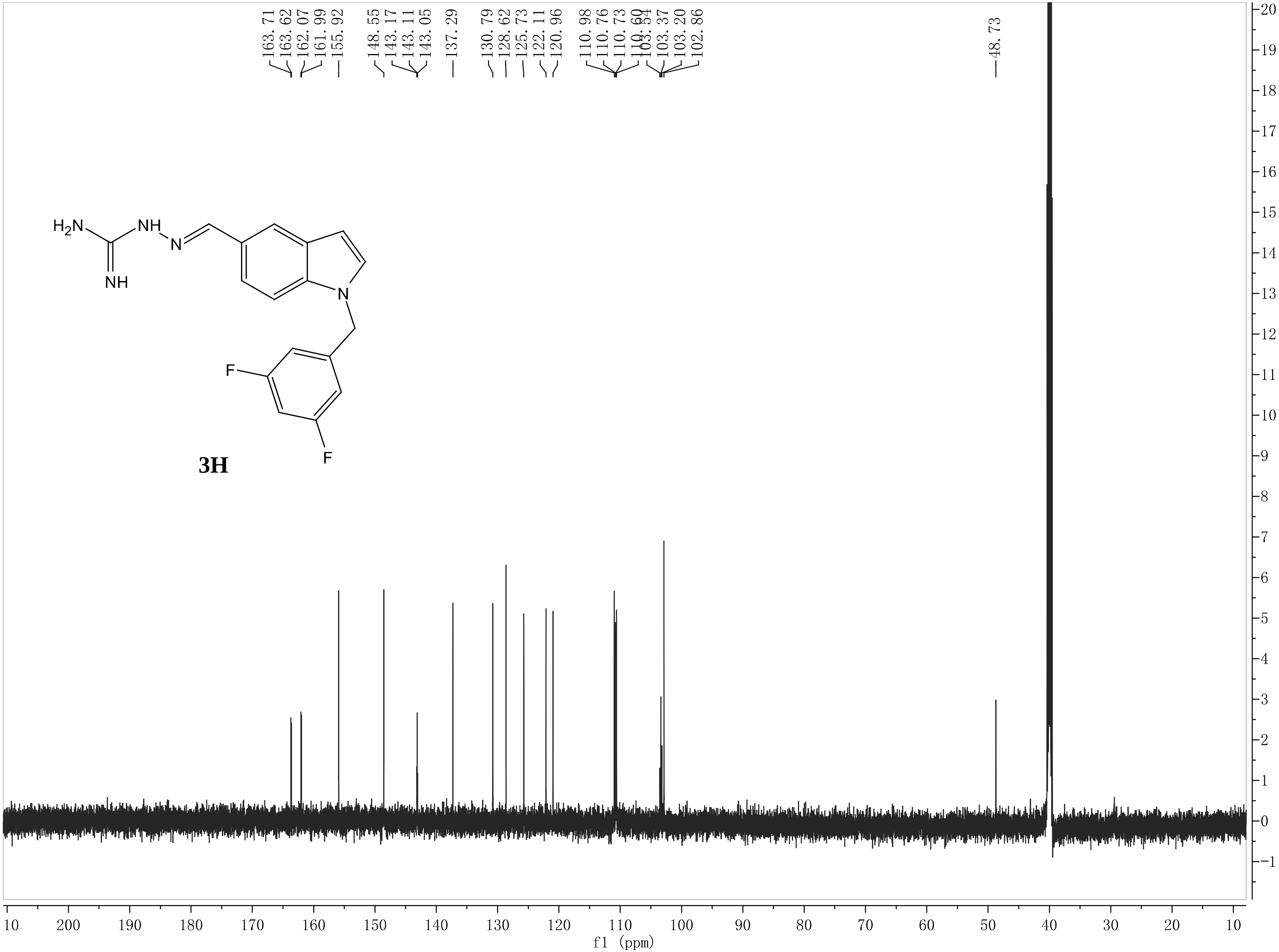
163.71
163.62
162.07
161.99
155.92
148.55
143.17
143.11
143.05
137.29
130.79
128.62
125.73
122.11
120.96
110.98
110.76
110.73
110.60
103.54
103.37
103.20
102.86
48.73
H_2N
NH
N
NH
N
F
F
3H
f1 (ppm)

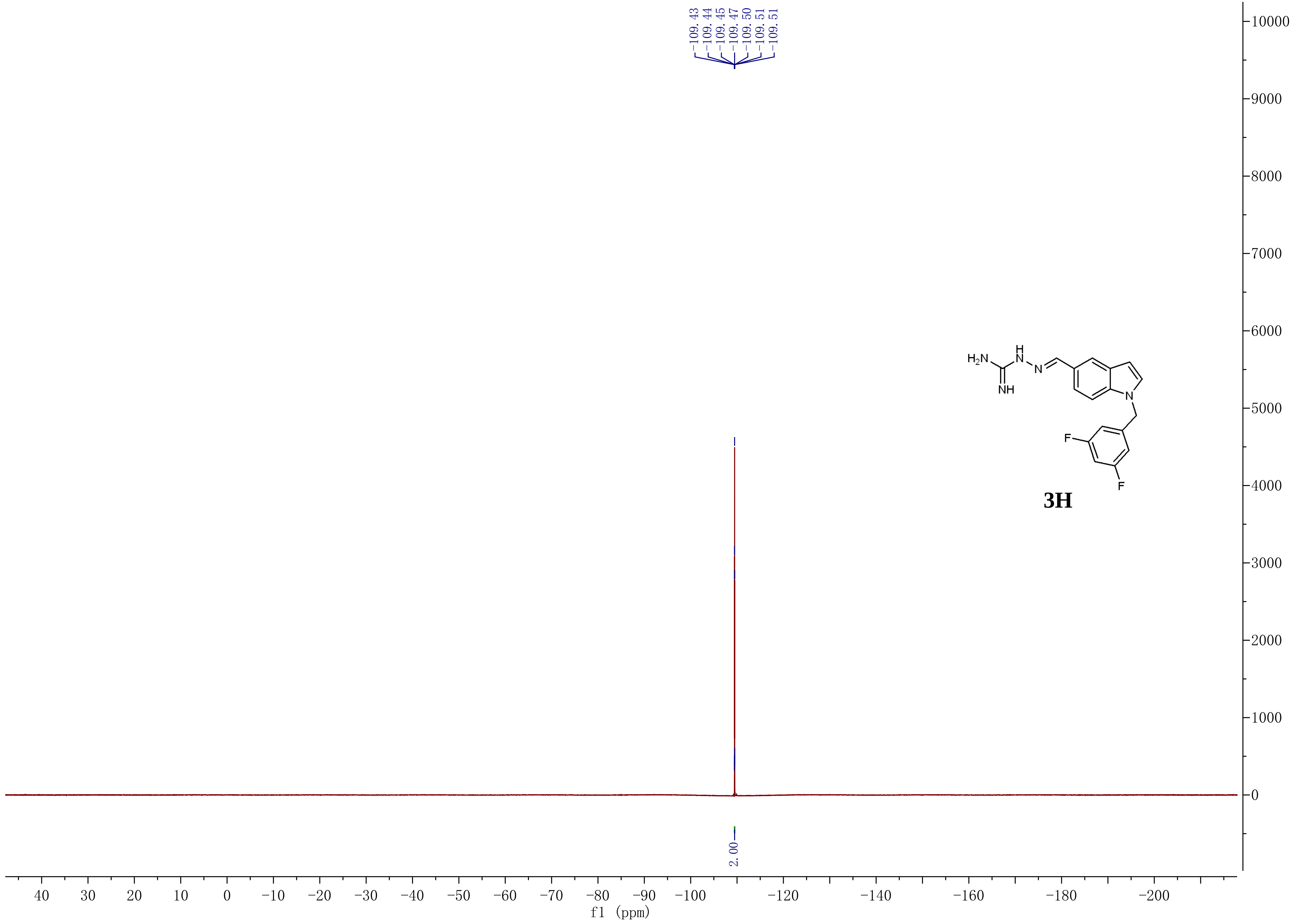

-109.43
-109.44
-109.45
-109.47
-109.50
-109.51
-109.51
H_2N
H
N
N
NH
N
F
F
3H
2.00
10000
9000
8000
7000
6000
5000
4000
3000
2000
1000
0
40 30 20 10 0 −10 −20 −30 −40 −50 −60 −70 −80 −90 −100 −120 −140 −160 −180 −200
f1 (ppm)

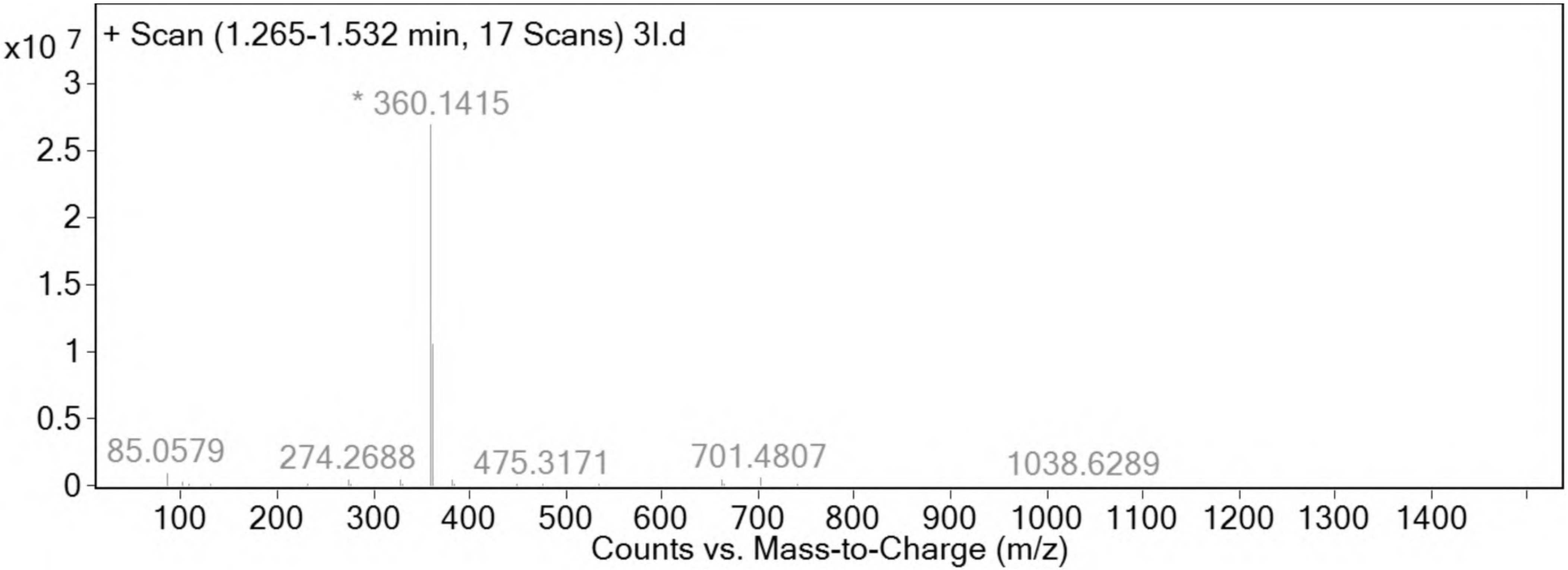

x10 7
+ Scan (1.265-1.532 min, 17 Scans) 3l.d
3
2.5
2
1.5
1
0.5
0
* 360.1415
85.0579
274.2688
475.3171
701.4807
1038.6289
100
200
300
400
500
600
700
800
900
1000
1100
1200
1300
1400
Counts vs. Mass-to-Charge (m/z)

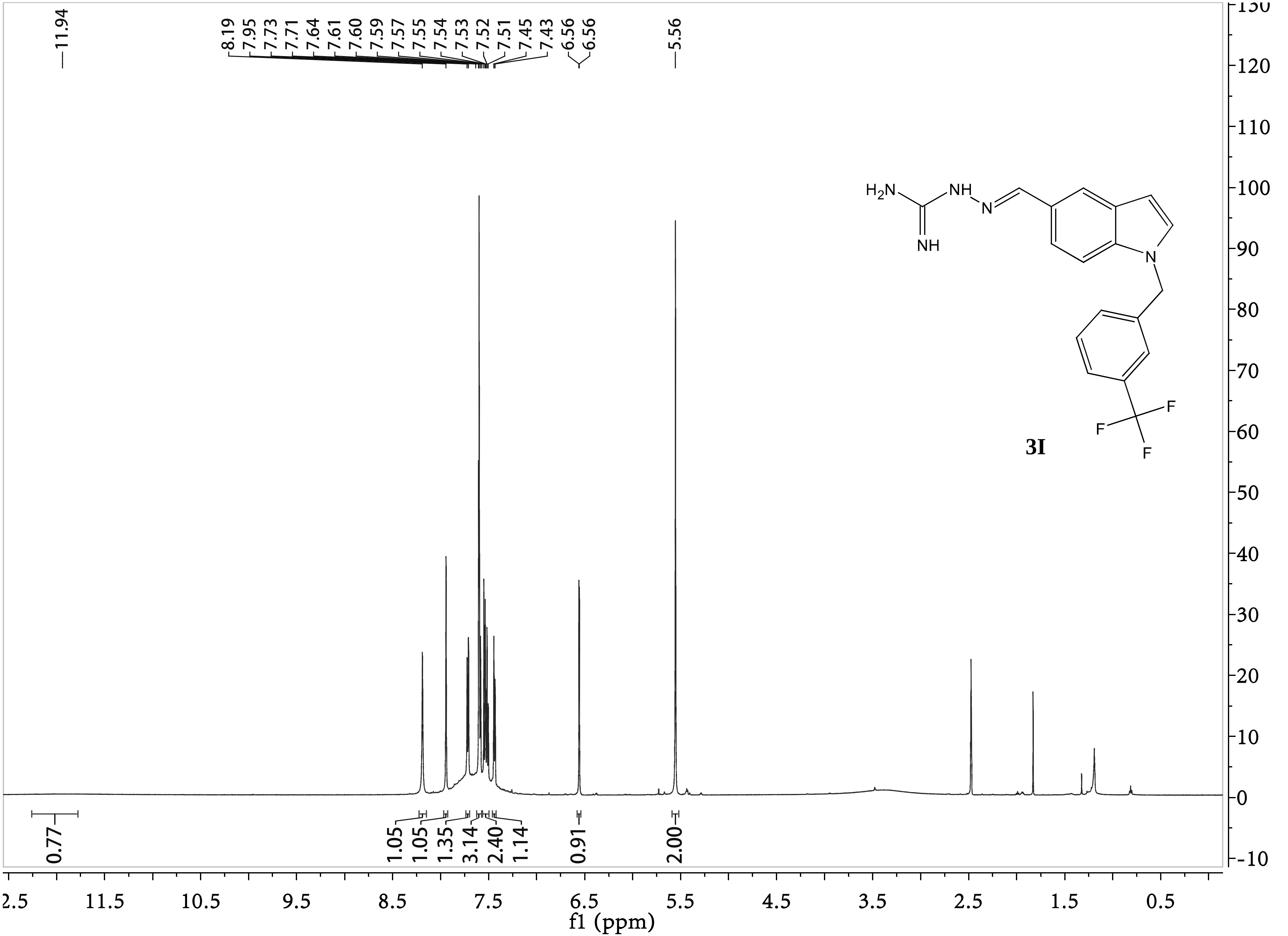

11.94
8.19
7.95
7.73
7.71
7.64
7.61
7.60
7.59
7.57
7.55
7.54
7.53
7.52
7.51
7.45
7.43
6.56
6.56
5.56
H2N
NH
N
NH
N
F
F
F
3I
0.77
1.05
1.05
1.35
3.14
2.40
1.14
0.91
2.00
f1 (ppm)

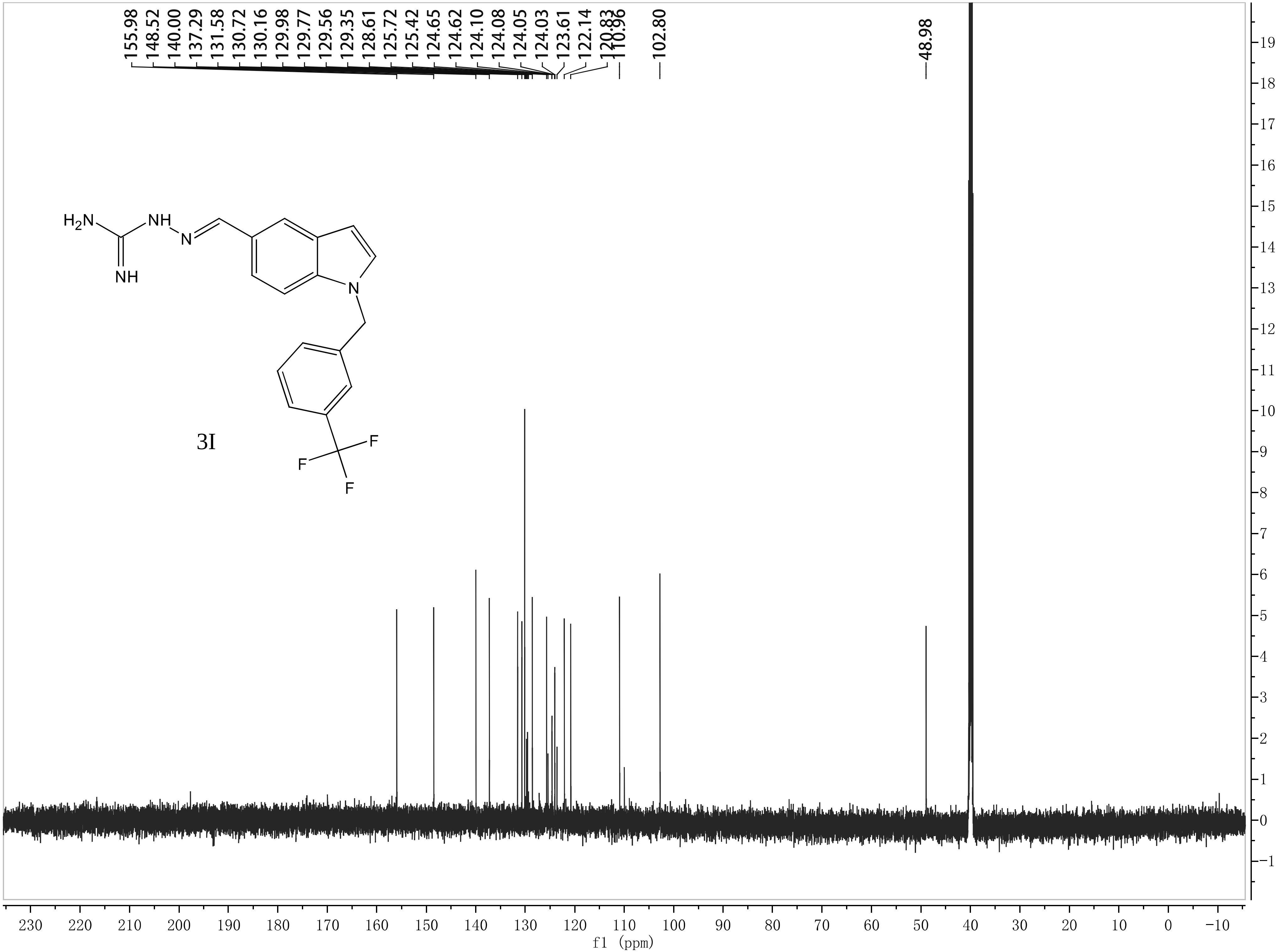
155.98
148.52
140.00
137.29
131.58
130.72
130.16
129.98
129.77
129.56
129.35
128.61
125.72
125.42
124.65
124.62
124.10
124.08
124.05
124.03
123.61
122.14
120.83
110.96
102.80
48.98
H2N
NH
N
NH
N
F
F
F
3I
230 220 210 200 190 180 170 160 150 140 130 120 110 100 90 80 70 60 50 40 30 20 10 0 −10
f1 (ppm)
19 18 17 16 15 14 13 12 11 10 9 8 7 6 5 4 3 2 1 0 −1

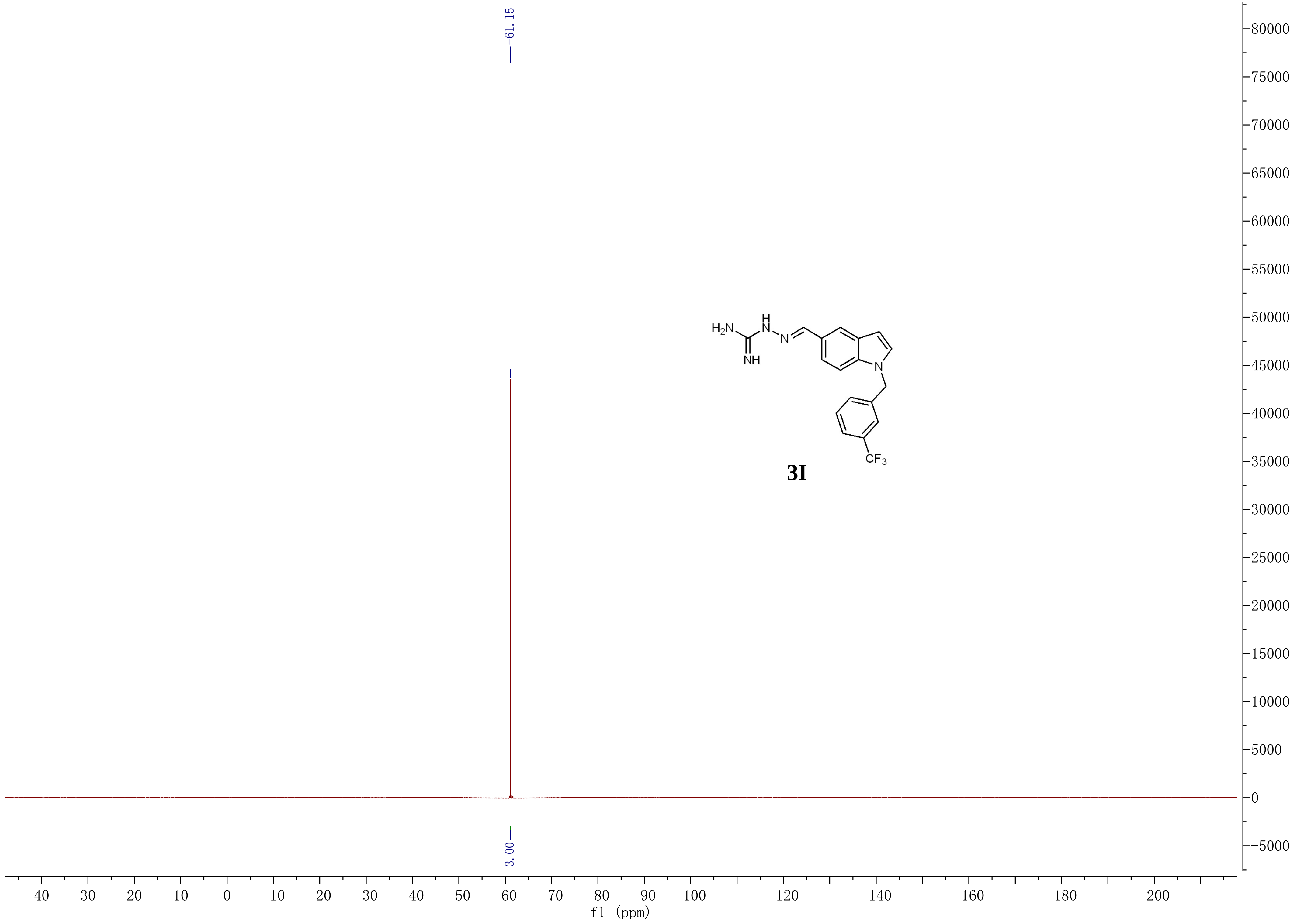

-61.15
3.00
f1 (ppm)
40 30 20 10 0 −10 −20 −30 −40 −50 −60 −70 −80 −90 −100 −120 −140 −160 −180 −200
80000 75000 70000 65000 60000 55000 50000 45000 40000 35000 30000 25000 20000 15000 10000 5000 0 −5000
H2N
H
N
N
NH
N
CF3
3I

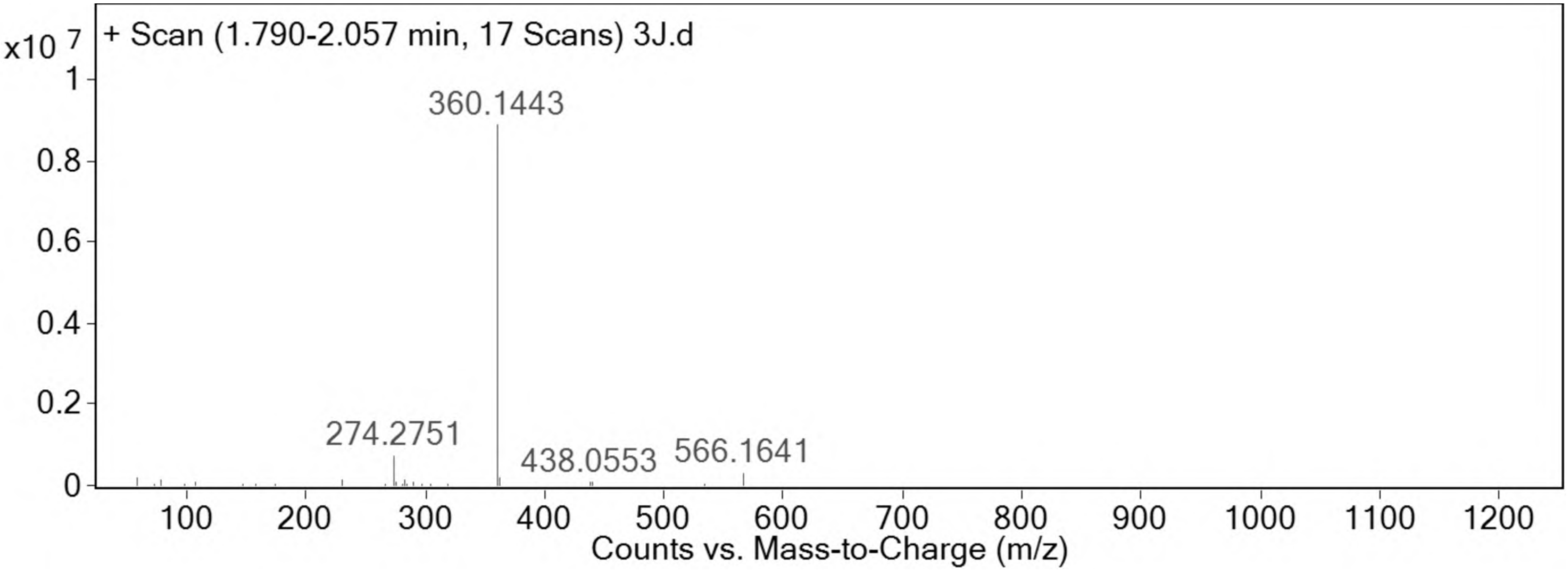

x10 7
+ Scan (1.790-2.057 min, 17 Scans) 3J.d
1
0.8
0.6
0.4
0.2
0
360.1443
274.2751
438.0553
566.1641
100
200
300
400
500
600
700
800
900
1000
1100
1200
Counts vs. Mass-to-Charge (m/z)

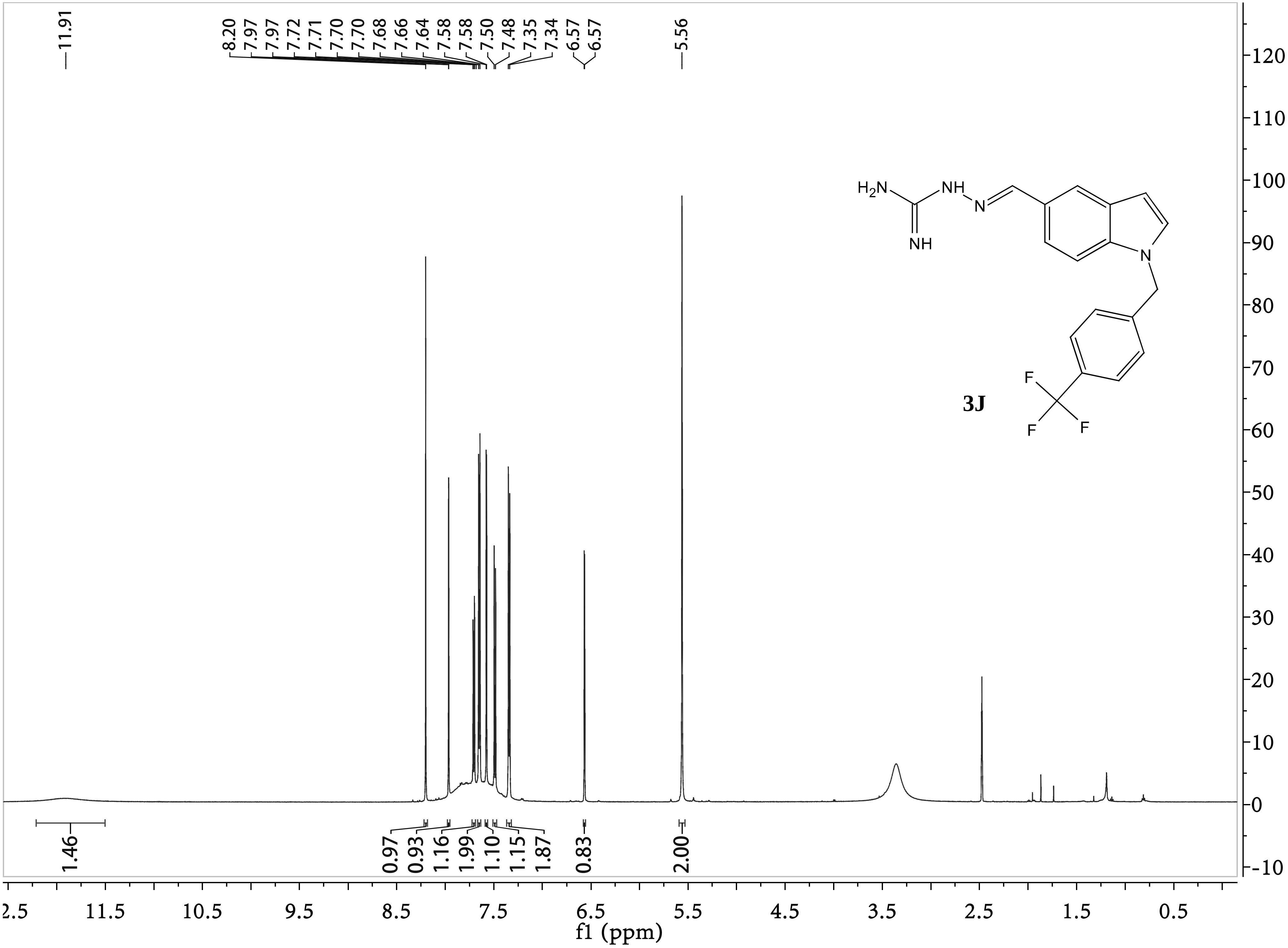

11.91
8.20
7.97
7.97
7.72
7.71
7.70
7.70
7.68
7.66
7.64
7.58
7.58
7.50
7.48
7.35
7.34
6.57
6.57
5.56
H2N
NH
N
NH
N
F
F
F
1.46
0.97
0.93
1.16
1.99
1.10
1.15
1.87
0.83
2.00
f1 (ppm)

3J

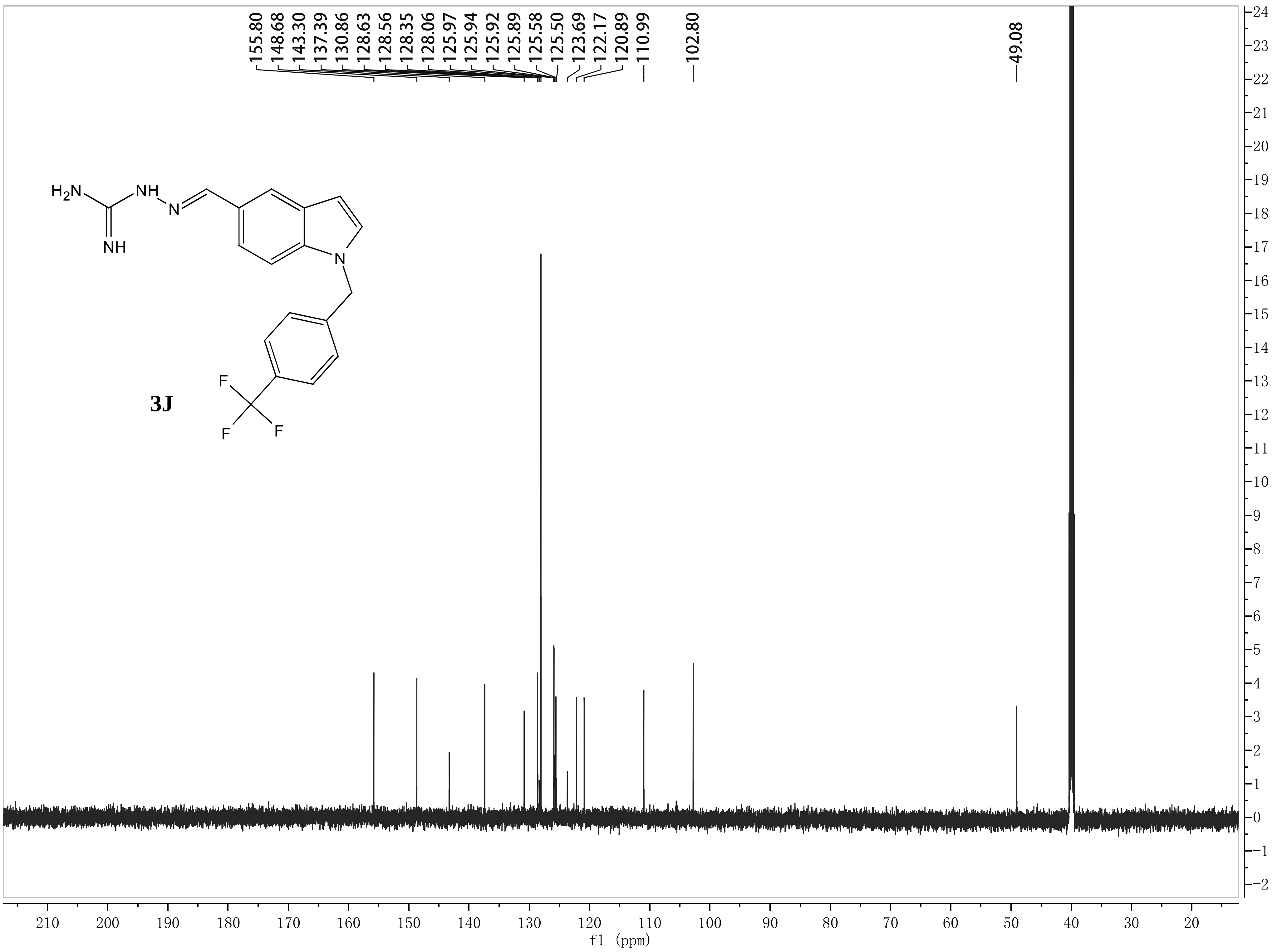

155.80
148.68
143.30
137.39
130.86
128.63
128.56
128.35
128.06
125.97
125.94
125.92
125.89
125.58
125.50
123.69
122.17
120.89
110.99
102.80
49.08
3J
f1 (ppm)

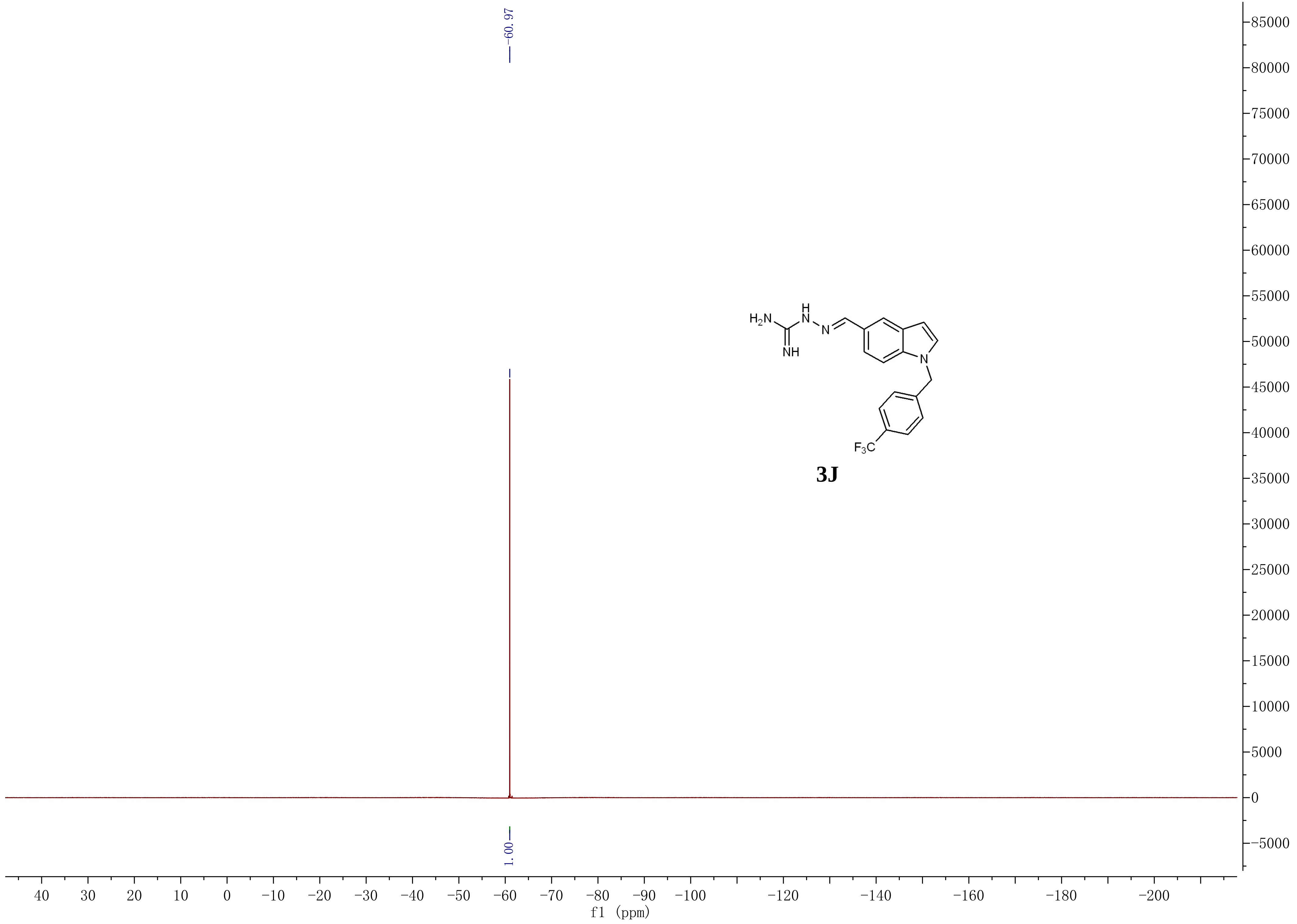
-60.97
1.00
3J
H2N
NH
F3C
f1 (ppm)

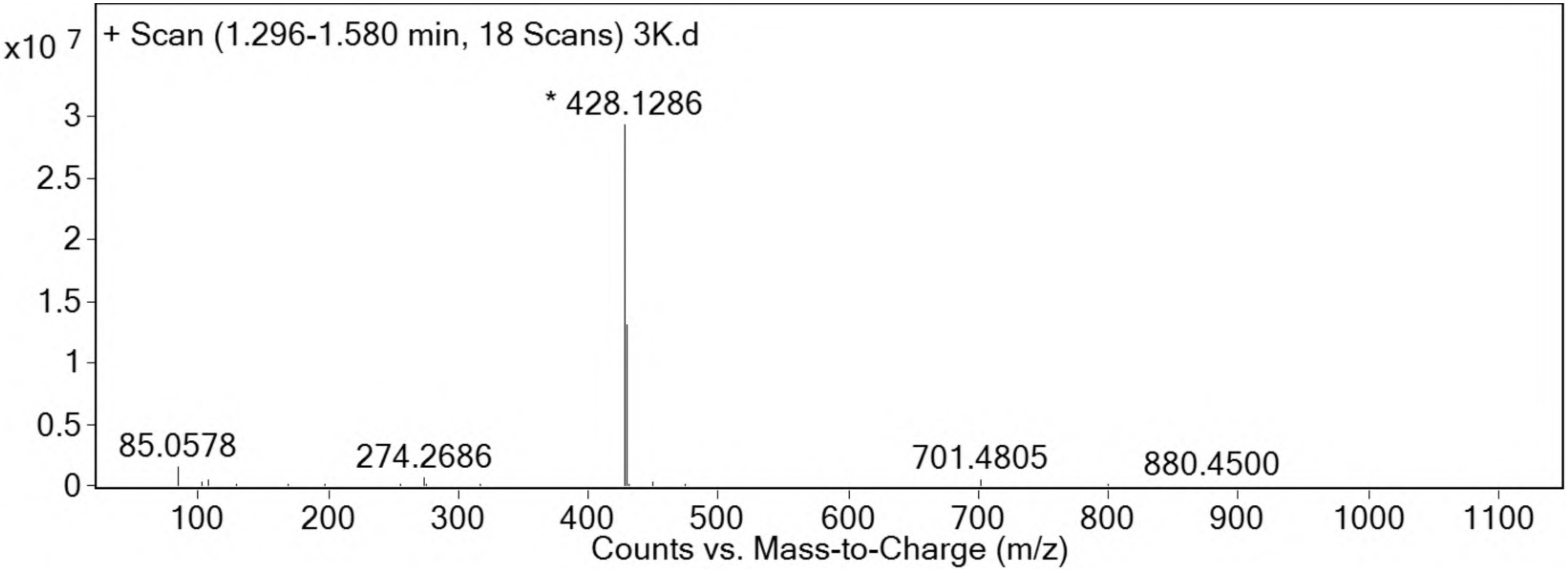
x10 7
+ Scan (1.296-1.580 min, 18 Scans) 3K.d
* 428.1286
85.0578
274.2686
701.4805
880.4500
3
2.5
2
1.5
1
0.5
0
100
200
300
400
500
600
700
800
900
1000
1100
Counts vs. Mass-to-Charge (m/z)

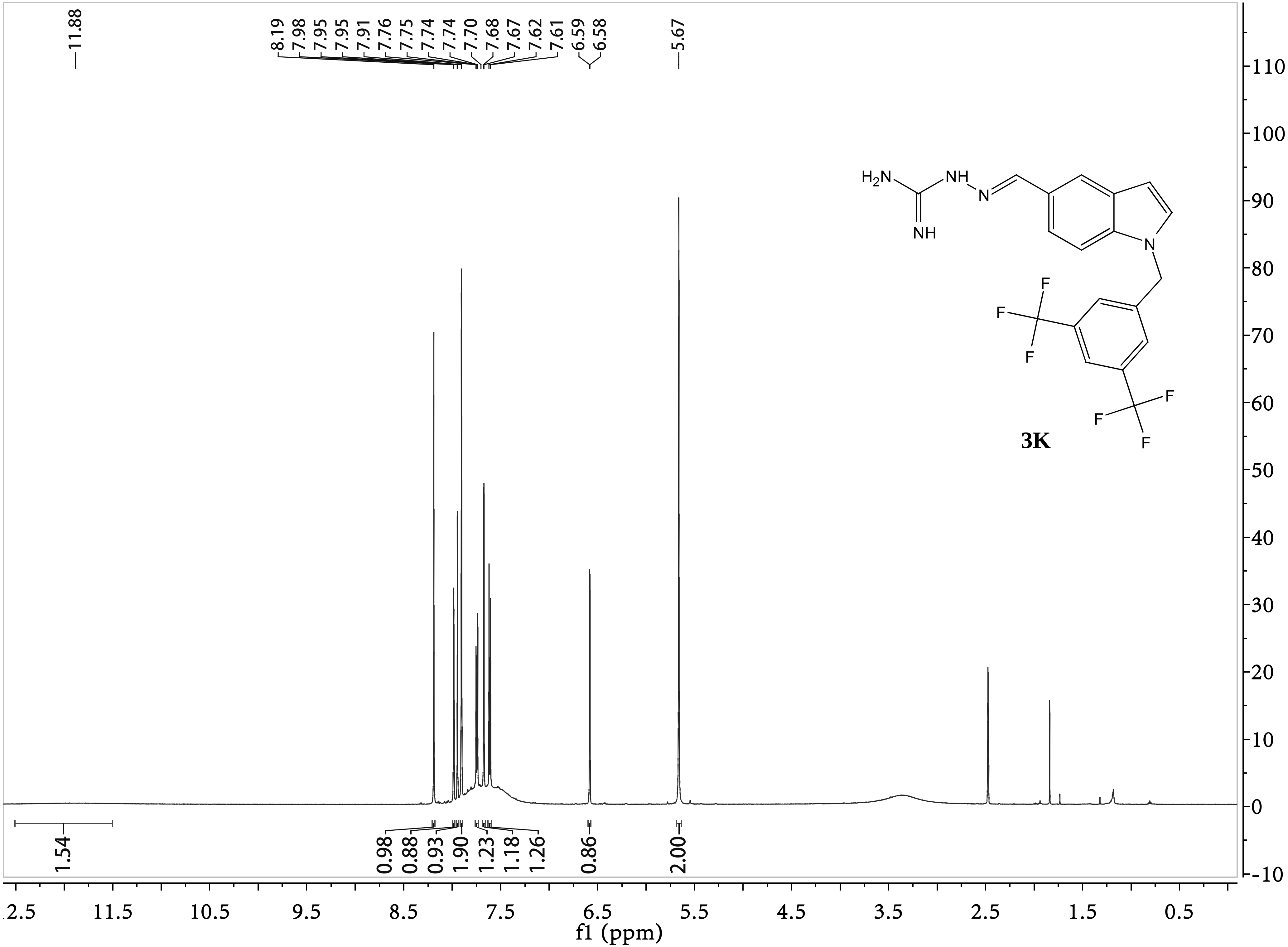
11.88
8.19 7.98 7.95 7.95 7.91 7.76 7.75 7.74 7.74 7.70 7.68 7.67 7.62 7.61 6.59 6.58
5.67
1.54
0.98 0.88 0.93 1.90 1.23 1.18 1.26
0.86
2.00
3K
f1 (ppm)

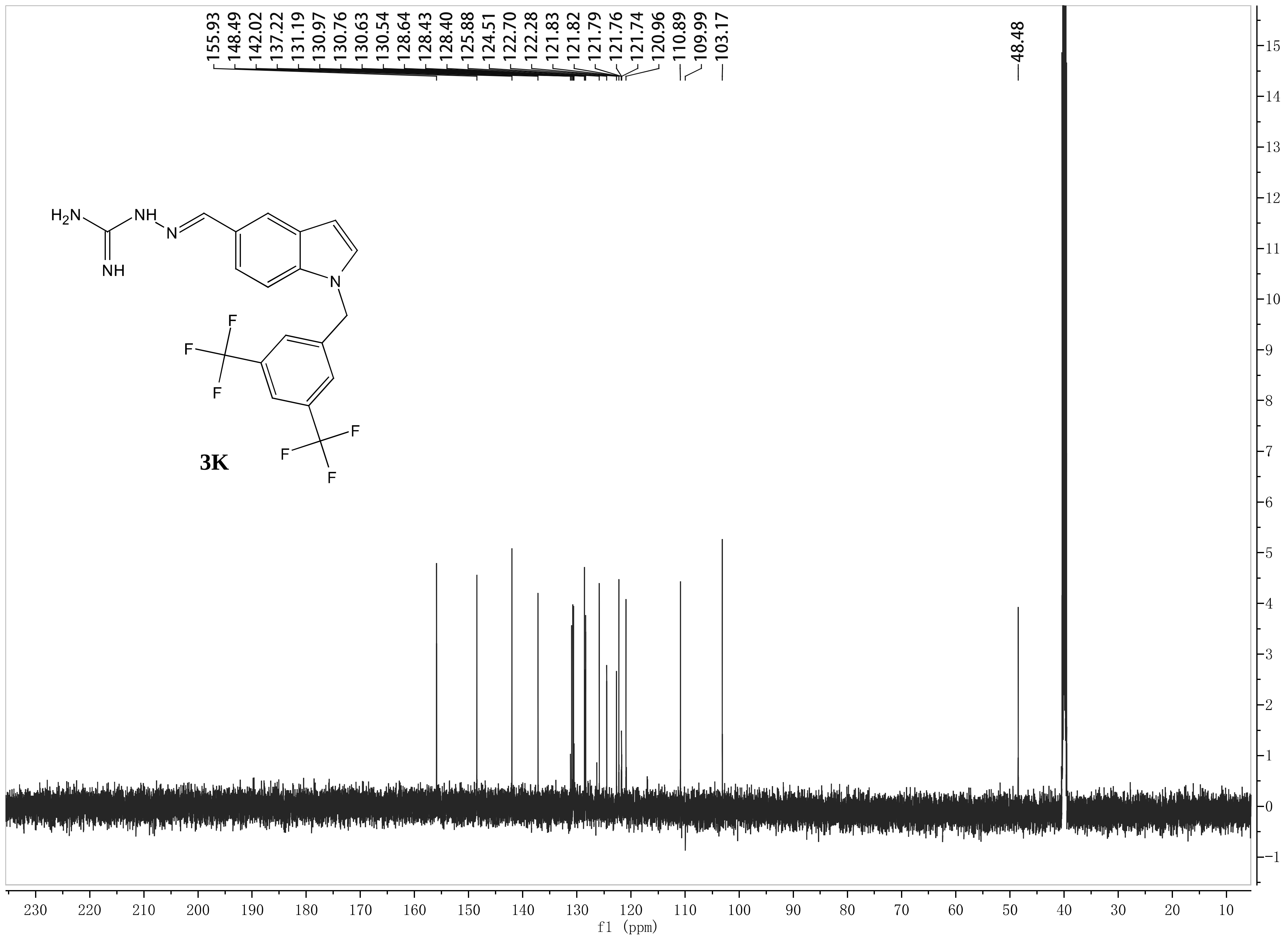
155.93
148.49
142.02
137.22
131.19
130.97
130.76
130.63
130.54
128.64
128.43
128.40
125.88
124.51
122.70
122.28
121.83
121.82
121.79
121.76
121.74
120.96
110.89
109.99
103.17
48.48
H_2N
NH
N
NH
N
F
3K
f1 (ppm)

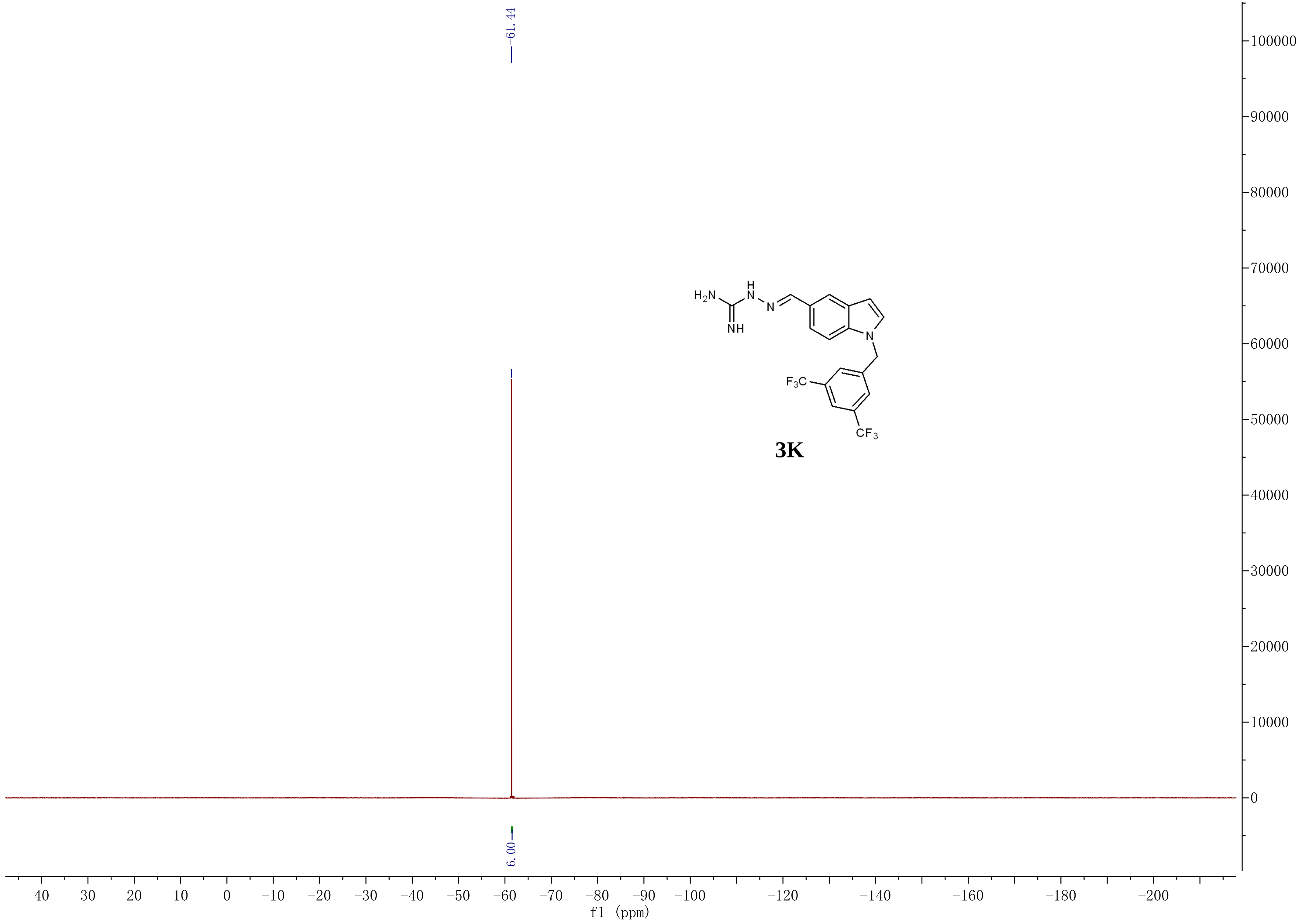

−61.44
H2N
H
N
N
NH
N
F3C
CF3
3K
6.00
100000
90000
80000
70000
60000
50000
40000
30000
20000
10000
0
40 30 20 10 0 −10 −20 −30 −40 −50 −60 −70 −80 −90 −100 −120 −140 −160 −180 −200
f1 (ppm)

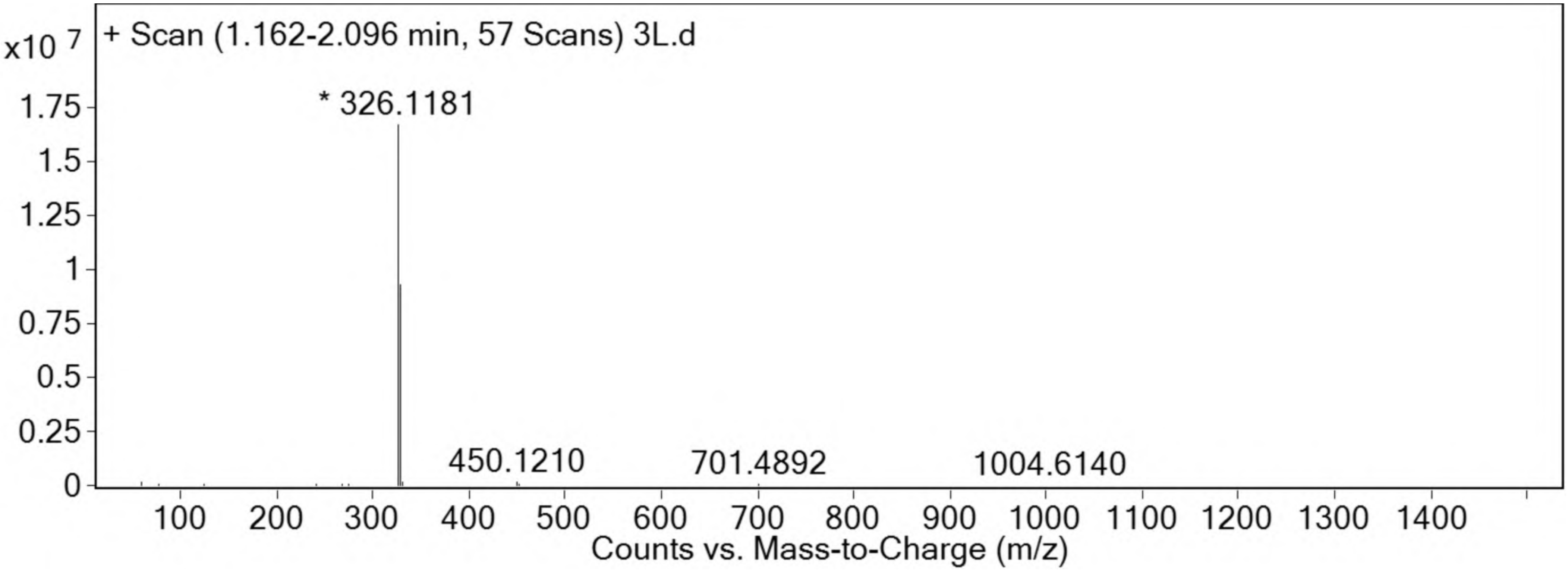
x10 7
+ Scan (1.162-2.096 min, 57 Scans) 3L.d
1.75
1.5
1.25
1
0.75
0.5
0.25
0
* 326.1181
450.1210
701.4892
1004.6140
100
200
300
400
500
600
700
800
900
1000
1100
1200
1300
1400
Counts vs. Mass-to-Charge (m/z)

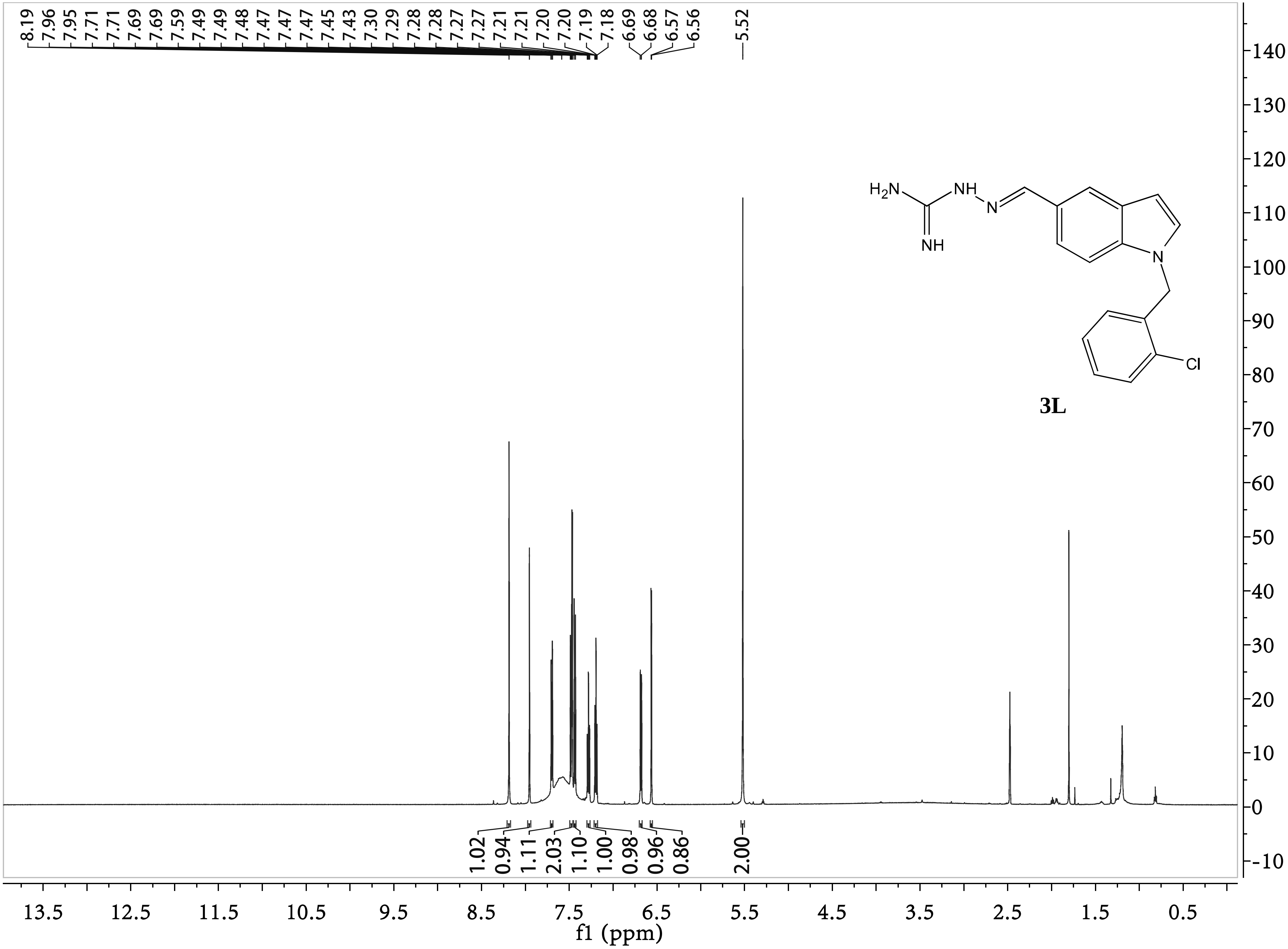
8.19 7.96 7.95 7.71 7.71 7.69 7.69 7.59 7.49 7.49 7.48 7.47 7.47 7.47 7.45 7.43 7.30 7.29 7.28 7.28 7.27 7.27 7.21 7.21 7.20 7.20 7.19 7.18 6.69 6.68 6.57 6.56
5.52
1.02 0.94 1.11 2.03 1.10 1.00 0.98 0.96 0.86
2.00
H2N
NH
N
NH
N
Cl
3L
f1 (ppm)

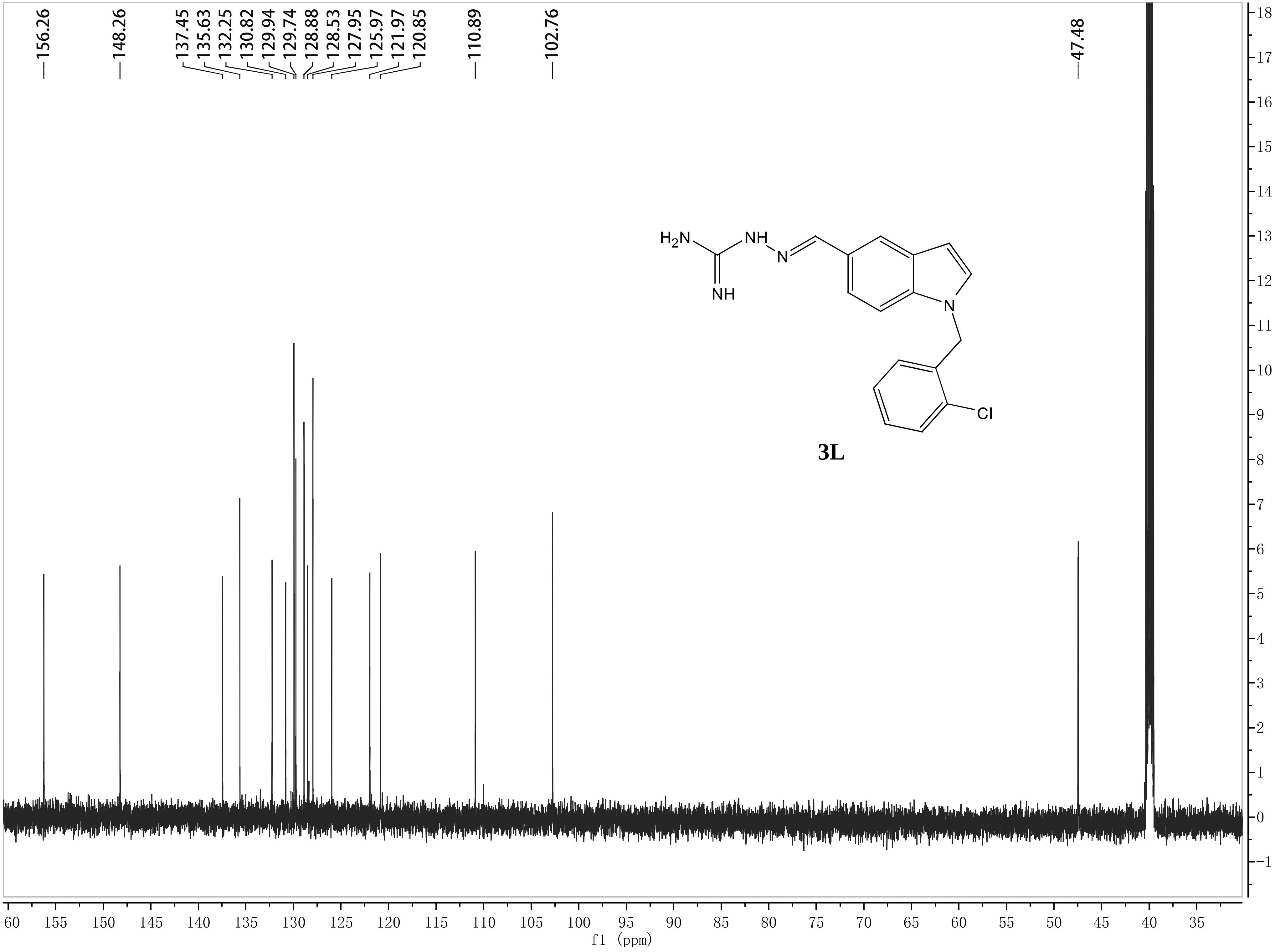
156.26
148.26
137.45
135.63
132.25
130.82
129.94
129.74
128.88
128.53
127.95
125.97
121.97
120.85
110.89
102.76
47.48
H2N
NH
N
NH
N
Cl
3L
f1 (ppm)

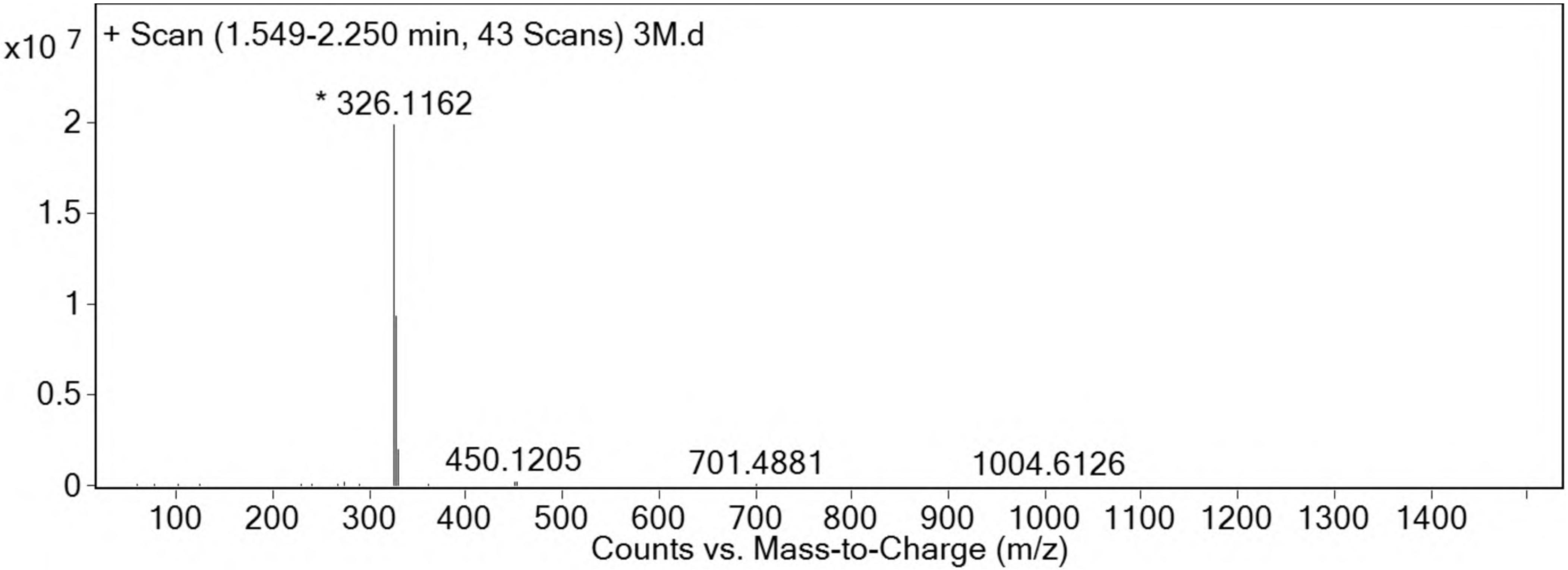

x10 7
+ Scan (1.549-2.250 min, 43 Scans) 3M.d
* 326.1162
450.1205
701.4881
1004.6126
2
1.5
1
0.5
0
100 200 300 400 500 600 700 800 900 1000 1100 1200 1300 1400
Counts vs. Mass-to-Charge (m/z)

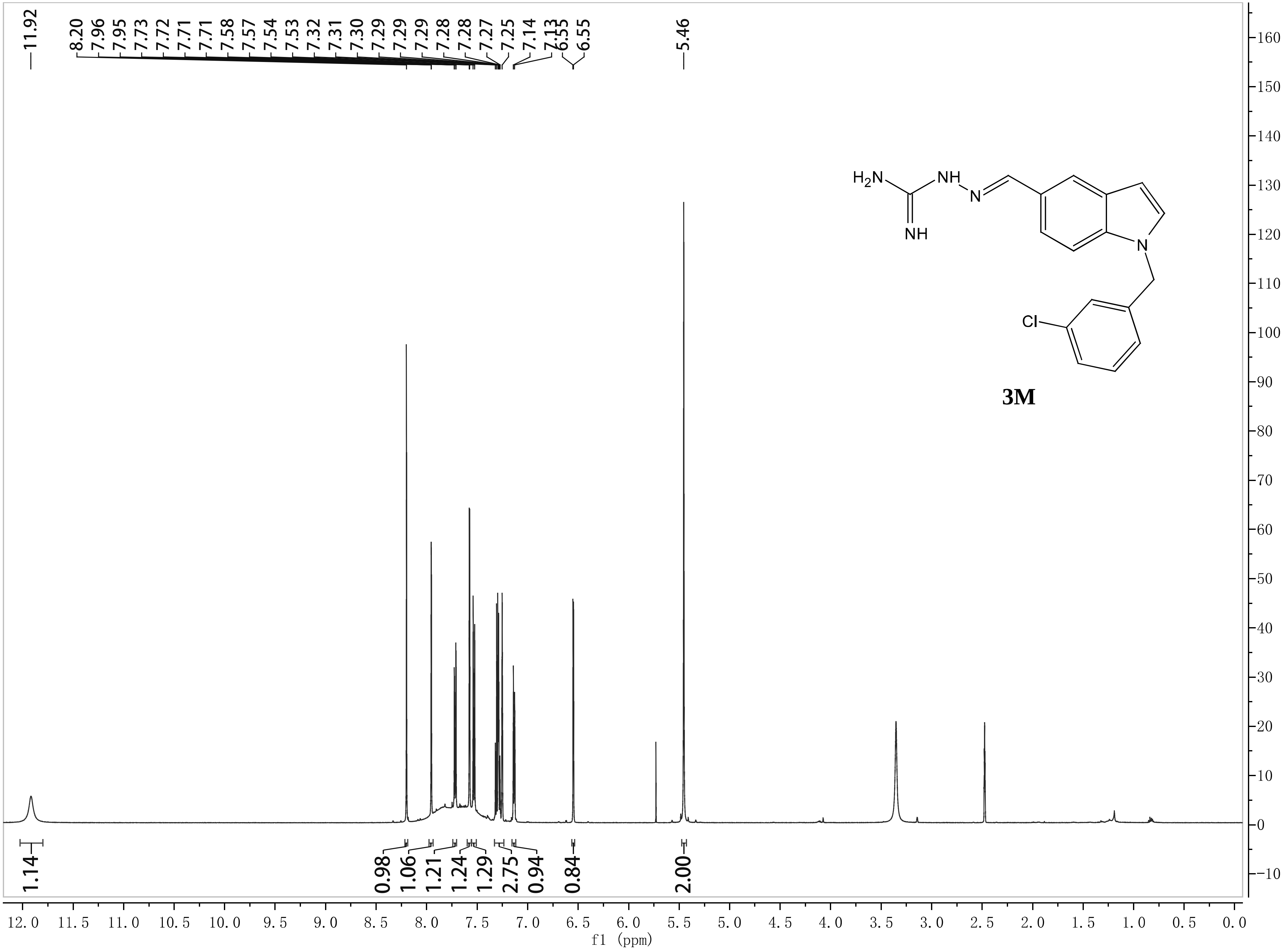

11.92
8.20
7.96
7.95
7.73
7.72
7.71
7.71
7.58
7.57
7.54
7.53
7.32
7.31
7.30
7.29
7.29
7.29
7.28
7.28
7.27
7.25
7.14
7.13
6.55
6.55
5.46
1.14
0.98
1.06
1.21
1.24
1.29
2.75
0.94
0.84
2.00
f1 (ppm)
H2N
NH
N
NH
N
Cl
3M

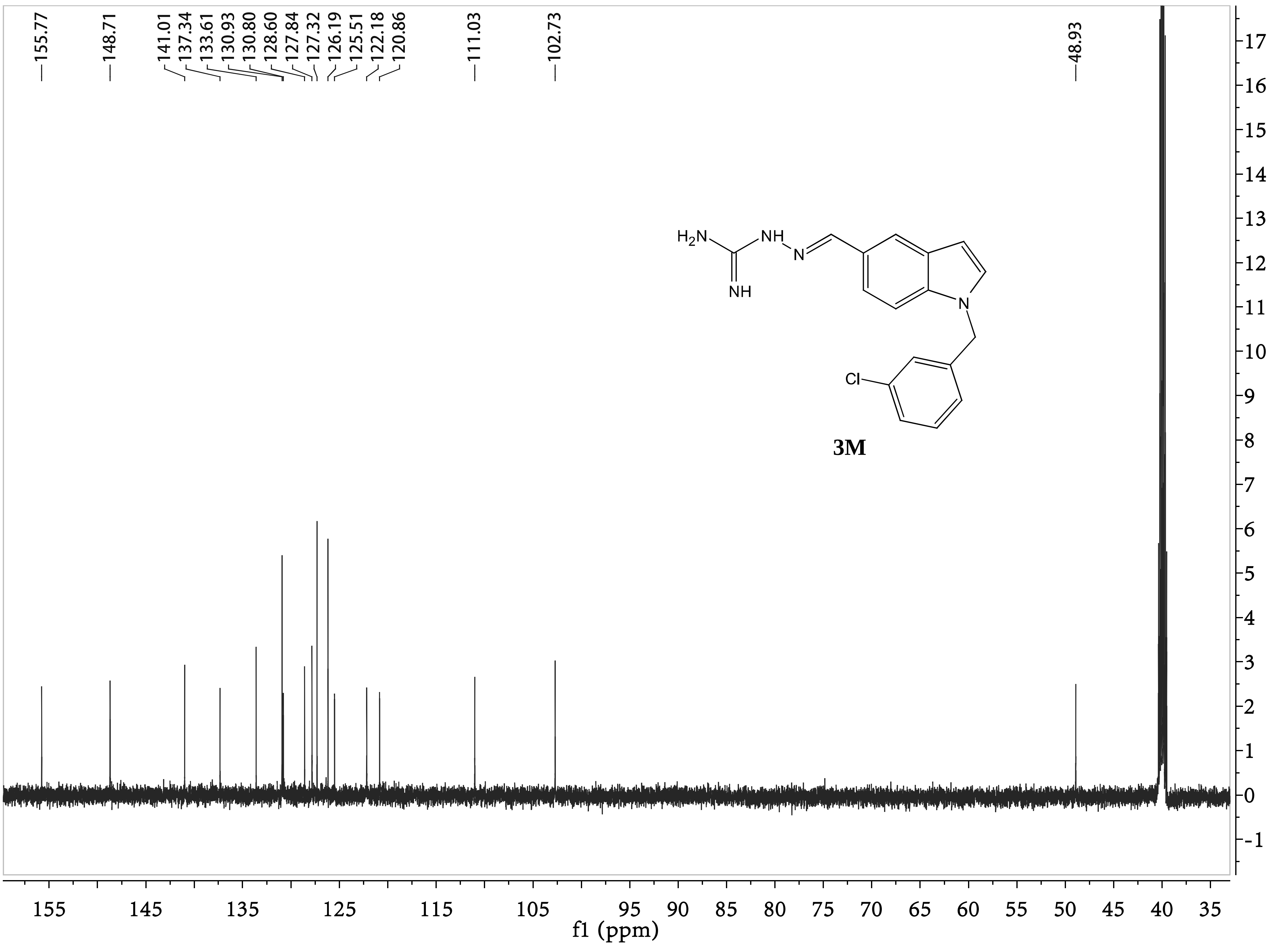

155.77
148.71
141.01
137.34
133.61
130.93
130.80
128.60
127.84
127.32
126.19
125.51
122.18
120.86
111.03
102.73
48.93
H_2N
NH
N
NH
N
Cl
f1 (ppm)

3M

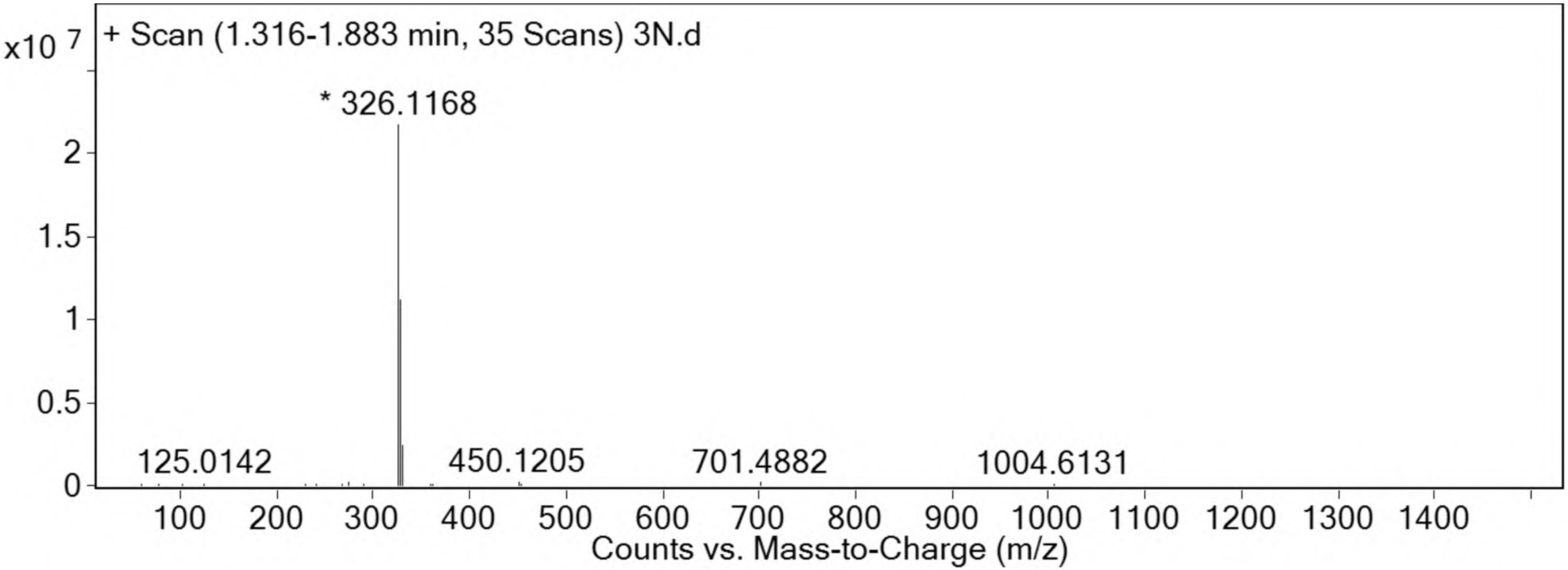

x10 7
+ Scan (1.316-1.883 min, 35 Scans) 3N.d
* 326.1168
2
1.5
1
0.5
0
125.0142
450.1205
701.4882
1004.6131
100
200
300
400
500
600
700
800
900
1000
1100
1200
1300
1400
Counts vs. Mass-to-Charge (m/z)

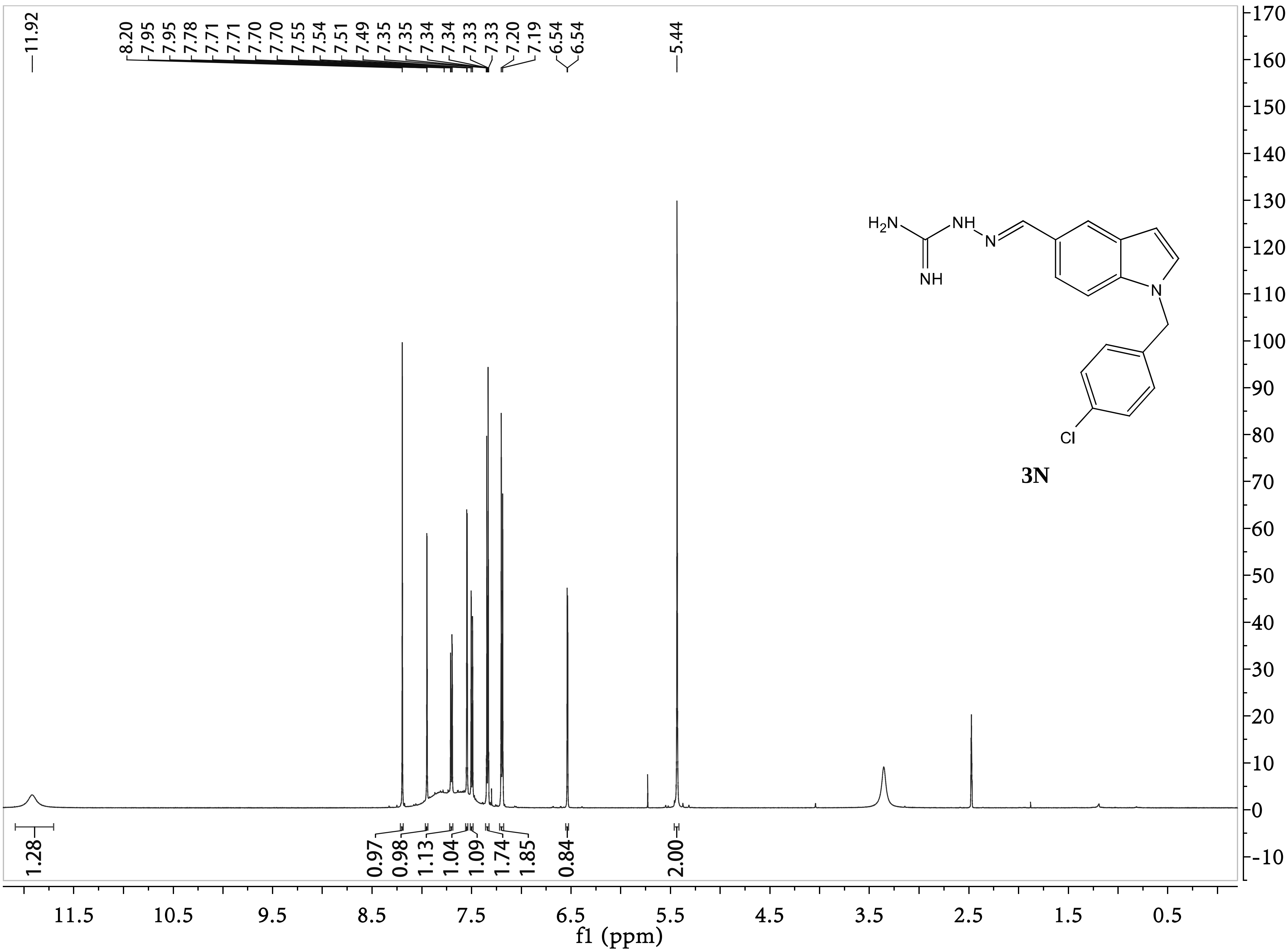

11.92
8.20
7.95
7.95
7.78
7.71
7.71
7.70
7.70
7.55
7.54
7.51
7.49
7.35
7.35
7.34
7.34
7.33
7.33
7.20
7.19
6.54
6.54
5.44
H_2N
NH
N
NH
N
Cl
3N
1.28
0.97
0.98
1.13
1.04
1.09
1.74
1.85
0.84
2.00
f1 (ppm)

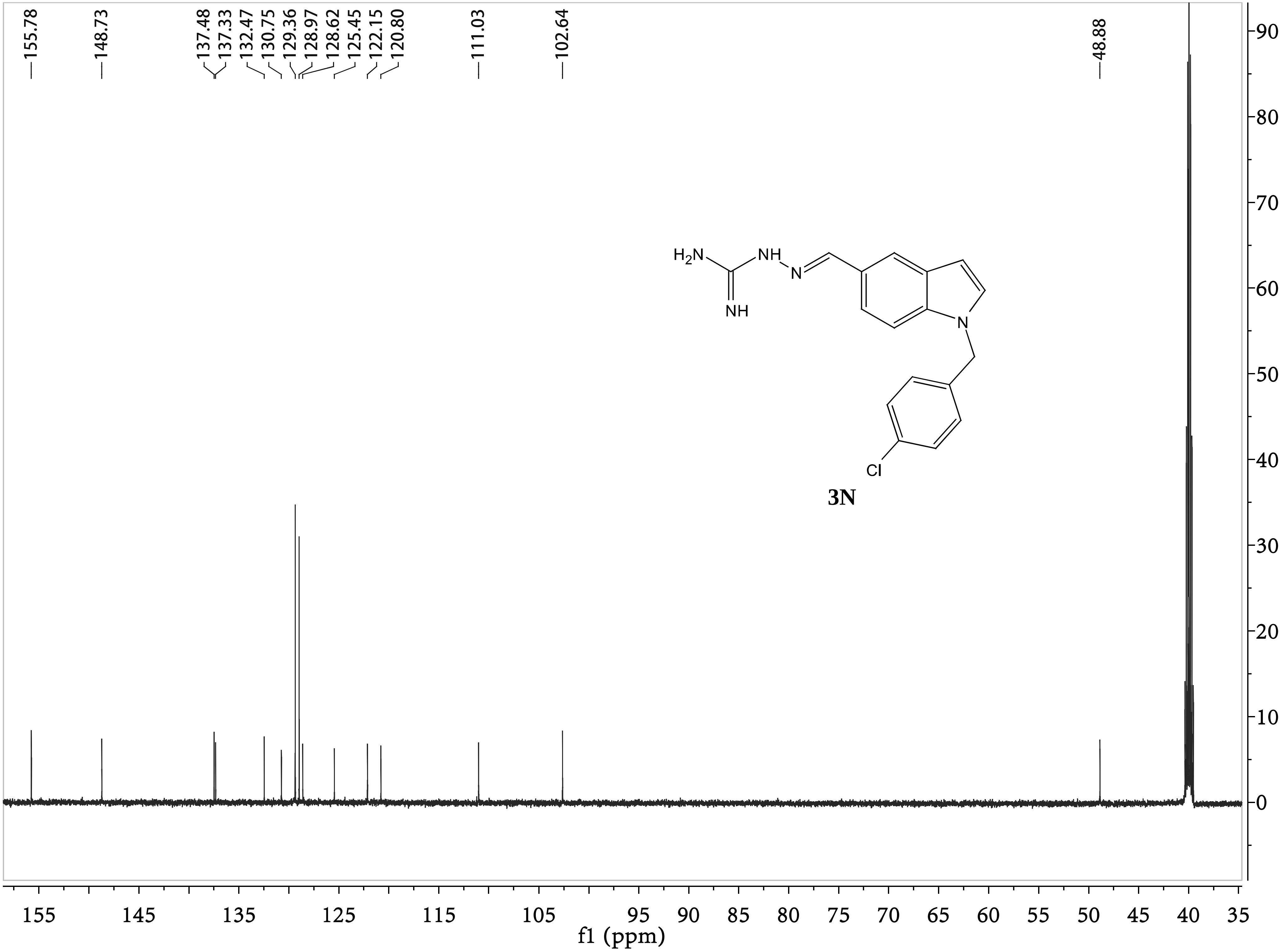
155.78
148.73
137.48
137.33
132.47
130.75
129.36
128.97
128.62
125.45
122.15
120.80
111.03
102.64
48.88
H2N
NH
N
NH
N
Cl
3N
f1 (ppm)

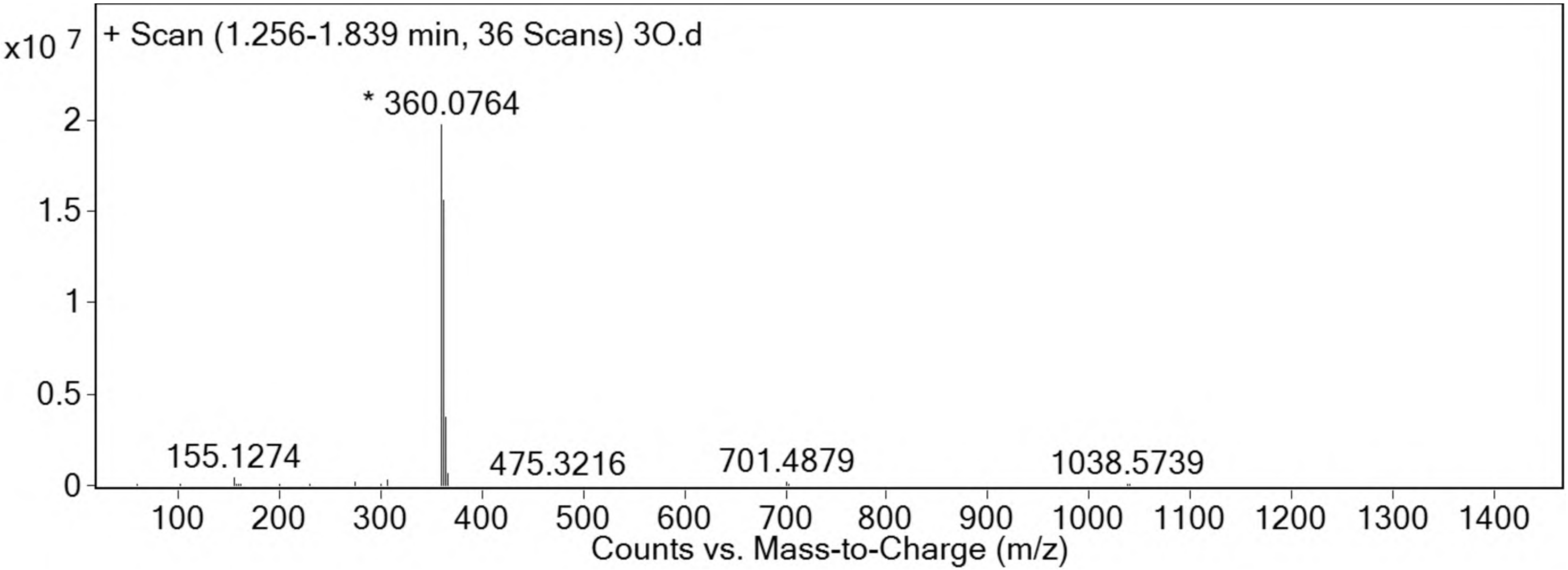
x10 7
+ Scan (1.256-1.839 min, 36 Scans) 3O.d
* 360.0764
155.1274
475.3216
701.4879
1038.5739
2
1.5
1
0.5
0
100 200 300 400 500 600 700 800 900 1000 1100 1200 1300 1400
Counts vs. Mass-to-Charge (m/z)

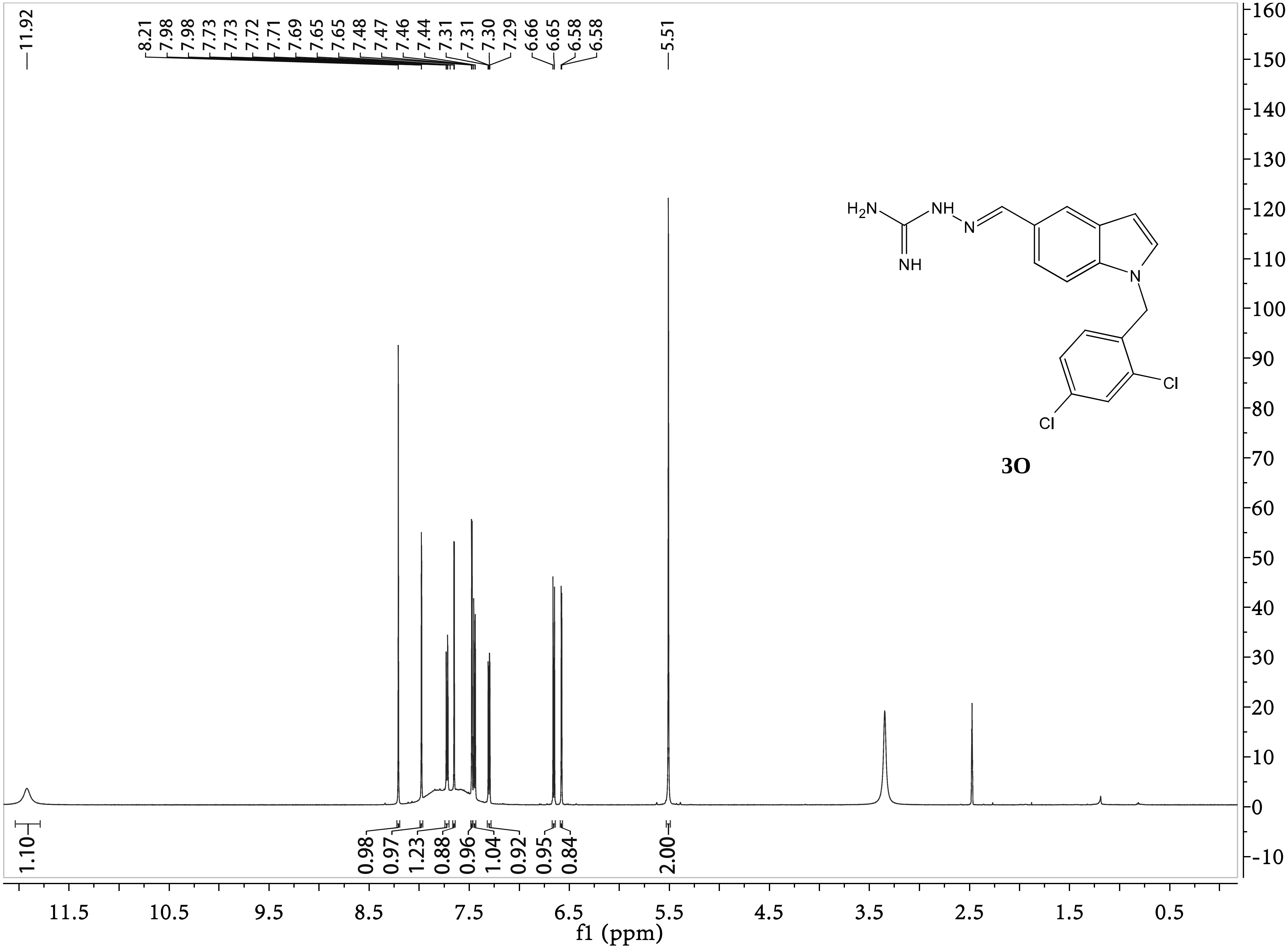

11.92
8.21 7.98 7.98 7.73 7.73 7.72 7.71 7.69 7.65 7.65 7.48 7.47 7.46 7.44 7.31 7.31 7.30 7.29 6.66 6.65 6.58 6.58
5.51
1.10
0.98 0.97 1.23 0.88 0.96 1.04 0.92 0.95 0.84
2.00
H_2N
NH
N
NH
N
Cl
Cl
3O
f1 (ppm)

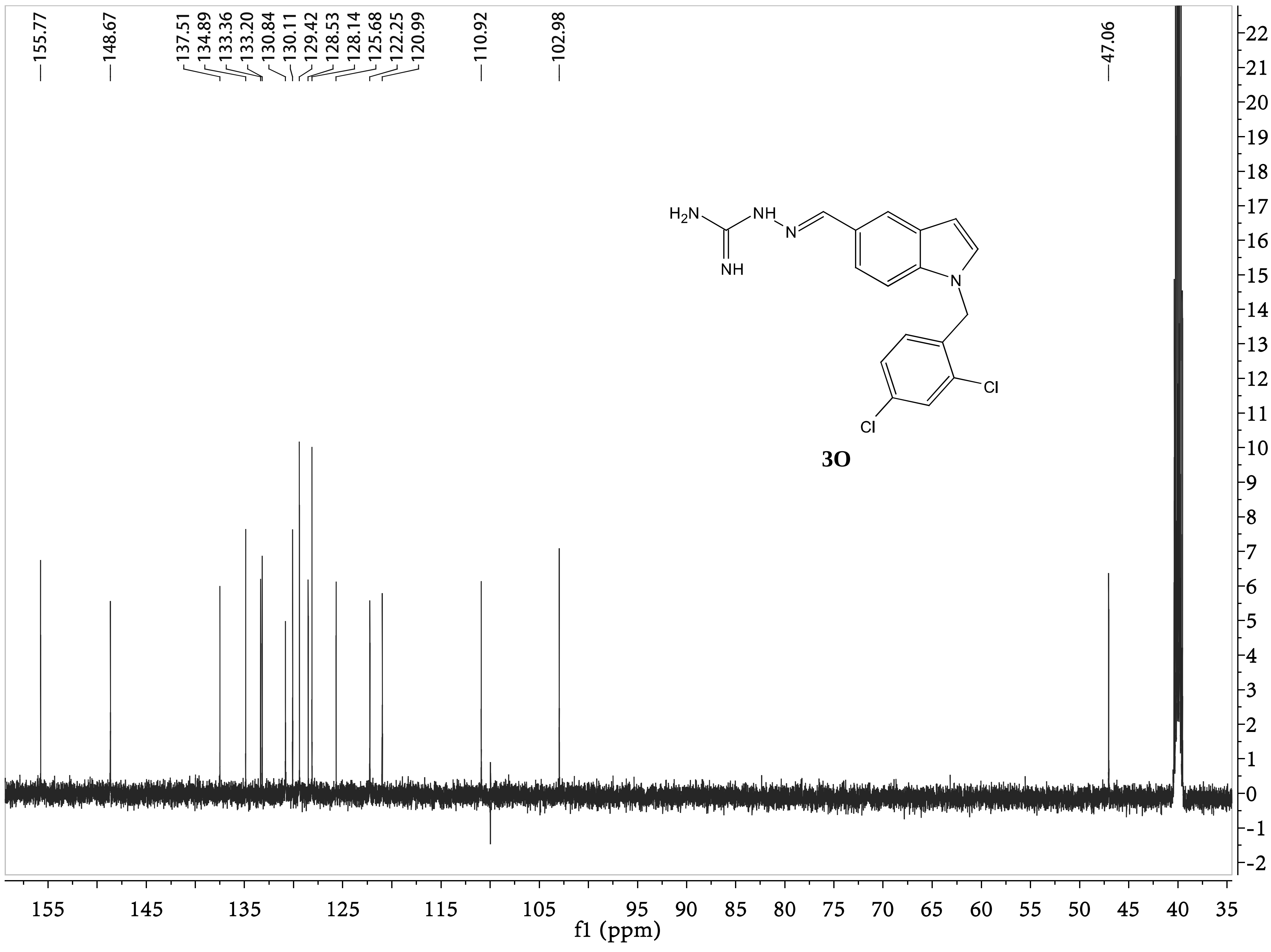
155.77
148.67
137.51
134.89
133.36
133.20
130.84
130.11
129.42
128.53
128.14
125.68
122.25
120.99
110.92
102.98
47.06
H2N
NH
N
NH
N
Cl
Cl
3O
f1 (ppm)

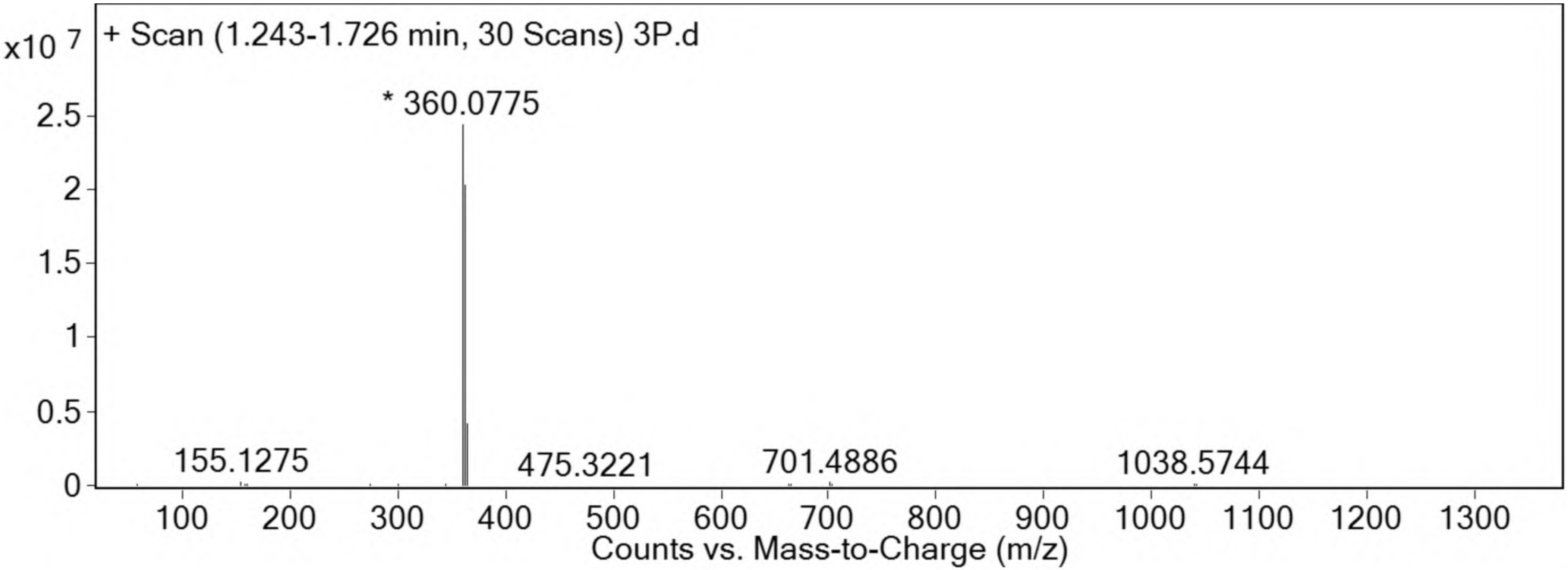
x10 7
+ Scan (1.243-1.726 min, 30 Scans) 3P.d
* 360.0775
155.1275
475.3221
701.4886
1038.5744
2.5
2
1.5
1
0.5
0
100 200 300 400 500 600 700 800 900 1000 1100 1200 1300
Counts vs. Mass-to-Charge (m/z)

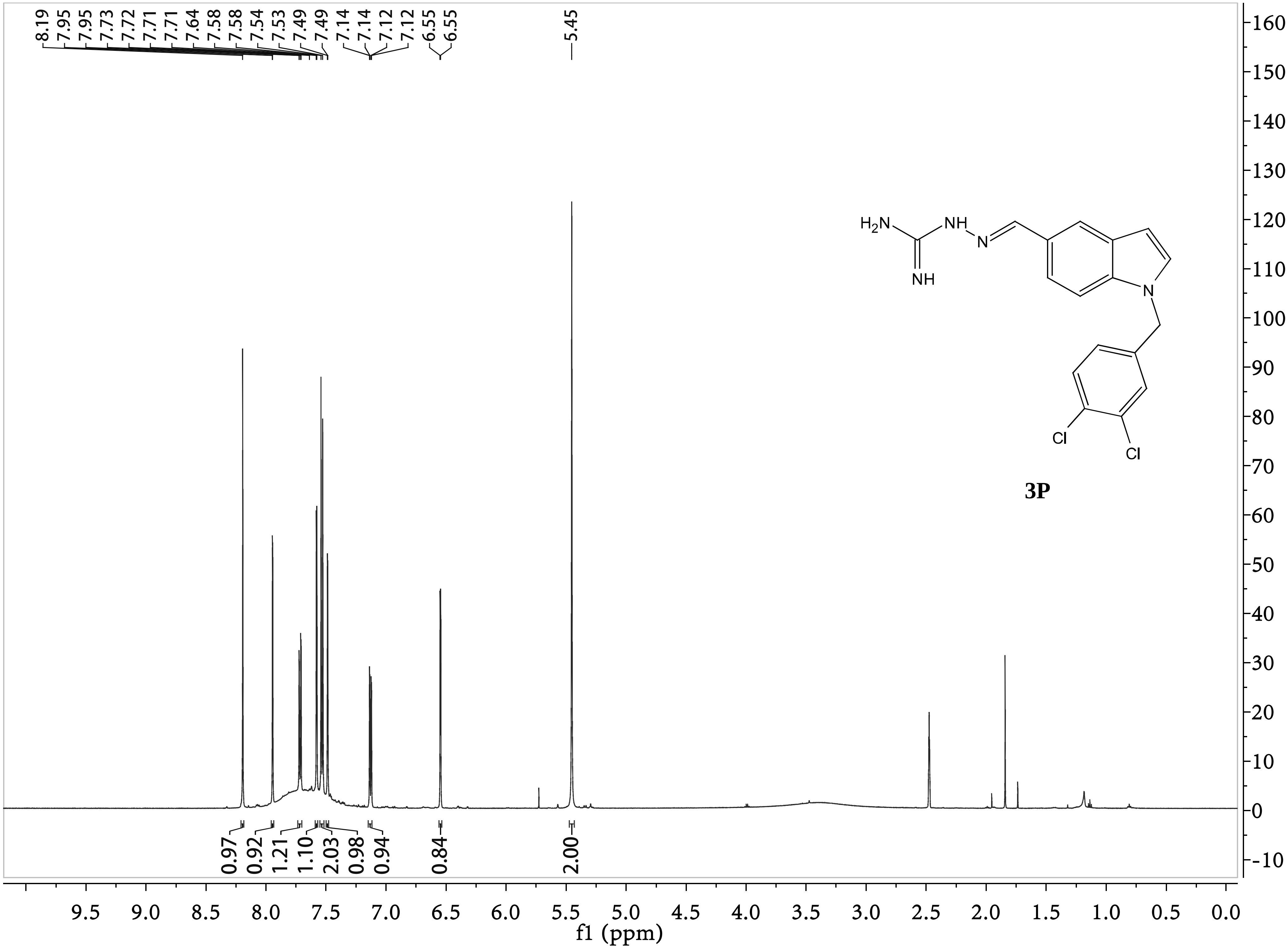
8.19 7.95 7.95 7.73 7.72 7.71 7.71 7.64 7.58 7.58 7.54 7.53 7.49 7.49 7.14 7.14 7.12 7.12 6.55 6.55
5.45
0.97 0.92 1.21 1.10 2.03 0.98 0.94 0.84
2.00
H_2N
NH
N
NH
N
Cl
Cl
3P
9.5 9.0 8.5 8.0 7.5 7.0 6.5 6.0 5.5 5.0 4.5 4.0 3.5 3.0 2.5 2.0 1.5 1.0 0.5 0.0
f1 (ppm)
160 150 140 130 120 110 100 90 80 70 60 50 40 30 20 10 0 -10

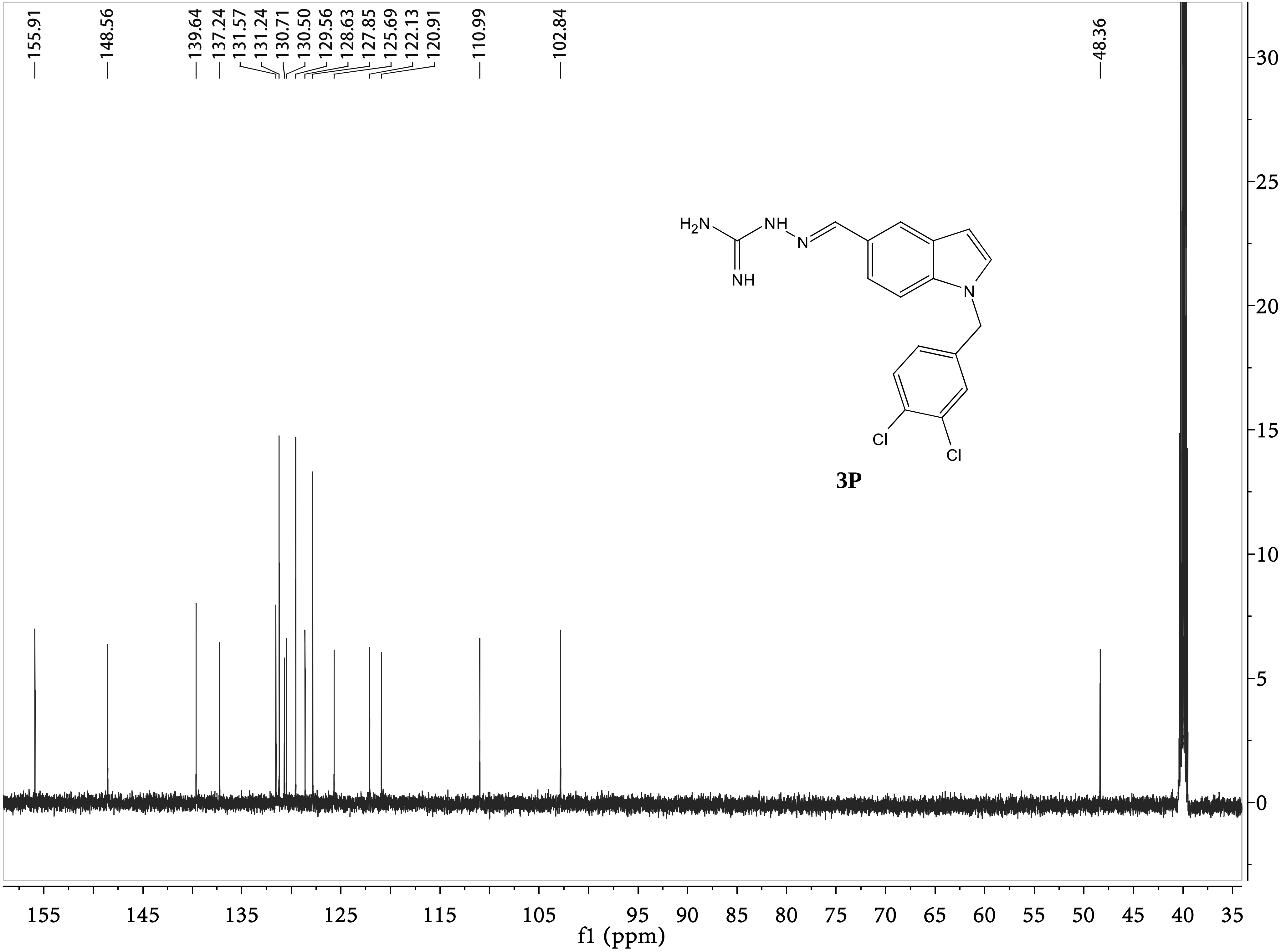
155.91
148.56
139.64
137.24
131.57
131.24
130.71
130.50
129.56
128.63
127.85
125.69
122.13
120.91
110.99
102.84
48.36
H2N
NH
N
NH
N
Cl
Cl
3P
155 145 135 125 115 105 95 90 85 80 75 70 65 60 55 50 45 40 35
f1 (ppm)
30 25 20 15 10 5 0

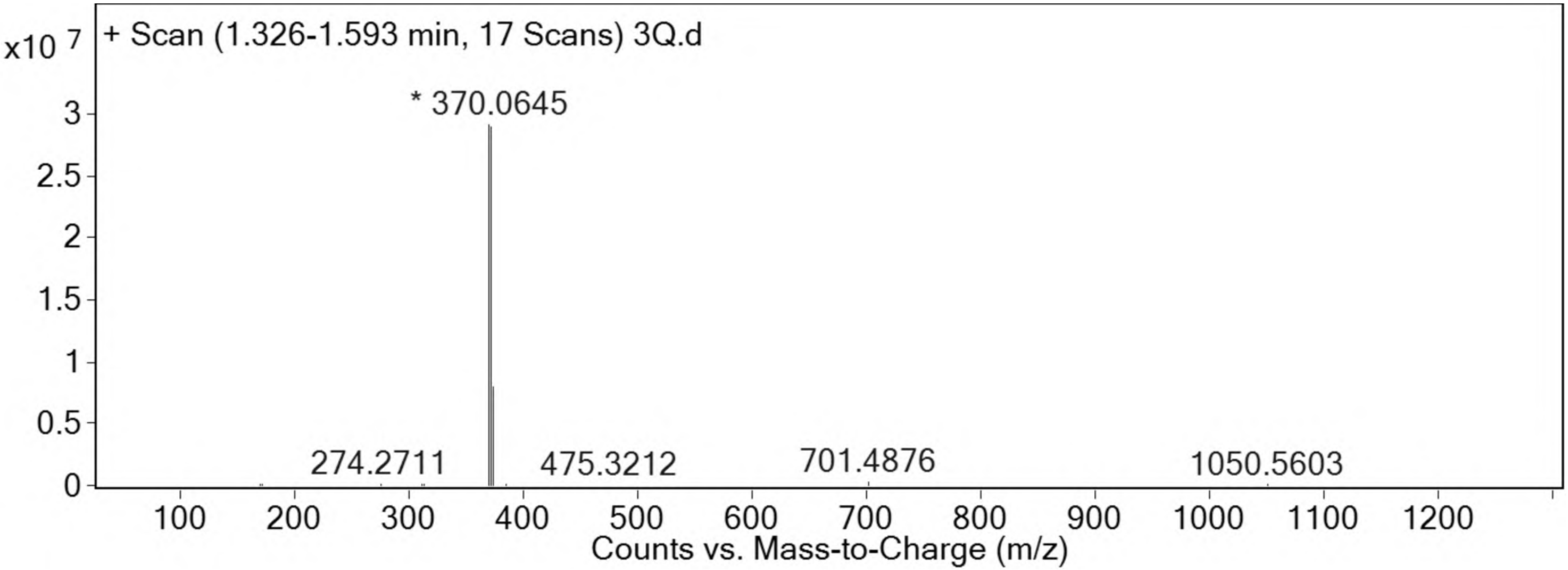

x10 7
+ Scan (1.326-1.593 min, 17 Scans) 3Q.d
* 370.0645
3
2.5
2
1.5
1
0.5
0
274.2711
475.3212
701.4876
1050.5603
100
200
300
400
500
600
700
800
900
1000
1100
1200
Counts vs. Mass-to-Charge (m/z)

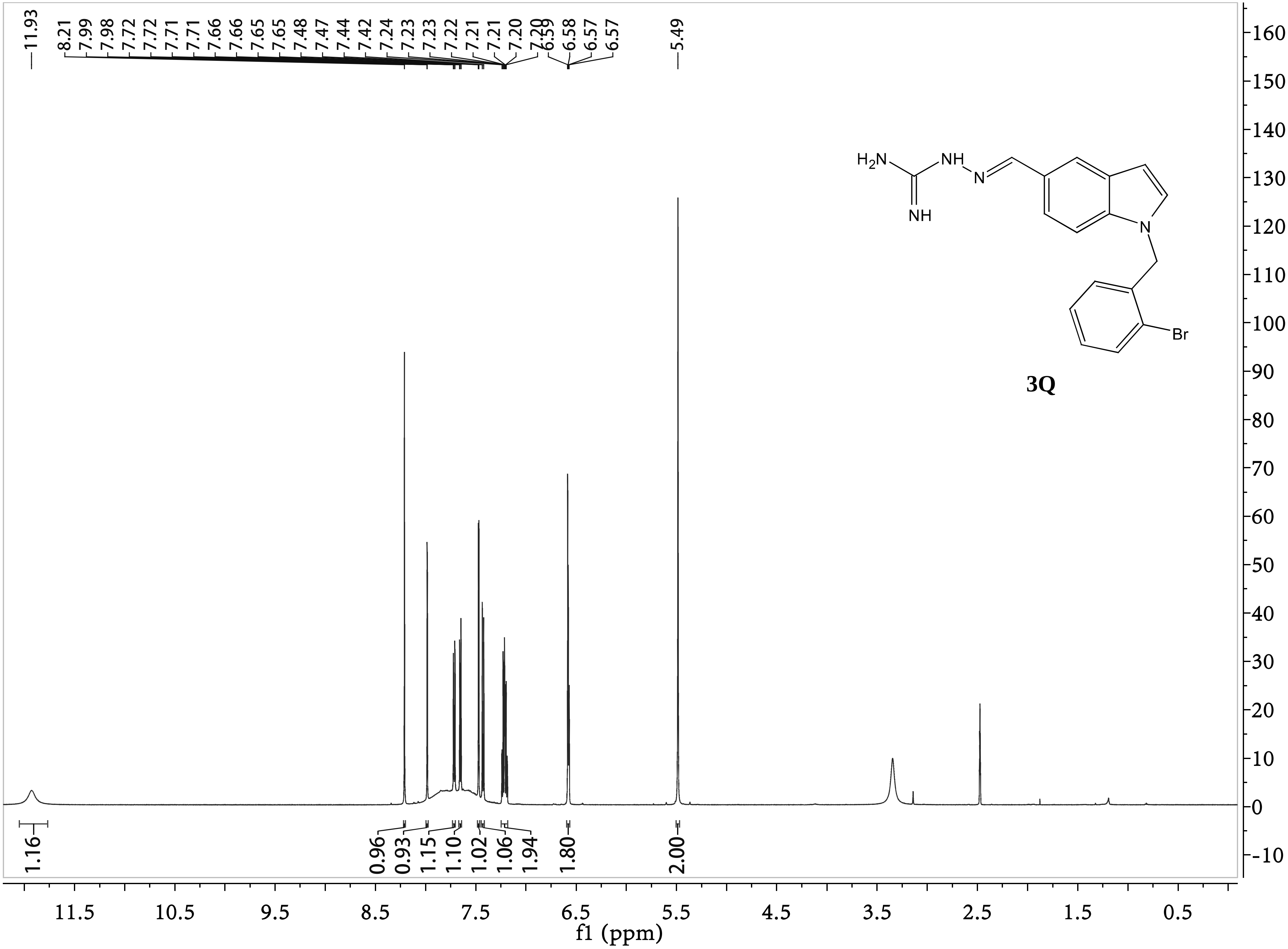

11.93
8.21
7.99
7.98
7.72
7.72
7.71
7.71
7.66
7.66
7.65
7.65
7.48
7.47
7.44
7.42
7.24
7.23
7.23
7.22
7.21
7.21
7.20
7.20
6.59
6.58
6.57
6.57
5.49
H_2N
NH
N
NH
N
Br
1.16
0.96
0.93
1.15
1.10
1.02
1.06
1.94
1.80
2.00
11.5
10.5
9.5
8.5
7.5
6.5
5.5
4.5
3.5
2.5
1.5
0.5
f1 (ppm)
160
150
140
130
120
110
100
90
80
70
60
50
40
30
20
10
0
-10

3Q

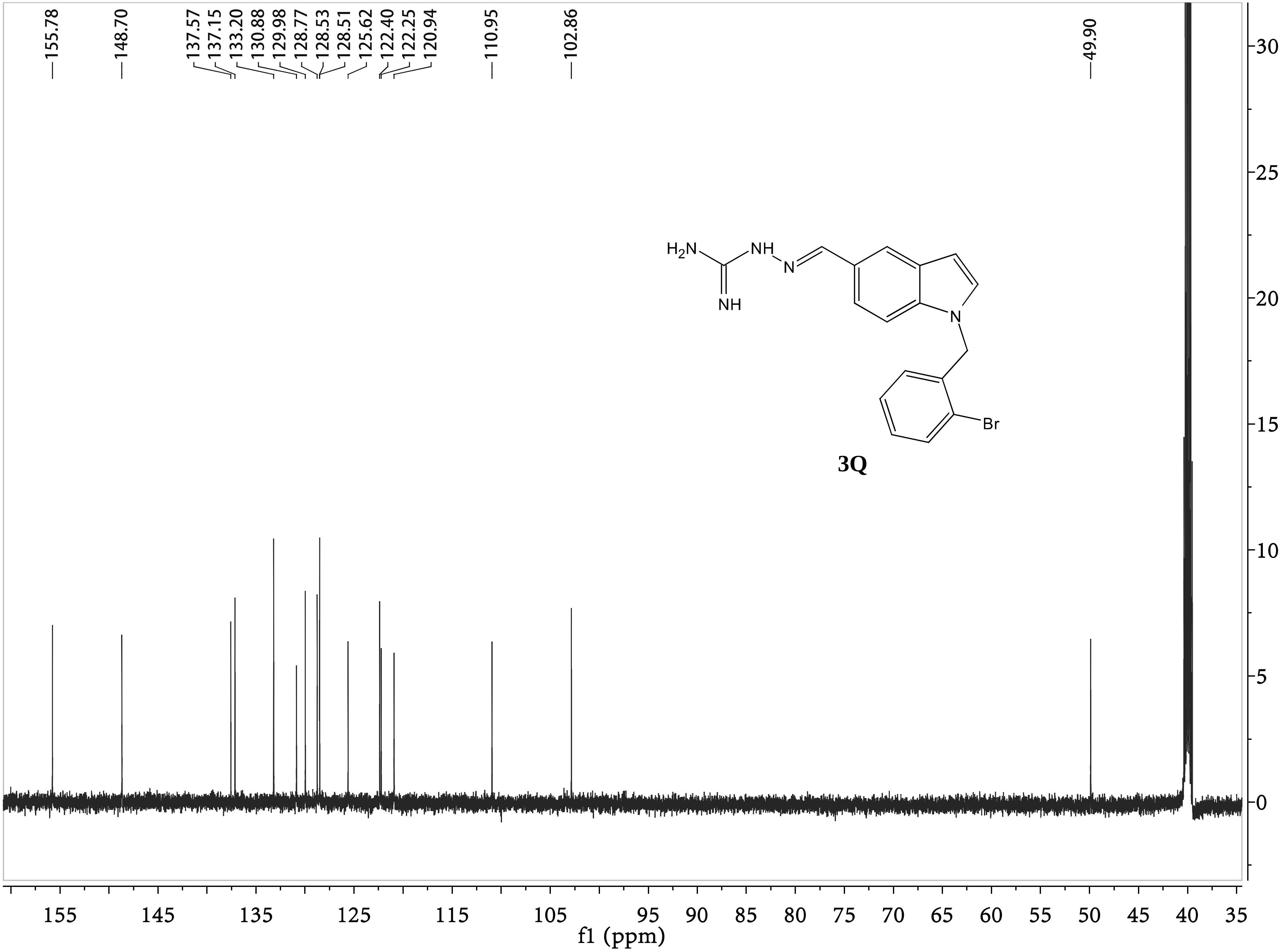

155.78
148.70
137.57
137.15
133.20
130.88
129.98
128.77
128.53
128.51
125.62
122.40
122.25
120.94
110.95
102.86
49.90
H_2N
NH
N
NH
N
Br
3Q
f1 (ppm)

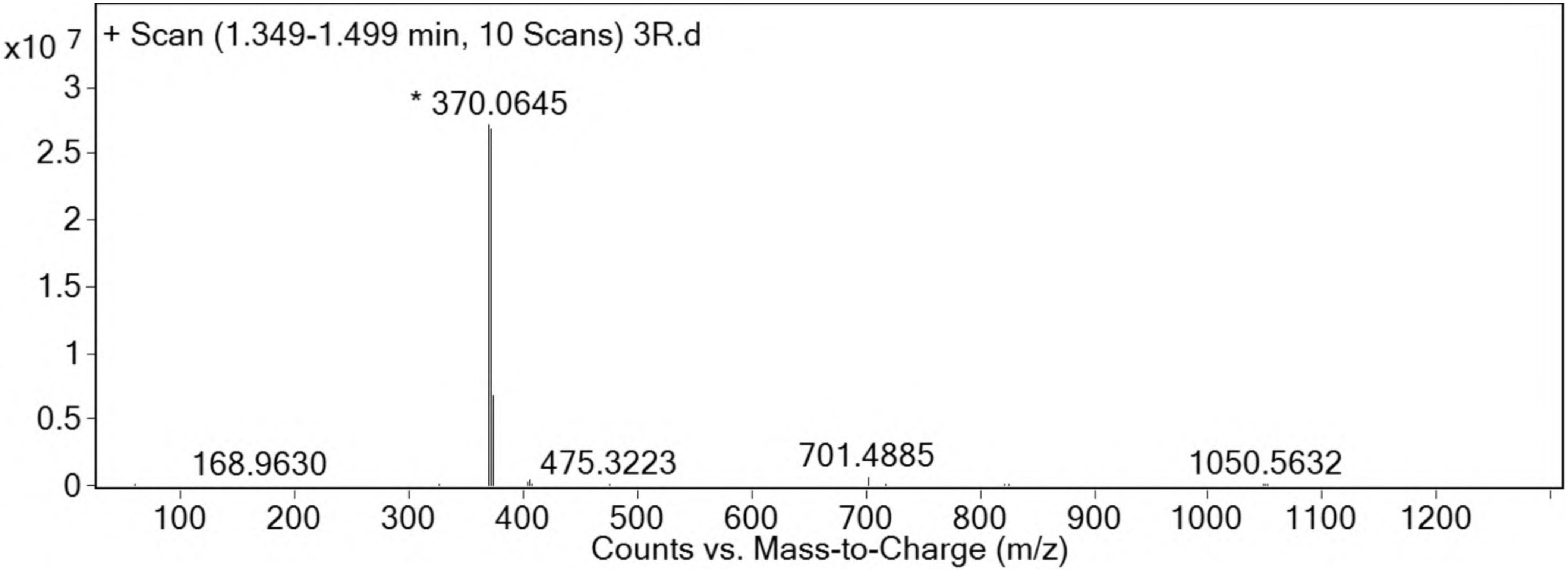

x10 7
+ Scan (1.349-1.499 min, 10 Scans) 3R.d
* 370.0645
168.9630
475.3223
701.4885
1050.5632
3
2.5
2
1.5
1
0.5
0
100
200
300
400
500
600
700
800
900
1000
1100
1200
Counts vs. Mass-to-Charge (m/z)

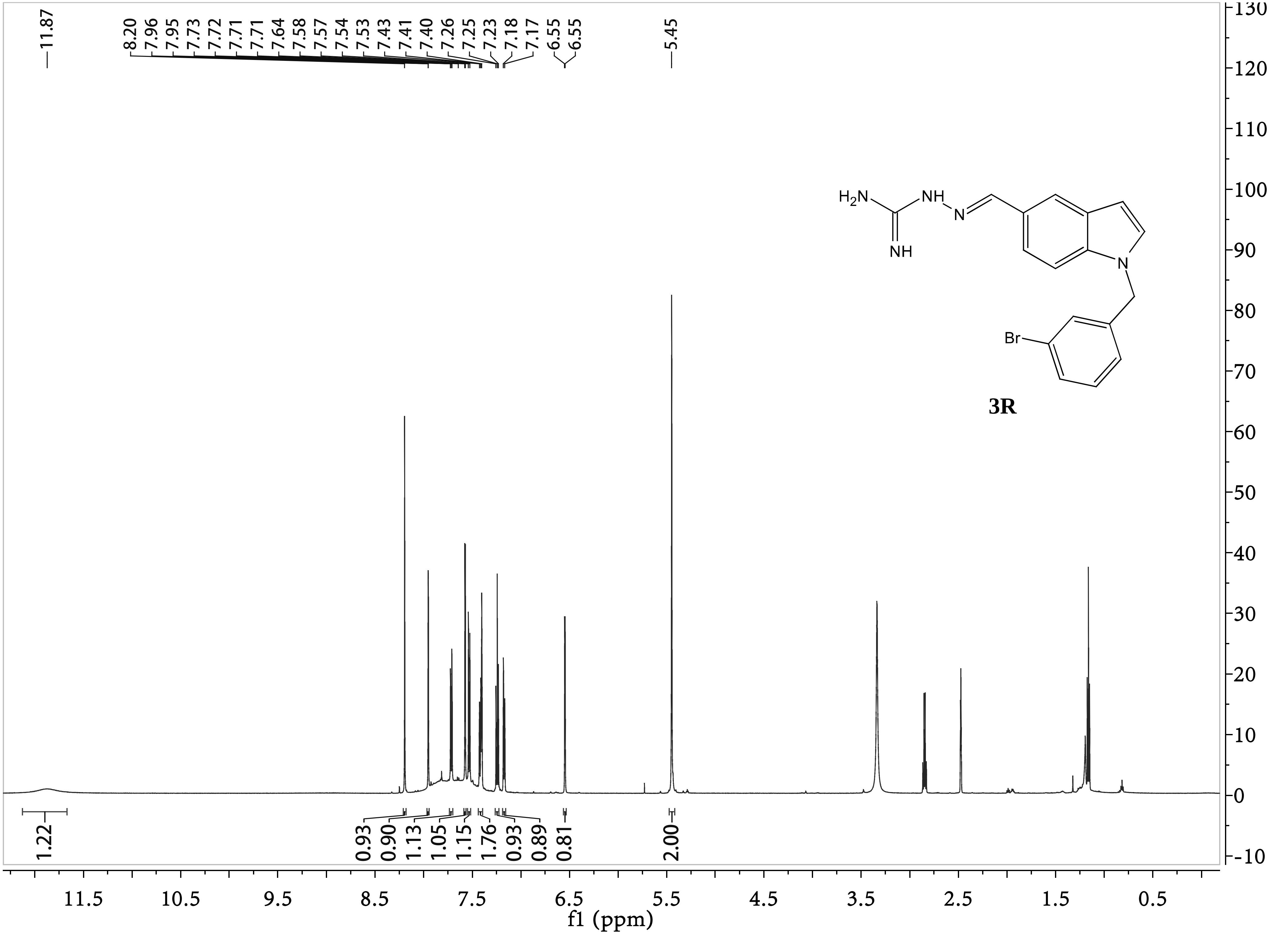
11.87
8.20
7.96
7.95
7.73
7.72
7.71
7.71
7.64
7.58
7.57
7.54
7.53
7.43
7.41
7.40
7.26
7.25
7.23
7.18
7.17
6.55
6.55
5.45
H_2N
NH
N
NH
N
Br
3R
1.22
0.93
0.90
1.13
1.05
1.15
1.76
0.93
0.89
0.81
2.00
f1 (ppm)

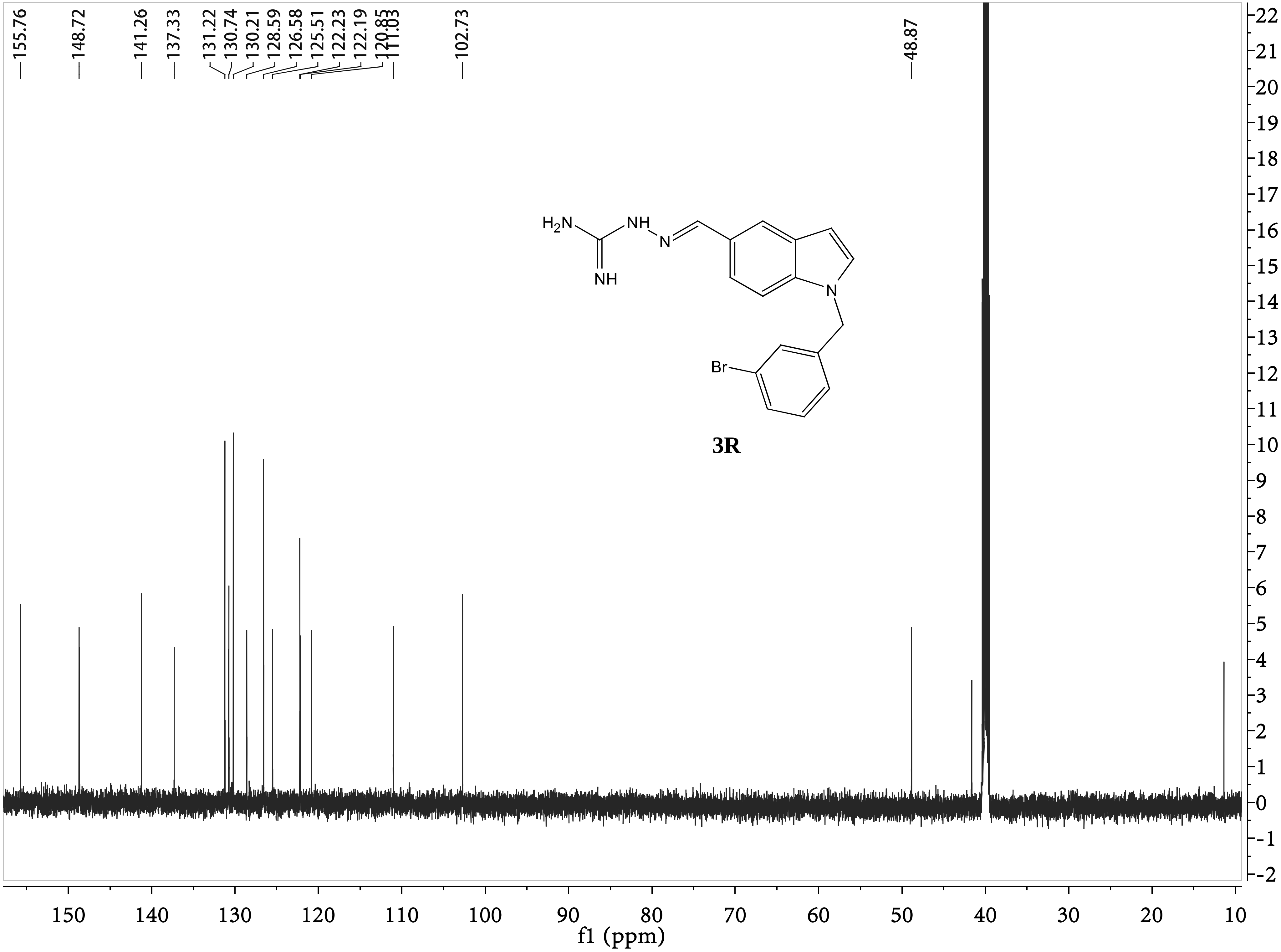

155.76
148.72
141.26
137.33
131.22
130.74
130.21
128.59
126.58
125.51
122.23
122.19
120.85
111.03
102.73
48.87
H2N
NH
N
NH
N
Br
3R
150 140 130 120 110 100 90 80 70 60 50 40 30 20 10
f1 (ppm)
22 21 20 19 18 17 16 15 14 13 12 11 10 9 8 7 6 5 4 3 2 1 0 -1 -2

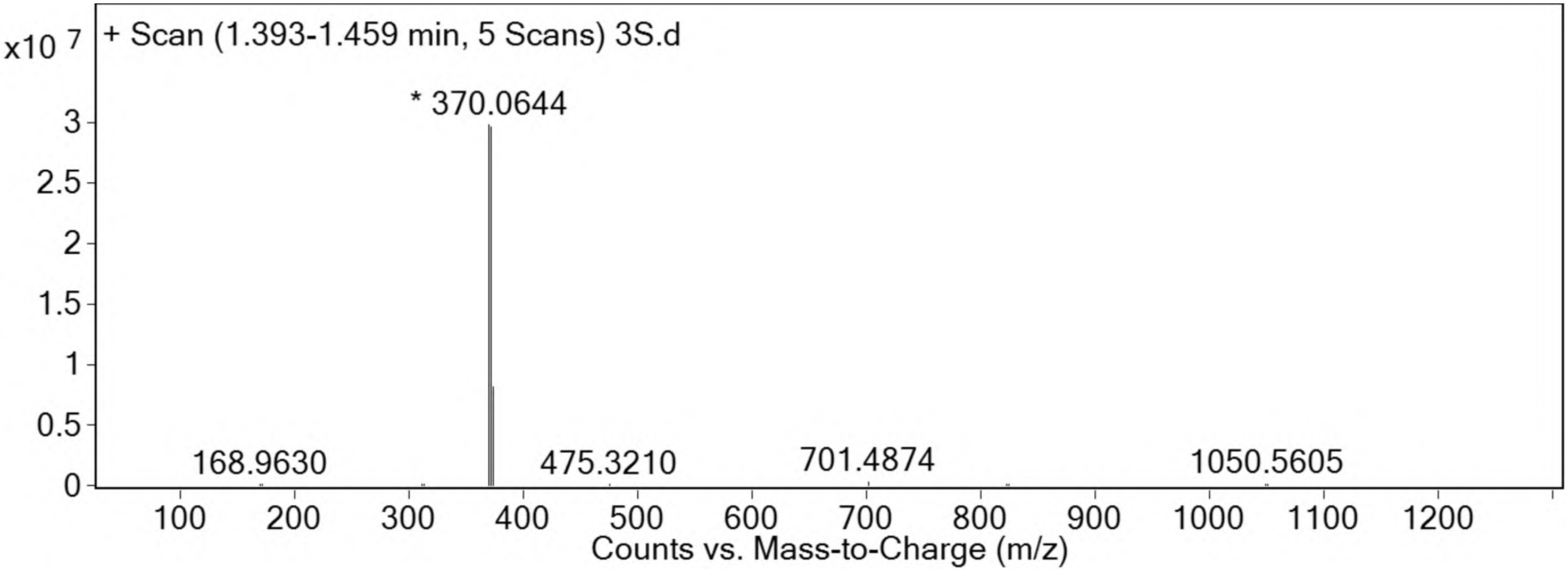

x10 7
+ Scan (1.393-1.459 min, 5 Scans) 3S.d
* 370.0644
3
2.5
2
1.5
1
0.5
0
168.9630
475.3210
701.4874
1050.5605
100
200
300
400
500
600
700
800
900
1000
1100
1200
Counts vs. Mass-to-Charge (m/z)

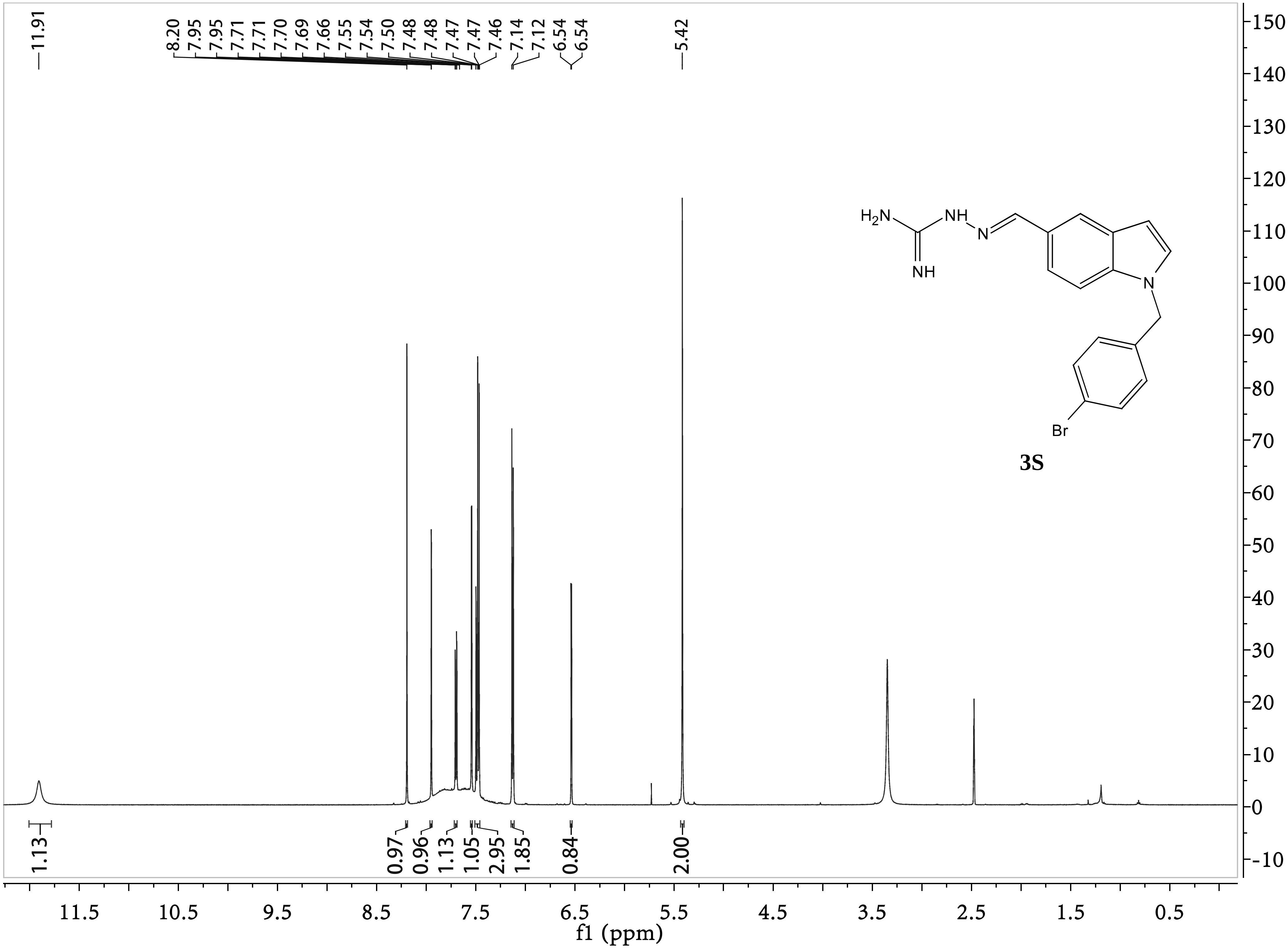
11.91
8.20 7.95 7.95 7.71 7.71 7.70 7.69 7.66 7.55 7.54 7.50 7.48 7.48 7.47 7.47 7.46 7.14 7.12 6.54 6.54
5.42
1.13 0.97 0.96 1.13 1.05 2.95 1.85 0.84 2.00
f1 (ppm)
H_2N
NH
N
NH
N
Br
3S

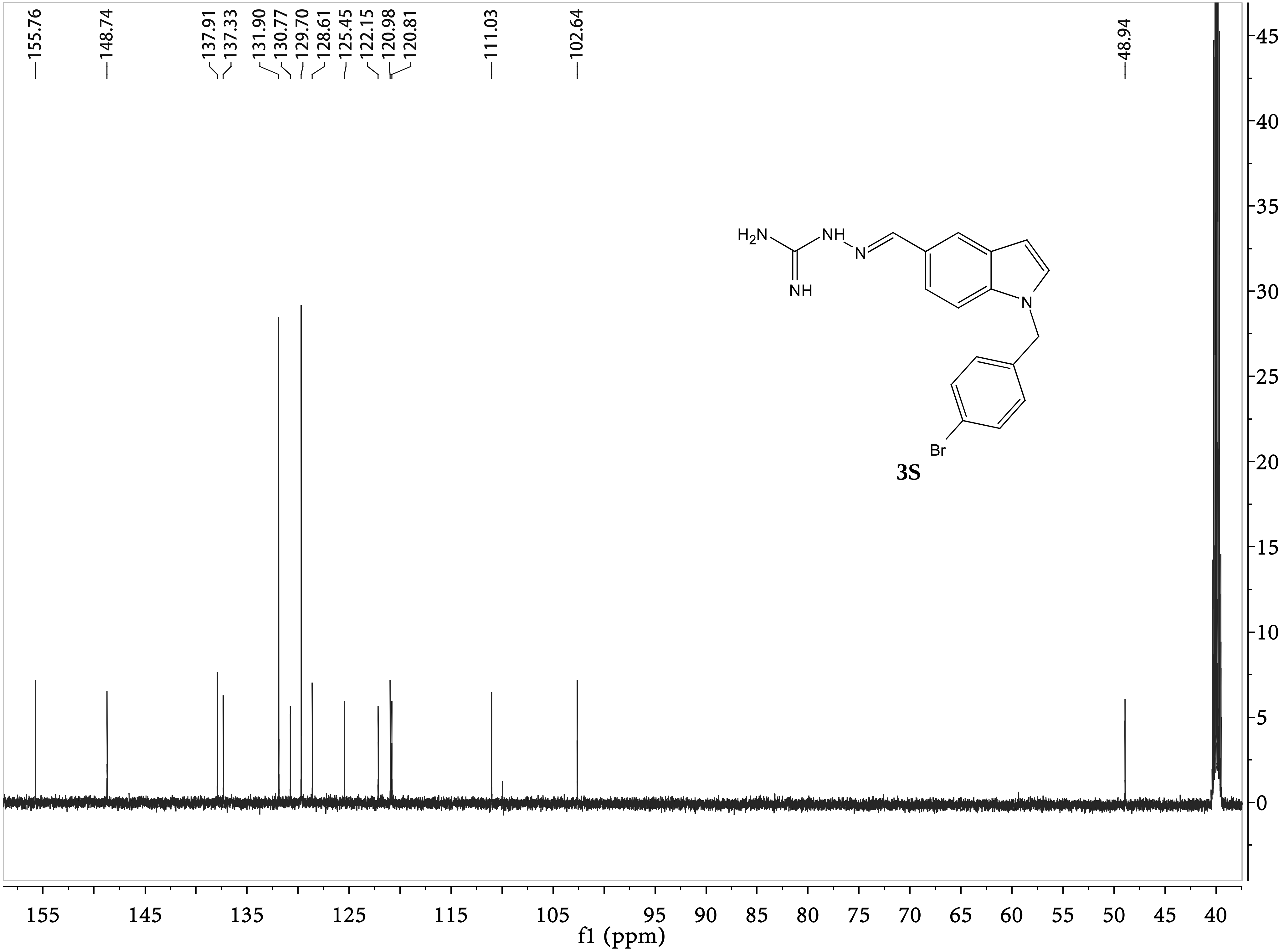
155.76
148.74
137.91
137.33
131.90
130.77
129.70
128.61
125.45
122.15
120.98
120.81
111.03
102.64
48.94
H2N
NH
N
NH
N
Br
3S
f1 (ppm)

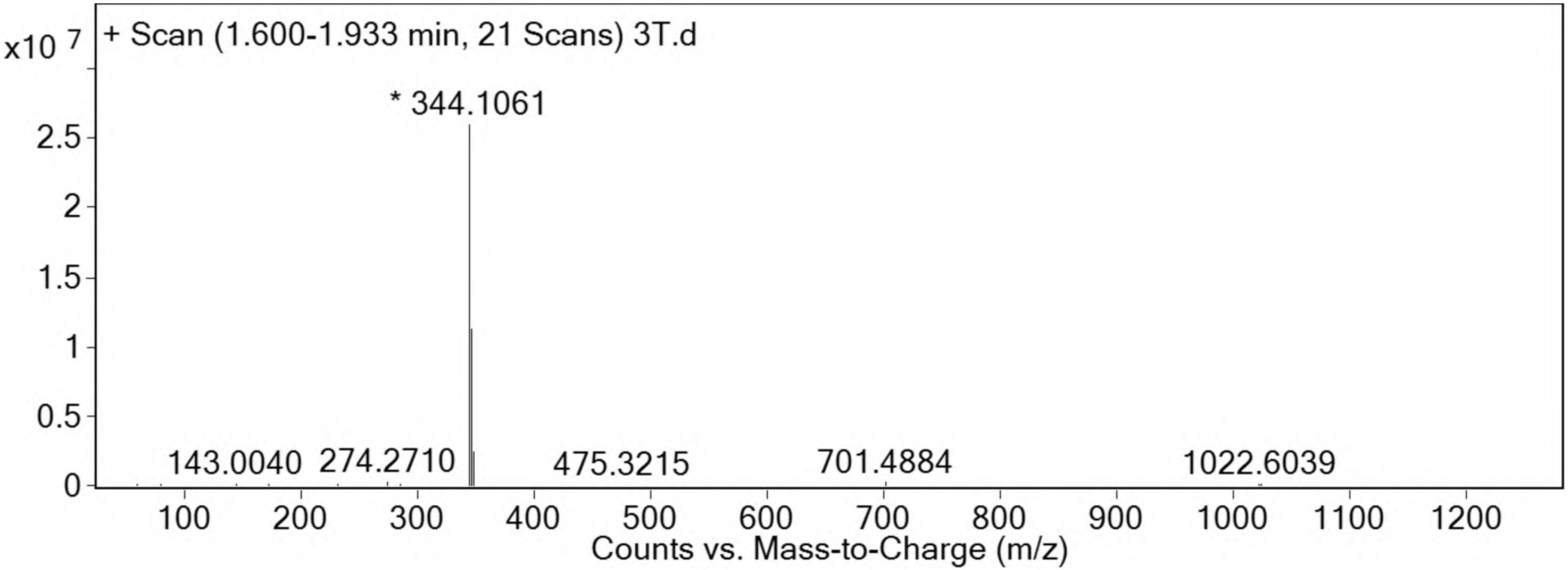

x10 7
+ Scan (1.600-1.933 min, 21 Scans) 3T.d
* 344.1061
2.5
2
1.5
1
0.5
0
143.0040
274.2710
475.3215
701.4884
1022.6039
100
200
300
400
500
600
700
800
900
1000
1100
1200
Counts vs. Mass-to-Charge (m/z)

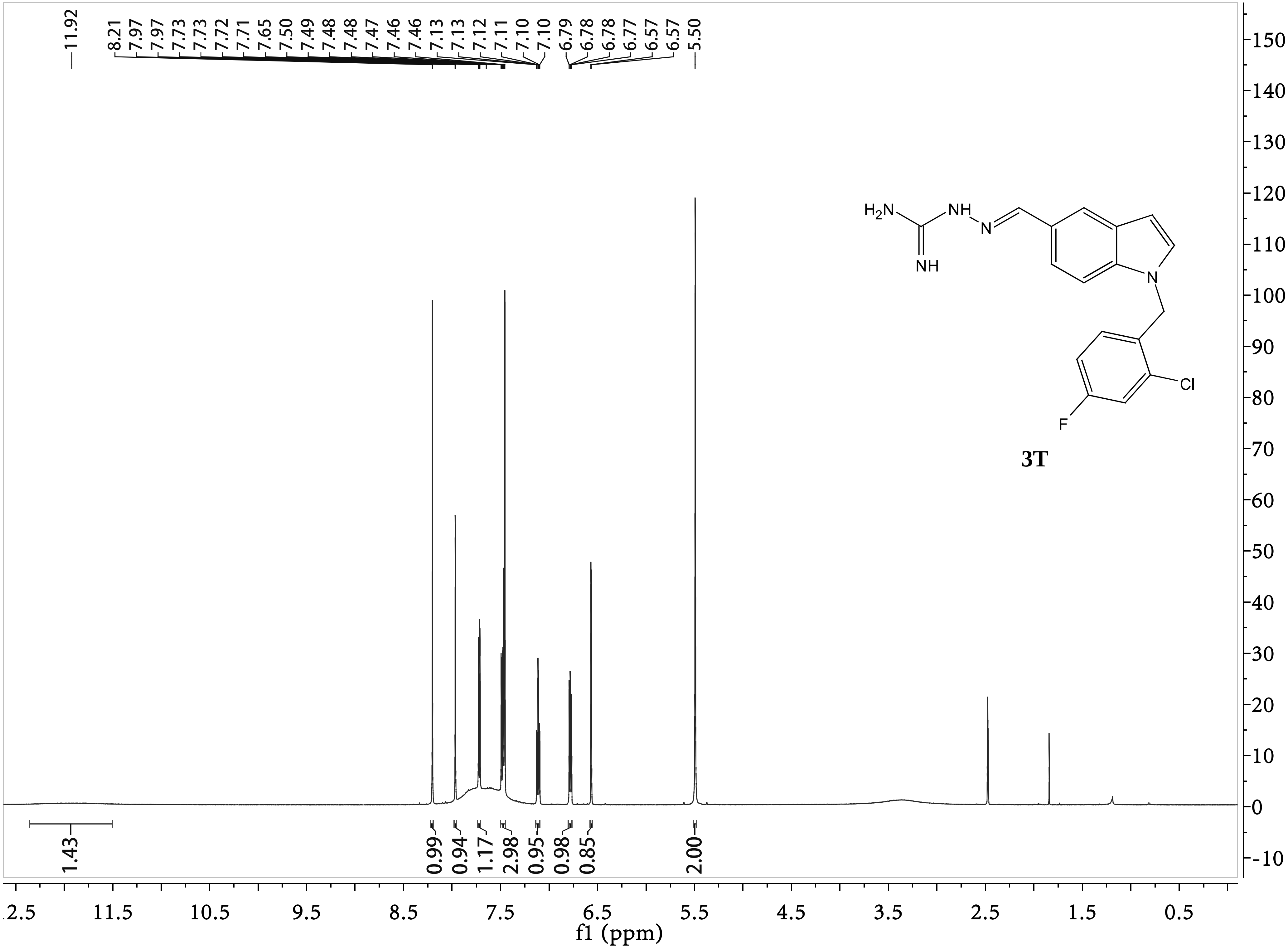

11.92
8.21
7.97
7.97
7.73
7.73
7.72
7.71
7.65
7.50
7.49
7.48
7.48
7.47
7.46
7.46
7.13
7.13
7.12
7.11
7.10
7.10
6.79
6.78
6.78
6.77
6.57
6.57
5.50
H2N
NH
N
NH
N
Cl
F
1.43
0.99
0.94
1.17
2.98
0.95
0.98
0.85
2.00
f1 (ppm)

3T

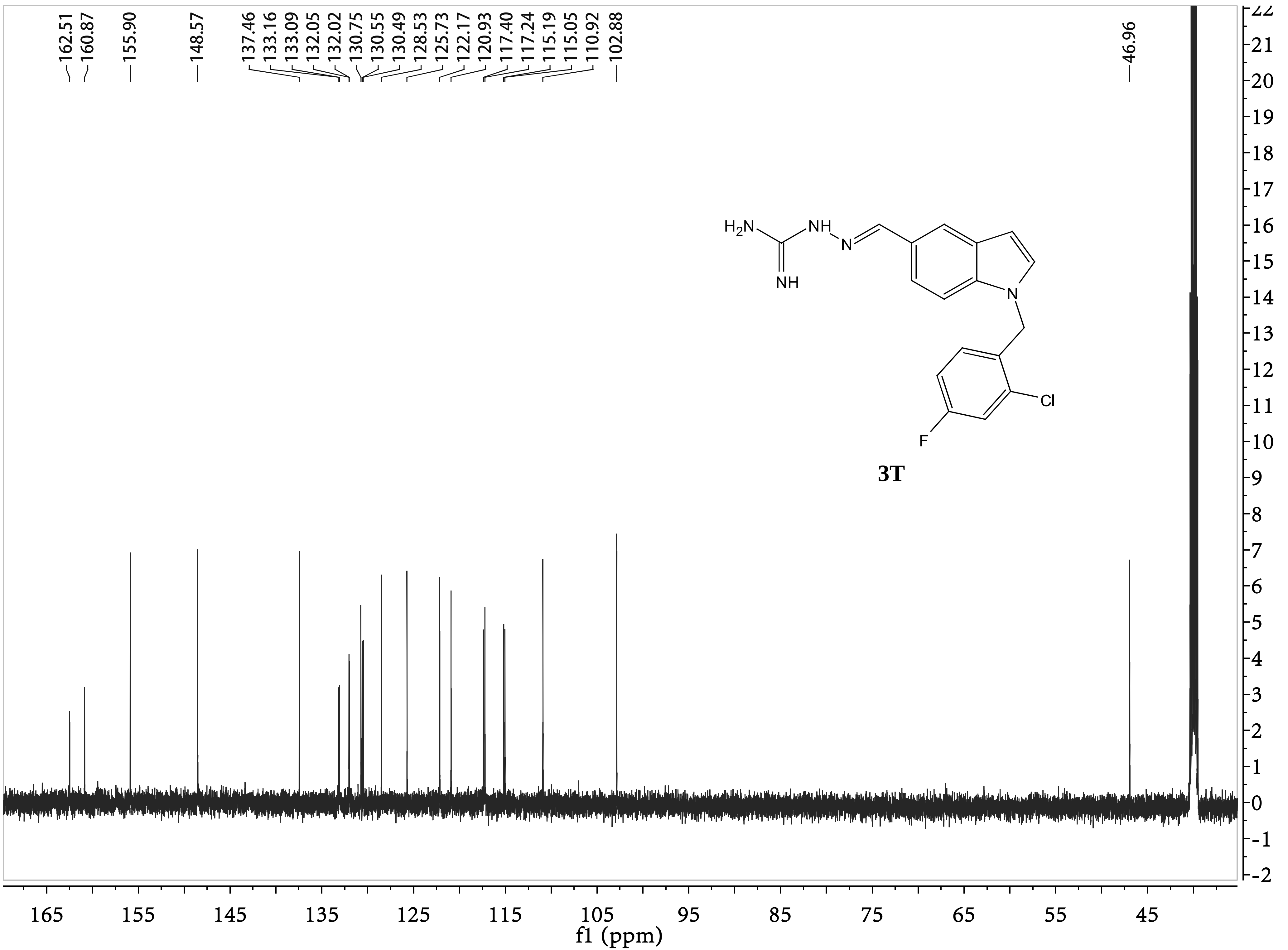
162.51
160.87
155.90
148.57
137.46
133.16
133.09
132.05
132.02
130.75
130.55
130.49
128.53
125.73
122.17
120.93
117.40
117.24
115.19
115.05
110.92
102.88
46.96
H2N
NH
N
NH
N
Cl
F
3T
f1 (ppm)

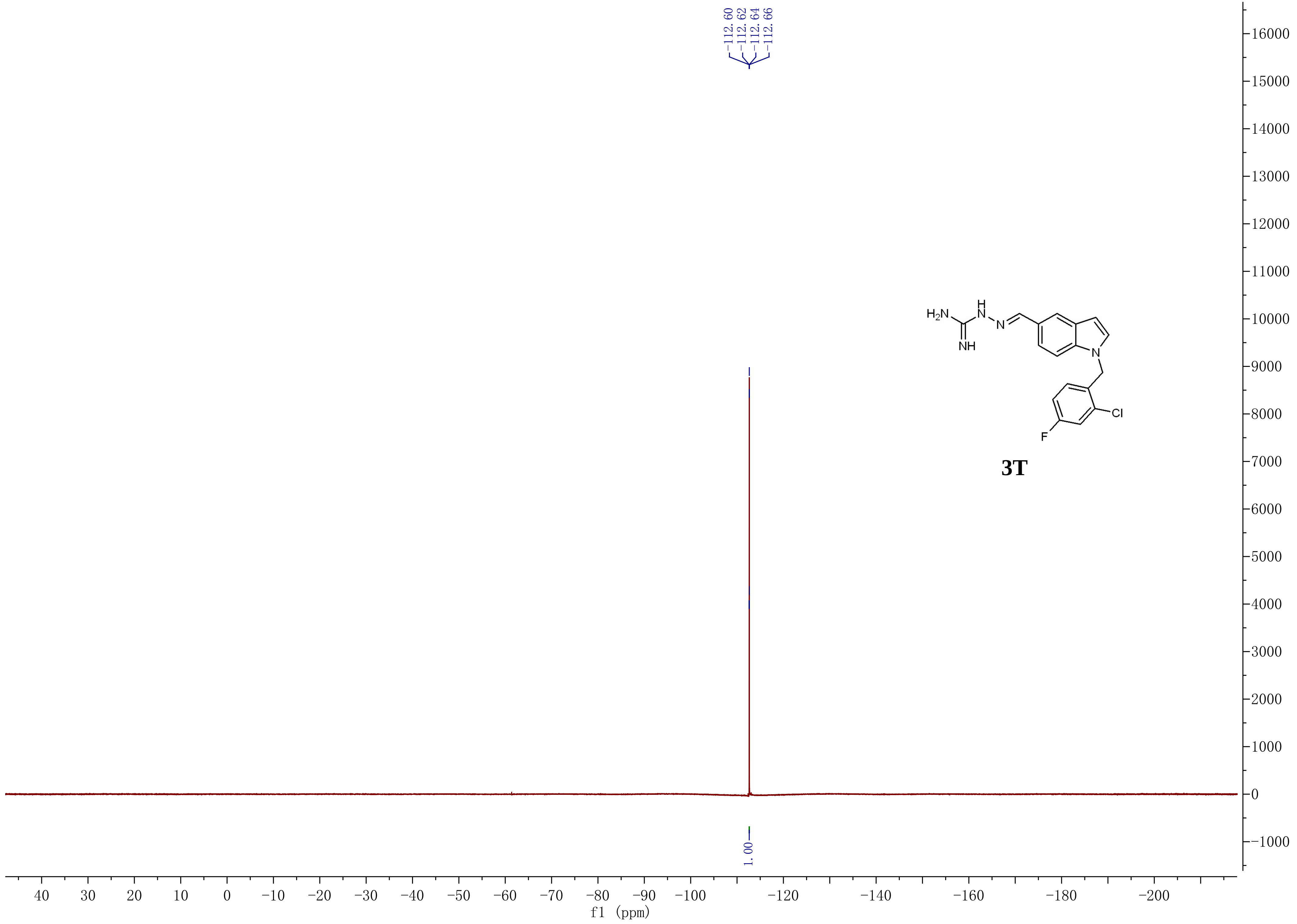

−112.60
−112.62
−112.64
−112.66
H2N
H
N
N
NH
N
Cl
F
3T
1.00
40 30 20 10 0 −10 −20 −30 −40 −50 −60 −70 −80 −90 −100 −120 −140 −160 −180 −200
f1 (ppm)
16000 15000 14000 13000 12000 11000 10000 9000 8000 7000 6000 5000 4000 3000 2000 1000 0 −1000

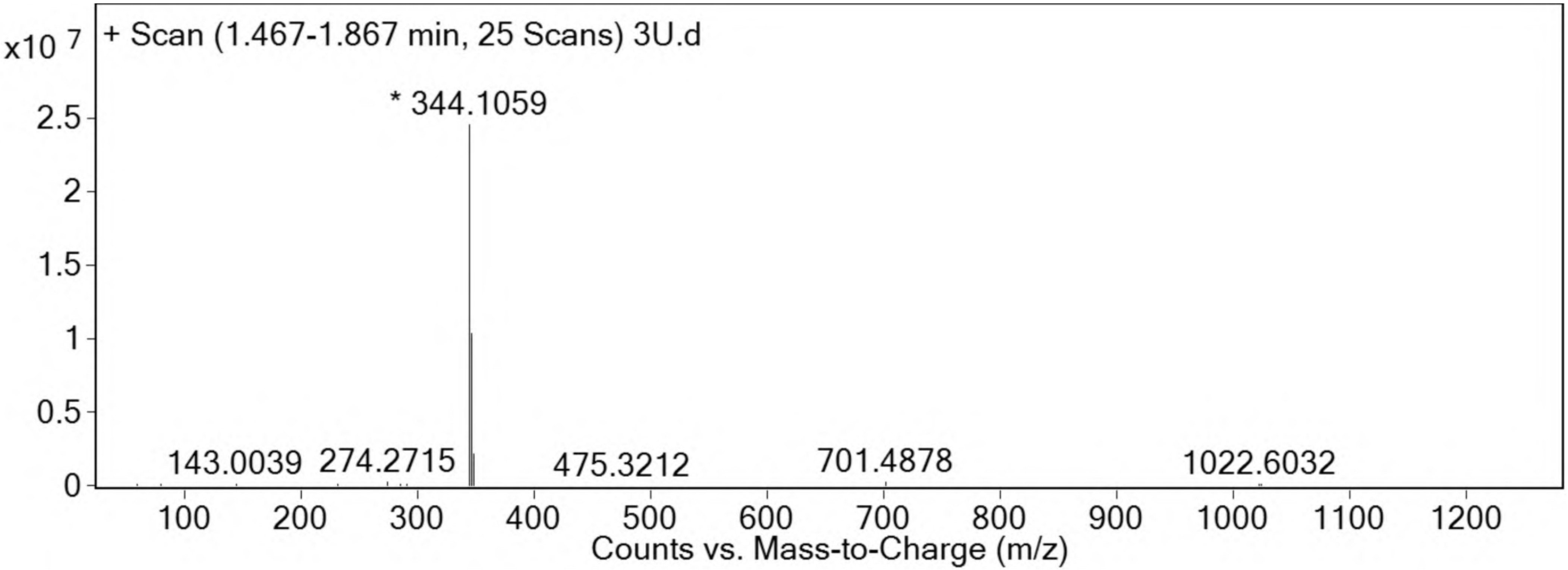
x10 7
+ Scan (1.467-1.867 min, 25 Scans) 3U.d
* 344.1059
143.0039
274.2715
475.3212
701.4878
1022.6032
2.5
2
1.5
1
0.5
0
100
200
300
400
500
600
700
800
900
1000
1100
1200
Counts vs. Mass-to-Charge (m/z)

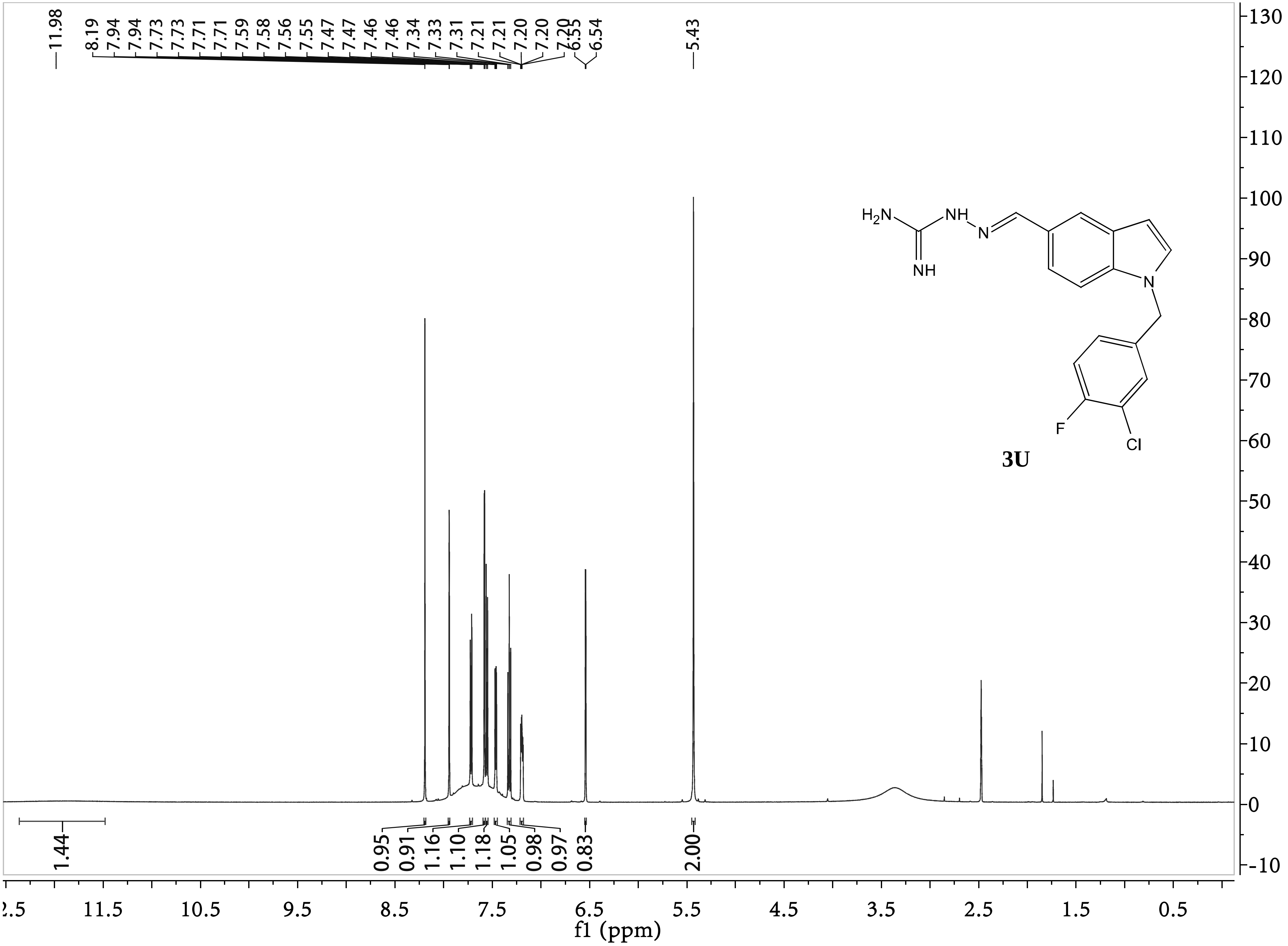

11.98
8.19
7.94
7.94
7.73
7.73
7.71
7.71
7.59
7.58
7.56
7.55
7.47
7.47
7.46
7.46
7.34
7.33
7.31
7.21
7.21
7.20
7.20
7.20
6.55
6.54
5.43
H2N
NH
N
NH
N
F
Cl
3U
1.44
0.95
0.91
1.16
1.10
1.18
1.05
0.98
0.97
0.83
2.00
f1 (ppm)

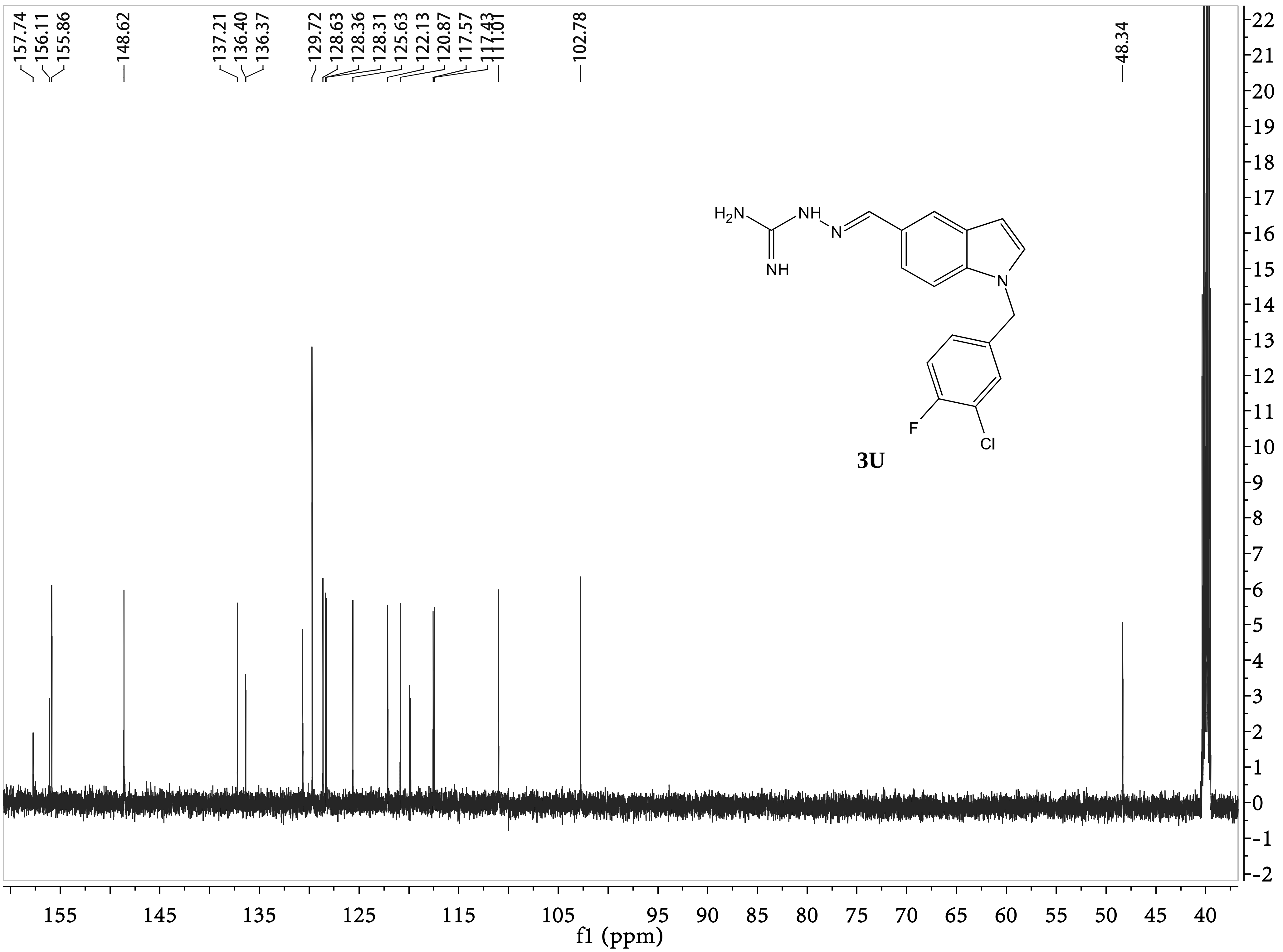

157.74
156.11
155.86
148.62
137.21
136.40
136.37
129.72
128.63
128.36
128.31
125.63
122.13
120.87
117.57
117.43
111.01
102.78
48.34
H_2N
NH
N
NH
N
F
Cl
3U
f1 (ppm)

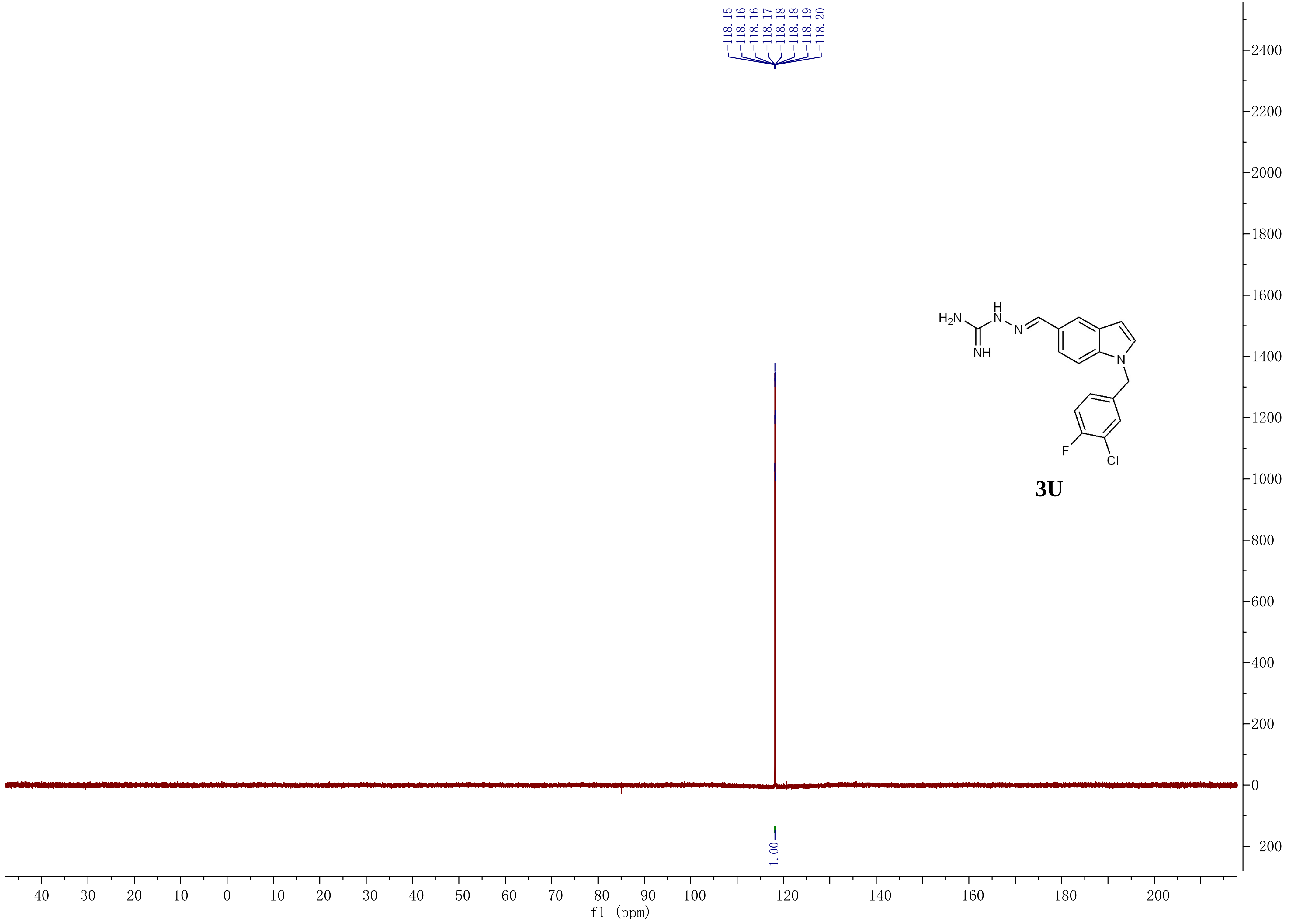

-118.15
-118.16
-118.16
-118.17
-118.18
-118.18
-118.19
-118.20
H2N
H
N
N
NH
N
F
Cl
3U
1.00
40 30 20 10 0 -10 -20 -30 -40 -50 -60 -70 -80 -90 -100 -120 -140 -160 -180 -200
f1 (ppm)
2400
2200
2000
1800
1600
1400
1200
1000
800
600
400
200
0
-200

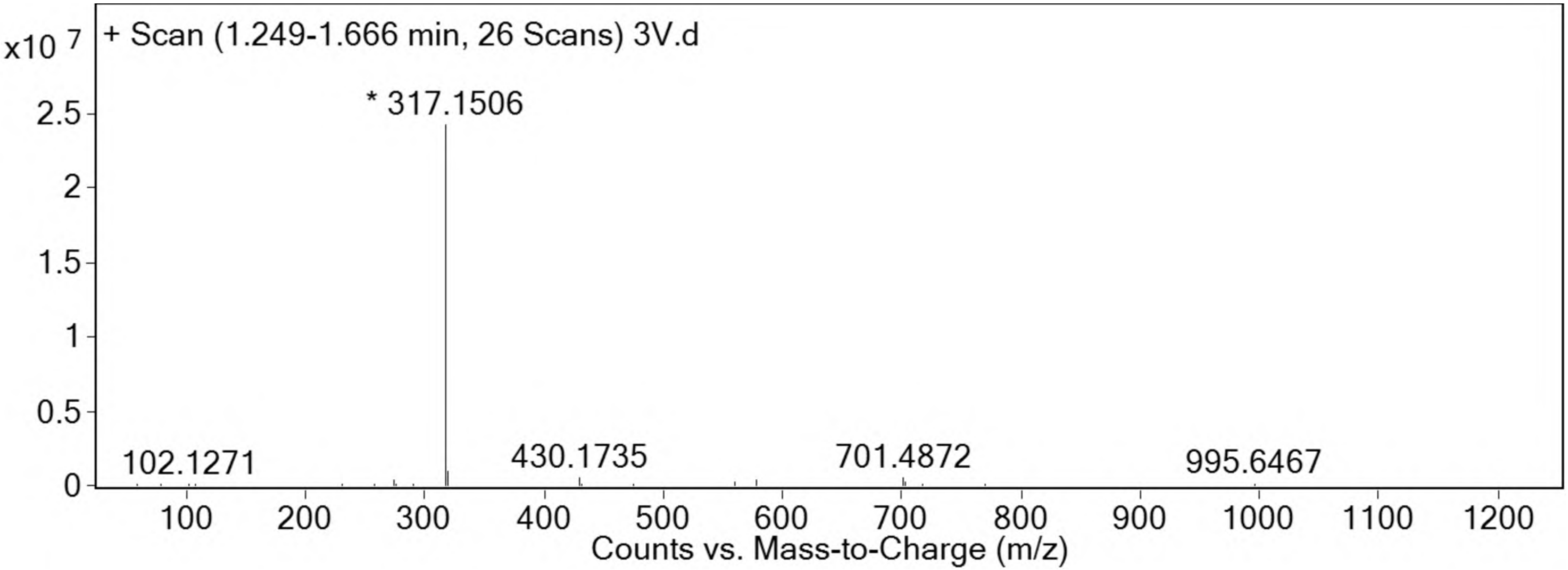
x10 7
+ Scan (1.249-1.666 min, 26 Scans) 3V.d
* 317.1506
102.1271
430.1735
701.4872
995.6467
2.5
2
1.5
1
0.5
0
100
200
300
400
500
600
700
800
900
1000
1100
1200
Counts vs. Mass-to-Charge (m/z)

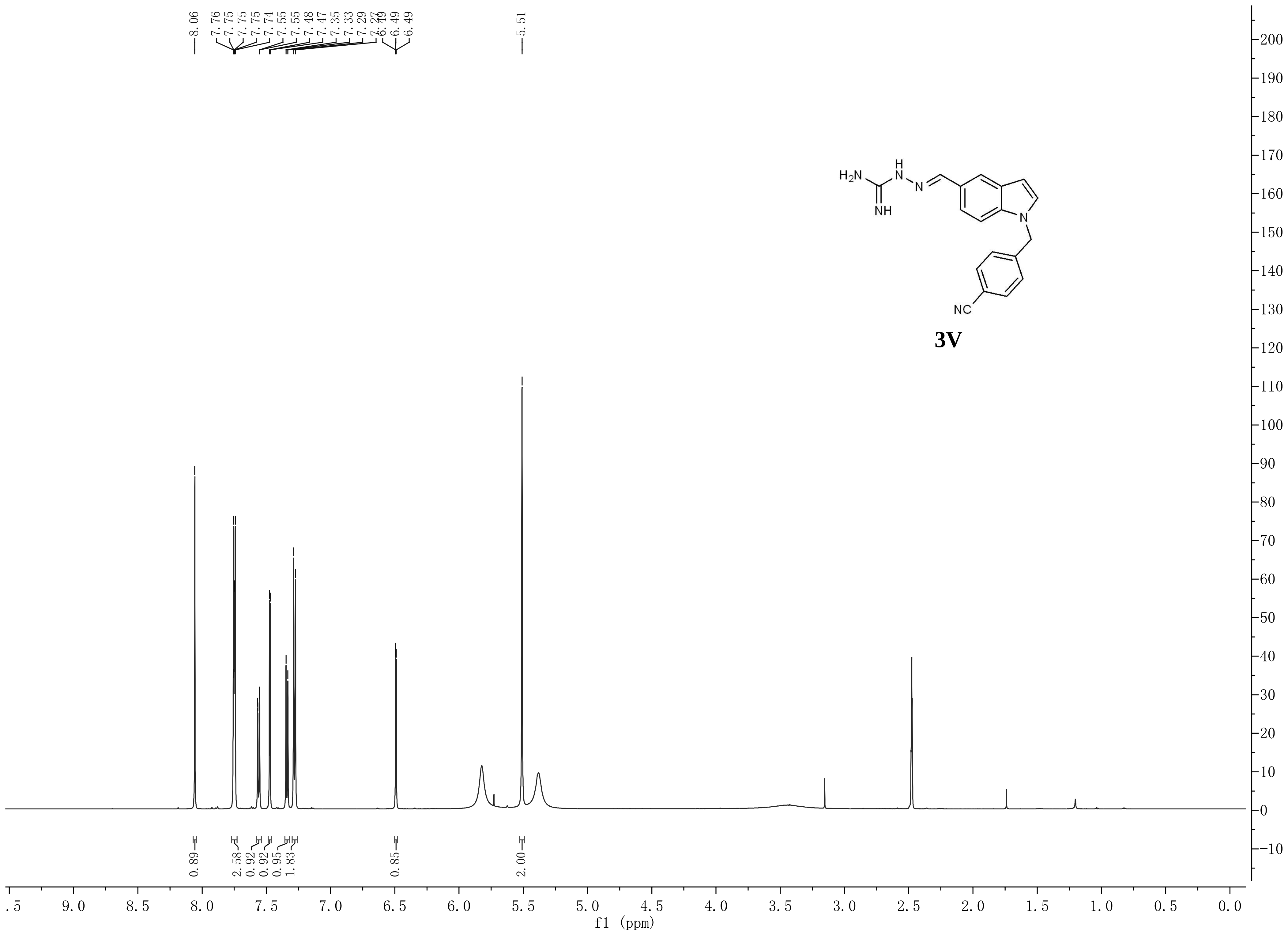
8.06
7.76
7.75
7.75
7.75
7.74
7.55
7.55
7.48
7.47
7.35
7.33
7.29
7.27
6.49
6.49
6.49
5.51
H2N
H
N
N
NH
N
NC
3V
0.89
2.58
0.92
0.92
0.95
1.83
0.85
2.00
.5
9.0
8.5
8.0
7.5
7.0
6.5
6.0
5.5
5.0
4.5
4.0
3.5
3.0
2.5
2.0
1.5
1.0
0.5
0.0
f1 (ppm)
200
190
180
170
160
150
140
130
120
110
100
90
80
70
60
50
40
30
20
10
0
-10

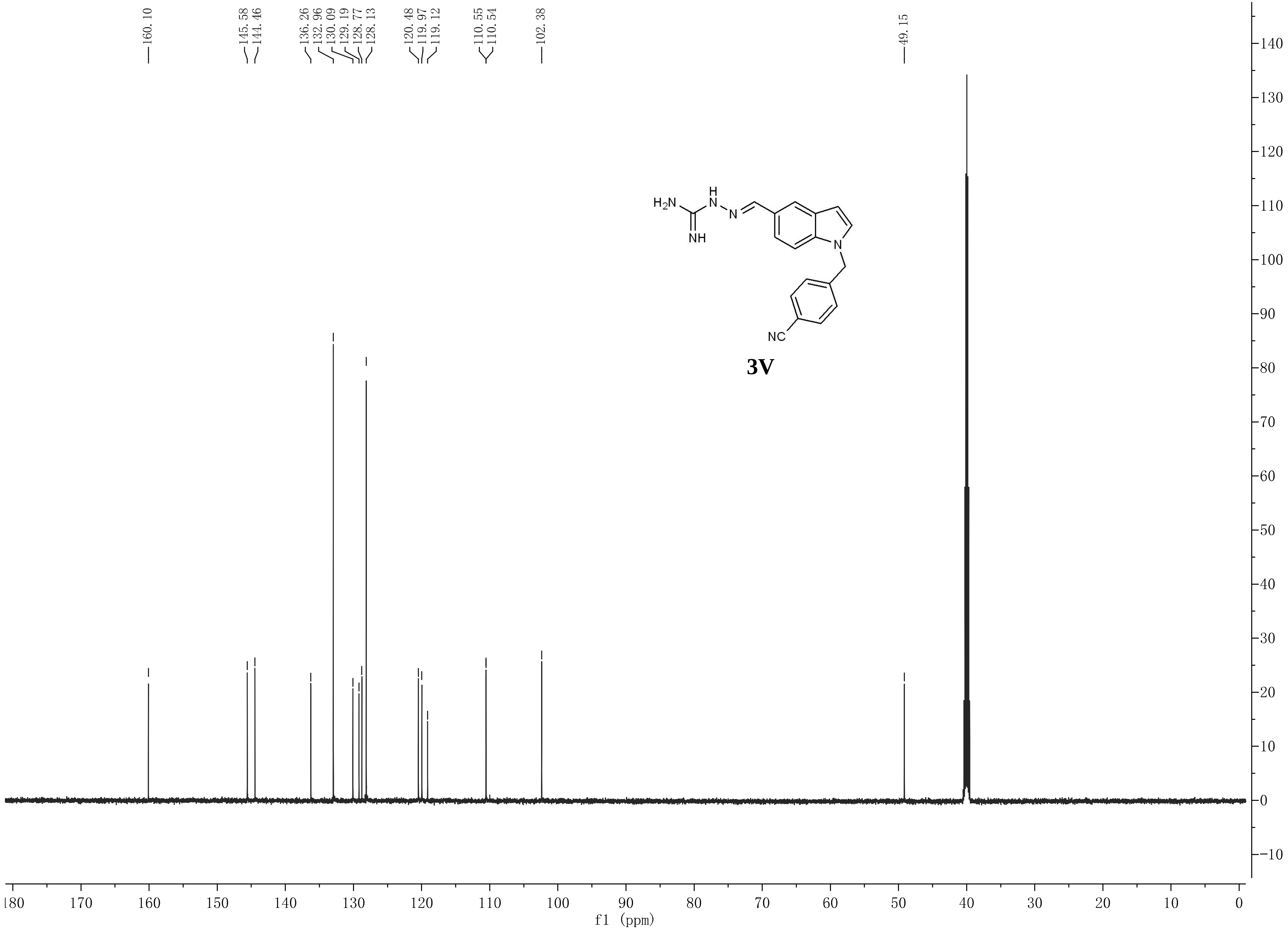
160.10
145.58
144.46
136.26
132.96
130.09
129.19
128.77
128.13
120.48
119.97
119.12
110.55
110.54
102.38
49.15
H2N
H
N
N
NH
N
NC
3V
f1 (ppm)

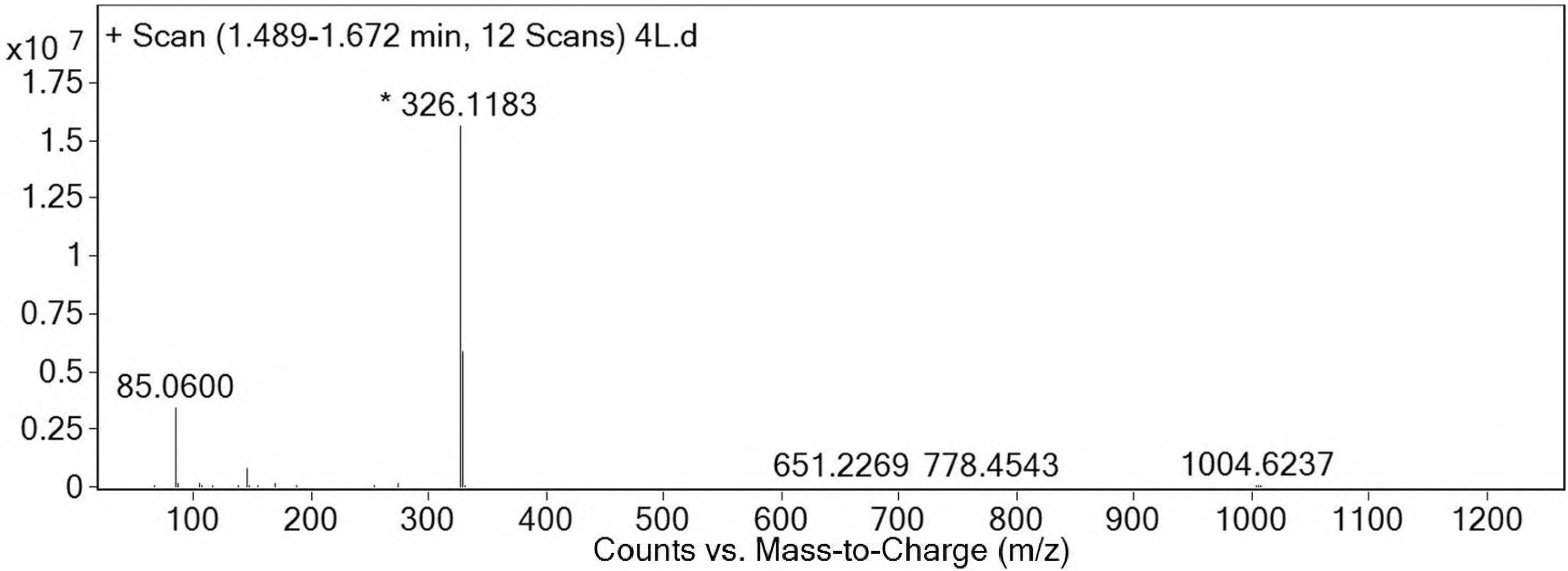
+ Scan (1.489-1.672 min, 12 Scans) 4L.d
x10 7
1.75
1.5
1.25
1
0.75
0.5
0.25
0
* 326.1183
85.0600
651.2269
778.4543
1004.6237
100
200
300
400
500
600
700
800
900
1000
1100
1200
Counts vs. Mass-to-Charge (m/z)

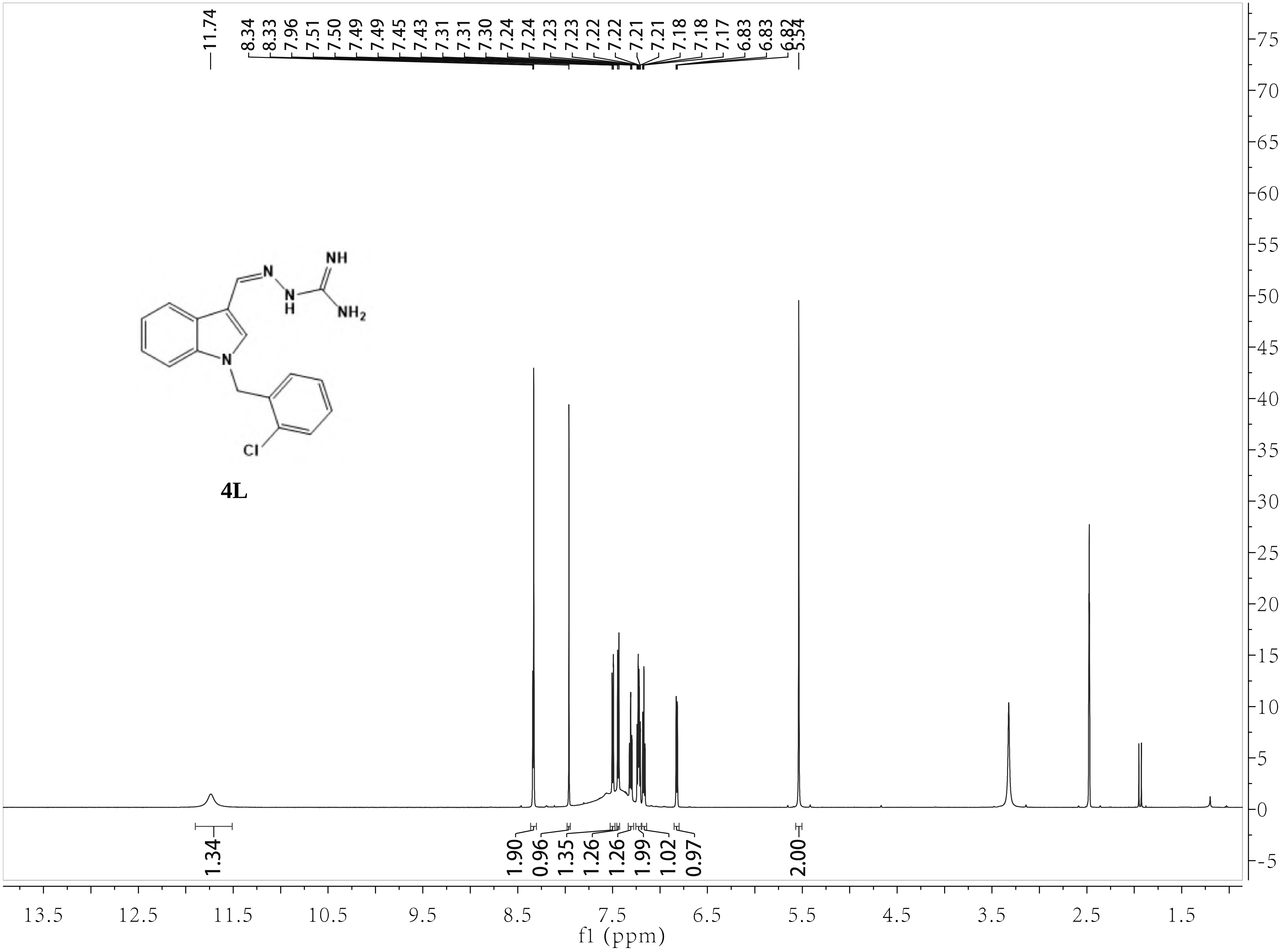
11.74
8.34
8.33
7.96
7.51
7.50
7.49
7.49
7.45
7.43
7.31
7.31
7.30
7.24
7.24
7.23
7.23
7.22
7.22
7.21
7.21
7.18
7.18
7.17
6.83
6.83
6.82
5.54
NH
N
N
H
NH2
N
Cl
4L
1.34
1.90
0.96
1.35
1.26
1.26
1.99
1.02
0.97
2.00
13.5
12.5
11.5
10.5
9.5
8.5
7.5
6.5
5.5
4.5
3.5
2.5
1.5
f1 (ppm)
75
70
65
60
55
50
45
40
35
30
25
20
15
10
5
0
-5

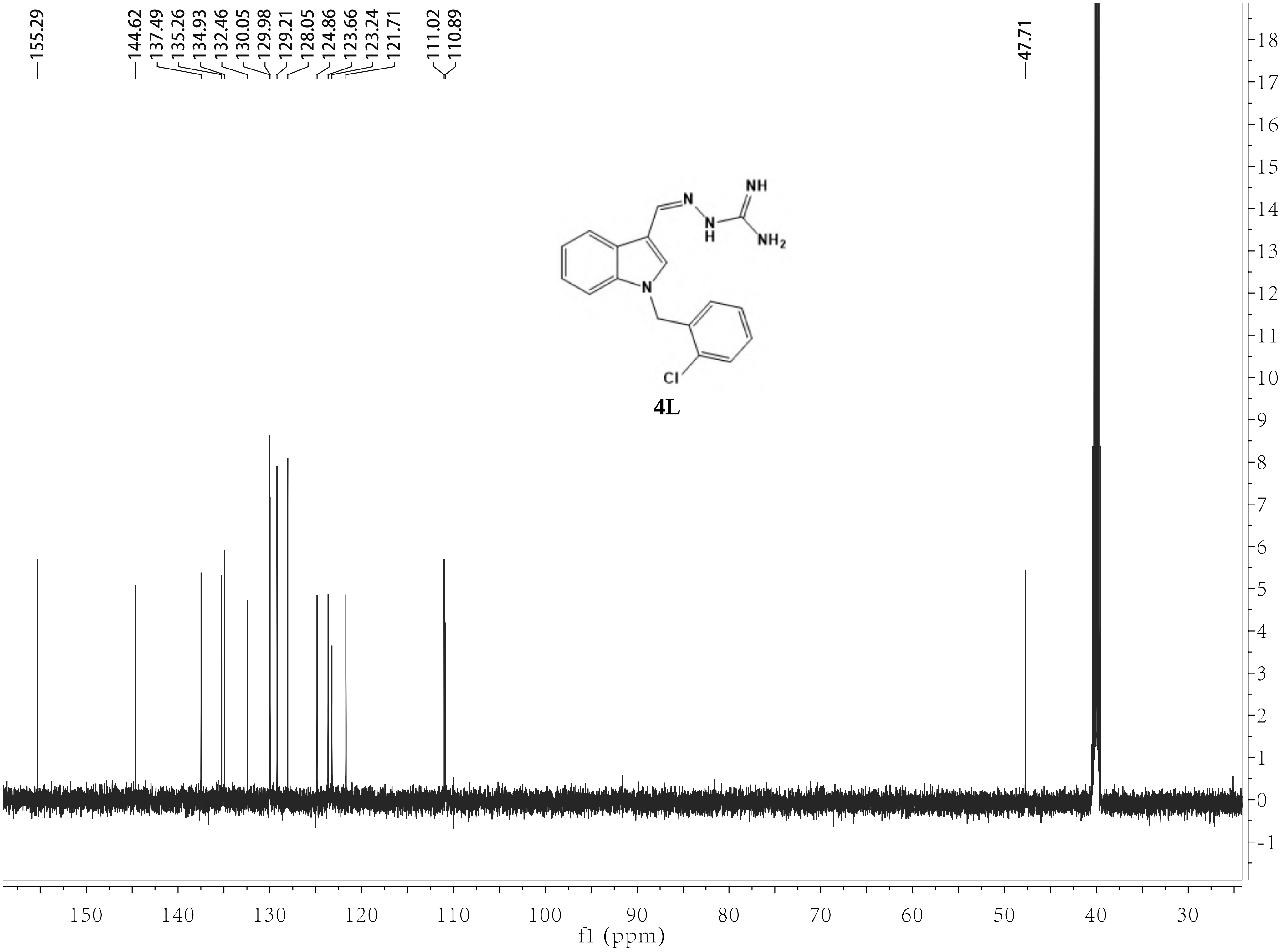
155.29
144.62
137.49
135.26
134.93
132.46
130.05
129.98
129.21
128.05
124.86
123.66
123.24
121.71
111.02
110.89
47.71
NH
N
N
H
NH2
N
Cl
4L
f1 (ppm)

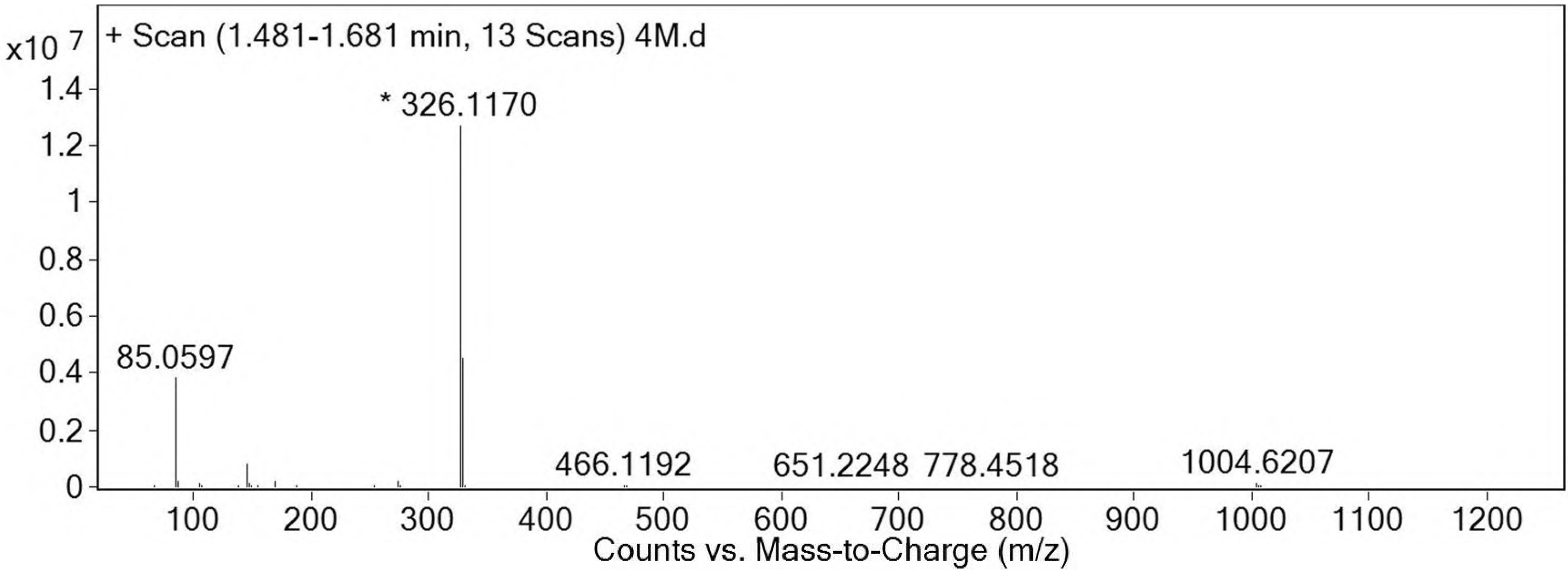

x10 7
+ Scan (1.481-1.681 min, 13 Scans) 4M.d
1.4
1.2
1
0.8
0.6
0.4
0.2
0
* 326.1170
85.0597
466.1192
651.2248
778.4518
1004.6207
100
200
300
400
500
600
700
800
900
1000
1100
1200
Counts vs. Mass-to-Charge (m/z)

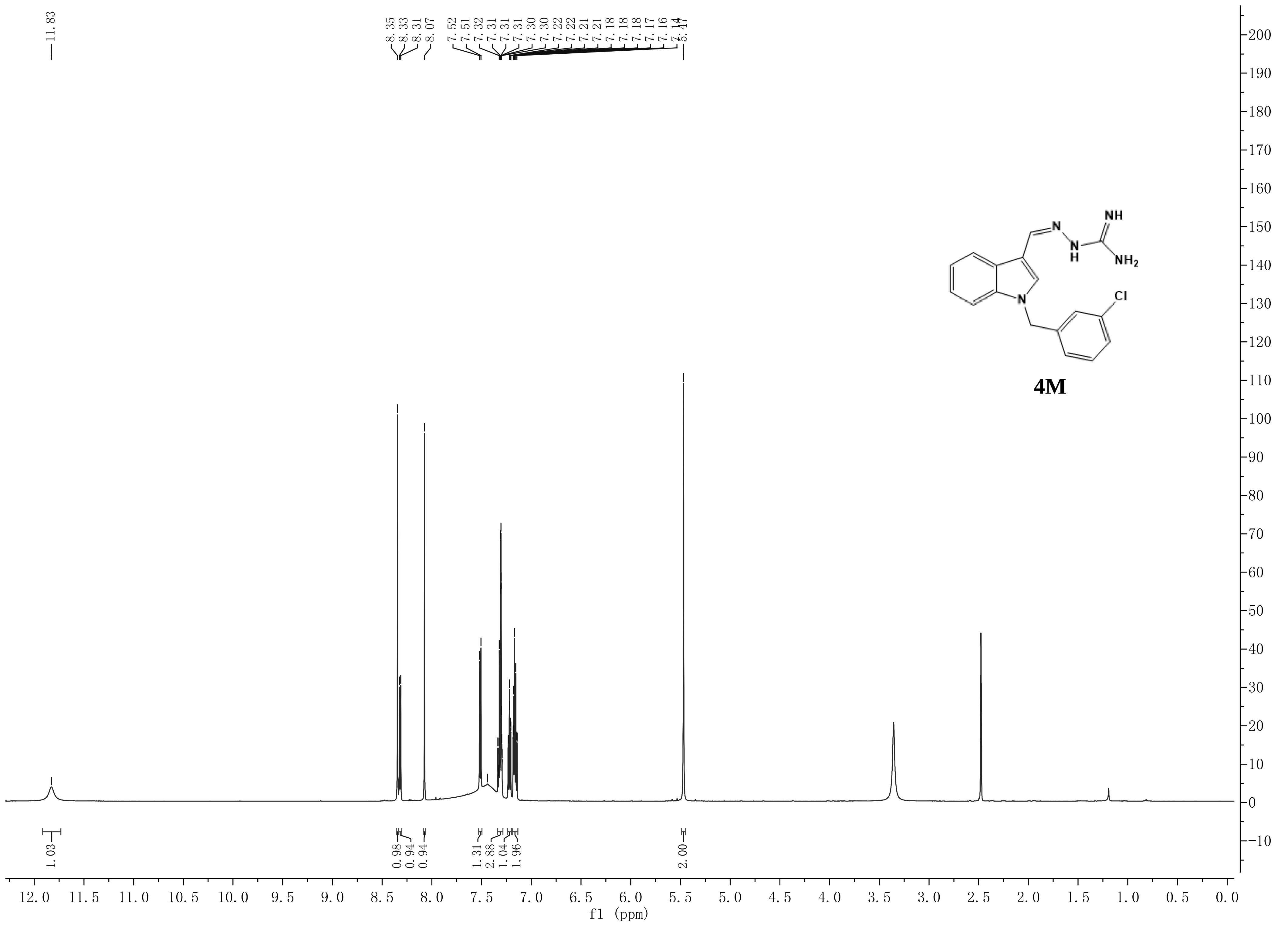
11.83
8.35
8.33
8.31
8.07
7.52
7.51
7.32
7.31
7.31
7.31
7.30
7.30
7.22
7.22
7.21
7.21
7.18
7.18
7.18
7.17
7.16
7.14
5.47
NH
N
N
H
NH2
N
Cl
4M
1.03
0.98
0.94
0.94
1.31
2.88
1.04
1.96
2.00
f1 (ppm)

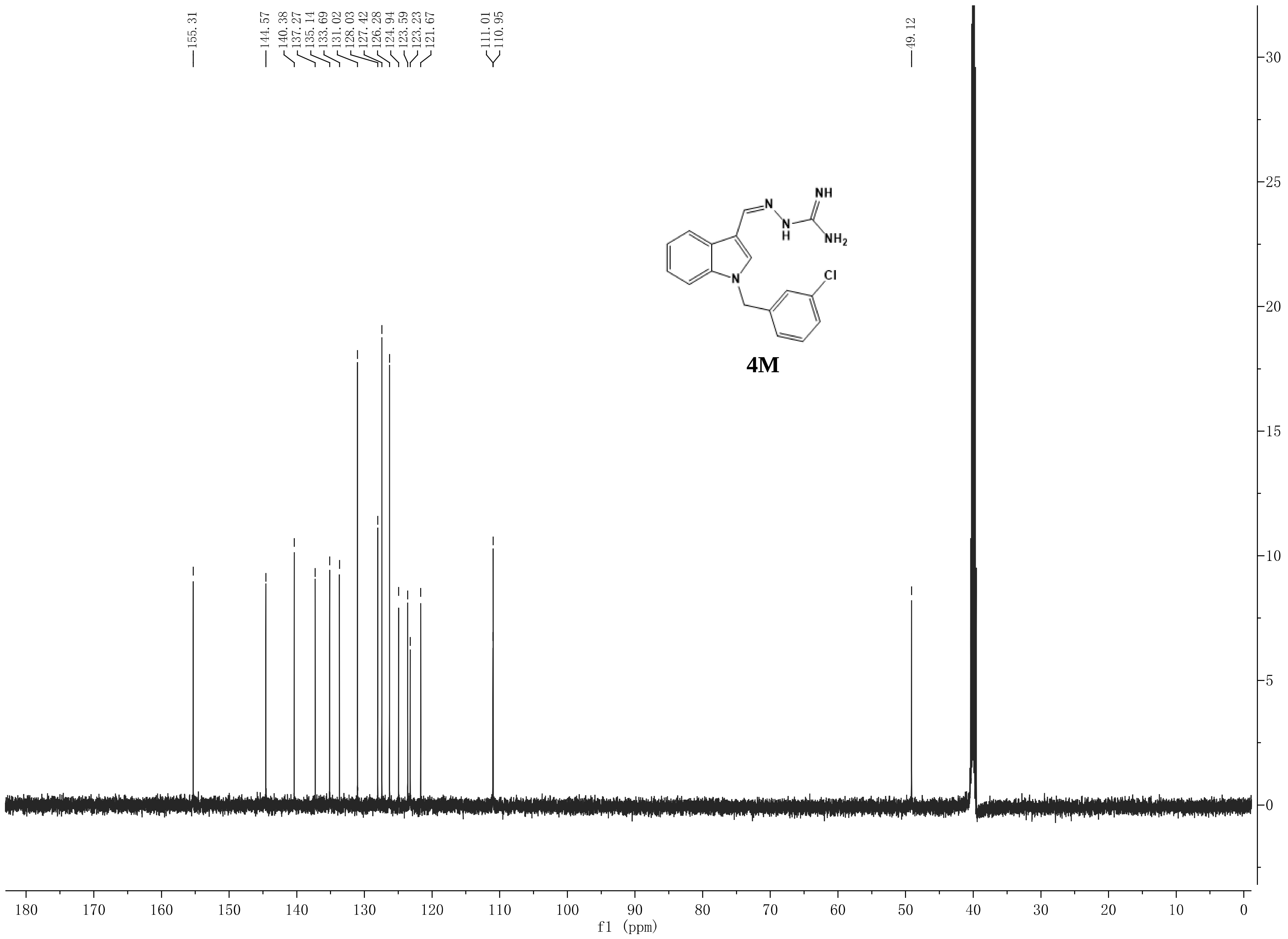
155.31
144.57
140.38
137.27
135.14
133.69
131.02
128.03
127.42
126.28
124.94
123.59
123.23
121.67
111.01
110.95
49.12
NH
N
N
H
NH2
N
Cl
4M
f1 (ppm)

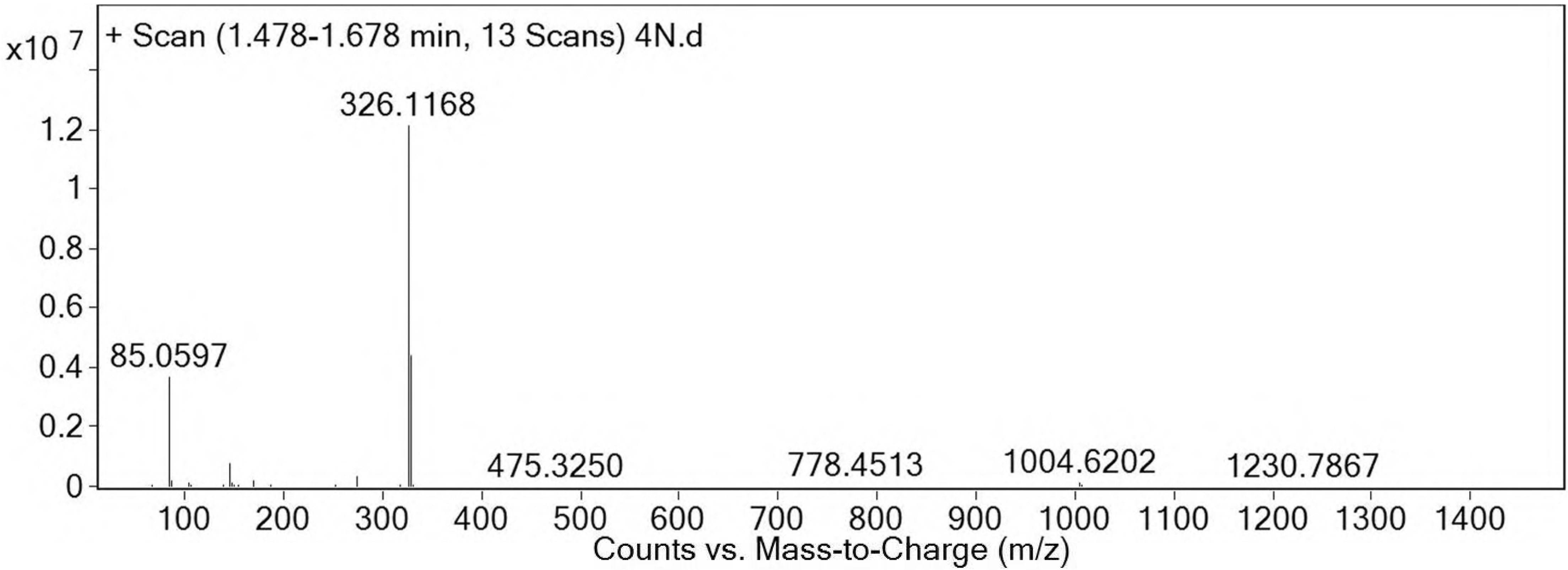

+ Scan (1.478-1.678 min, 13 Scans) 4N.d
x10 7
1.2
1
0.8
0.6
0.4
0.2
0
326.1168
85.0597
475.3250
778.4513
1004.6202
1230.7867
100
200
300
400
500
600
700
800
900
1000
1100
1200
1300
1400
Counts vs. Mass-to-Charge (m/z)

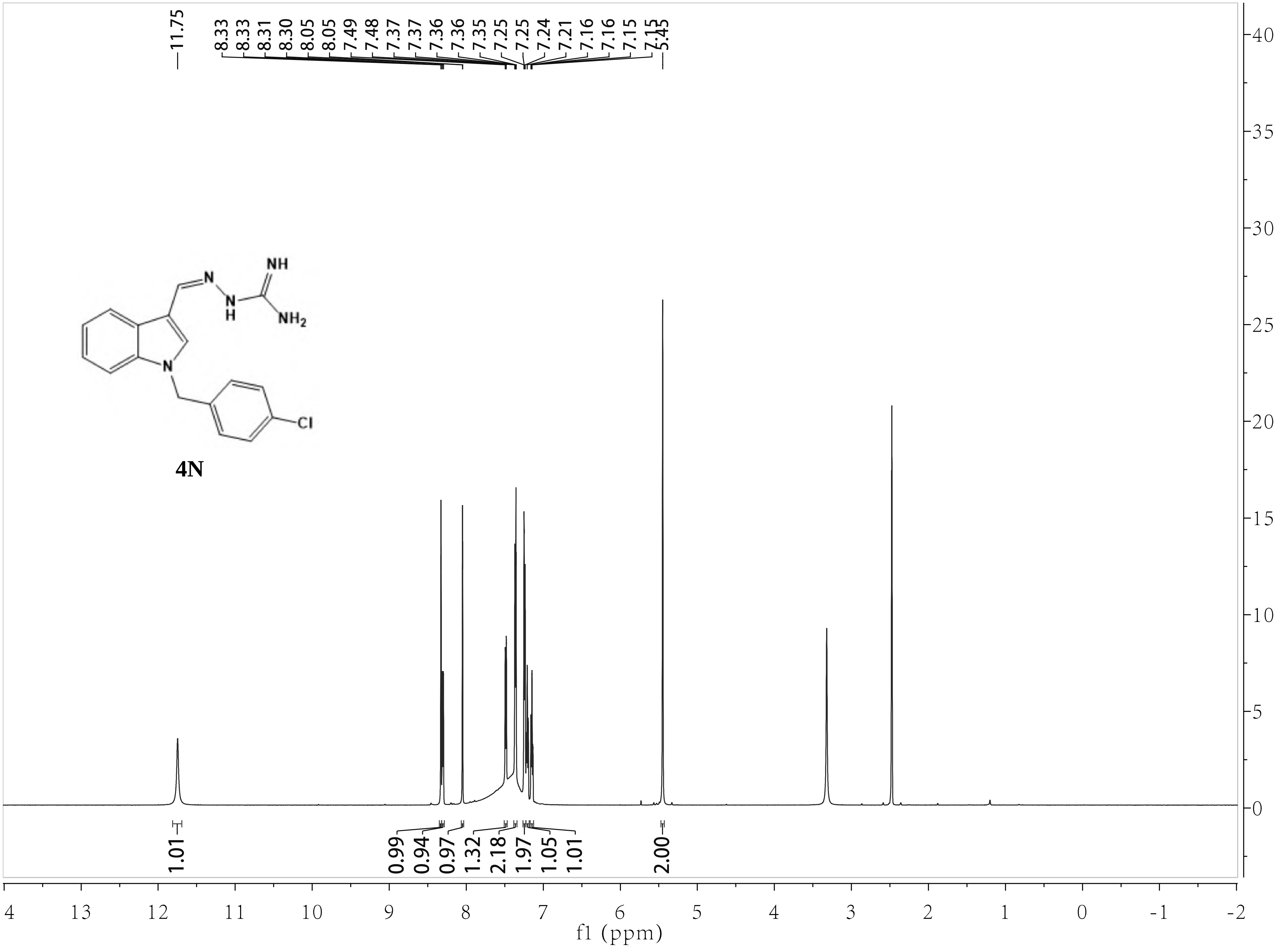
11.75
8.33
8.33
8.31
8.30
8.05
8.05
7.49
7.48
7.37
7.37
7.36
7.36
7.35
7.25
7.25
7.24
7.21
7.16
7.16
7.15
7.15
5.45
NH
N
N
H
NH2
N
Cl
4N
1.01
0.99
0.94
0.97
1.32
2.18
1.97
1.97
1.05
1.01
2.00
4
13
12
11
10
9
8
7
6
5
4
3
2
1
0
-1
-2
f1 (ppm)
40
35
30
25
20
15
10
5
0

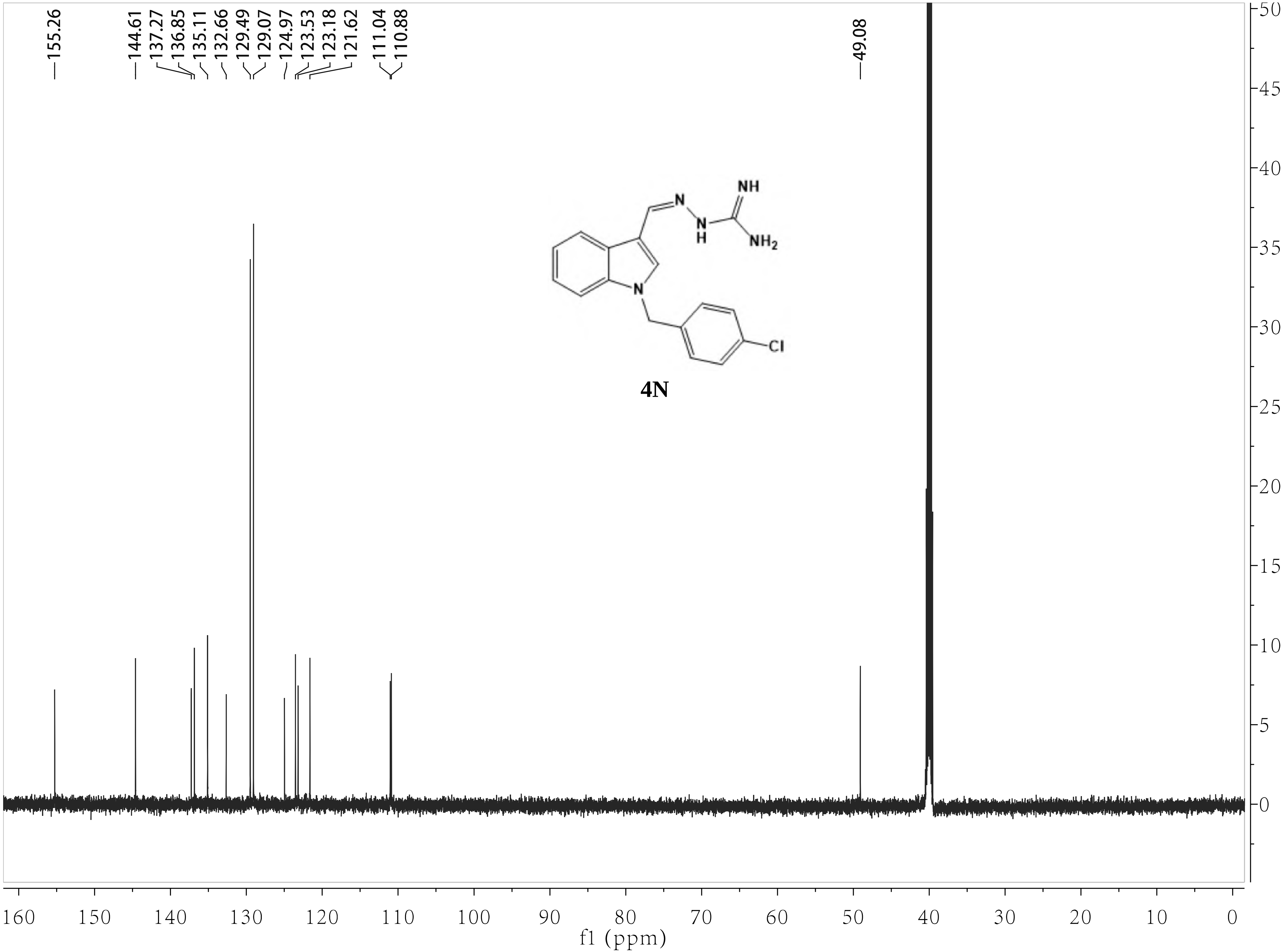
155.26
144.61
137.27
136.85
135.11
132.66
129.49
129.07
124.97
123.53
123.18
121.62
111.04
110.88
49.08
NH
N
N
H
NH2
N
Cl
4N
f1 (ppm)

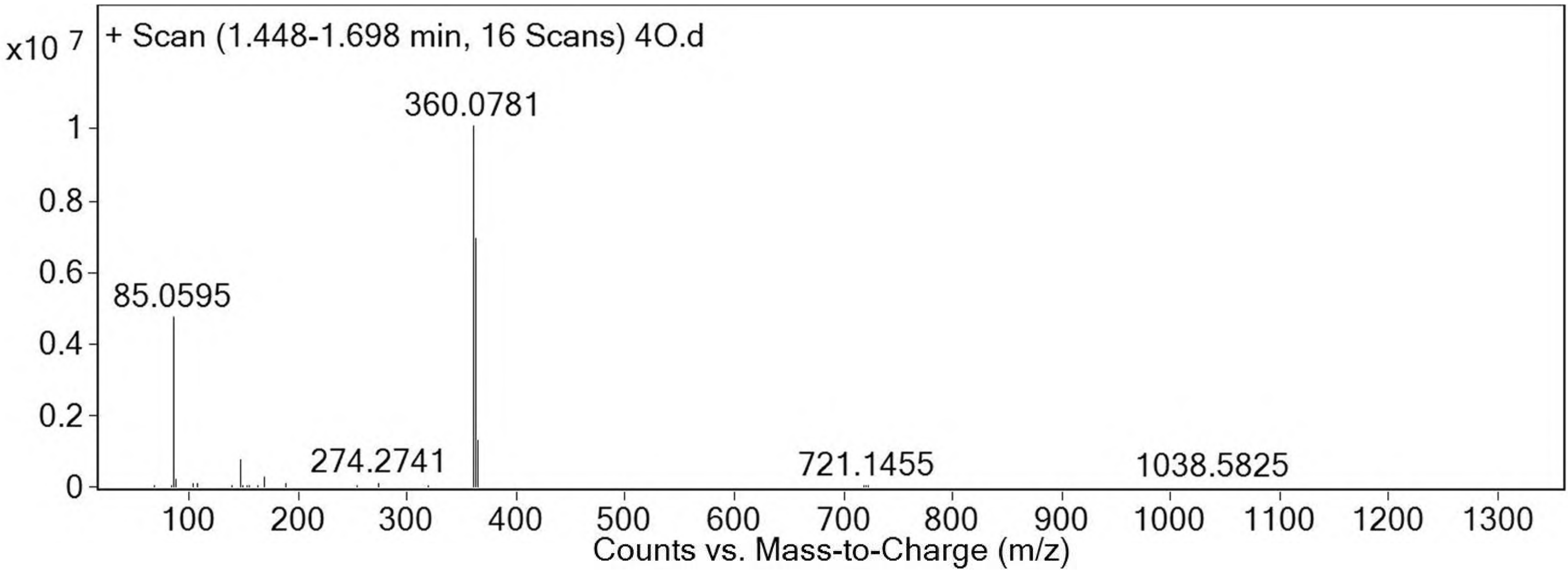

x10 7
+ Scan (1.448-1.698 min, 16 Scans) 4O.d
360.0781
85.0595
274.2741
721.1455
1038.5825
1
0.8
0.6
0.4
0.2
0
100
200
300
400
500
600
700
800
900
1000
1100
1200
1300
Counts vs. Mass-to-Charge (m/z)

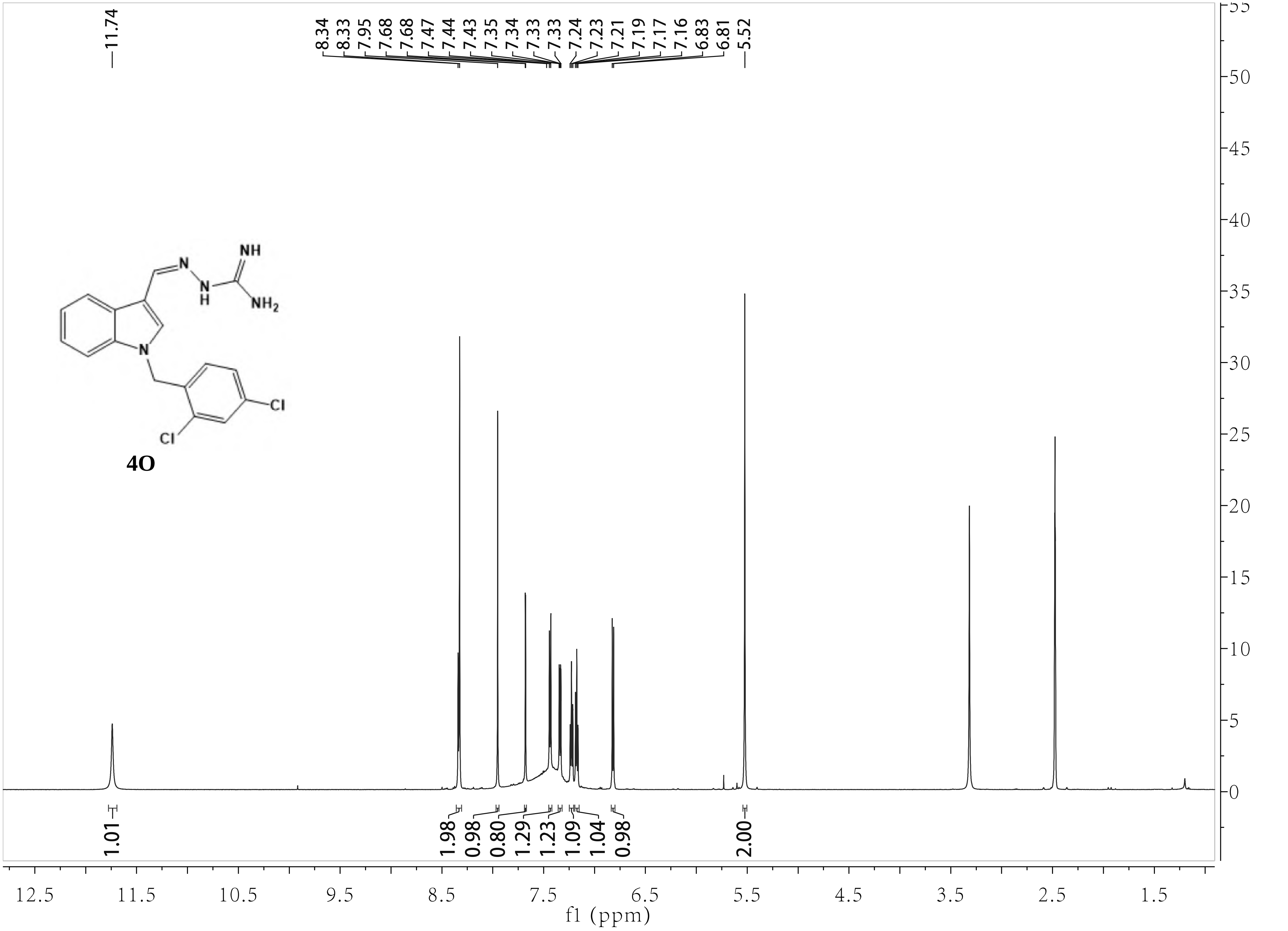
11.74
8.34
8.33
7.95
7.68
7.68
7.47
7.44
7.43
7.35
7.34
7.33
7.33
7.24
7.23
7.21
7.19
7.17
7.16
6.83
6.81
5.52
NH
N
N
H
NH2
N
Cl
Cl
4O
1.01
1.98
0.98
0.80
1.29
1.23
1.09
1.04
0.98
2.00
12.5
11.5
10.5
9.5
8.5
7.5
6.5
5.5
4.5
3.5
2.5
1.5
f1 (ppm)
55
50
45
40
35
30
25
20
15
10
5
0

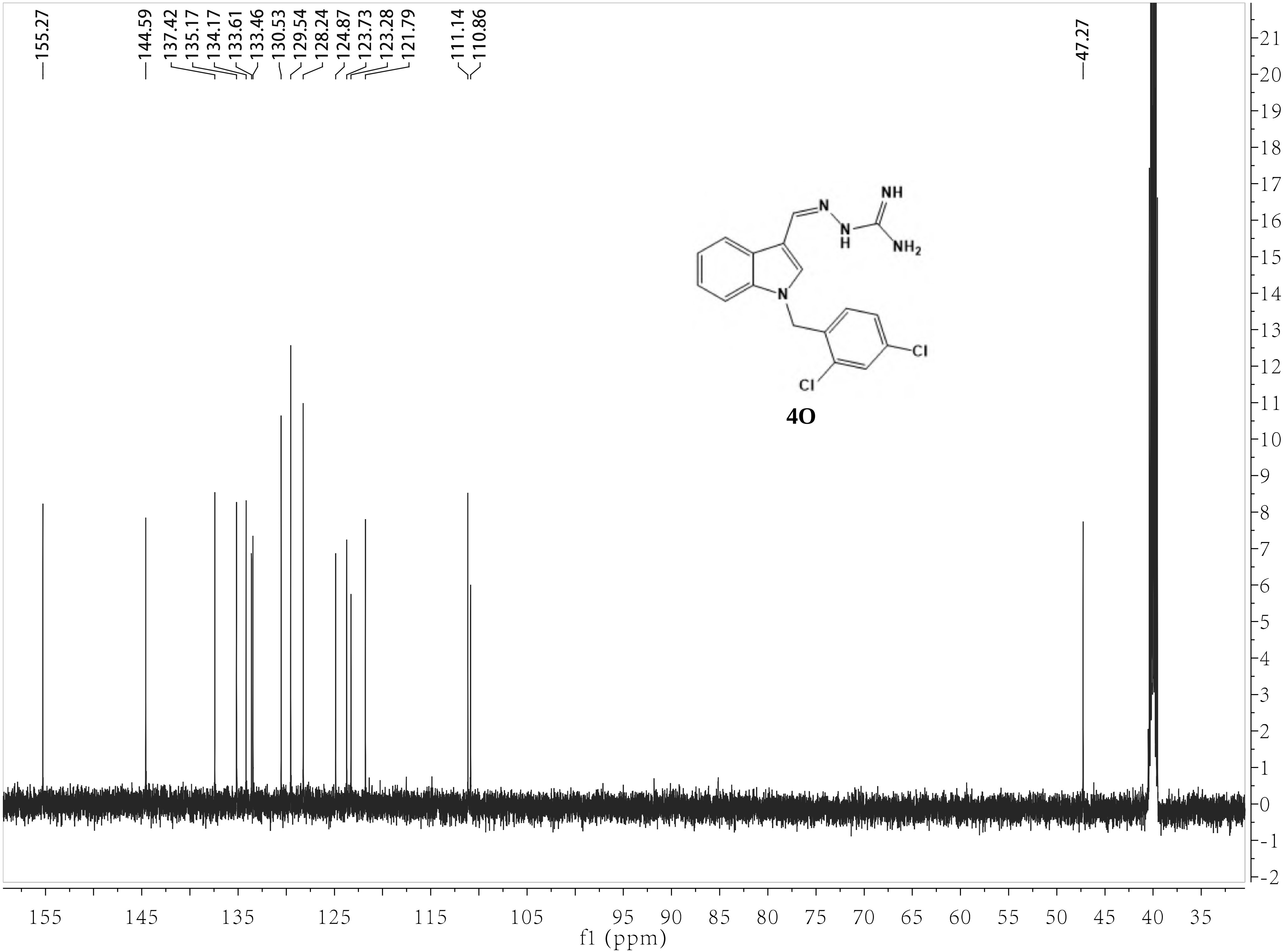
155.27
144.59
137.42
135.17
134.17
133.61
133.46
130.53
129.54
128.24
124.87
123.73
123.28
121.79
111.14
110.86
47.27
NH
N
N
H
NH2
N
Cl
Cl
4O
f1 (ppm)

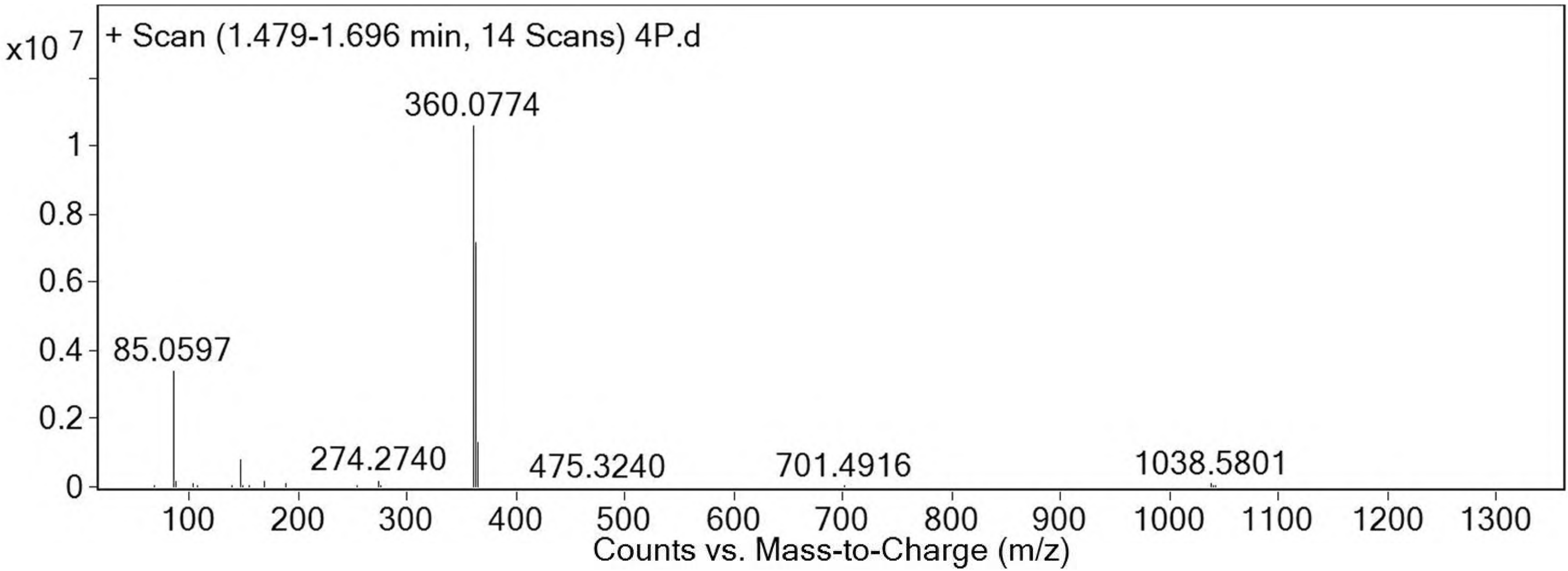
+ Scan (1.479-1.696 min, 14 Scans) 4P.d
x10 7
360.0774
85.0597
274.2740
475.3240
701.4916
1038.5801
0
0.2
0.4
0.6
0.8
1
100
200
300
400
500
600
700
800
900
1000
1100
1200
1300
Counts vs. Mass-to-Charge (m/z)

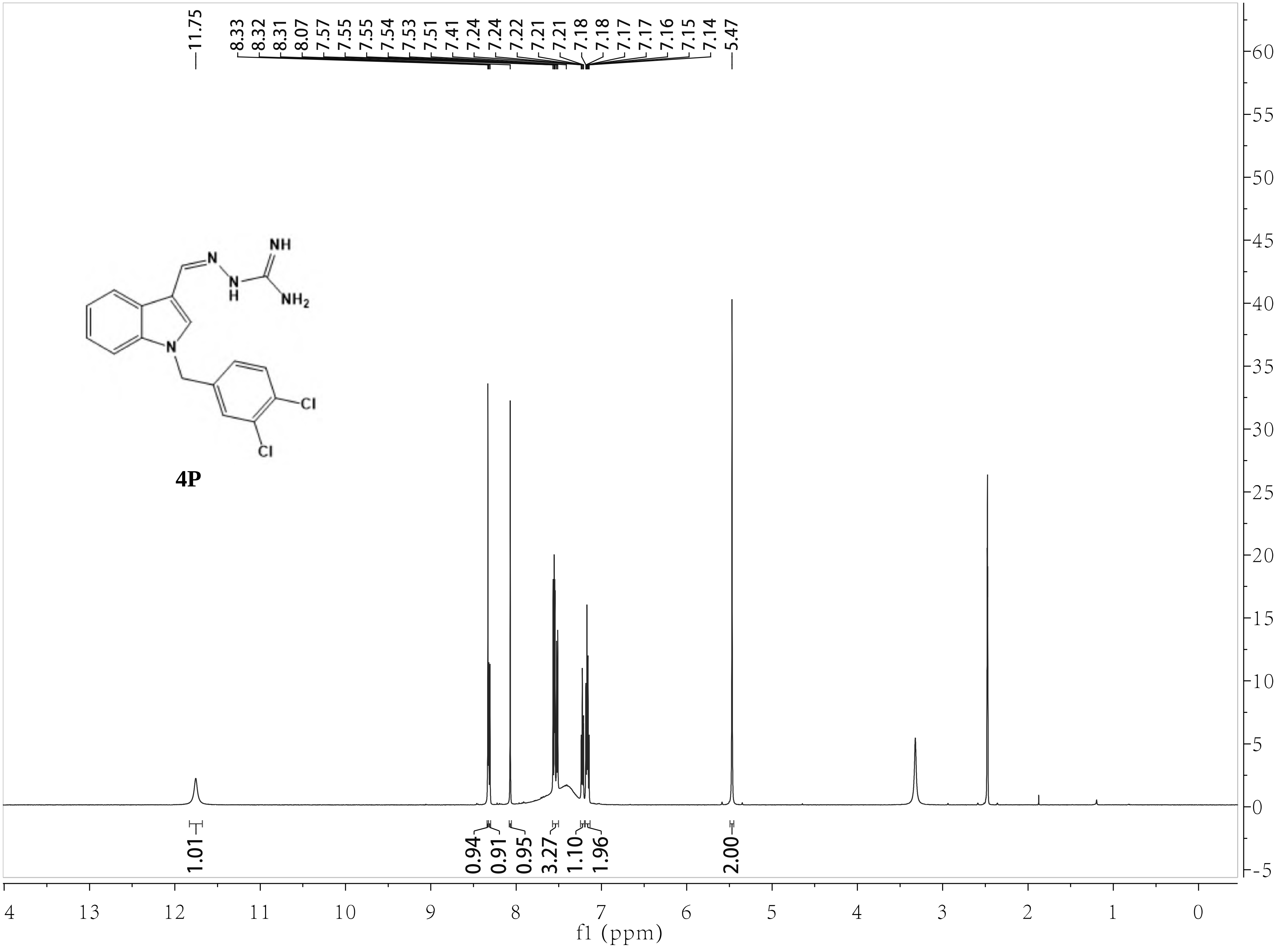
11.75
8.33
8.32
8.31
8.07
7.57
7.55
7.55
7.54
7.53
7.51
7.41
7.24
7.24
7.22
7.21
7.21
7.18
7.18
7.17
7.17
7.16
7.15
7.14
5.47
NH
N
N
H
NH2
N
Cl
Cl
4P
1.01
0.94
0.91
0.95
3.27
1.10
1.96
2.00
f1 (ppm)

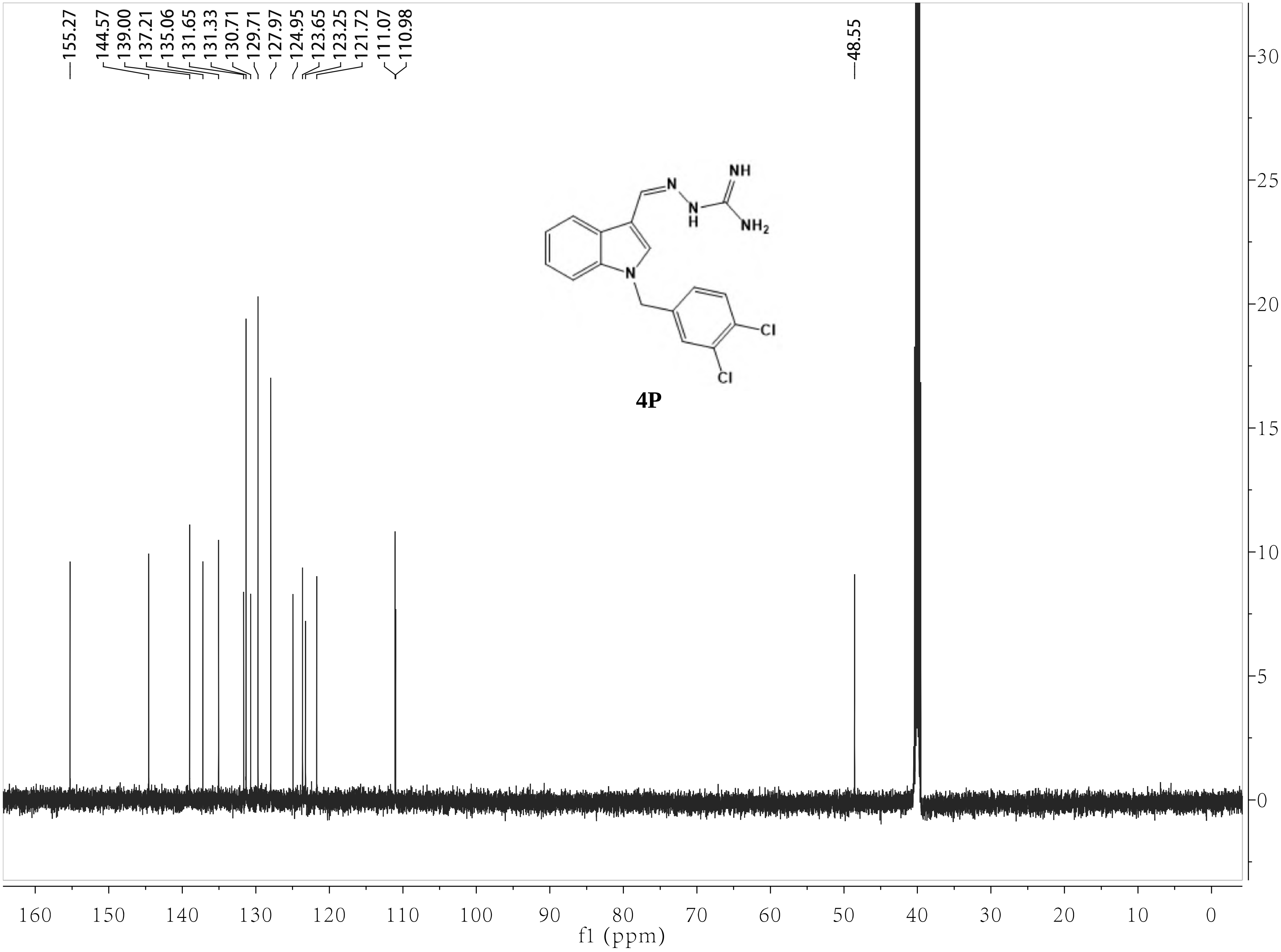

155.27
144.57
139.00
137.21
135.06
131.65
131.33
130.71
129.71
127.97
124.95
123.65
123.25
121.72
111.07
110.98
48.55
NH
N
N
H
NH2
N
Cl
Cl
4P
160 150 140 130 120 110 100 90 80 70 60 50 40 30 20 10 0
f1 (ppm)
30 25 20 15 10 5 0

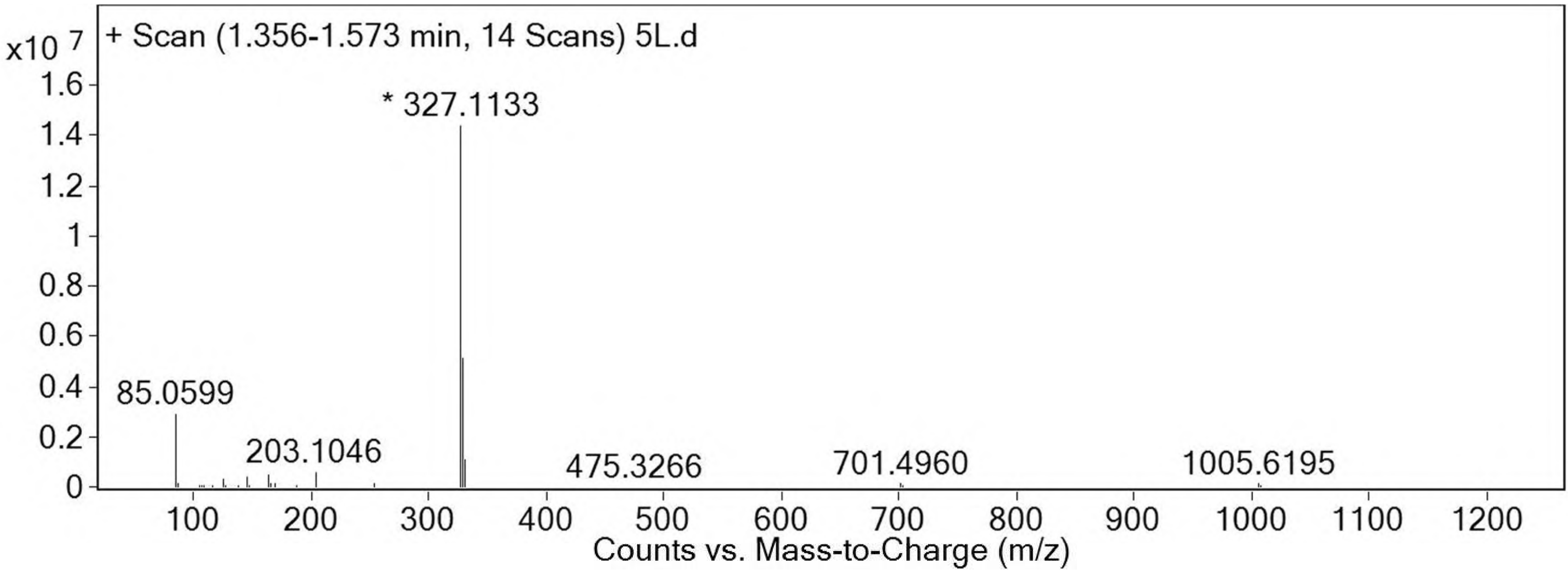

x10 7
+ Scan (1.356-1.573 min, 14 Scans) 5L.d
1.6
1.4
1.2
1
0.8
0.6
0.4
0.2
0
* 327.1133
85.0599
203.1046
475.3266
701.4960
1005.6195
100
200
300
400
500
600
700
800
900
1000
1100
1200
Counts vs. Mass-to-Charge (m/z)

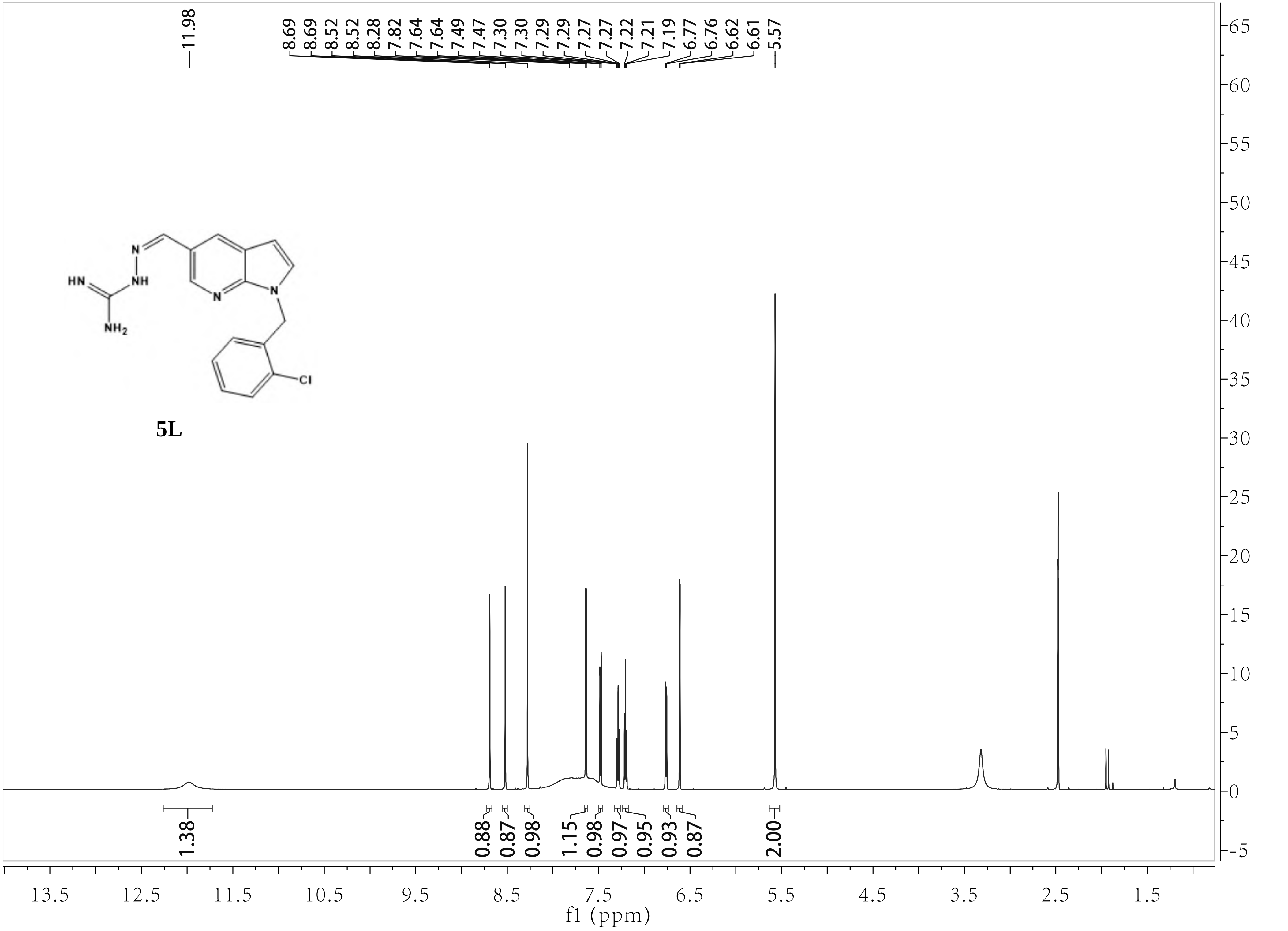

11.98
8.69 8.69 8.52 8.52 8.28 7.82 7.64 7.64 7.49 7.47 7.30 7.30 7.29 7.29 7.27 7.27 7.22 7.21 7.19 6.77 6.76 6.62 6.61
5.57
HN
NH
N
NH2
N
N
Cl
5L
1.38
0.88 0.87 0.98 1.15 0.98 0.97 0.95 0.93 0.87
2.00
13.5 12.5 11.5 10.5 9.5 8.5 7.5 6.5 5.5 4.5 3.5 2.5 1.5
f1 (ppm)
65 60 55 50 45 40 35 30 25 20 15 10 5 0 -5

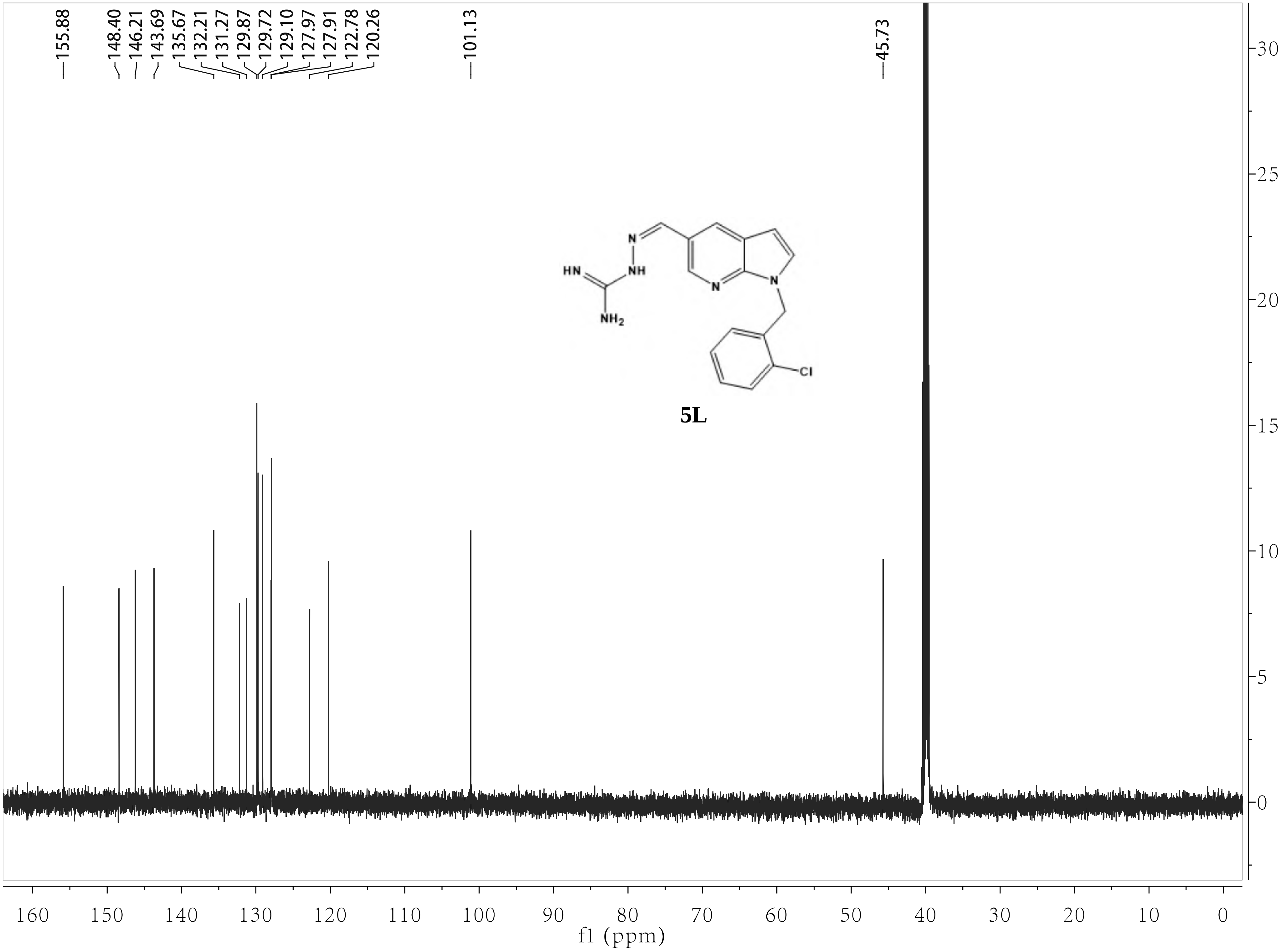
155.88
148.40
146.21
143.69
135.67
132.21
131.27
129.87
129.72
129.10
127.97
127.91
122.78
120.26
101.13
45.73
HN
N
NH
NH2
N
N
Cl
5L
160 150 140 130 120 110 100 90 80 70 60 50 40 30 20 10 0
f1 (ppm)
30 25 20 15 10 5 0

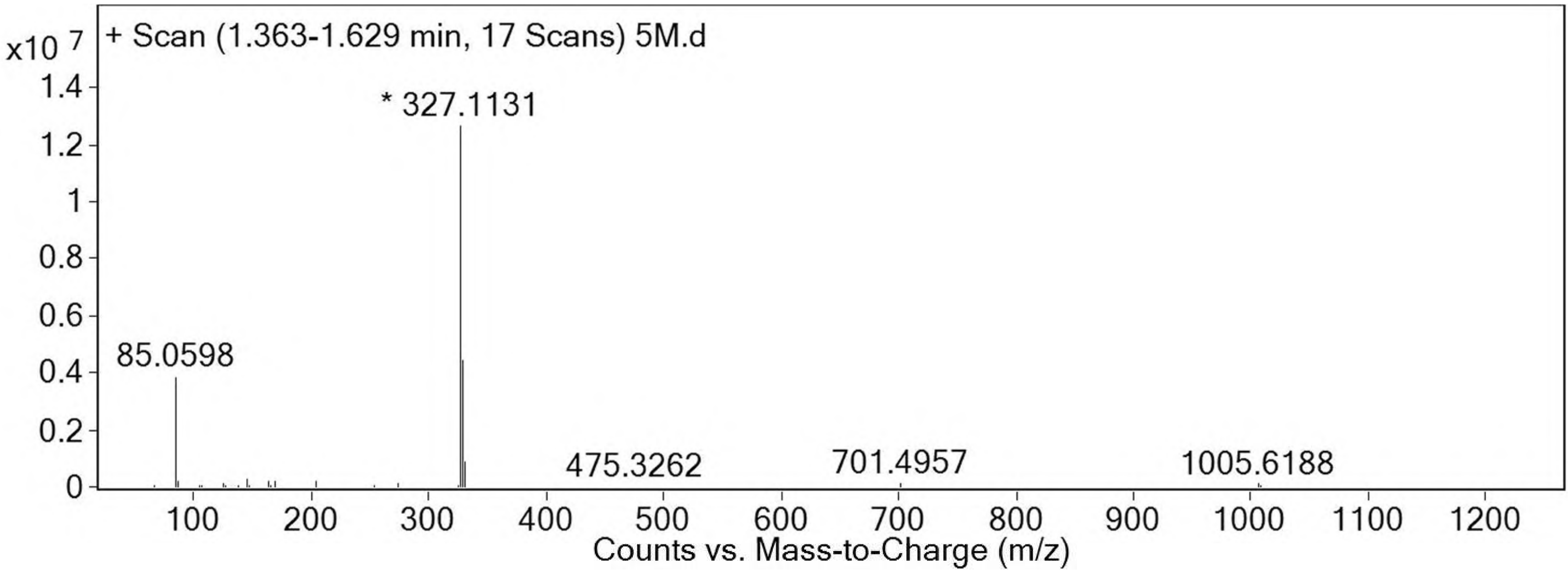

x10 7
+ Scan (1.363-1.629 min, 17 Scans) 5M.d
1.4
1.2
1
0.8
0.6
0.4
0.2
0
* 327.1131
85.0598
475.3262
701.4957
1005.6188
100
200
300
400
500
600
700
800
900
1000
1100
1200
Counts vs. Mass-to-Charge (m/z)

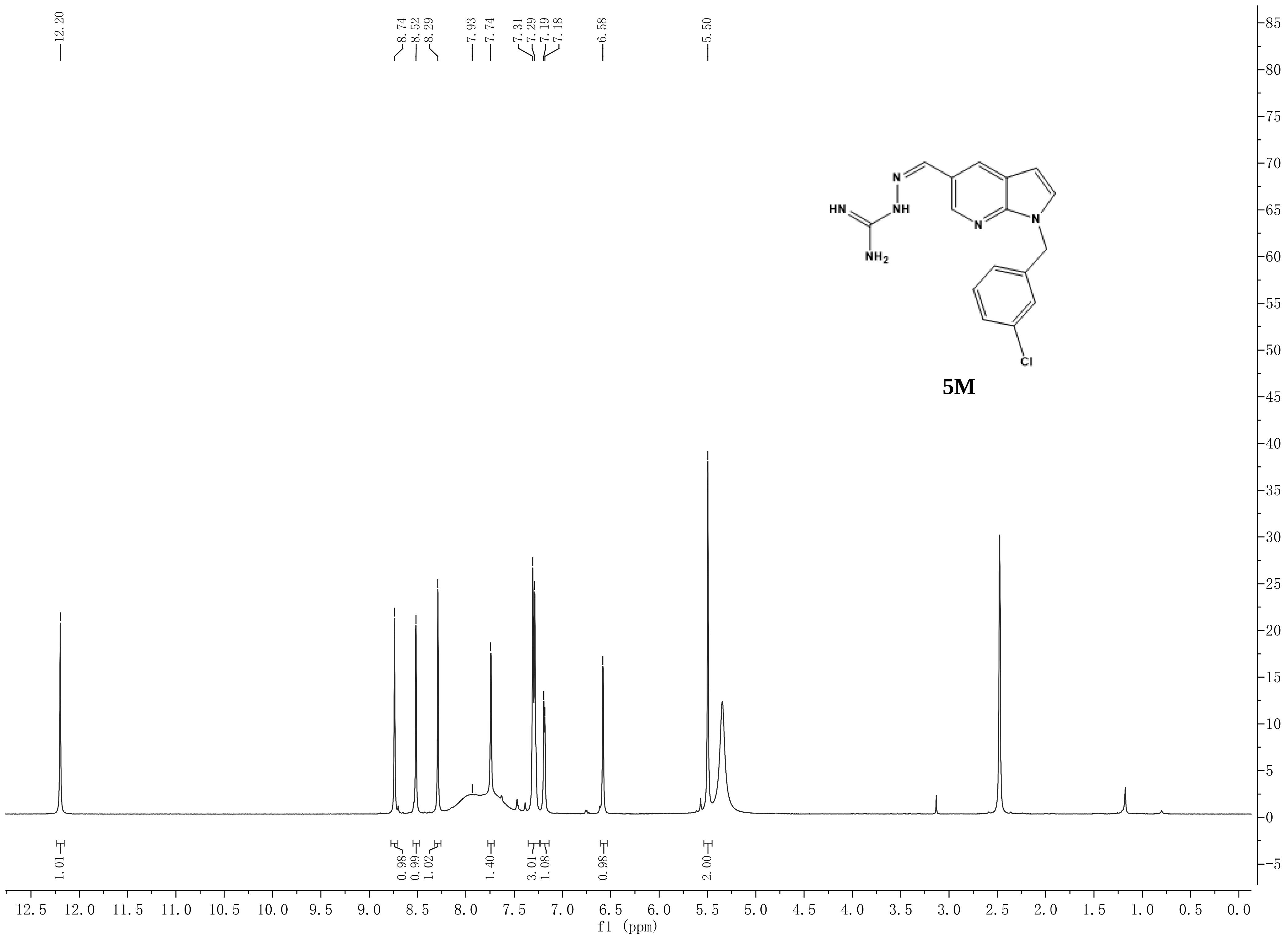

12.20
8.74
8.52
8.29
7.93
7.74
7.31
7.29
7.19
7.18
6.58
5.50
HN
N
NH
NH2
N
N
Cl
5M
1.01
0.98
0.99
1.02
1.40
3.01
1.08
0.98
2.00
f1 (ppm)

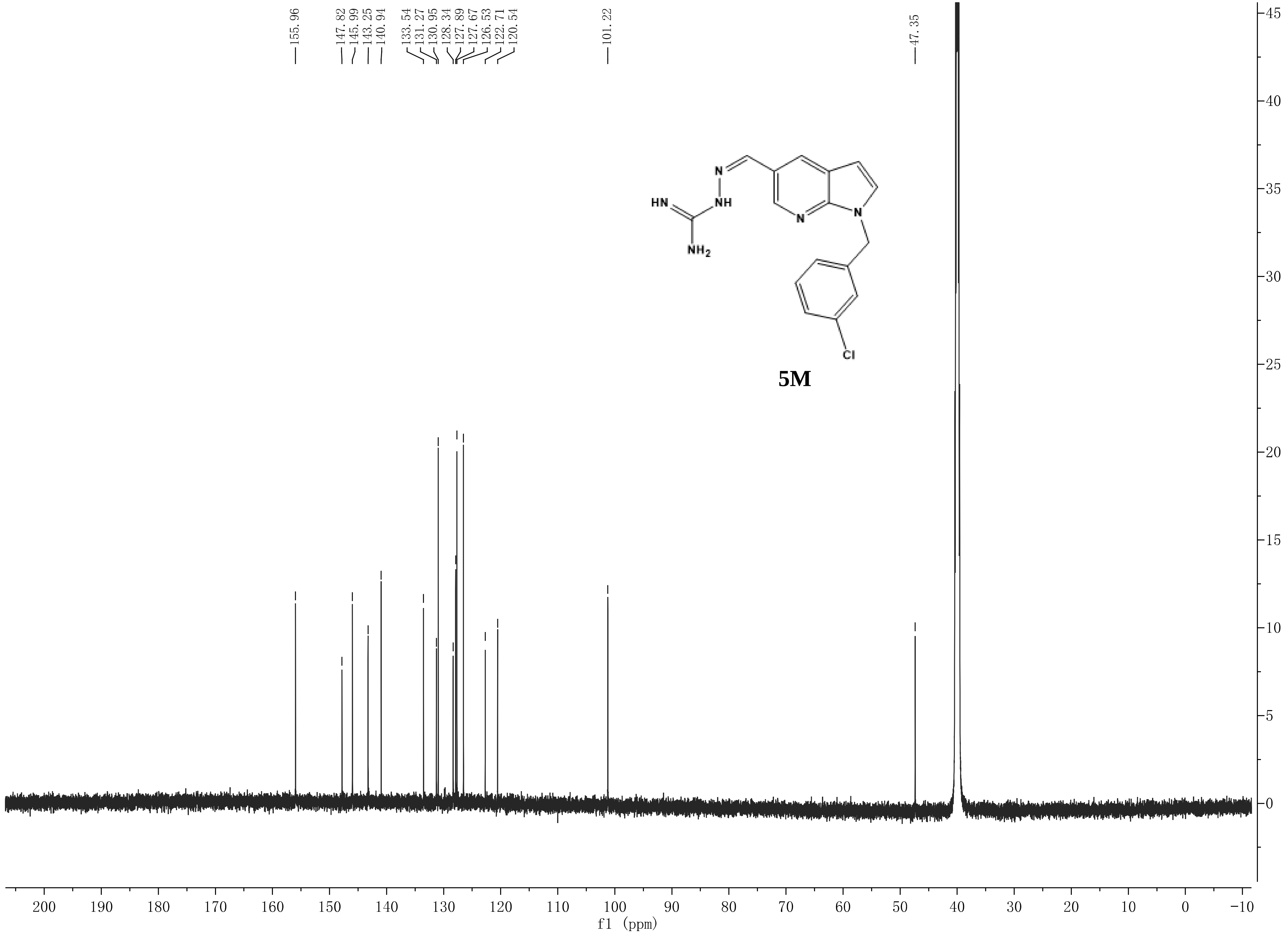
155.96
147.82
145.99
143.25
140.94
133.54
131.27
130.95
128.34
127.89
127.67
126.53
122.71
120.54
101.22
47.35
HN
N
NH
NH_2
N
N
Cl
5M
f1 (ppm)

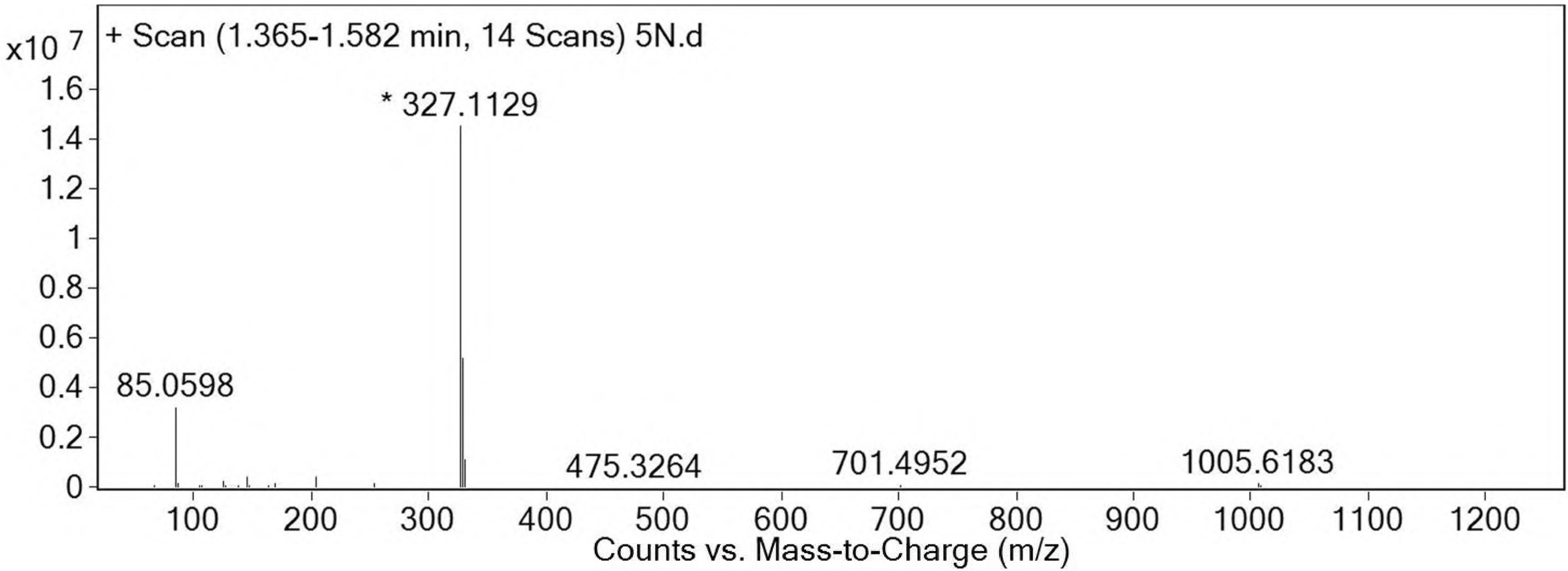

x10 7
+ Scan (1.365-1.582 min, 14 Scans) 5N.d
1.6
1.4
1.2
1
0.8
0.6
0.4
0.2
0
* 327.1129
85.0598
475.3264
701.4952
1005.6183
100
200
300
400
500
600
700
800
900
1000
1100
1200
Counts vs. Mass-to-Charge (m/z)

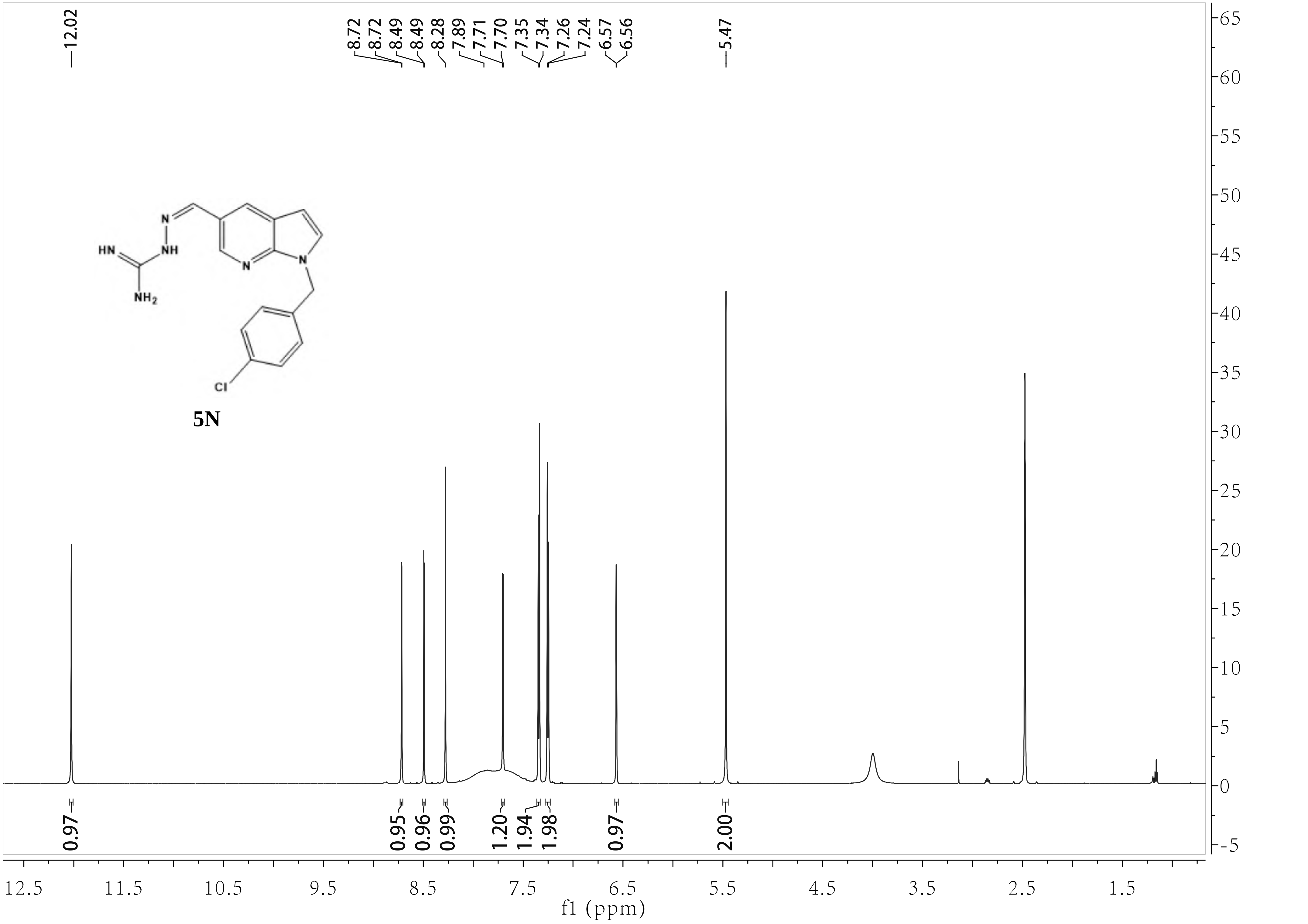

12.02
8.72
8.72
8.49
8.49
8.28
7.89
7.71
7.70
7.35
7.34
7.26
7.24
6.57
6.56
5.47
HN
NH
N
NH2
N
N
Cl
5N
0.97
0.95
0.96
0.99
1.20
1.94
1.98
0.97
2.00
f1 (ppm)

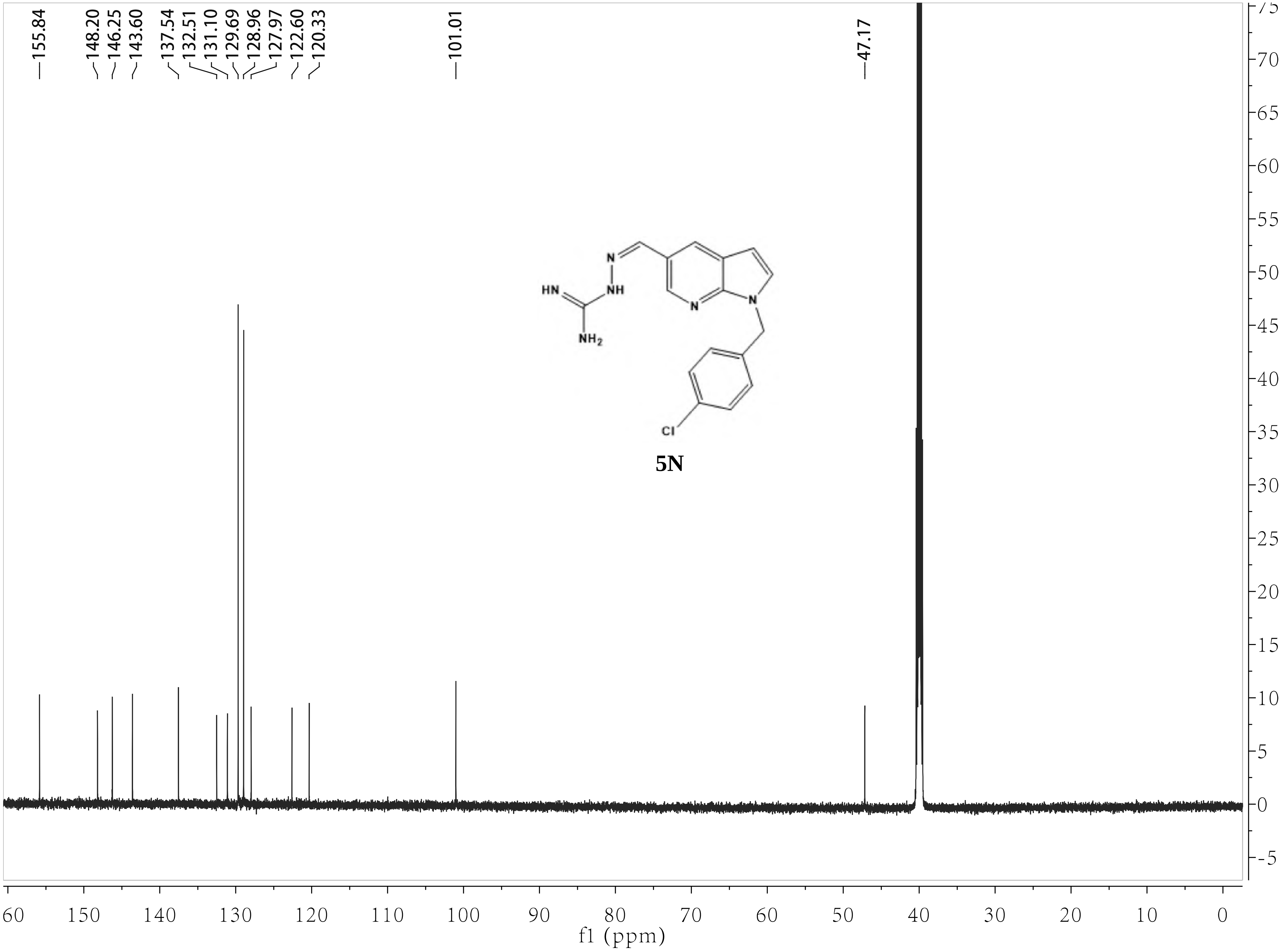
155.84
148.20
146.25
143.60
137.54
132.51
131.10
129.69
128.96
127.97
122.60
120.33
101.01
47.17
HN
NH
N
NH2
N
N
Cl
5N
f1 (ppm)

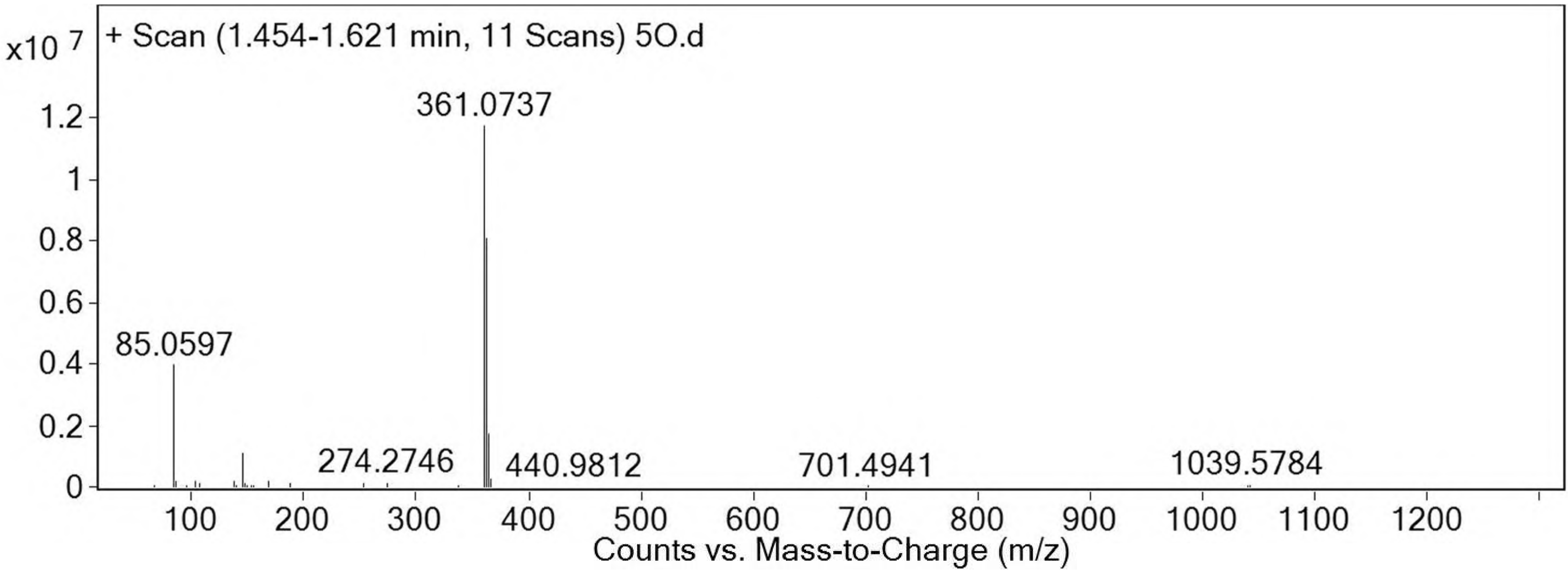
x10 7
+ Scan (1.454-1.621 min, 11 Scans) 5O.d
1.2
1
0.8
0.6
0.4
0.2
0
361.0737
85.0597
274.2746
440.9812
701.4941
1039.5784
100
200
300
400
500
600
700
800
900
1000
1100
1200
Counts vs. Mass-to-Charge (m/z)

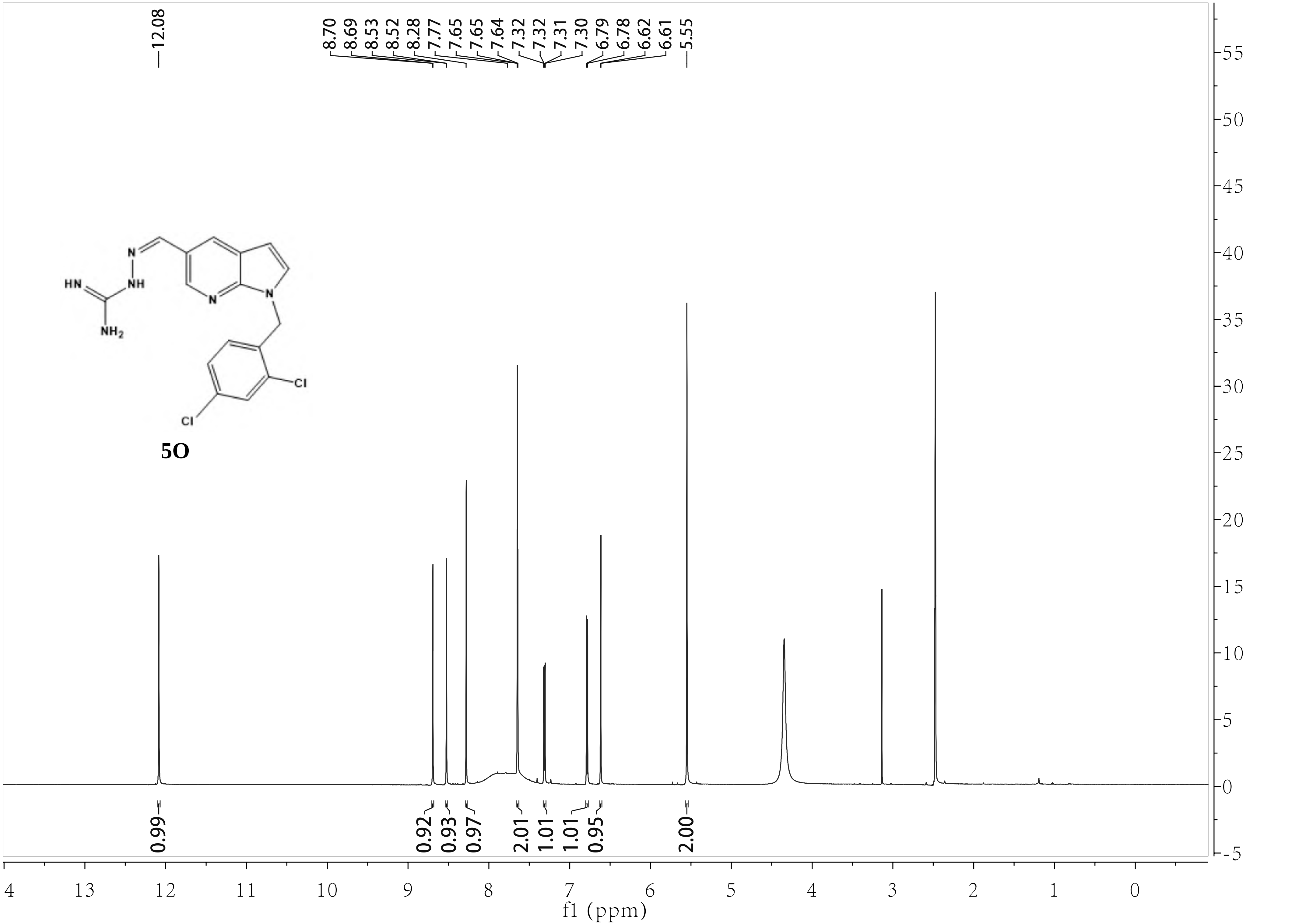
12.08
8.70 8.69 8.53 8.52 8.28 7.77 7.65 7.65 7.64 7.32 7.32 7.31 7.30 6.79 6.78 6.62 6.61 5.55
0.99 0.92 0.93 0.97 2.01 1.01 1.01 0.95 2.00
HN
NH
N
NH2
N
N
Cl
Cl
5O
f1 (ppm)

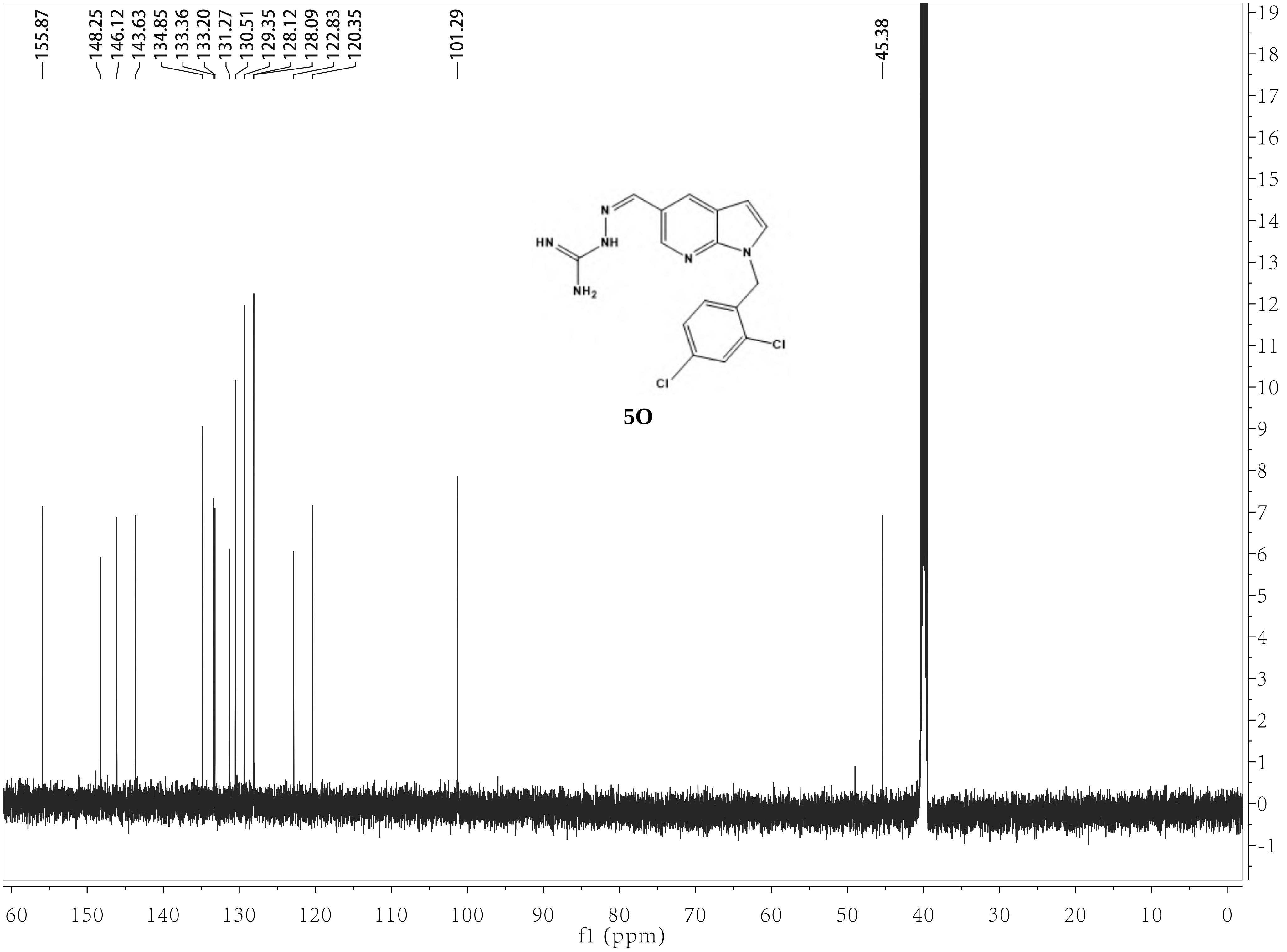
155.87
148.25
146.12
143.63
134.85
133.36
133.20
131.27
130.51
129.35
128.12
128.09
122.83
120.35
101.29
45.38
HN
NH
N
NH2
N
N
Cl
Cl
5O
f1 (ppm)

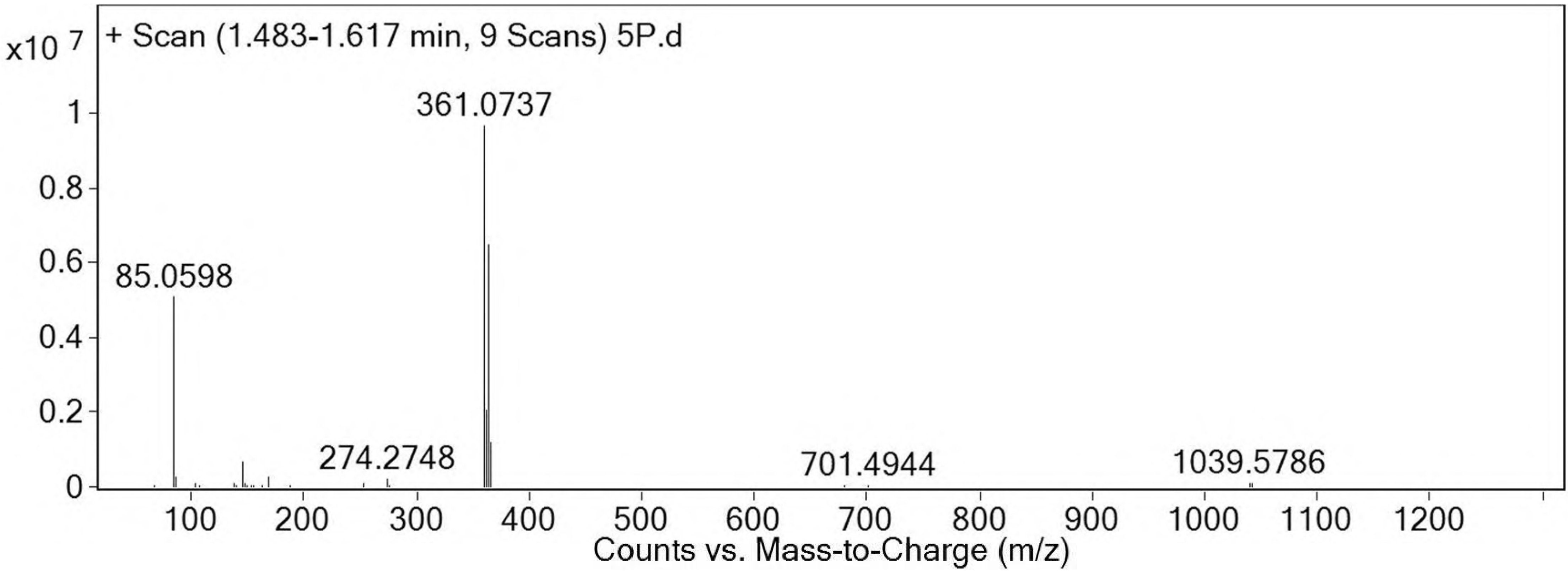
x10 7
+ Scan (1.483-1.617 min, 9 Scans) 5P.d
1
0.8
0.6
0.4
0.2
0
361.0737
85.0598
274.2748
701.4944
1039.5786
100
200
300
400
500
600
700
800
900
1000
1100
1200
Counts vs. Mass-to-Charge (m/z)

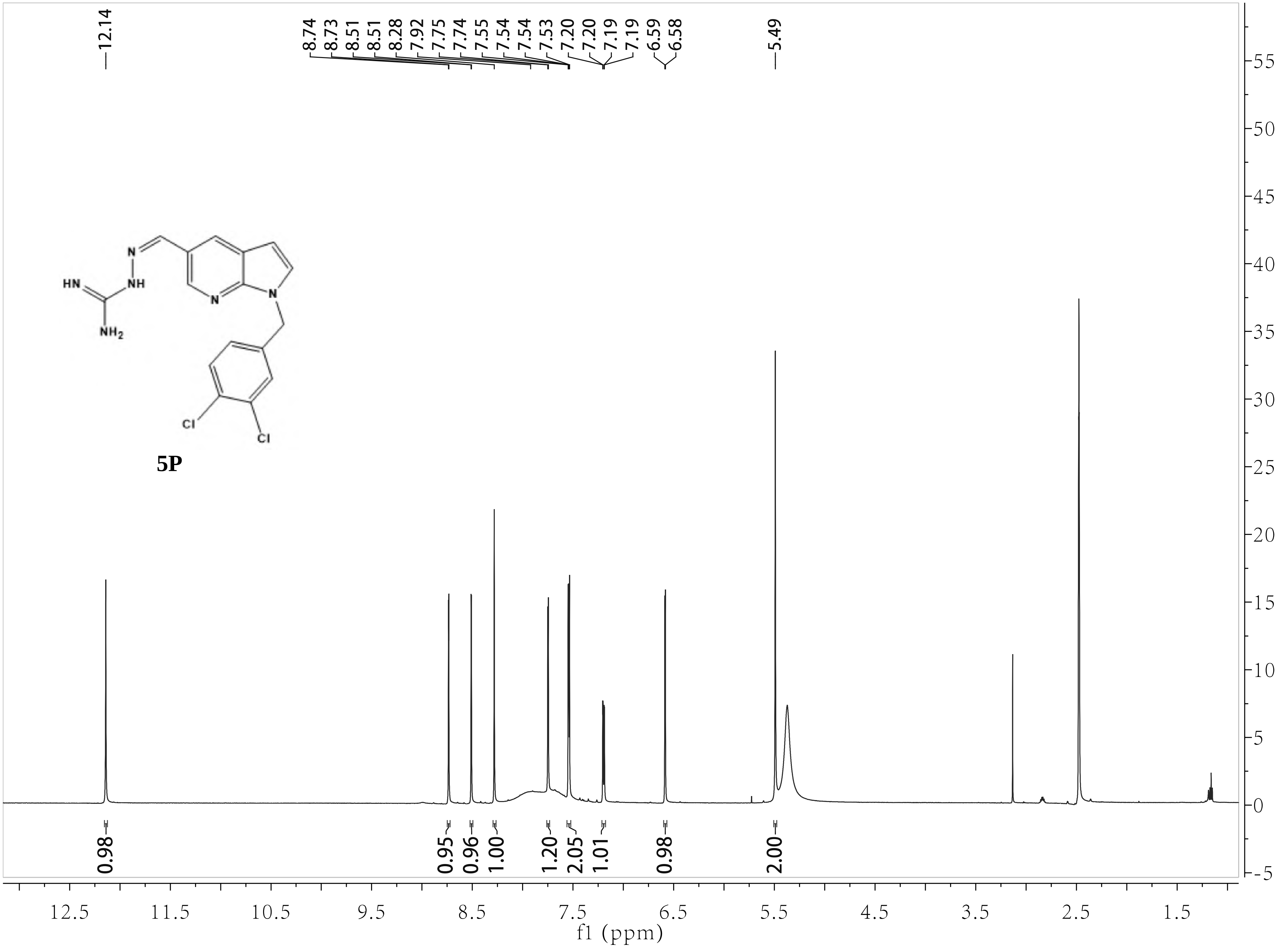
12.14
8.74
8.73
8.51
8.51
8.28
7.92
7.75
7.74
7.55
7.54
7.54
7.53
7.20
7.20
7.19
7.19
6.59
6.58
5.49
HN
N
NH
NH2
N
N
Cl
Cl
5P
0.98
0.95
0.96
1.00
1.20
2.05
1.01
0.98
2.00
12.5
11.5
10.5
9.5
8.5
7.5
6.5
5.5
4.5
3.5
2.5
1.5
f1 (ppm)
55
50
45
40
35
30
25
20
15
10
5
0
-5

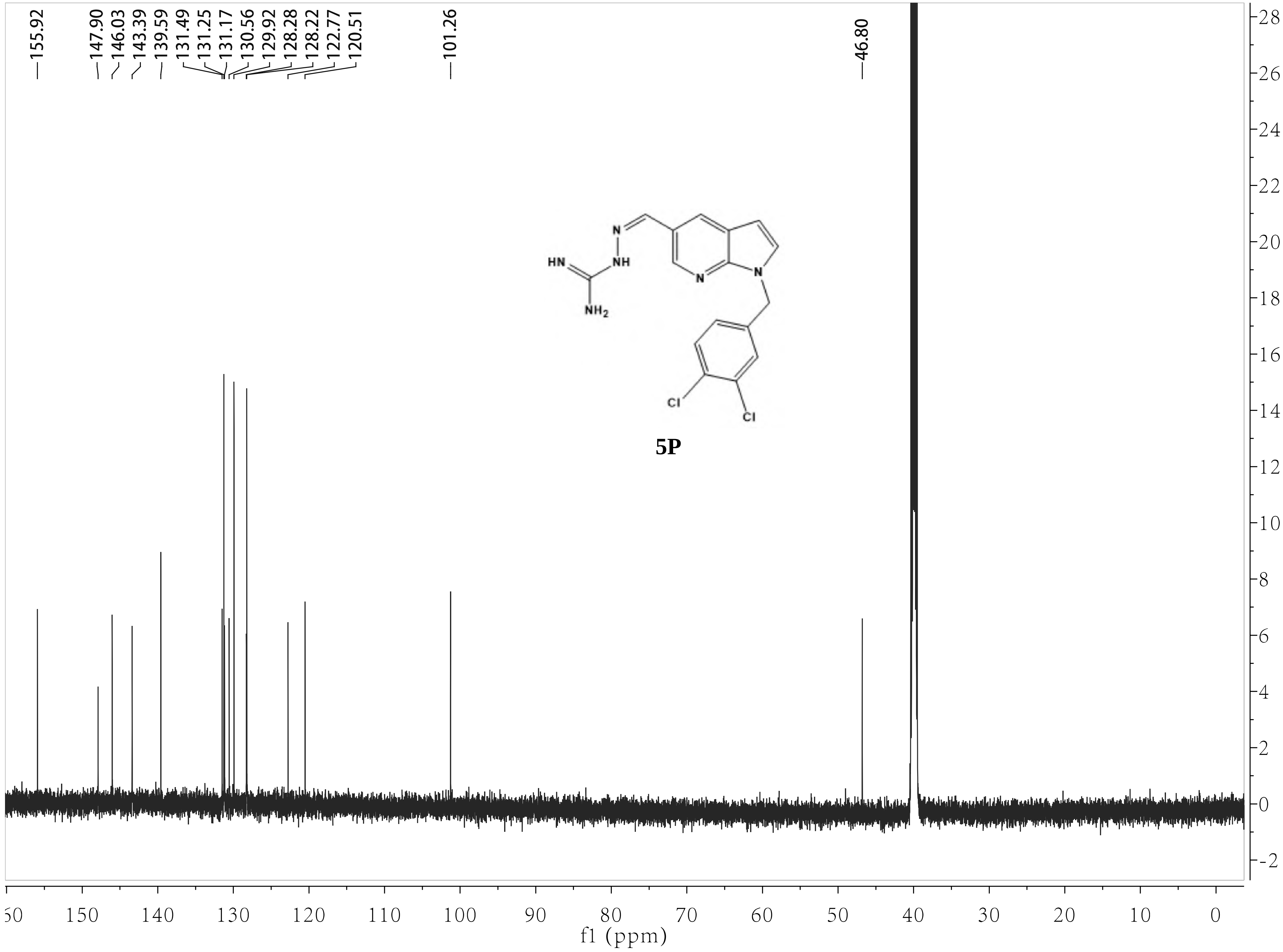
155.92
147.90
146.03
143.39
139.59
131.49
131.25
131.17
130.56
129.92
128.28
128.22
122.77
120.51
101.26
46.80
HN
N
NH
NH2
N
N
Cl
Cl
5P
28
26
24
22
20
18
16
14
12
10
8
6
4
2
0
-2
150
140
130
120
110
100
90
80
70
60
50
40
30
20
10
0
f1 (ppm)

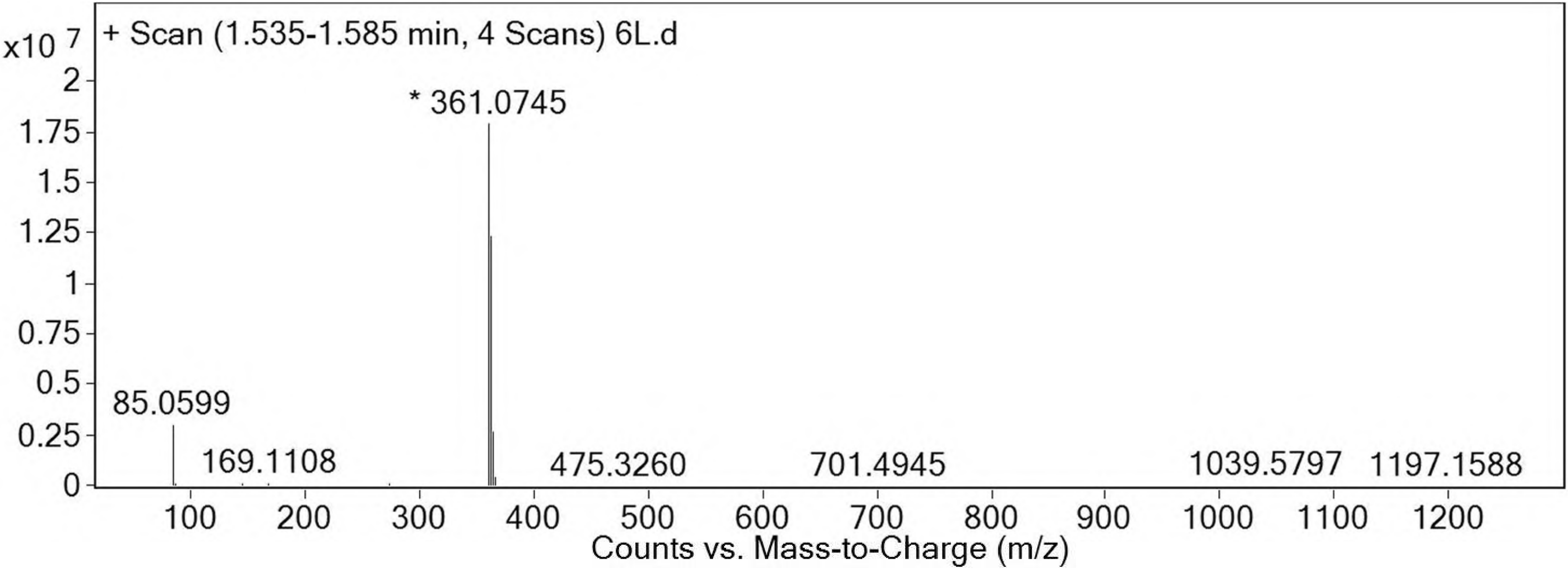

+ Scan (1.535-1.585 min, 4 Scans) 6L.d
x10 7
2
1.75
1.5
1.25
1
0.75
0.5
0.25
0
* 361.0745
85.0599
169.1108
475.3260
701.4945
1039.5797
1197.1588
100
200
300
400
500
600
700
800
900
1000
1100
1200
Counts vs. Mass-to-Charge (m/z)

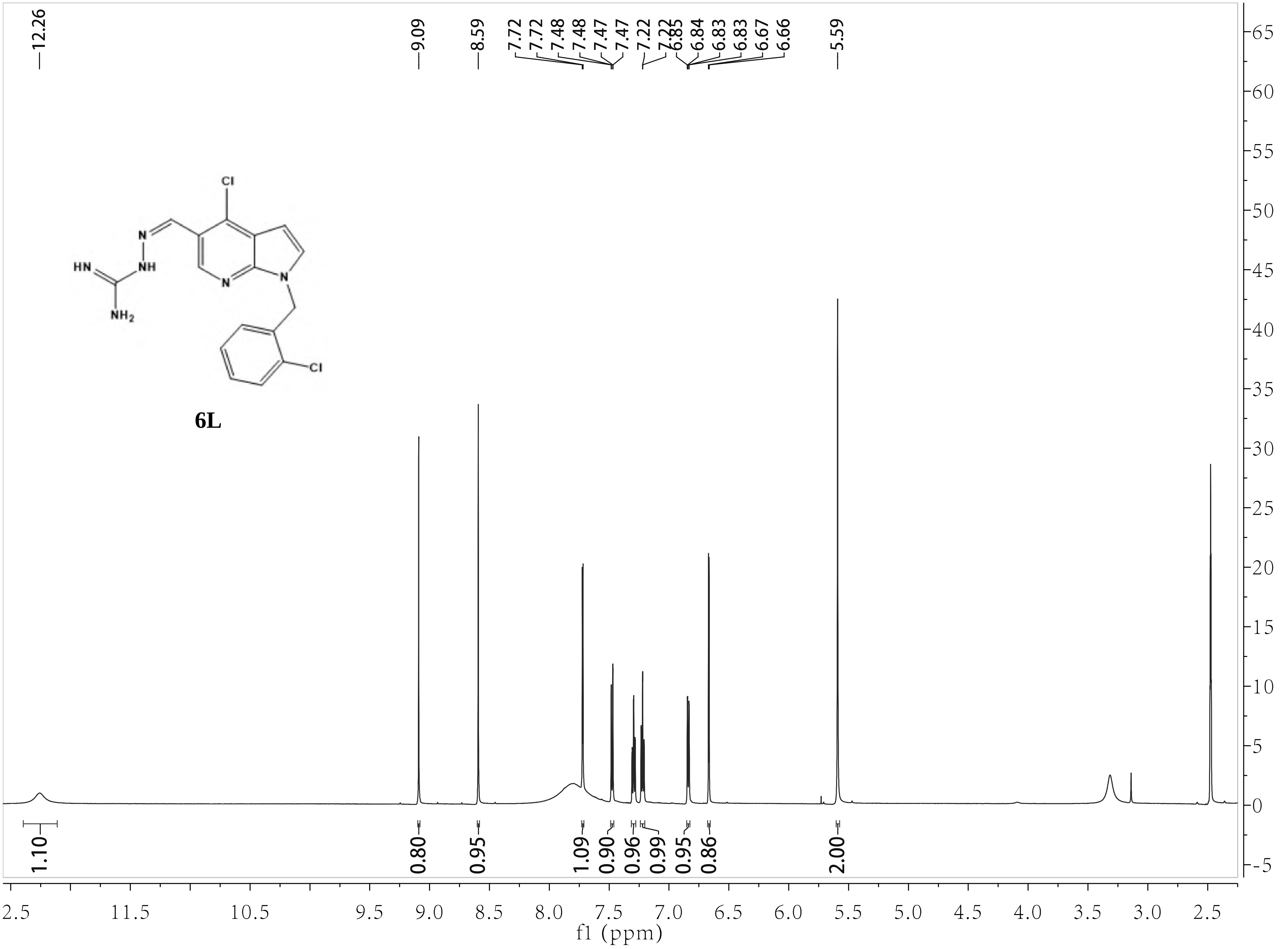
12.26
9.09
8.59
7.72
7.72
7.48
7.48
7.47
7.47
7.22
7.22
6.85
6.84
6.83
6.83
6.67
6.66
5.59
Cl
N
NH
HN
NH2
N
N
Cl
6L
1.10
0.80
0.95
1.09
0.90
0.96
0.99
0.95
0.86
2.00
f1 (ppm)

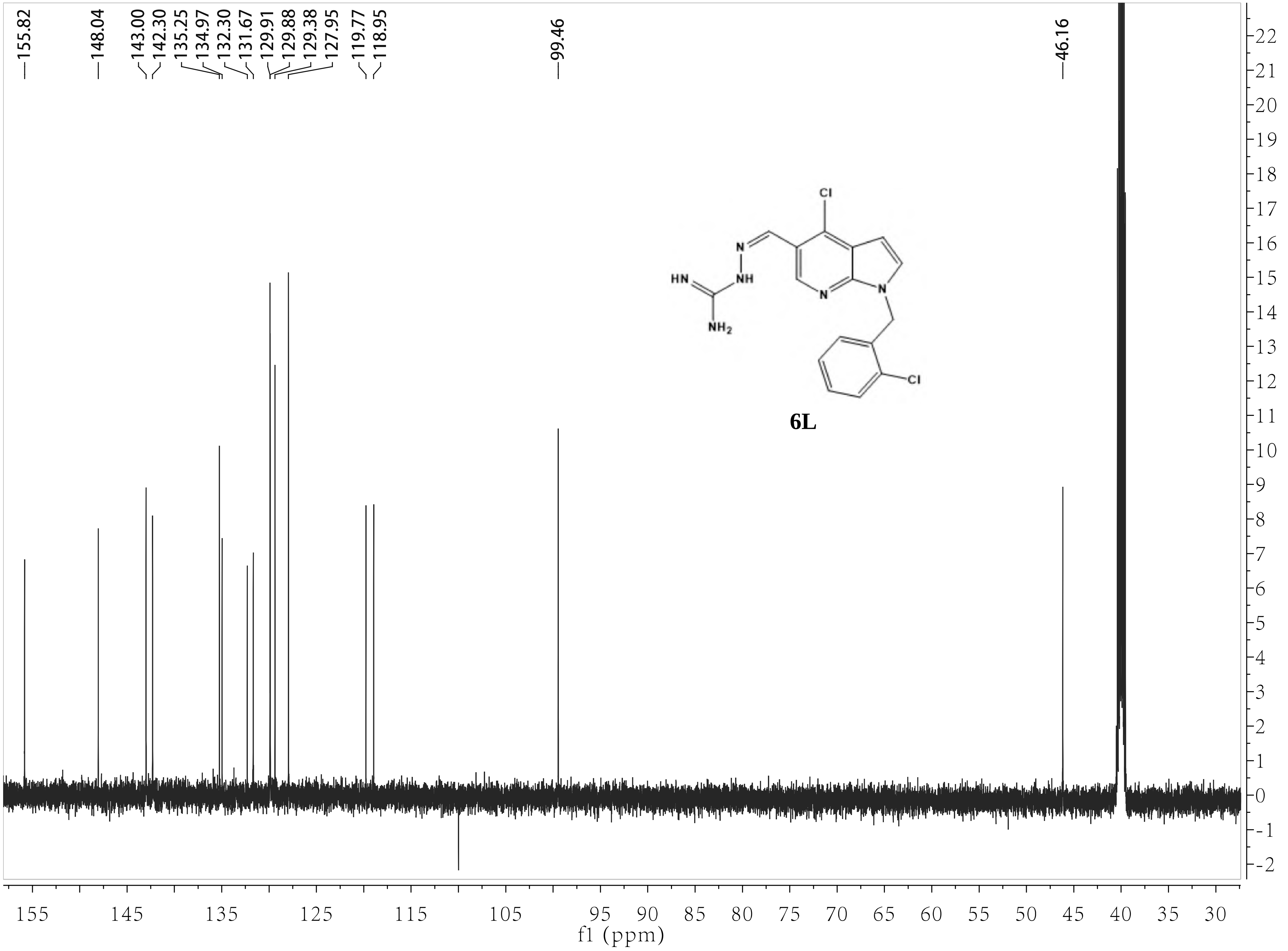

155.82
148.04
143.00
142.30
135.25
134.97
132.30
131.67
129.91
129.88
129.38
127.95
119.77
118.95
99.46
46.16
Cl
N
HN
NH
NH_2
N
N
Cl
6L
f1 (ppm)

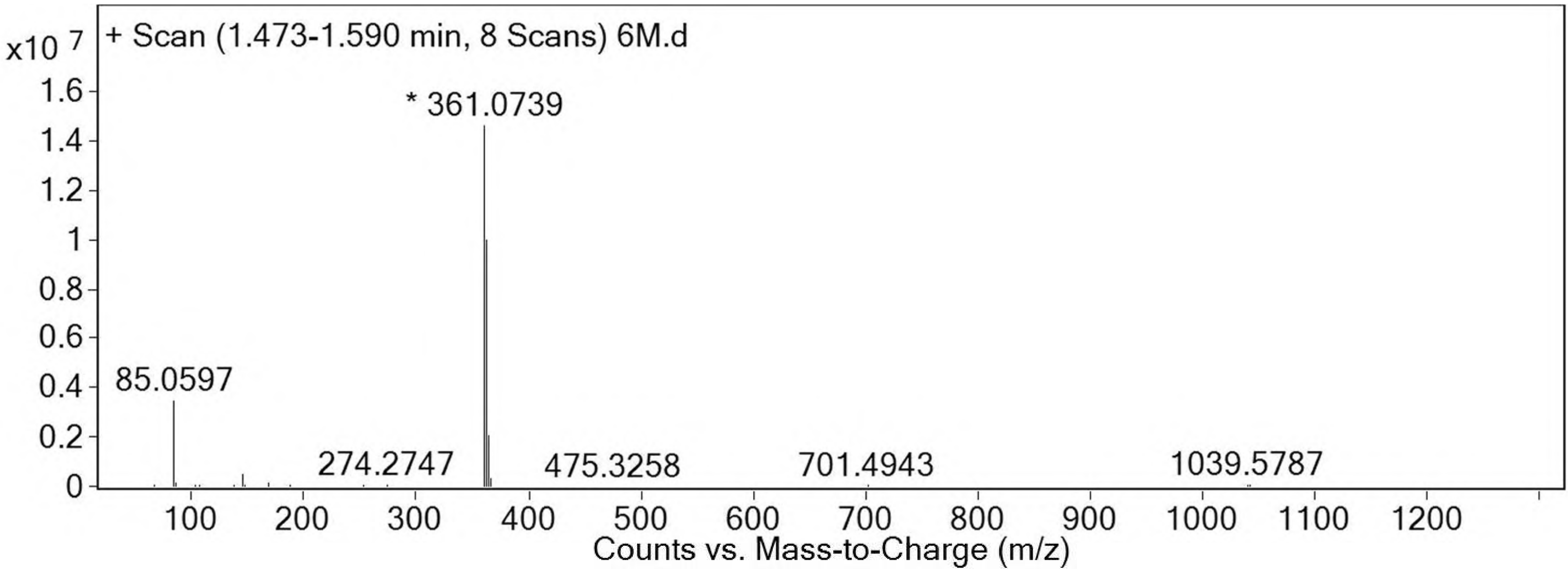

x10 7
+ Scan (1.473-1.590 min, 8 Scans) 6M.d
1.6
1.4
1.2
1
0.8
0.6
0.4
0.2
0
* 361.0739
85.0597
274.2747
475.3258
701.4943
1039.5787
100
200
300
400
500
600
700
800
900
1000
1100
1200
Counts vs. Mass-to-Charge (m/z)

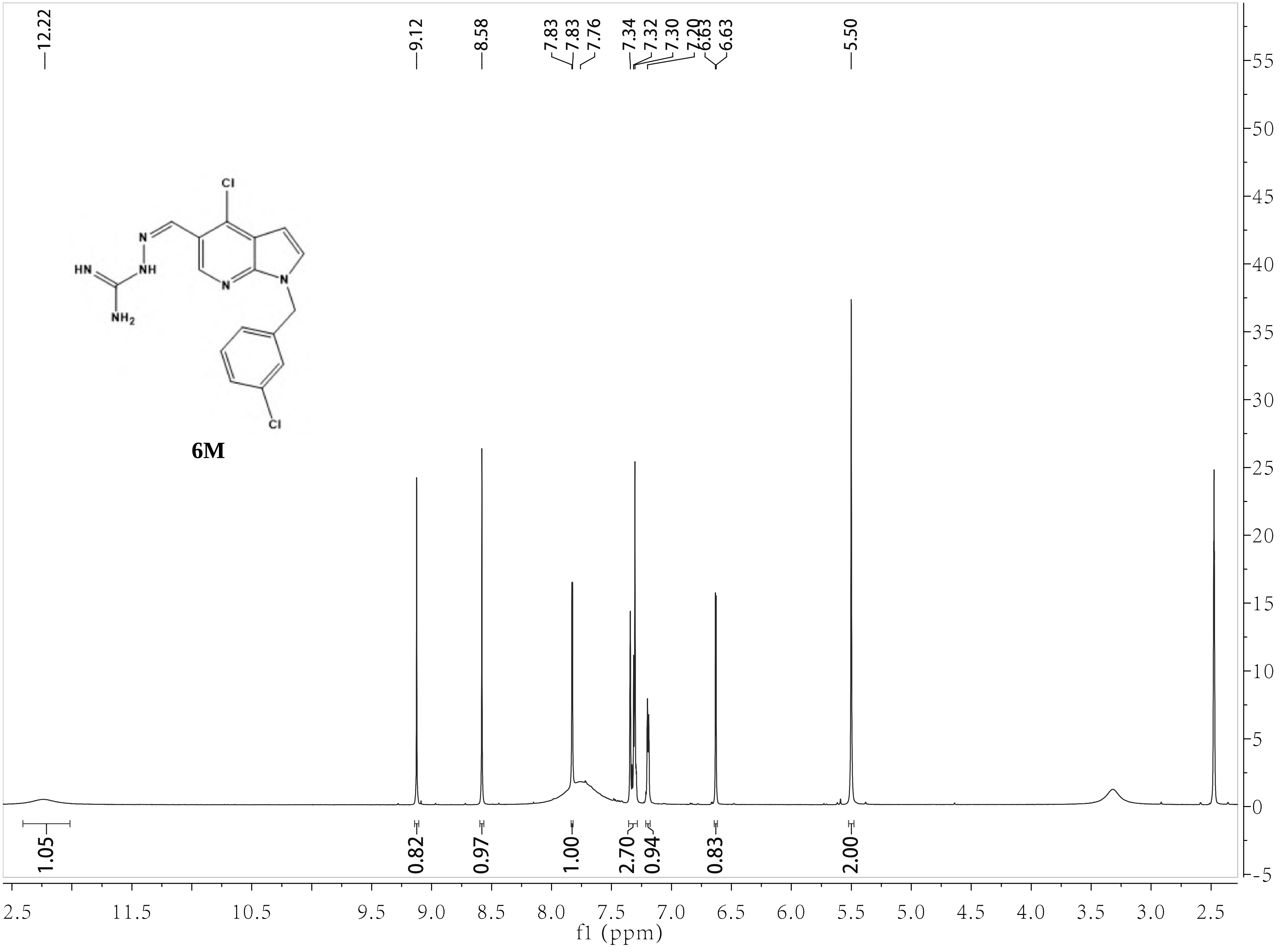
12.22
9.12
8.58
7.83
7.83
7.76
7.34
7.32
7.30
7.20
6.63
6.63
5.50
Cl
N
HN
NH
NH2
N
N
Cl
6M
1.05
0.82
0.97
1.00
2.70
0.94
0.83
2.00
2.5 11.5 10.5 9.5 9.0 8.5 8.0 7.5 7.0 6.5 6.0 5.5 5.0 4.5 4.0 3.5 3.0 2.5
f1 (ppm)
55 50 45 40 35 30 25 20 15 10 5 0 -5

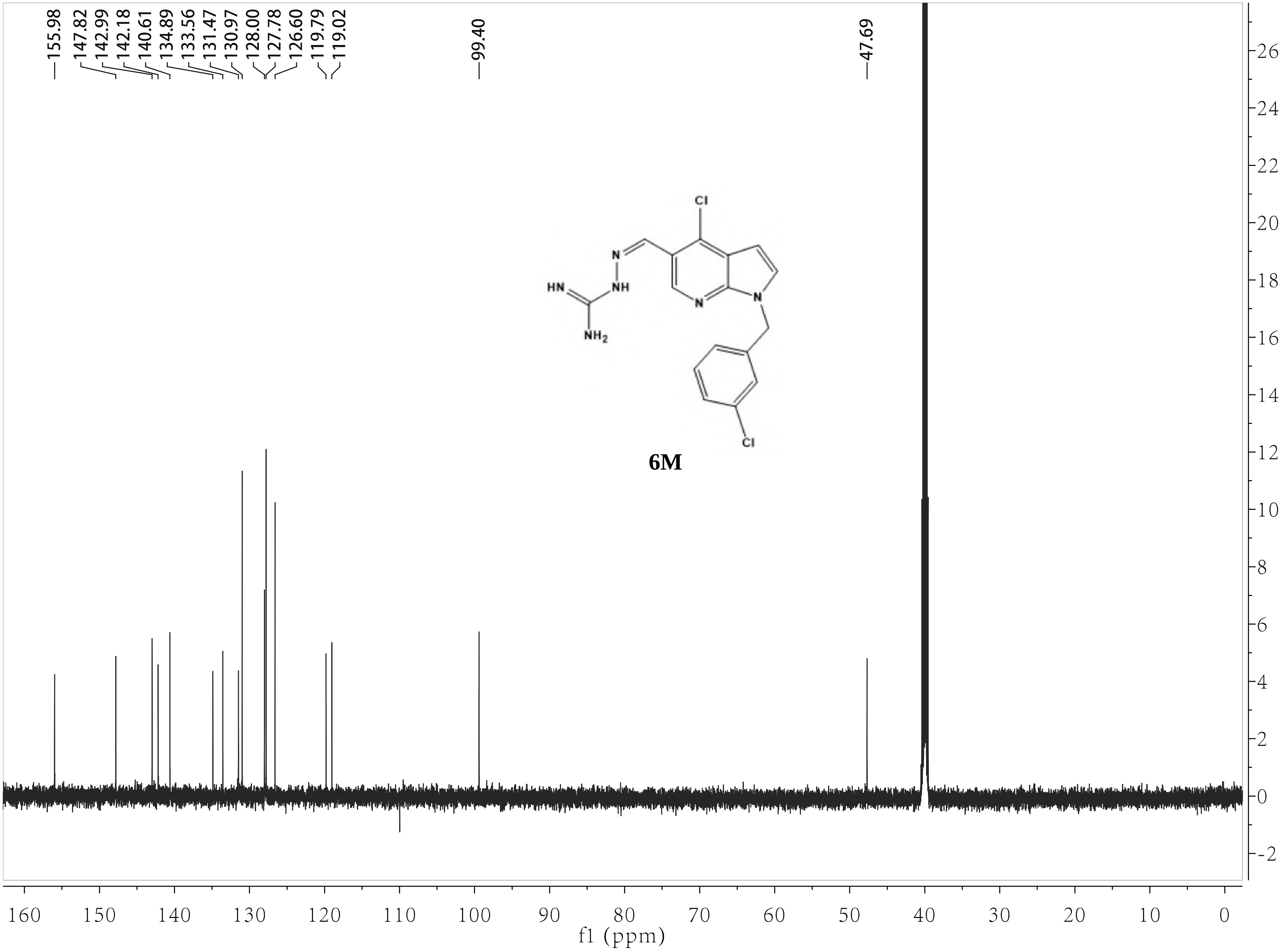
155.98
147.82
142.99
142.18
140.61
134.89
133.56
131.47
130.97
128.00
127.78
126.60
119.79
119.02
99.40
47.69
Cl
N
NH
HN
NH2
N
N
Cl
6M
160 150 140 130 120 110 100 90 80 70 60 50 40 30 20 10 0
f1 (ppm)

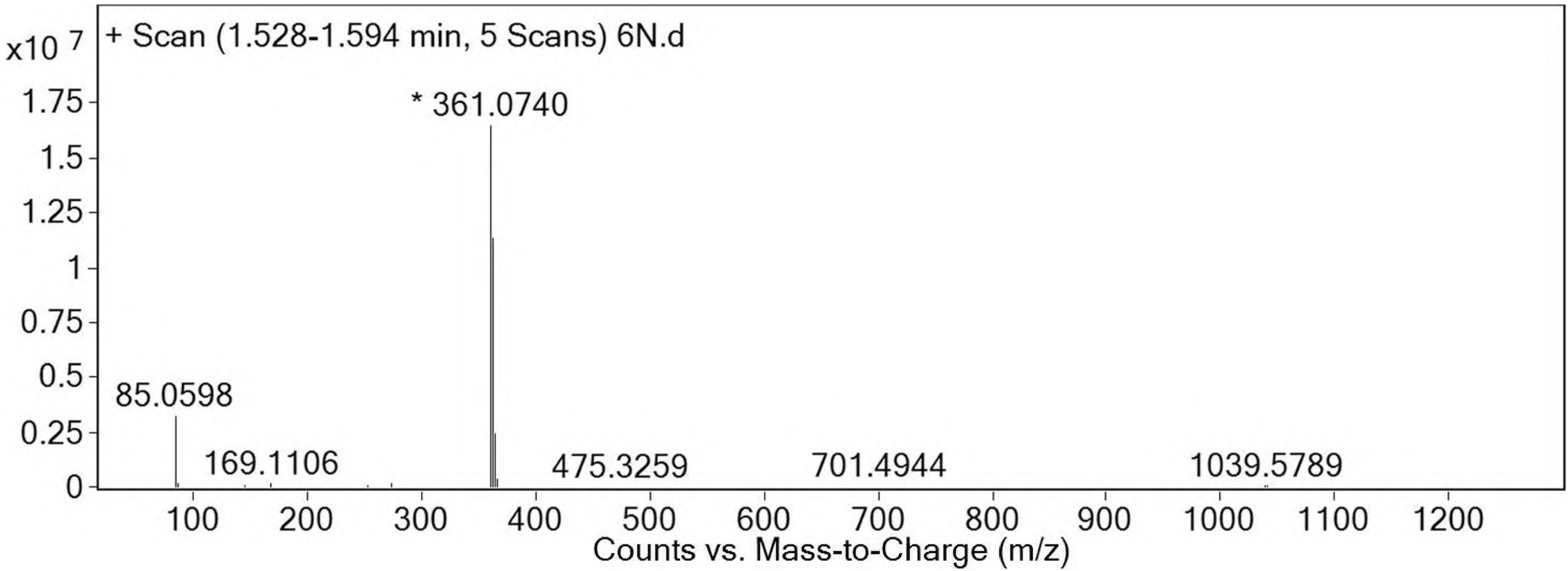
+ Scan (1.528-1.594 min, 5 Scans) 6N.d
x10 7
1.75
1.5
1.25
1
0.75
0.5
0.25
0
* 361.0740
85.0598
169.1106
475.3259
701.4944
1039.5789
100
200
300
400
500
600
700
800
900
1000
1100
1200
Counts vs. Mass-to-Charge (m/z)

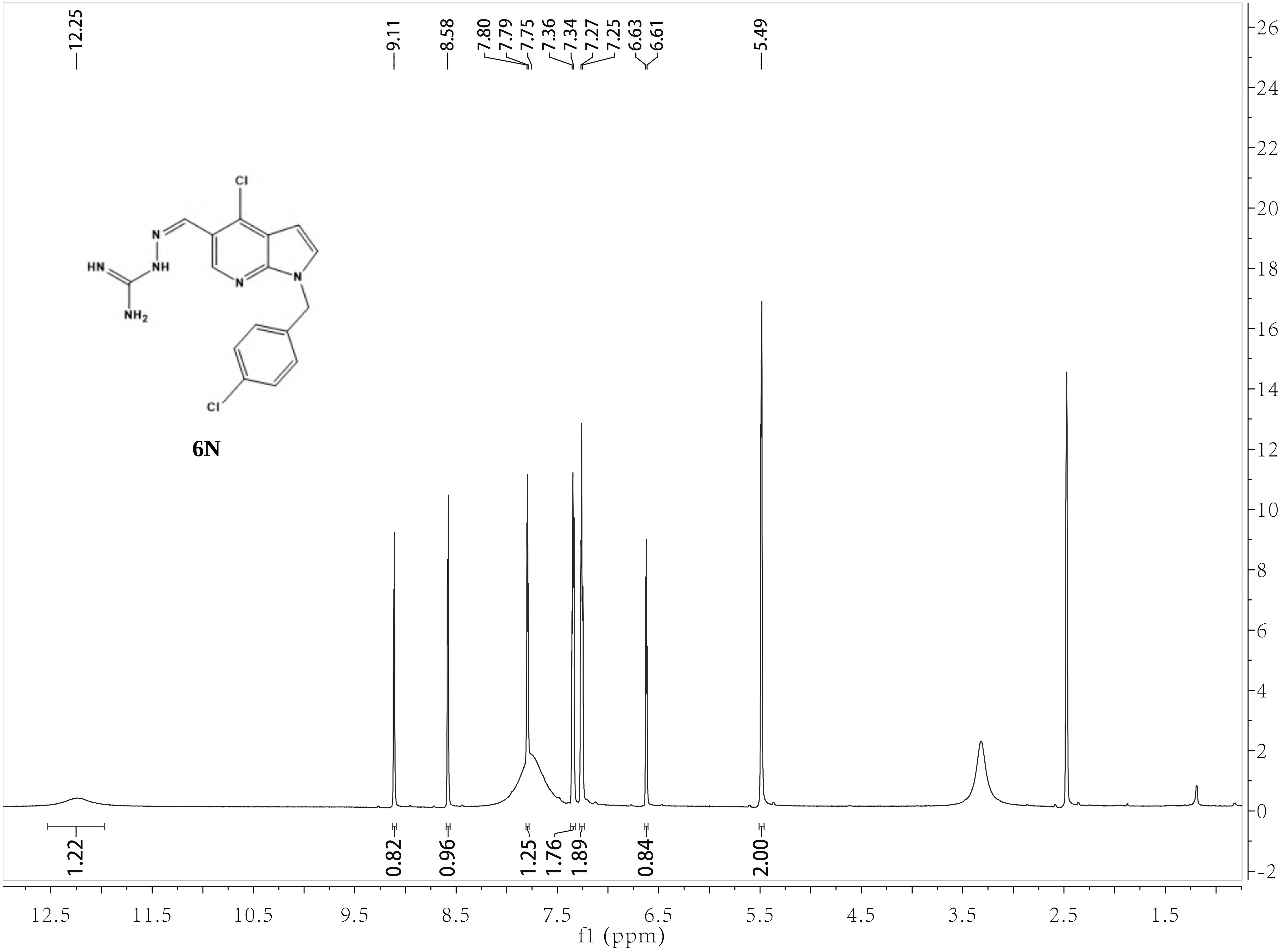
12.25
9.11
8.58
7.80
7.79
7.75
7.36
7.34
7.27
7.25
6.63
6.61
5.49
Cl
N
HN
NH
NH2
N
N
Cl
6N
1.22
0.82
0.96
1.25
1.76
1.89
0.84
2.00
12.5
11.5
10.5
9.5
8.5
7.5
6.5
5.5
4.5
3.5
2.5
1.5
f1 (ppm)
26
24
22
20
18
16
14
12
10
8
6
4
2
0
-2

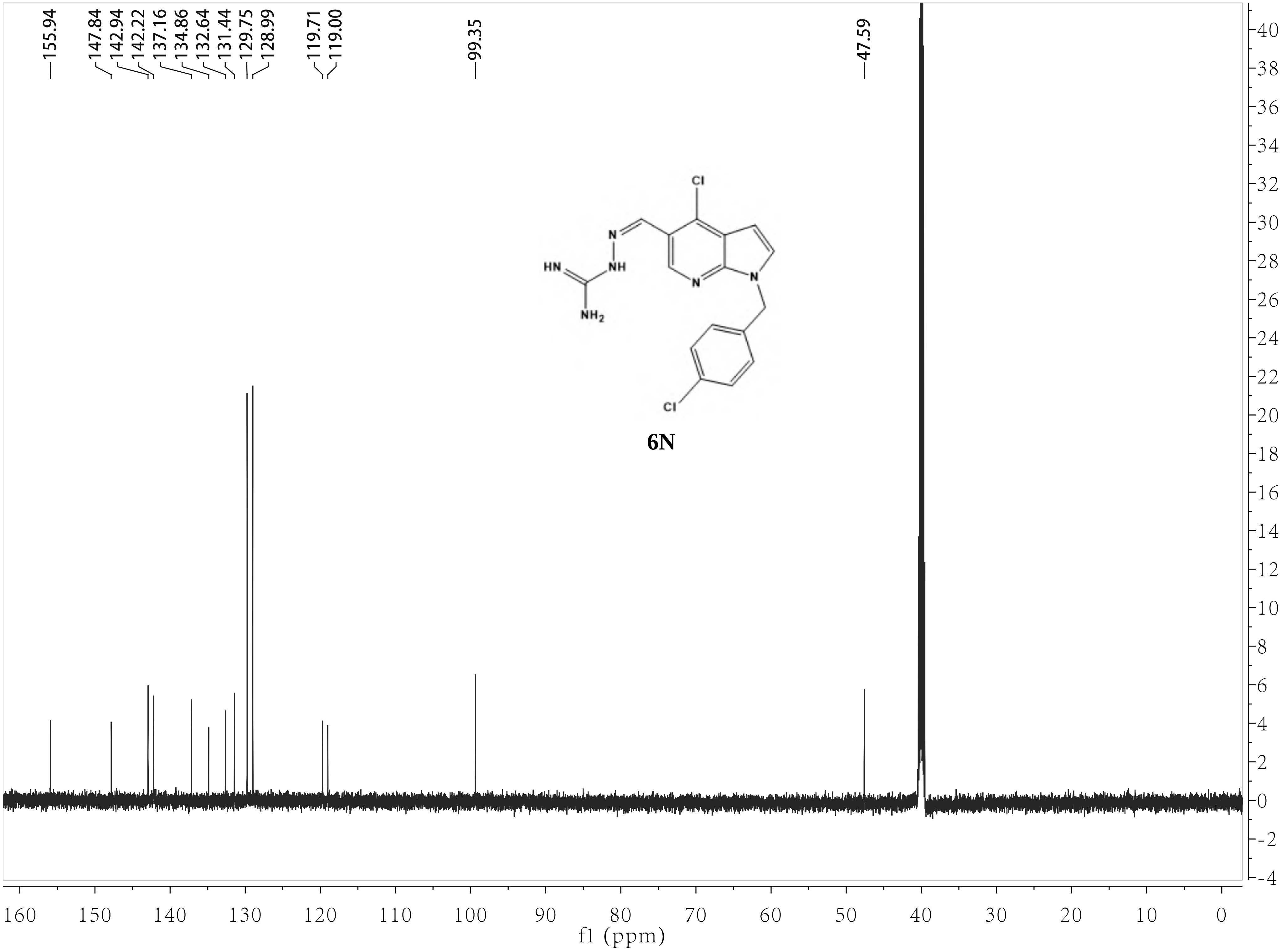

155.94
147.84
142.94
142.22
137.16
134.86
132.64
131.44
129.75
128.99
119.71
119.00
99.35
47.59
Cl
N
HN
NH
NH2
N
N
Cl
f1 (ppm)

6N

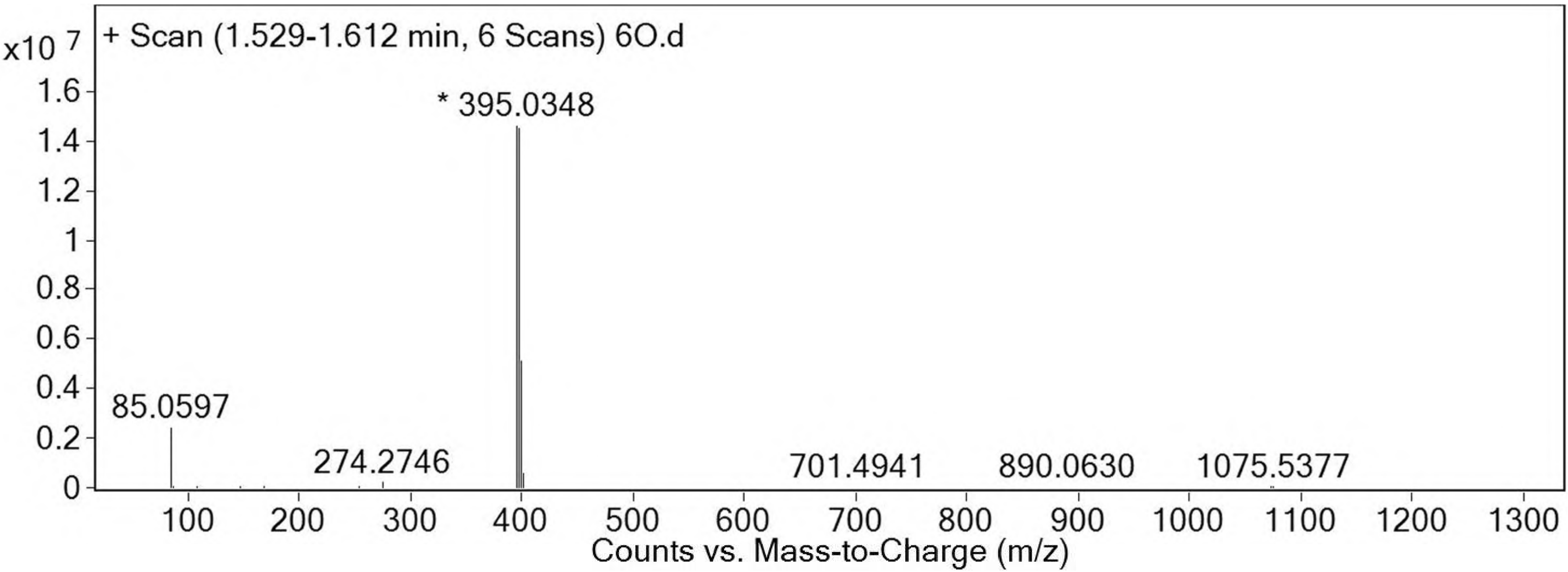
x10 7
+ Scan (1.529-1.612 min, 6 Scans) 6O.d
* 395.0348
85.0597
274.2746
701.4941
890.0630
1075.5377
0
0.2
0.4
0.6
0.8
1
1.2
1.4
1.6
100
200
300
400
500
600
700
800
900
1000
1100
1200
1300
Counts vs. Mass-to-Charge (m/z)

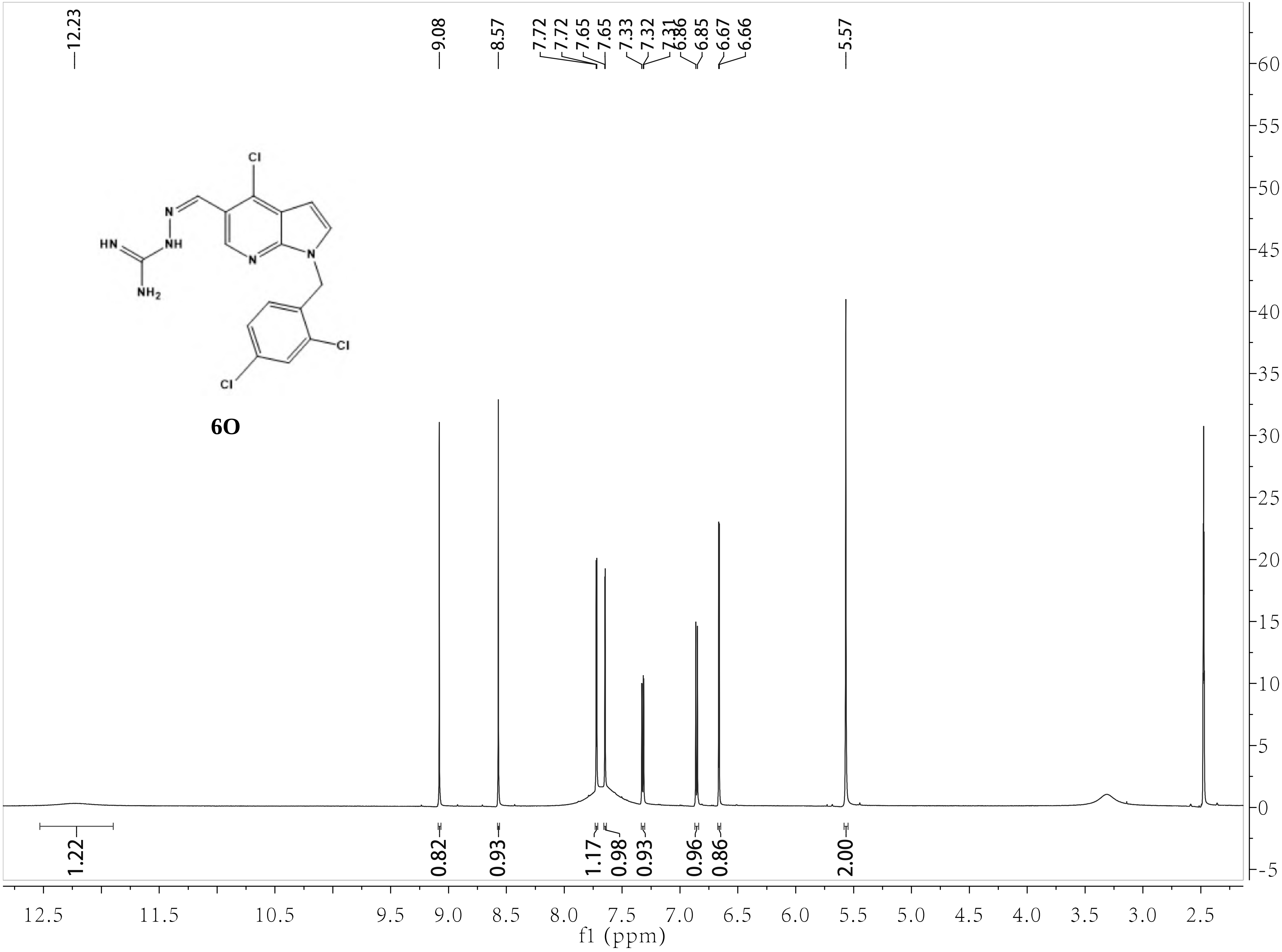

12.23
9.08
8.57
7.72
7.72
7.65
7.65
7.33
7.32
7.31
6.86
6.85
6.67
6.66
5.57
Cl
N
HN
NH
NH2
N
N
Cl
Cl
6O
1.22
0.82
0.93
1.17
0.98
0.93
0.96
0.86
2.00
12.5
11.5
10.5
9.5
9.0
8.5
8.0
7.5
7.0
6.5
6.0
5.5
5.0
4.5
4.0
3.5
3.0
2.5
f1 (ppm)
60
55
50
45
40
35
30
25
20
15
10
5
0
-5

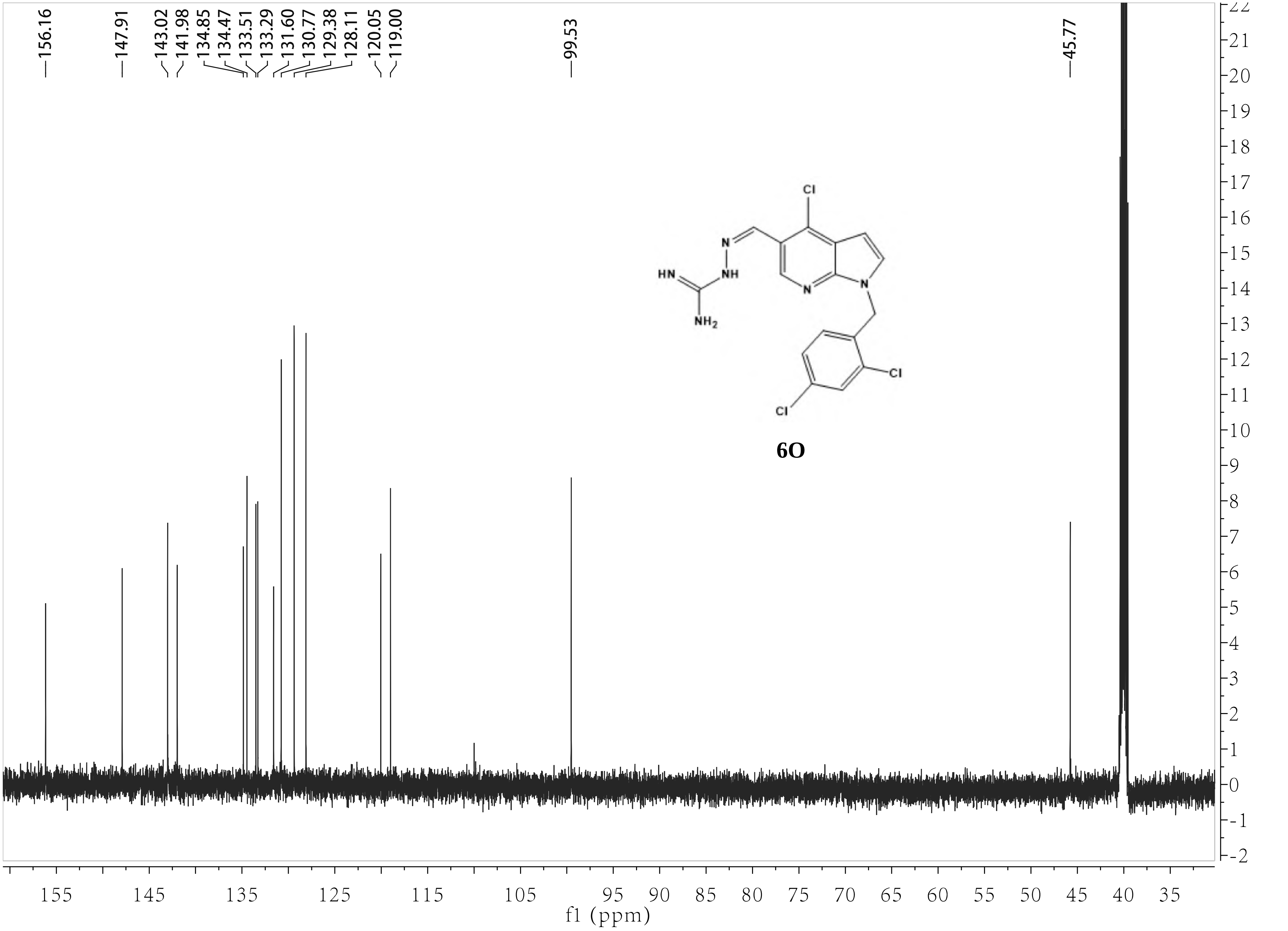
156.16
147.91
143.02
141.98
134.85
134.47
133.51
133.29
131.60
130.77
129.38
128.11
120.05
119.00
99.53
45.77
Cl
N
HN
NH
N
N
NH2
Cl
Cl
6O
f1 (ppm)

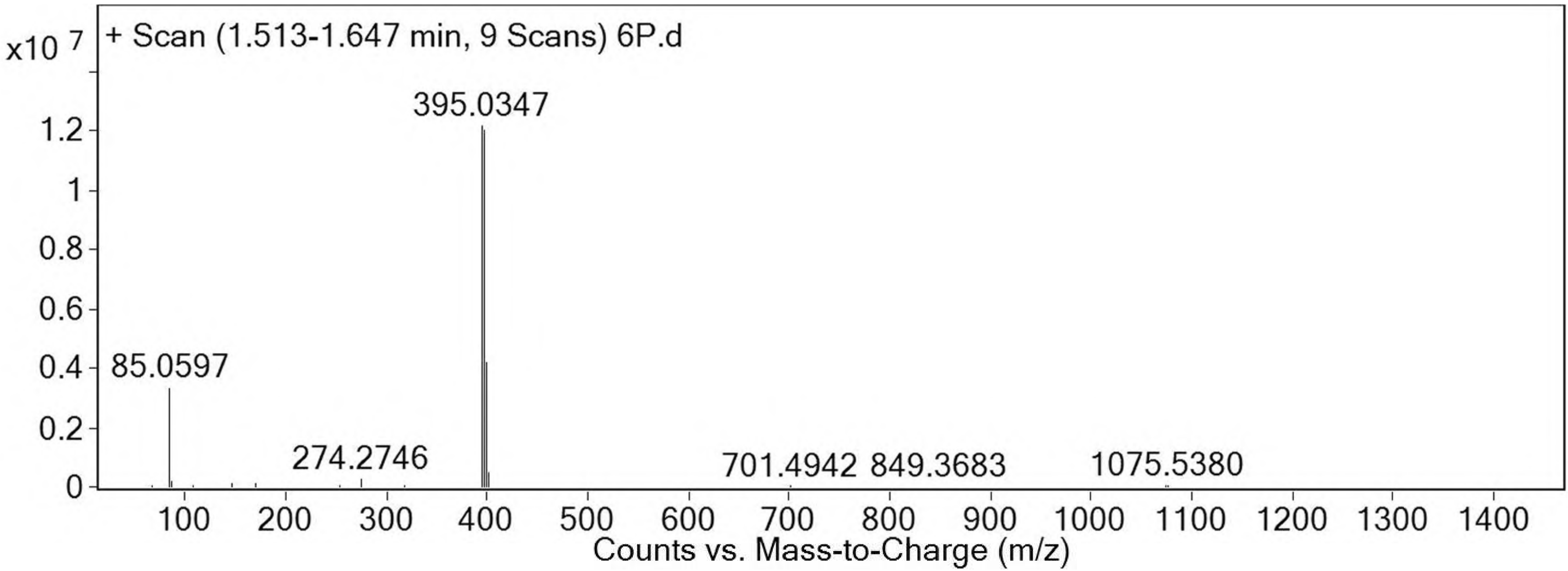

x10 7
+ Scan (1.513-1.647 min, 9 Scans) 6P.d
395.0347
85.0597
274.2746
701.4942
849.3683
1075.5380
1.2
1
0.8
0.6
0.4
0.2
0
100
200
300
400
500
600
700
800
900
1000
1100
1200
1300
1400
Counts vs. Mass-to-Charge (m/z)

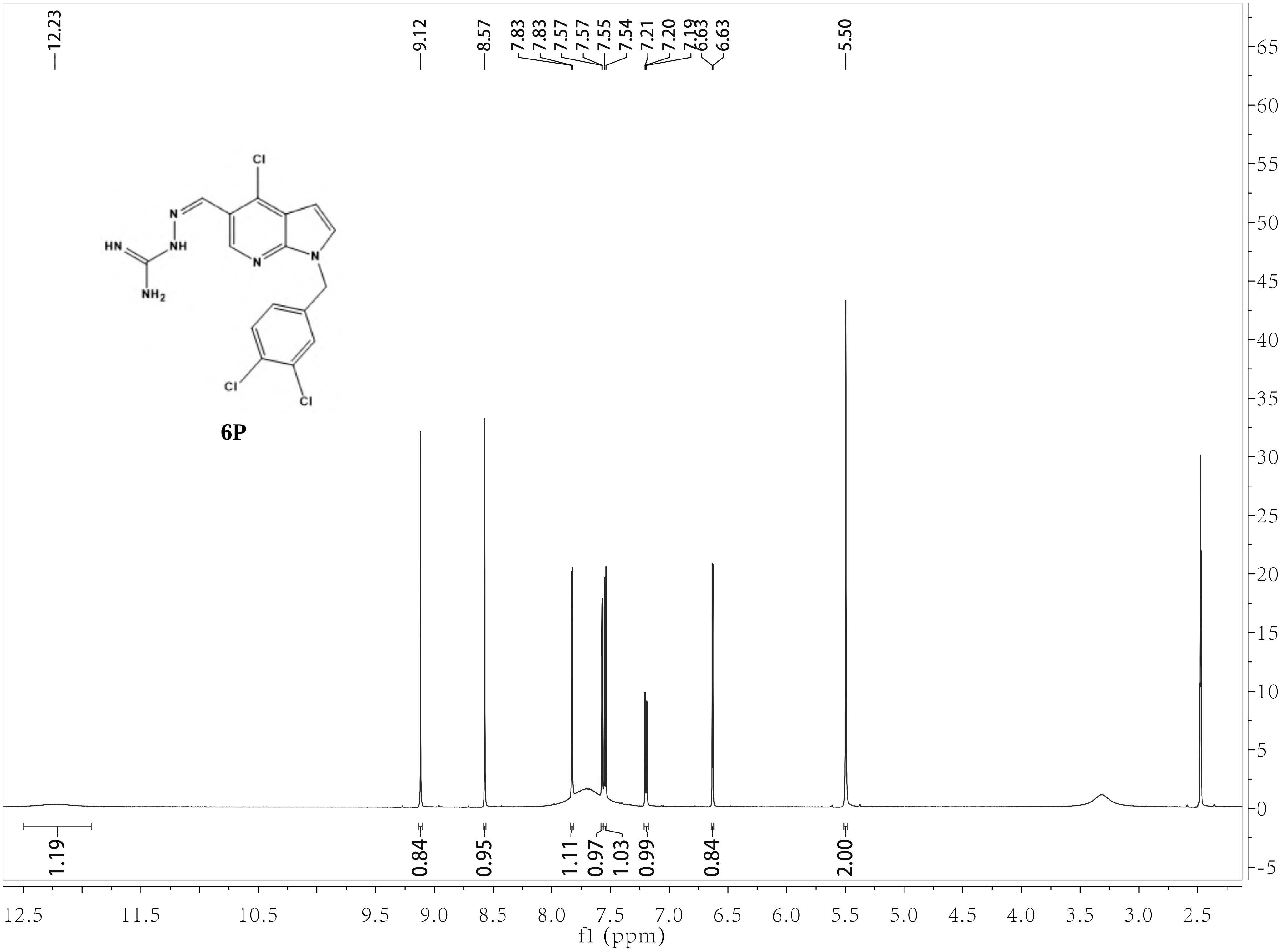
12.23
9.12
8.57
7.83
7.83
7.57
7.57
7.55
7.54
7.21
7.20
7.19
6.63
6.63
5.50
Cl
N
HN
NH
N
N
NH2
Cl
Cl
6P
1.19
0.84
0.95
1.11
0.97
1.03
0.99
0.84
2.00
f1 (ppm)
12.5
11.5
10.5
9.5
9.0
8.5
8.0
7.5
7.0
6.5
6.0
5.5
5.0
4.5
4.0
3.5
3.0
2.5
65
60
55
50
45
40
35
30
25
20
15
10
5
0
-5

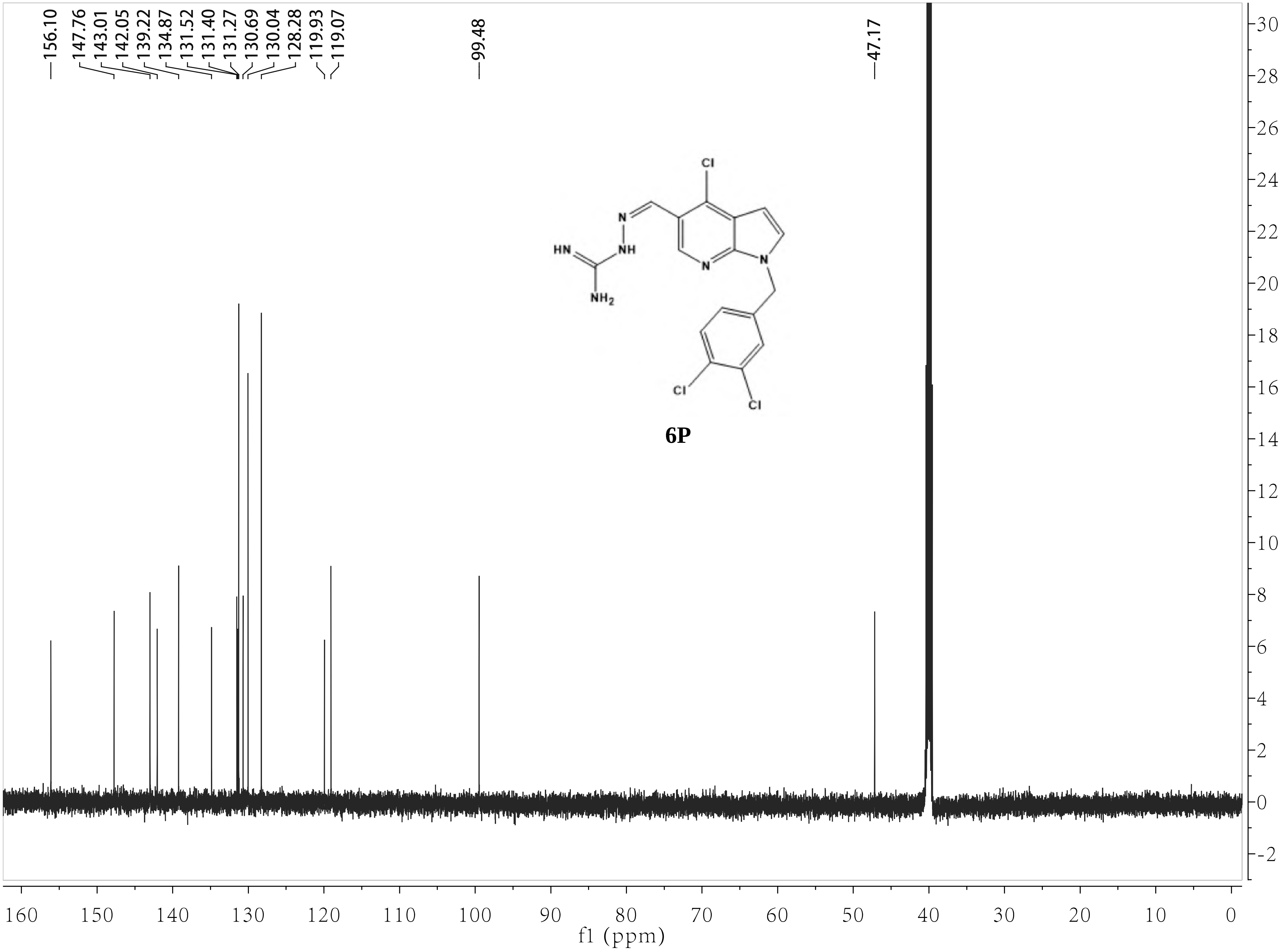
156.10
147.76
143.01
142.05
139.22
134.87
131.52
131.40
131.27
130.69
130.04
128.28
119.93
119.07
99.48
47.17
Cl
N
HN
NH
NH2
N
N
Cl
Cl
6P
f1 (ppm)
