# Supplementary material for: Synthesis, Antimicrobial Activities, and Model of Action of Indolyl Derivatives Containing Amino-Guanidinium Moieties
Source: Molecules. 2025 Feb 14;30(4):887. doi: 10.3390/molecules30040887 (PMC11858076; doi:10.3390/molecules30040887)
Supplement: Supplementary file 1 [file molecules-30-00887-s001.zip › Supplementary Materials S2.pdf]

Supplementary Information

Synthesis, Antimicrobial Activities, and Model of Action of Indolyl Derivatives Containing Amino-Guanidinium Moieties

Yu-Xi Li 1,†, Xiang Geng 1,2,†, Qi Tao 1, Ruo-Chen Hao 1, Ya-Jun Yang 1, Xi-Wang Liu 1,* and Jian-Yong Li 1,*

1 Key Lab of New Animal Drug of Gansu Province, Key Lab of Veterinary Pharmaceutical Development of Ministry of Agriculture and Rural Affairs, Lanzhou Institute of Husbandry and Pharmaceutical Sciences of Chi-nese Academy of Agricultural Sciences, Lanzhou 730050, China

2 School of Health Nursing, Fuyang Vocational Technical College, Fuyang 236000, China

* Correspondence: xiwangliu@126.com (X.-W.L.); lijy1971@163.com (J.-Y.L.)

† The authors contributed equally to this work.

Supplemental Table of Contents

HPLC profiles of all compounds

The purity of compounds was determined by analytical HPLC (Agilent Technologies 1290 Infinity) using a Agilent, 1.8 μm, Eclipse plus C18, 50 mm×3.0 mm and flow rate of 0.3 mL/min. Isocratic conditions were the following: solvent A (0.1% formic acid in water) and solvent B (0.1% formic acid in acetonitrile), 35% A, 65% B, stop time 5 min, UV detection at 280 nm. All the tested compounds were obtained with ≥95% purity by HPLC.

Qualitative Analysis Report

**Data Filename**	3A.d	**Sample Name**	3A
**Sample Type**	Sample	**Position**	P1-A2
**Instrument Name**	Instrument 1	**User Name**	
**Acq Method**	20241027 scan +.m	**Acquired Time**	10/28/2024 2:19:24 AM
**IRM Calibration Status**	Success	**DA Method**	01.m
**Comment**			

Sample Group **Info.**

Acquisition SW 6200 series TOF/6500 series
Version Q-TOF B.05.01 (B5125.1)

User Chromatograms

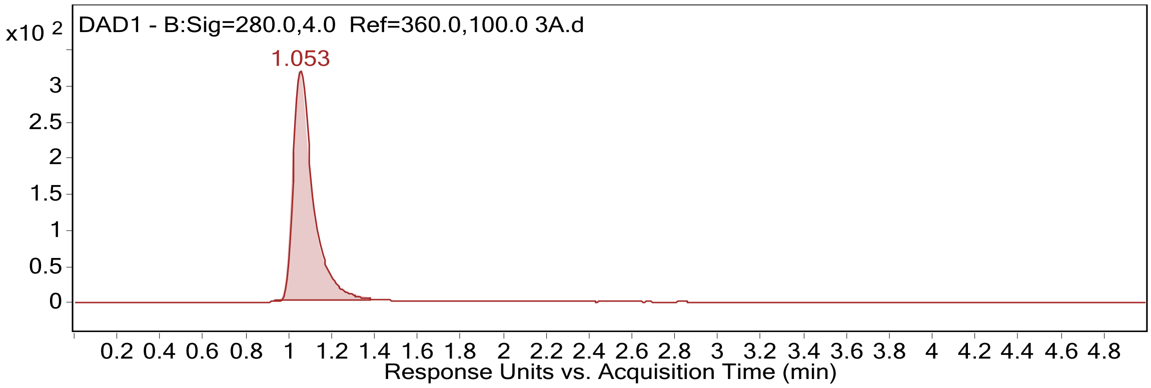

Integration Peak List

Peak	Start	RT	End	Height	Area	Area %
1	0.933	1.053	1.38	318.93	2110.48	100

--- End Of Report ---

Qualitative Analysis Report

**Data Filename**	3B.d	**Sample Name**	3B
**Sample Type**	Sample	**Position**	P1-A3
**Instrument Name**	Instrument 1	**User Name**	
**Acq Method**	20241027 scan +.m	**Acquired Time**	10/28/2024 2:25:21 AM
**IRM Calibration Status**	Success	**DA Method**	01.m
**Comment**			

Sample Group **Info.**

Acquisition SW Version 6200 series TOF/6500 series Q-TOF B.05.01 (B5125.1)

User Chromatograms

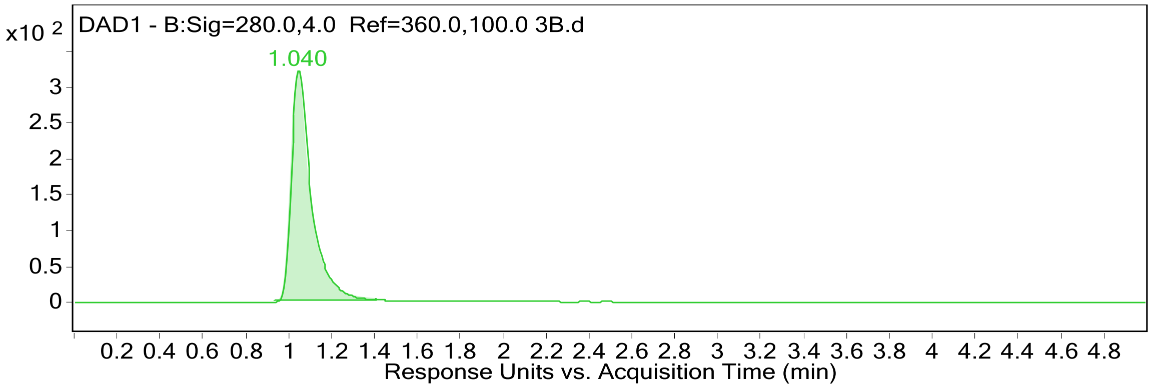

Integration Peak List

Peak	Start	RT	End	Height	Area	Area %
1	0.933	1.04	1.407	320.83	2126.15	100

--- End Of Report ---

Qualitative Analysis Report

**Data Filename**	3C.d	**Sample Name**	3C
**Sample Type**	Sample	**Position**	P1-A4
**Instrument Name**	Instrument 1	**User Name**	
**Acq Method**	20241027 scan +.m	**Acquired Time**	10/28/2024 2:31:19 AM
**IRM Calibration Status**	Success	**DA Method**	01.m
**Comment**			

Sample Group **Info.**

Acquisition SW 6200 series TOF/6500 series
Version Q-TOF B.05.01 (B5125.1)

User Chromatograms

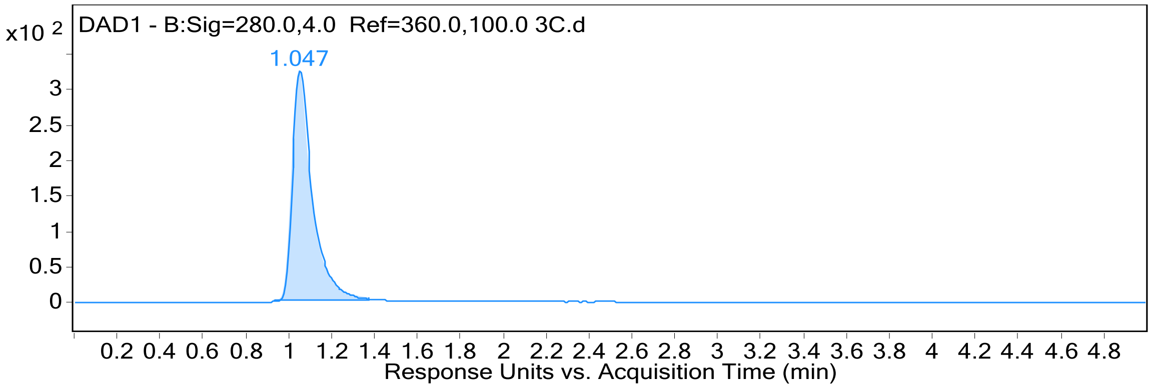

Integration Peak List

Peak	Start	RT	End	Height	Area	Area %
1	0.933	1.047	1.373	324.58	2147.76	100

--- End Of Report ---

Qualitative Analysis Report

**Data Filename**	3D.d	**Sample Name**	3D
**Sample Type**	Sample	**Position**	P1-A5
**Instrument Name**	Instrument 1	**User Name**	
**Acq Method**	20241027 scan +.m	**Acquired Time**	10/28/2024 2:37:17 AM
**IRM Calibration Status**	Success	**DA Method**	01.m
**Comment**			

Sample Group **Info.**

Acquisition SW Version 6200 series TOF/6500 series Q-TOF B.05.01 (B5125.1)

User Chromatograms

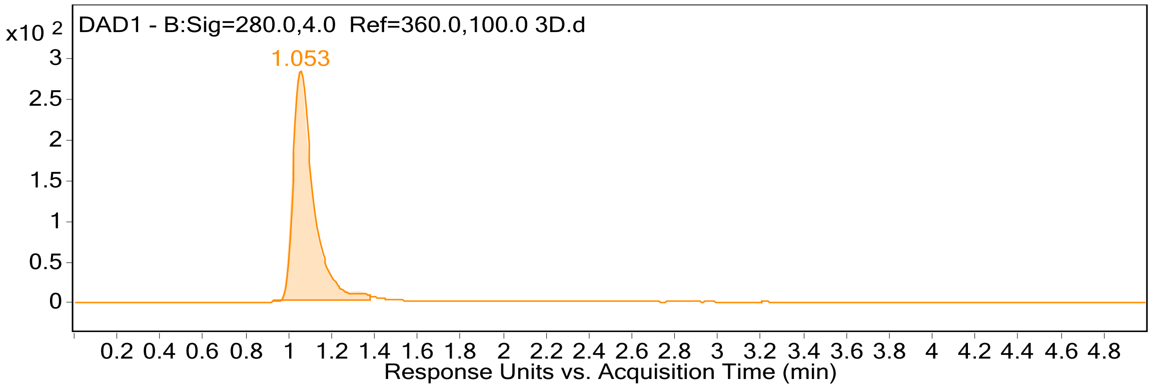

Integration Peak List

Peak	Start	RT	End	Height	Area	Area %
1	0.927	1.053	1.38	283.72	1917.01	100

--- End Of Report ---

Qualitative Analysis Report

**Data Filename**	3E.d	**Sample Name**	3E
**Sample Type**	Sample	**Position**	P1-A6
**Instrument Name**	Instrument 1	**User Name**	
**Acq Method**	20241027 scan +.m	**Acquired Time**	10/28/2024 2:43:14 AM
**IRM Calibration Status**	Success	**DA Method**	01.m
**Comment**			

Sample Group **Info.**
Acquisition SW Version 6200 series TOF/6500 series Q-TOF B.05.01 (B5125.1)

User Chromatograms

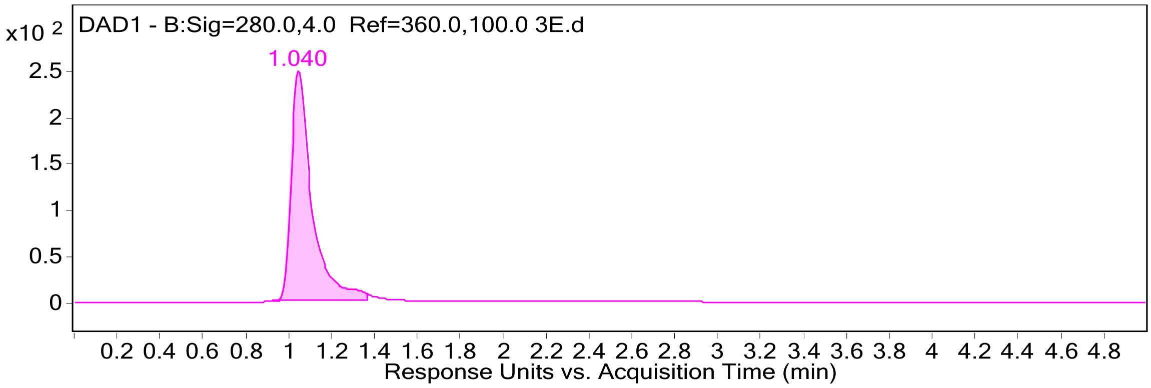

Integration Peak List

Peak	Start	RT	End	Height	Area	Area %
1	0.927	1.04	1.367	248.74	1696.79	100

--- End Of Report ---

Qualitative Analysis Report

**Data Filename**	3F.d	**Sample Name**	3F
**Sample Type**	Sample	**Position**	P1-A7
**Instrument Name**	Instrument 1	**User Name**	
**Acq Method**	20241027 scan +.m	**Acquired Time**	10/28/2024 2:55:09 AM
**IRM Calibration Status**	Success	**DA Method**	01.m
**Comment**			

Sample Group **Info.**

Acquisition SW Version 6200 series TOF/6500 series Q-TOF B.05.01 (B5125.1)

User Chromatograms

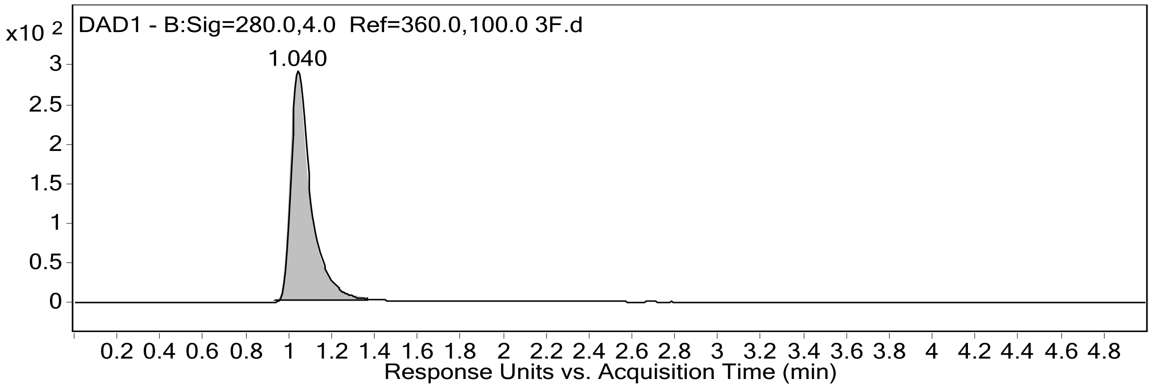

Integration Peak List

Peak	Start	RT	End	Height	Area	Area %
1	0.933	1.04	1.367	290.85	1917.3	100

--- End Of Report ---

Qualitative Analysis Report

**Data Filename**	3G.d	**Sample Name**	3G
**Sample Type**	Sample	**Position**	P1-A8
**Instrument Name**	Instrument 1	**User Name**	
**Acq Method**	20241027 scan +.m	**Acquired Time**	10/28/2024 3:01:06 AM
**IRM Calibration Status**	Success	**DA Method**	01.m
**Comment**			

**Sample Group**		**Info.**
**Acquisition SW Version**	6200 series TOF/6500 series Q-TOF B.05.01 (B5125.1)	

User Chromatograms

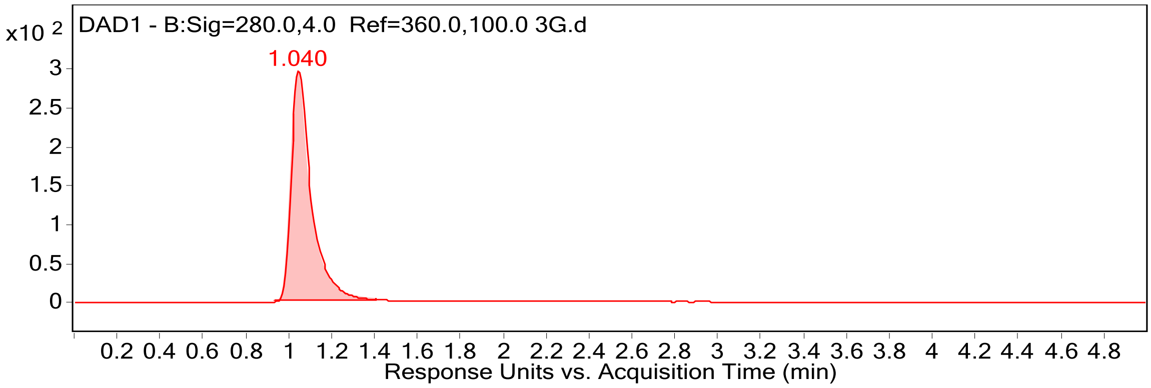

Integration Peak List

Peak	Start	RT	End	Height	Area	Area %
1	0.933	1.04	1.407	295.81	1970.34	100

--- End Of Report ---

Qualitative Analysis Report

**Data Filename**	3H.d	**Sample Name**	3H
**Sample Type**	Sample	**Position**	P1-A9
**Instrument Name**	Instrument 1	**User Name**	
**Acq Method**	20241027 scan +.m	**Acquired Time**	10/28/2024 3:07:01 AM
**IRM Calibration Status**	Success	**DA Method**	01.m
**Comment**			

Sample Group **Info.**

Acquisition SW 6200 series TOF/6500 series
Version Q-TOF B.05.01 (B5125.1)

User Chromatograms

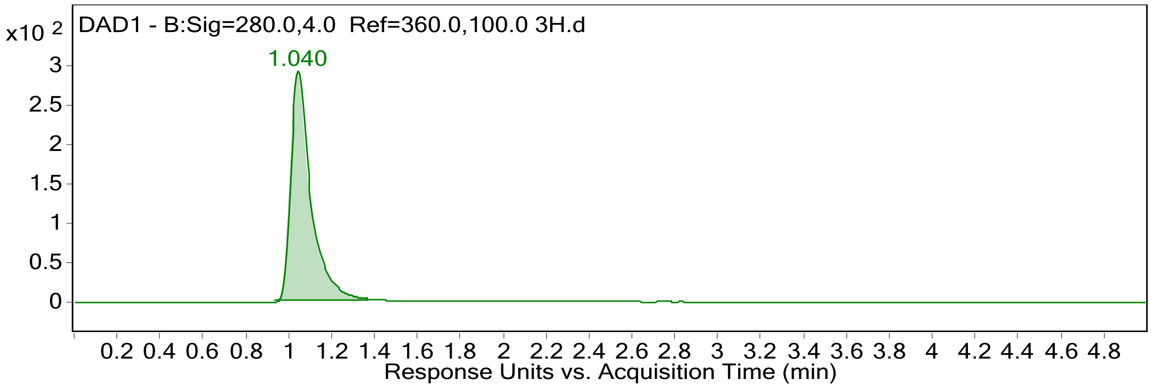

Integration Peak List

Peak	Start	RT	End	Height	Area	Area %
1	0.933	1.04	1.367	291.57	1932.02	100

--- End Of Report ---

Qualitative Analysis Report

**Data Filename**	3I.d	**Sample Name**	3I
**Sample Type**	Sample	**Position**	P1-B1
**Instrument Name**	Instrument 1	**User Name**	
**Acq Method**	20241027 scan +.m	**Acquired Time**	10/28/2024 3:12:57 AM
**IRM Calibration Status**	Success	**DA Method**	01.m
**Comment**			

Sample Group **Info.**

Acquisition SW 6200 series TOF/6500 series
Version Q-TOF B.05.01 (B5125.1)

User Chromatograms

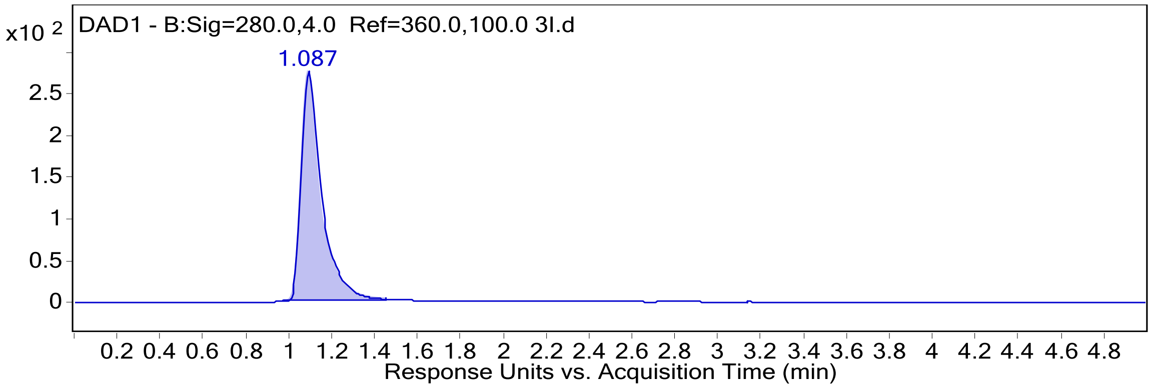

Integration Peak List

Peak	Start	RT	End	Height	Area	Area %
1	0.973	1.087	1.453	275.5	1874.41	100

--- End Of Report ---

Qualitative Analysis Report

**Data Filename**	3J.d	**Sample Name**	3J
**Sample Type**	Sample	**Position**	P1-B2
**Instrument Name**	Instrument 1	**User Name**	
**Acq Method**	20241027 scan +.m	**Acquired Time**	10/28/2024 3:18:56 AM
**IRM Calibration Status**	Success	**DA Method**	01.m
**Comment**			

Sample Group **Info.**

Acquisition SW 6200 series TOF/6500 series
Version Q-TOF B.05.01 (B5125.1)

User Chromatograms

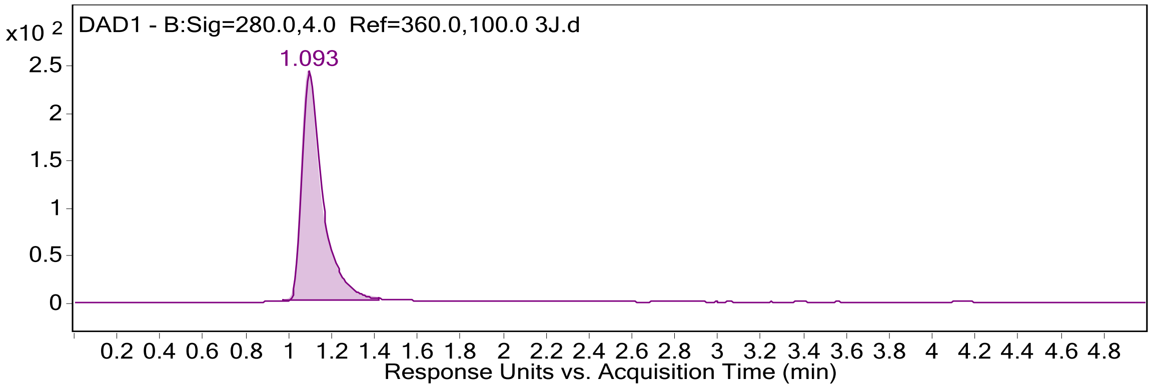

Integration Peak List

Peak	Start	RT	End	Height	Area	Area %
1	0.973	1.093	1.42	242.91	1670.74	100

--- End Of Report ---

Qualitative Analysis Report

**Data Filename**	3K.d	**Sample Name**	3K
**Sample Type**	Sample	**Position**	P1-B3
**Instrument Name**	Instrument 1	**User Name**	
**Acq Method**	20241027 scan +.m	**Acquired Time**	10/28/2024 3:30:50 AM
**IRM Calibration Status**	Success	**DA Method**	01.m
**Comment**			

Sample Group **Info.**

Acquisition SW Version 6200 series TOF/6500 series Q-TOF B.05.01 (B5125.1)

User Chromatograms

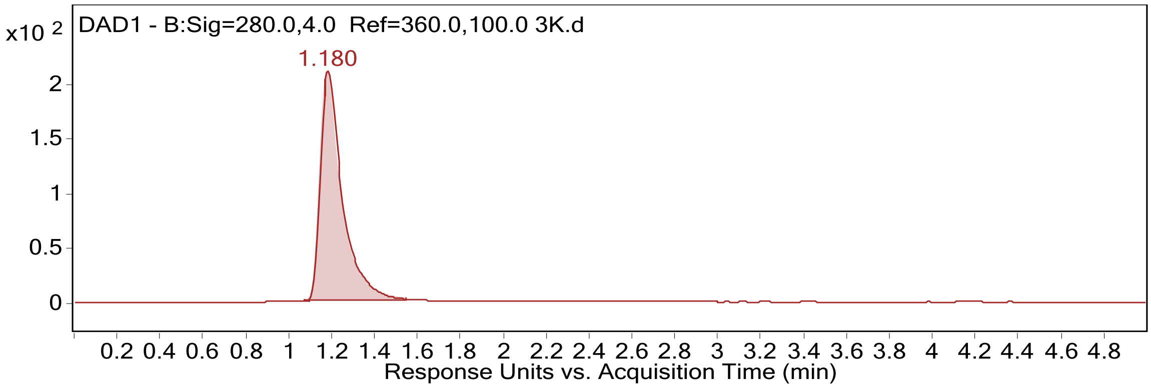

Integration Peak List

Peak	Start	RT	End	Height	Area	Area %
1	1.073	1.18	1.547	210.38	1533.82	100

--- End Of Report ---

Qualitative Analysis Report

**Data Filename**	3L.d	**Sample Name**	3L
**Sample Type**	Sample	**Position**	P1-B4
**Instrument Name**	Instrument 1	**User Name**	
**Acq Method**	20241027 scan +.m	**Acquired Time**	10/28/2024 3:36:48 AM
**IRM Calibration Status**	Success	**DA Method**	01.m
**Comment**			

Sample Group **Info.**

Acquisition SW Version 6200 series TOF/6500 series Q-TOF B.05.01 (B5125.1)

User Chromatograms

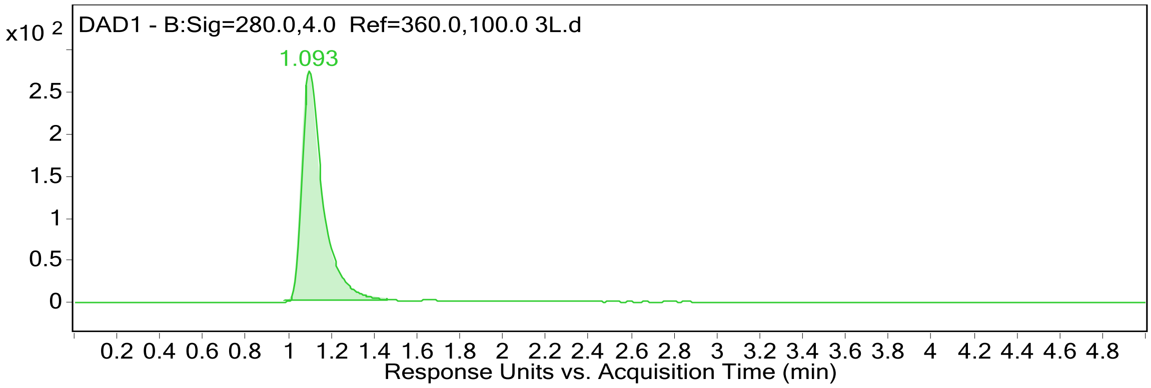

Integration Peak List

Peak	Start	RT	End	Height	Area	Area %
1	0.98	1.093	1.46	272.61	1897.47	100

--- End Of Report ---

Qualitative Analysis Report

**Data Filename**	3M.d	**Sample Name**	3M
**Sample Type**	Sample	**Position**	P1-B5
**Instrument Name**	Instrument 1	**User Name**	
**Acq Method**	20241027 scan +.m	**Acquired Time**	10/28/2024 3:42:46 AM
**IRM Calibration Status**	Success	**DA Method**	01.m
**Comment**			

Sample Group **Info.**

Acquisition SW Version 6200 series TOF/6500 series Q-TOF B.05.01 (B5125.1)

User Chromatograms

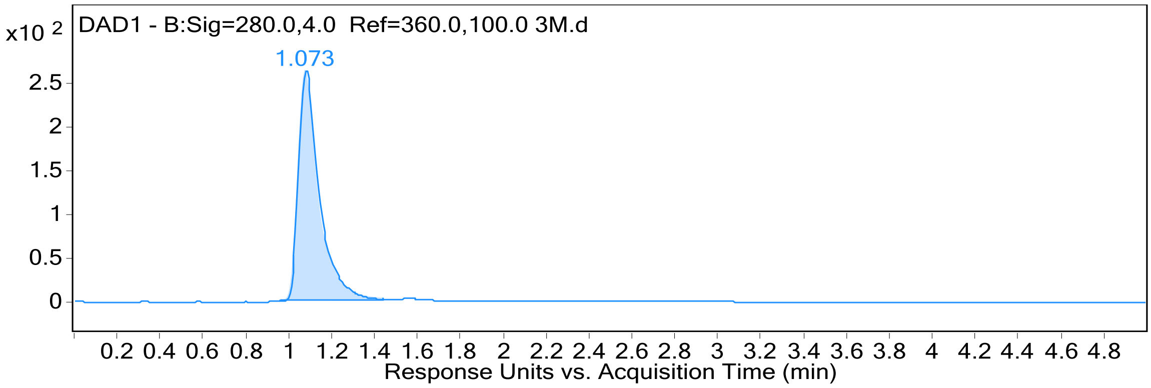

Integration Peak List

Peak	Start	RT	End	Height	Area	Area %
1	0.96	1.073	1.44	262.42	1817.43	100

--- End Of Report ---

Qualitative Analysis Report

**Data Filename**	3N.d	**Sample Name**	3N
**Sample Type**	Sample	**Position**	P1-B6
**Instrument Name**	Instrument 1	**User Name**	
**Acq Method**	20241027 scan +.m	**Acquired Time**	10/28/2024 3:48:46 AM
**IRM Calibration Status**	Success	**DA Method**	01.m
**Comment**			

Sample Group **Info.**

Acquisition SW Version 6200 series TOF/6500 series Q-TOF B.05.01 (B5125.1)

User Chromatograms

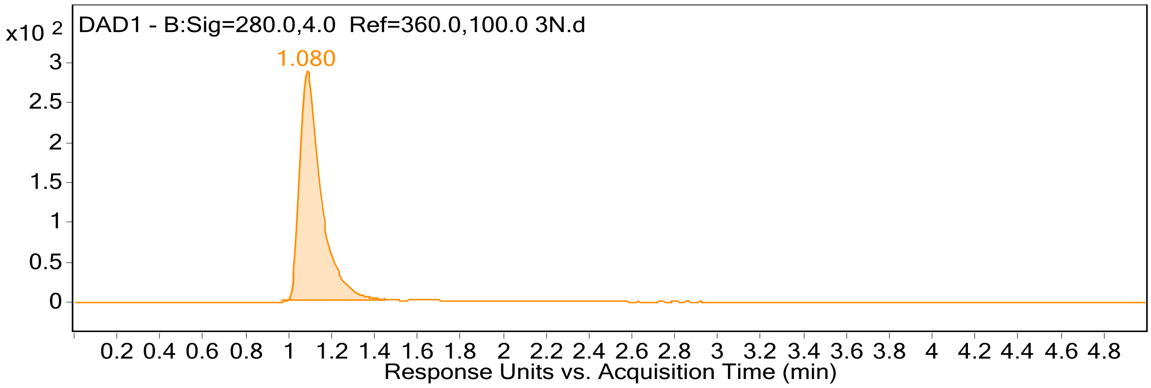

Integration Peak List

Peak	Start	RT	End	Height	Area	Area %
1	0.967	1.08	1.447	288.23	2028.35	100

--- End Of Report ---

Qualitative Analysis Report

**Data Filename**	3O.d	**Sample Name**	3O
**Sample Type**	Sample	**Position**	P1-B7
**Instrument Name**	Instrument 1	**User Name**	
**Acq Method**	20241027 scan +.m	**Acquired Time**	10/28/2024 3:54:44 AM
**IRM Calibration Status**	Success	**DA Method**	01.m
**Comment**			

Sample Group **Info.**

Acquisition SW 6200 series TOF/6500 series
Version Q-TOF B.05.01 (B5125.1)

User Chromatograms

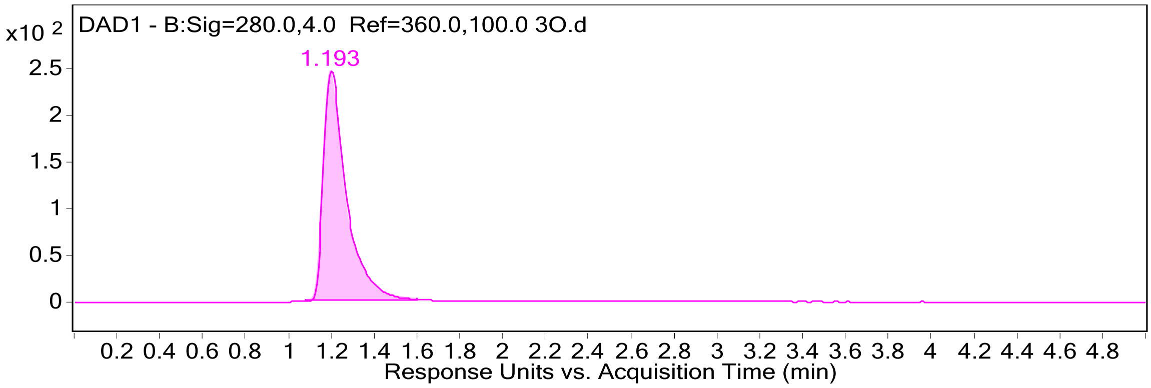

Integration Peak List

Peak	Start	RT	End	Height	Area	Area %
1	1.08	1.193	1.6	245.44	1868	100

--- End Of Report ---

Qualitative Analysis Report

**Data Filename**	3P.d	**Sample Name**	3P
**Sample Type**	Sample	**Position**	P1-B8
**Instrument Name**	Instrument 1	**User Name**	
**Acq Method**	20241027 scan +.m	**Acquired Time**	10/28/2024 4:06:40 AM
**IRM Calibration Status**	Success	**DA Method**	01.m
**Comment**			

Sample Group **Info.**

Acquisition SW Version 6200 series TOF/6500 series Q-TOF B.05.01 (B5125.1)

User Chromatograms

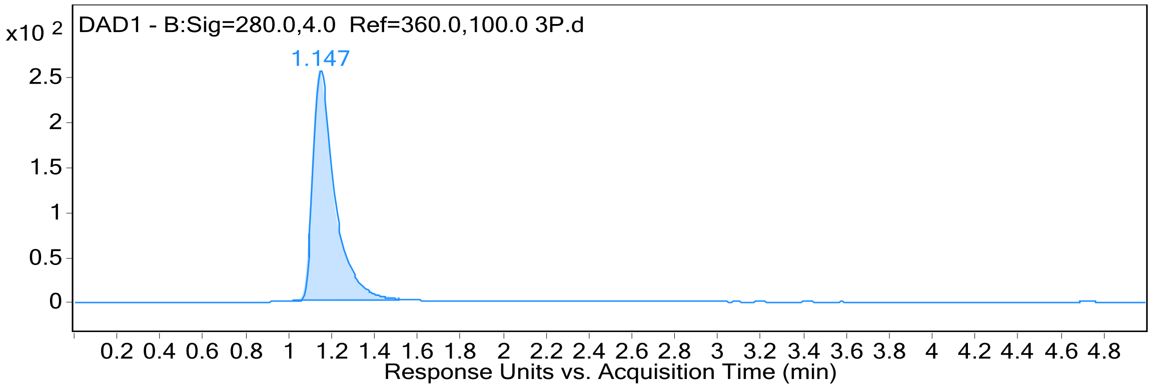

Integration Peak List

Peak	Start	RT	End	Height	Area	Area %
1	1.02	1.147	1.513	255.46	1848.34	100

--- End Of Report ---

Qualitative Analysis Report

**Data Filename**	3Q.d	**Sample Name**	3Q
**Sample Type**	Sample	**Position**	P1-B9
**Instrument Name**	Instrument 1	**User Name**	
**Acq Method**	20241027 scan +.m	**Acquired Time**	10/28/2024 4:12:35 AM
**IRM Calibration Status**	Success	**DA Method**	01.m
**Comment**			

Sample Group **Info.**

Acquisition SW 6200 series TOF/6500 series

Version Q-TOF B.05.01 (B5125.1)

User Chromatograms

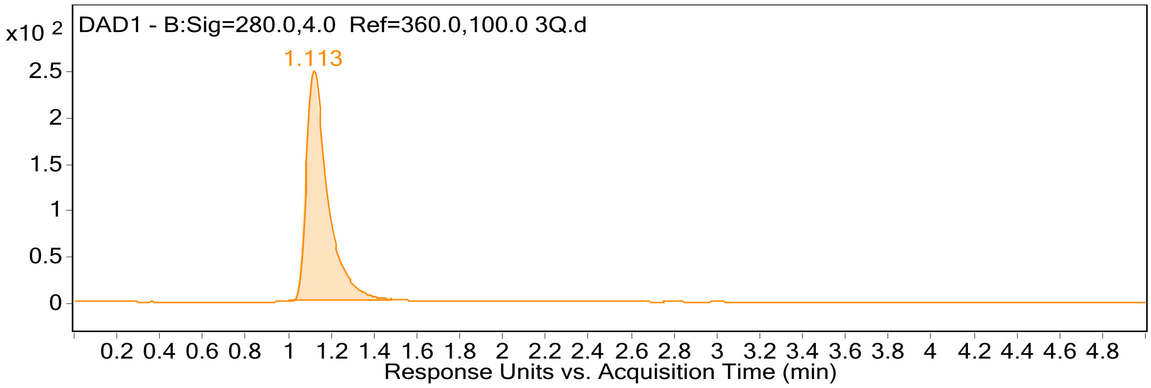

Integration Peak List

Peak	Start	RT	End	Height	Area	Area %
1	1	1.113	1.48	249.15	1763.88	100

--- End Of Report ---

Qualitative Analysis Report

**Data Filename**	3R.d	**Sample Name**	3R
**Sample Type**	Sample	**Position**	P1-C1
**Instrument Name**	Instrument 1	**User Name**	
**Acq Method**	20241027 scan +.m	**Acquired Time**	10/28/2024 4:18:32 AM
**IRM Calibration Status**	Success	**DA Method**	01.m
**Comment**			

Sample Group **Info.**

Acquisition SW 6200 series TOF/6500 series
Version Q-TOF B.05.01 (B5125.1)

User Chromatograms

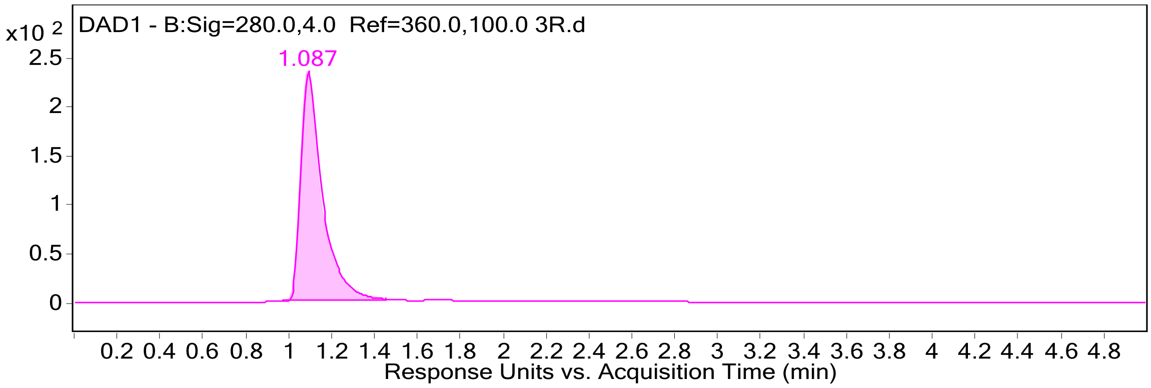

Integration Peak List

Peak	Start	RT	End	Height	Area	Area %
1	0.973	1.087	1.453	234.94	1668.68	100

--- End Of Report ---

Qualitative Analysis Report

**Data Filename**	3S.d	**Sample Name**	3S
**Sample Type**	Sample	**Position**	P1-C2
**Instrument Name**	Instrument 1	**User Name**	
**Acq Method**	20241027 scan +.m	**Acquired Time**	10/28/2024 4:24:30 AM
**IRM Calibration Status**	Success	**DA Method**	01.m
**Comment**			

Sample Group **Info.**
Acquisition SW Version 6200 series TOF/6500 series Q-TOF B.05.01 (B5125.1)

User Chromatograms

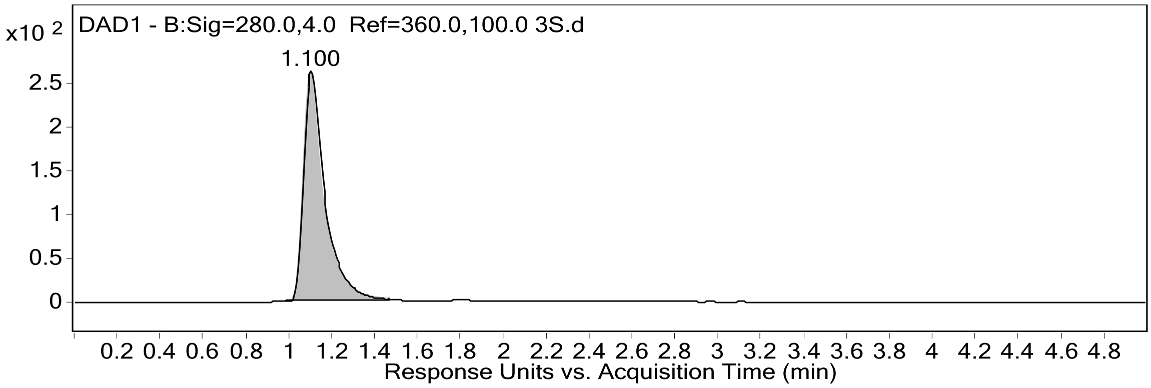

Integration Peak List

Peak	Start	RT	End	Height	Area	Area %
1	0.987	1.1	1.467	262.3	1836.68	100

--- End Of Report ---

Qualitative Analysis Report

**Data Filename**	3T.d	**Sample Name**	3T
**Sample Type**	Sample	**Position**	P1-C3
**Instrument Name**	Instrument 1	**User Name**	
**Acq Method**	20241027 scan +.m	**Acquired Time**	10/28/2024 4:30:29 AM
**IRM Calibration Status**	Success	**DA Method**	01.m
**Comment**			

Sample Group **Info.**

Acquisition SW Version 6200 series TOF/6500 series Q-TOF B.05.01 (B5125.1)

User Chromatograms

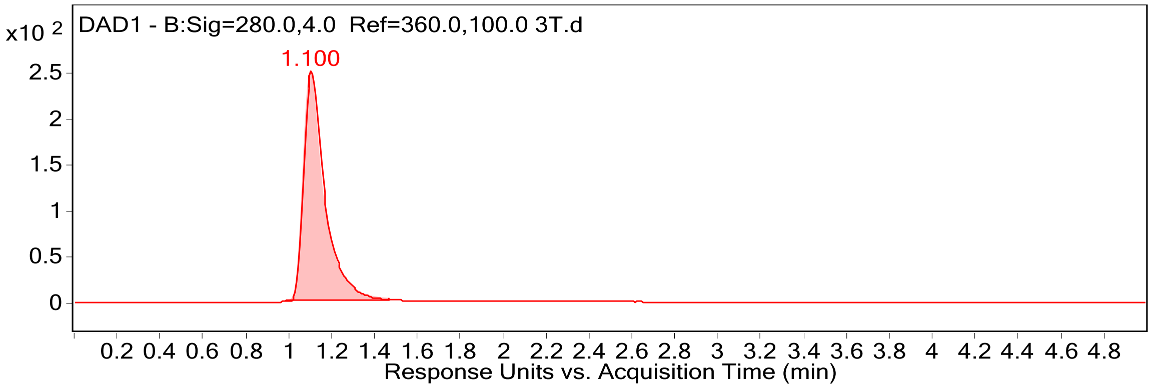

Integration Peak List

Peak	Start	RT	End	Height	Area	Area %
1	0.987	1.1	1.467	251.2	1766.94	100

--- End Of Report ---

Qualitative Analysis Report

**Data Filename**	3U.d	**Sample Name**	3U
**Sample Type**	Sample	**Position**	P1-C4
**Instrument Name**	Instrument 1	**User Name**	
**Acq Method**	20241027 scan +.m	**Acquired Time**	10/28/2024 4:42:26 AM
**IRM Calibration Status**	Success	**DA Method**	01.m
**Comment**			

Sample Group **Info.**
Acquisition SW 6200 series TOF/6500 series
Version Q-TOF B.05.01 (B5125.1)

User Chromatograms

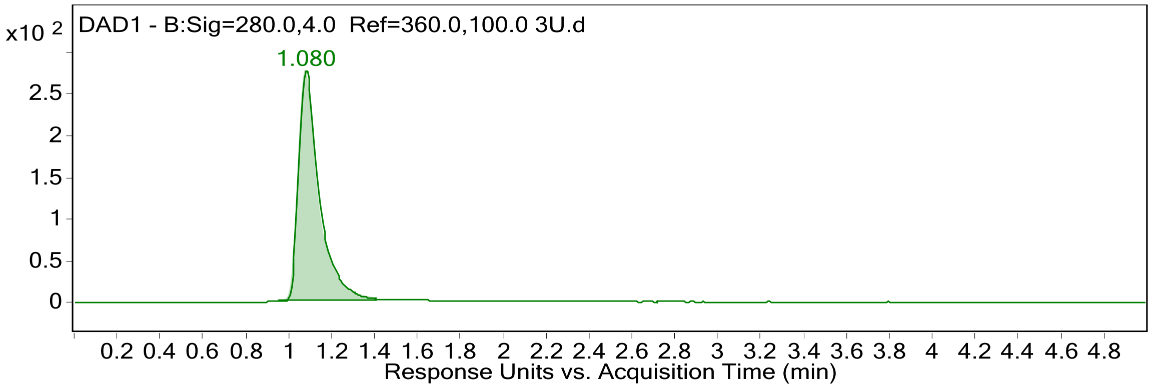

Integration Peak List

Peak	Start	RT	End	Height	Area	Area %
1	0.953	1.08	1.407	276.13	1886.69	100

--- End Of Report ---

Qualitative Analysis Report

**Data Filename**	3V.d	**Sample Name**	3V
**Sample Type**	Sample	**Position**	P1-C5
**Instrument Name**	Instrument 1	**User Name**	
**Acq Method**	20241027 scan +.m	**Acquired Time**	10/28/2024 4:48:24 AM
**IRM Calibration Status**	Success	**DA Method**	01.m
**Comment**			

Sample Group **Info.**

Acquisition SW 6200 series TOF/6500 series
Version Q-TOF B.05.01 (B5125.1)

User Chromatograms

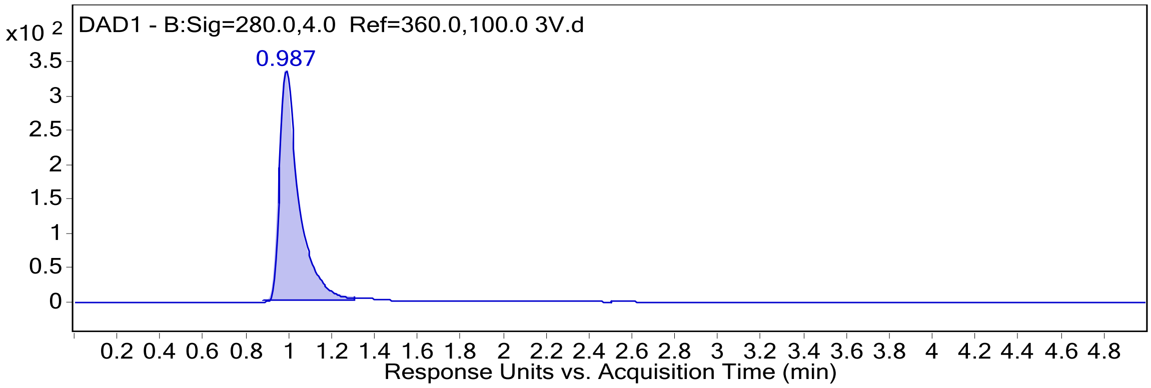

Integration Peak List

Peak	Start	RT	End	Height	Area	Area %
1	0.88	0.987	1.307	334.68	2143.23	100

--- End Of Report ---

Qualitative Analysis Report

**Data Filename**	4L.d	**Sample Name**	4L
**Sample Type**	Sample	**Position**	P1-A2
**Instrument Name**	Instrument 1	**User Name**	
**Acq Method**	20241027 scan +.m	**Acquired Time**	10/28/2024 5:13:05 PM
**IRM Calibration Status**	Success	**DA Method**	01.m
**Comment**			

Sample Group **Info.**

Acquisition SW Version 6200 series TOF/6500 series Q-TOF B.05.01 (B5125.1)

User Chromatograms

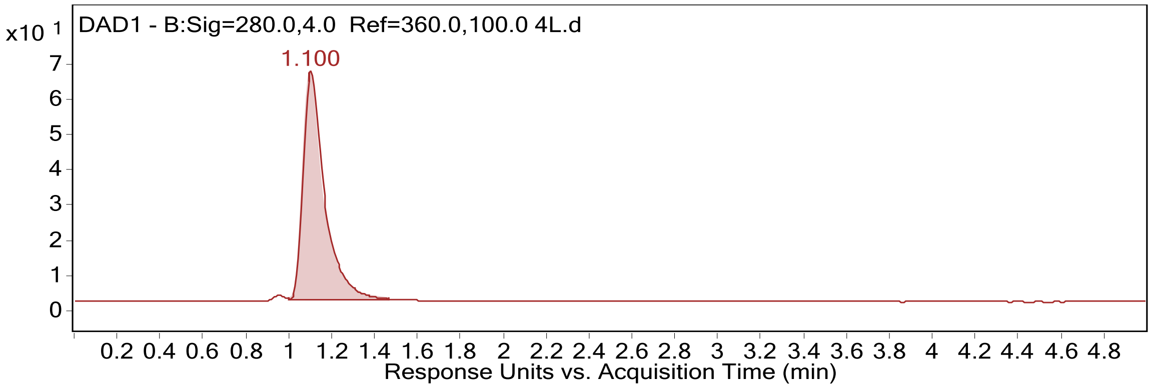

Integration Peak List

Peak	Start	RT	End	Height	Area	Area %
1	1	1.1	1.467	65.11	461.81	100

--- End Of Report ---

Qualitative Analysis Report

**Data Filename**	4M.d	**Sample Name**	4M
**Sample Type**	Sample	**Position**	P1-A3
**Instrument Name**	Instrument 1	**User Name**	
**Acq Method**	20241027 scan +.m	**Acquired Time**	10/28/2024 5:19:03 PM
**IRM Calibration Status**	Success	**DA Method**	01.m
**Comment**			

Sample Group **Info.**

Acquisition SW Version 6200 series TOF/6500 series Q-TOF B.05.01 (B5125.1)

User Chromatograms

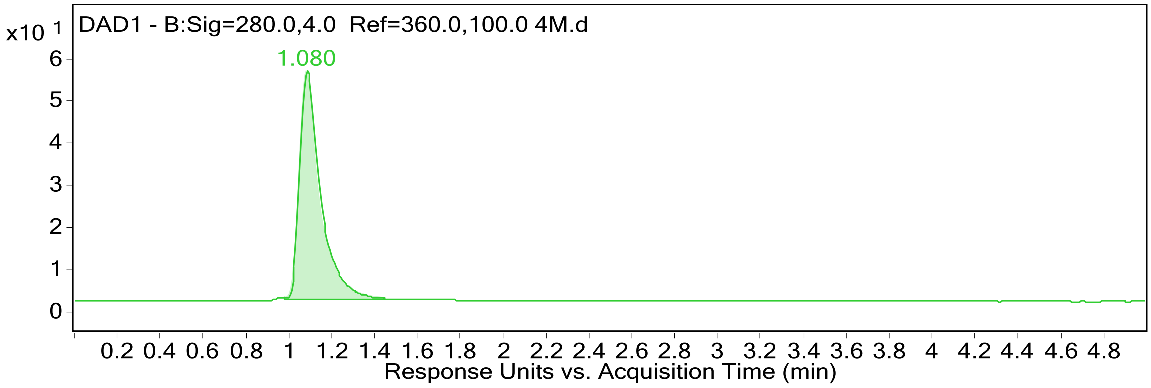

Integration Peak List

Peak	Start	RT	End	Height	Area	Area %
1	0.98	1.08	1.447	54.44	380.71	100

--- End Of Report ---

Qualitative Analysis Report

**Data Filename**	4N.d	**Sample Name**	4N
**Sample Type**	Sample	**Position**	P1-A4
**Instrument Name**	Instrument 1	**User Name**	
**Acq Method**	20241027 scan +.m	**Acquired Time**	10/28/2024 5:25:01 PM
**IRM Calibration Status**	Success	**DA Method**	01.m
**Comment**			

Sample Group **Info.**
Acquisition SW 6200 series TOF/6500 series
Version Q-TOF B.05.01 (B5125.1)

User Chromatograms

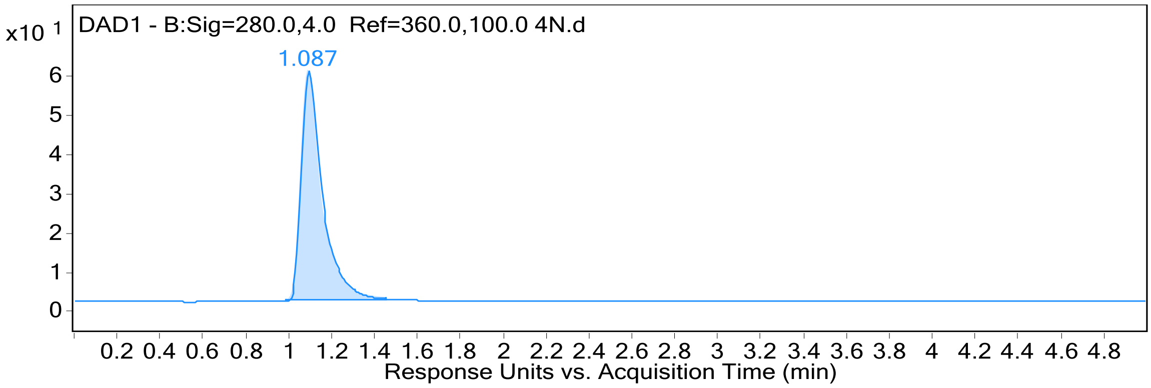

Integration Peak List

Peak	Start	RT	End	Height	Area	Area %
1	0.987	1.087	1.453	58.56	411.76	100

--- End Of Report ---

Qualitative Analysis Report

**Data Filename**	4O.d	**Sample Name**	4O
**Sample Type**	Sample	**Position**	P1-A5
**Instrument Name**	Instrument 1	**User Name**	
**Acq Method**	20241027 scan +.m	**Acquired Time**	10/28/2024 5:31:00 PM
**IRM Calibration Status**	Success	**DA Method**	01.m
**Comment**			

Sample Group **Info.**

Acquisition SW Version 6200 series TOF/6500 series Q-TOF B.05.01 (B5125.1)

User Chromatograms

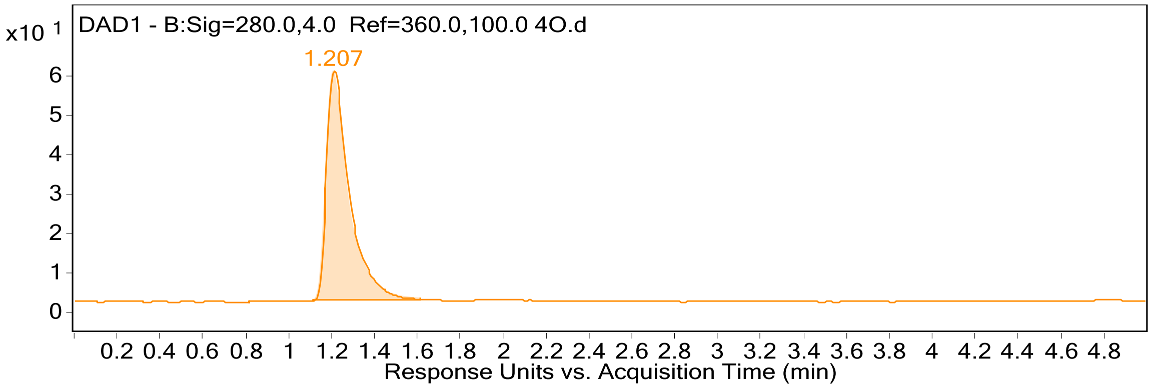

Integration Peak List

Peak	Start	RT	End	Height	Area	Area %
1	1.113	1.207	1.613	58.56	447.95	100

--- End Of Report ---

Qualitative Analysis Report

**Data Filename**	4P.d	**Sample Name**	4P
**Sample Type**	Sample	**Position**	P1-A6
**Instrument Name**	Instrument 1	**User Name**	
**Acq Method**	20241027 scan +.m	**Acquired Time**	10/28/2024 5:36:59 PM
**IRM Calibration Status**	Success	**DA Method**	01.m
**Comment**			

Sample Group **Info.**

Acquisition SW Version 6200 series TOF/6500 series Q-TOF B.05.01 (B5125.1)

User Chromatograms

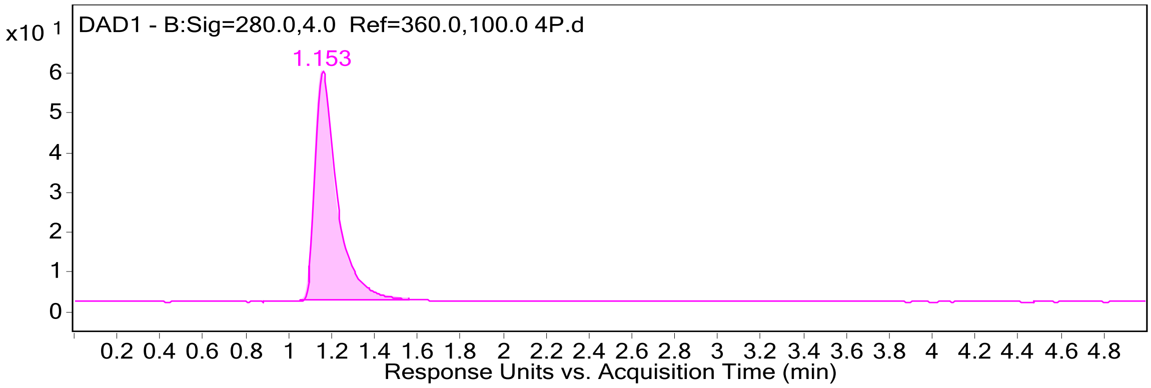

Integration Peak List

Peak	Start	RT	End	Height	Area	Area %
1	1.053	1.153	1.56	57.77	420.92	100

--- End Of Report ---

Qualitative Analysis Report

**Data Filename**	5L.d	**Sample Name**	5L
**Sample Type**	Sample	**Position**	P1-A7
**Instrument Name**	Instrument 1	**User Name**	
**Acq Method**	20241027 scan +.m	**Acquired Time**	10/28/2024 5:48:56 PM
**IRM Calibration Status**	Success	**DA Method**	01.m
**Comment**			

Sample Group **Info.**

Acquisition SW Version 6200 series TOF/6500 series Q-TOF B.05.01 (B5125.1)

User Chromatograms

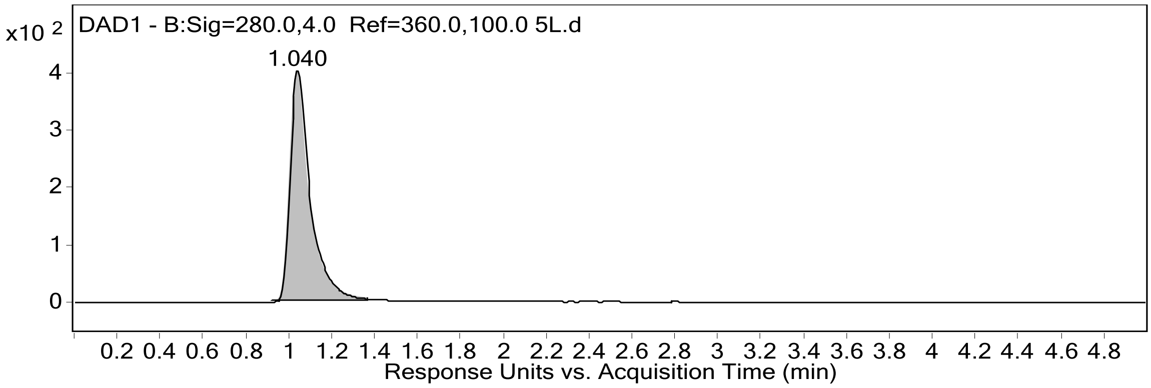

Integration Peak List

Peak	Start	RT	End	Height	Area	Area %
1	0.92	1.04	1.367	402.47	2675.5	100

--- End Of Report ---

Qualitative Analysis Report

**Data Filename**	5M.d	**Sample Name**	5M
**Sample Type**	Sample	**Position**	P1-A8
**Instrument Name**	Instrument 1	**User Name**	
**Acq Method**	20241027 scan +.m	**Acquired Time**	10/28/2024 5:54:55 PM
**IRM Calibration Status**	Success	**DA Method**	01.m
**Comment**			

Sample Group **Info.**

Acquisition SW Version 6200 series TOF/6500 series Q-TOF B.05.01 (B5125.1)

User Chromatograms

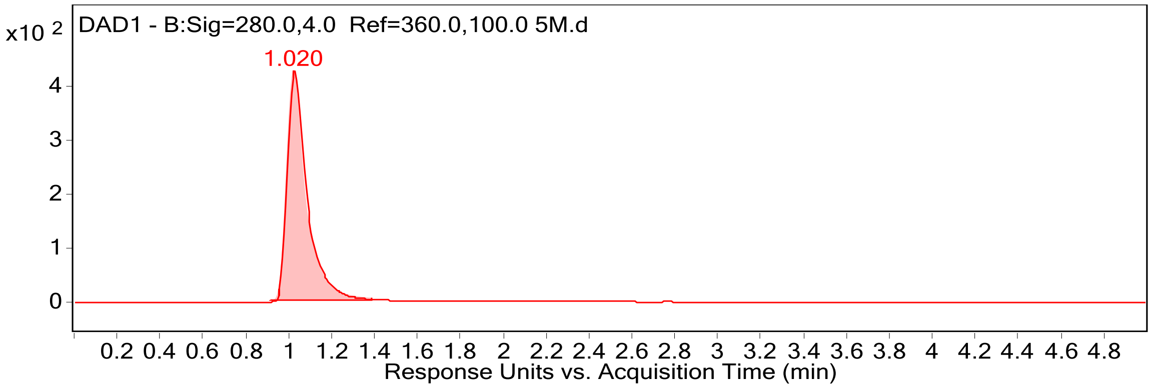

Integration Peak List

Peak	Start	RT	End	Height	Area	Area %
1	0.913	1.02	1.387	428.11	2830.36	100

--- End Of Report ---

Qualitative Analysis Report

**Data Filename**	5N.d	**Sample Name**	5N
**Sample Type**	Sample	**Position**	P1-A9
**Instrument Name**	Instrument 1	**User Name**	
**Acq Method**	20241027 scan +.m	**Acquired Time**	10/28/2024 6:00:51 PM
**IRM Calibration Status**	Success	**DA Method**	01.m
**Comment**			

Sample Group **Info.**

Acquisition SW Version 6200 series TOF/6500 series Q-TOF B.05.01 (B5125.1)

User Chromatograms

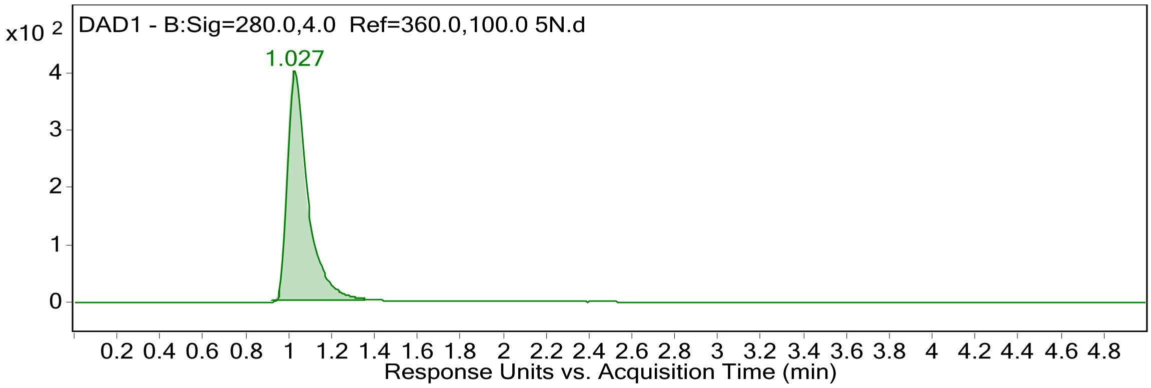

Integration Peak List

Peak	Start	RT	End	Height	Area	Area %
1	0.92	1.027	1.353	402.71	2698.04	100

--- End Of Report ---

Qualitative Analysis Report

**Data Filename**	5O.d	**Sample Name**	5O
**Sample Type**	Sample	**Position**	P1-B1
**Instrument Name**	Instrument 1	**User Name**	
**Acq Method**	20241027 scan +.m	**Acquired Time**	10/28/2024 6:06:46 PM
**IRM Calibration Status**	Success	**DA Method**	01.m
**Comment**			

Sample Group **Info.**

Acquisition SW 6200 series TOF/6500 series
Version Q-TOF B.05.01 (B5125.1)

User Chromatograms

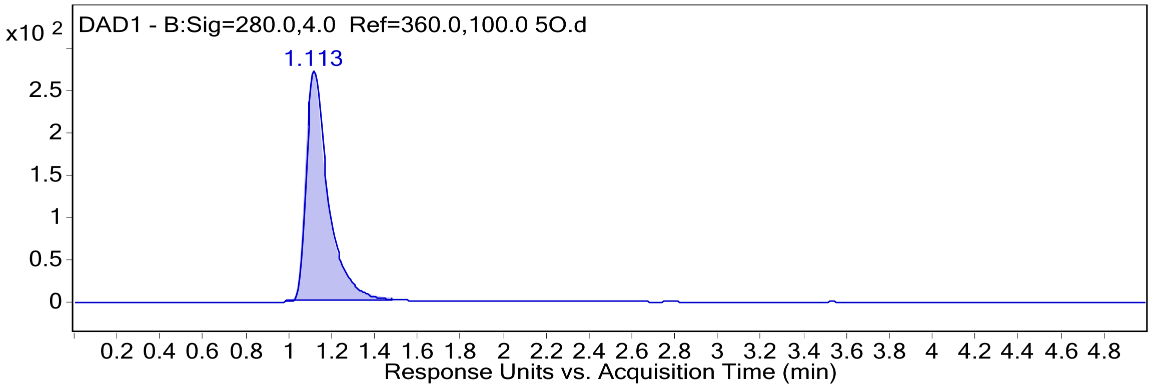

Integration Peak List

Peak	Start	RT	End	Height	Area	Area %
1	0.987	1.113	1.48	272.01	1970.9	100

--- End Of Report ---

Qualitative Analysis Report

**Data Filename**	5P.d	**Sample Name**	5P
**Sample Type**	Sample	**Position**	P1-B2
**Instrument Name**	Instrument 1	**User Name**	
**Acq Method**	20241027 scan +.m	**Acquired Time**	10/28/2024 6:12:45 PM
**IRM Calibration Status**	Success	**DA Method**	01.m
**Comment**			

Sample Group **Info.**

Acquisition SW 6200 series TOF/6500 series

Version Q-TOF B.05.01 (B5125.1)

User Chromatograms

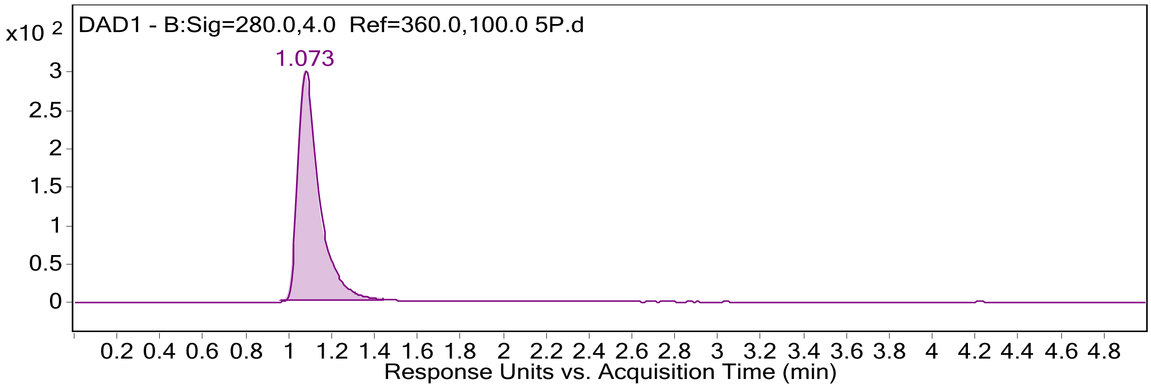

Integration Peak List

Peak	Start	RT	End	Height	Area	Area %
1	0.96	1.073	1.44	299.49	2113.53	100

--- End Of Report ---

Qualitative Analysis Report

**Data Filename**	6L.d	**Sample Name**	6L
**Sample Type**	Sample	**Position**	P1-B3
**Instrument Name**	Instrument 1	**User Name**	
**Acq Method**	20241027 scan +.m	**Acquired Time**	10/28/2024 6:24:41 PM
**IRM Calibration Status**	Success	**DA Method**	01.m
**Comment**			

Sample Group **Info.**
Acquisition SW 6200 series TOF/6500 series
Version Q-TOF B.05.01 (B5125.1)

User Chromatograms

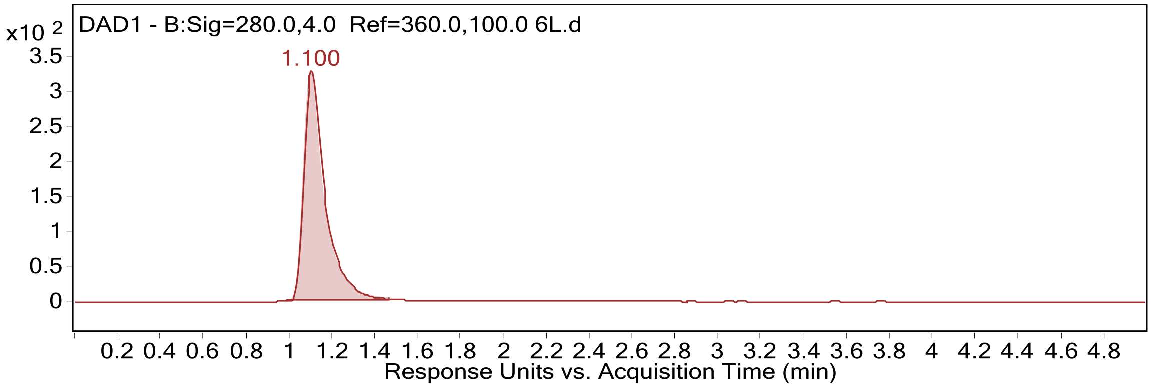

Integration Peak List

Peak	Start	RT	End	Height	Area	Area %
1	0.987	1.1	1.467	328.56	2302.29	100

--- End Of Report ---

Qualitative Analysis Report

**Data Filename**	6M.d	**Sample Name**	6M
**Sample Type**	Sample	**Position**	P1-B4
**Instrument Name**	Instrument 1	**User Name**	
**Acq Method**	20241027 scan +.m	**Acquired Time**	10/28/2024 6:30:39 PM
**IRM Calibration Status**	Success	**DA Method**	01.m
**Comment**			

Sample Group **Info.**

Acquisition SW 6200 series TOF/6500 series
Version Q-TOF B.05.01 (B5125.1)

User Chromatograms

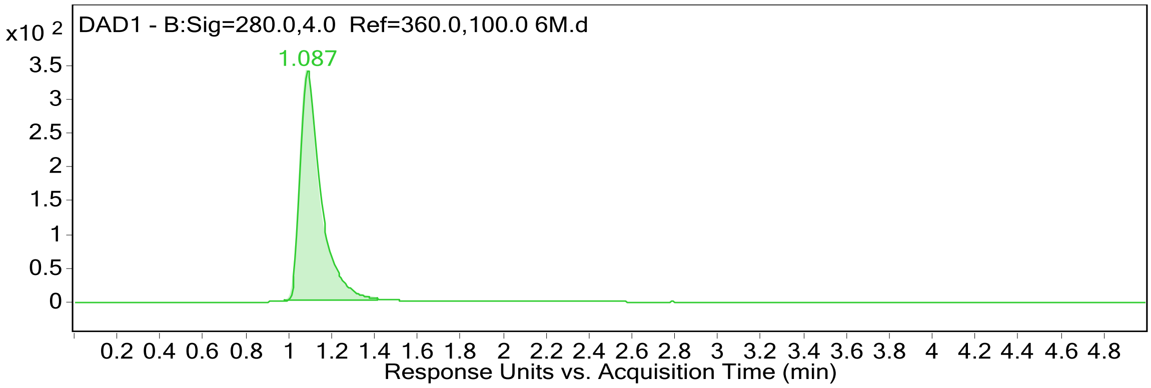

Integration Peak List

Peak	Start	RT	End	Height	Area	Area %
1	0.98	1.087	1.413	339.95	2339.28	100

--- End Of Report ---

Qualitative Analysis Report

**Data Filename**	6N.d	**Sample Name**	6N
**Sample Type**	Sample	**Position**	P1-B5
**Instrument Name**	Instrument 1	**User Name**	
**Acq Method**	20241027 scan +.m	**Acquired Time**	10/28/2024 6:36:41 PM
**IRM Calibration Status**	Success	**DA Method**	01.m
**Comment**			

Sample Group **Info.**

Acquisition SW Version 6200 series TOF/6500 series Q-TOF B.05.01 (B5125.1)

User Chromatograms

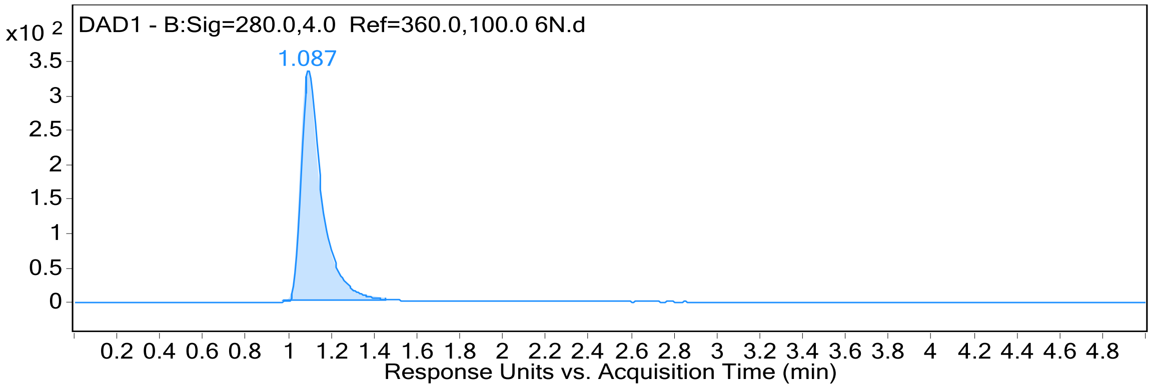

Integration Peak List

Peak	Start	RT	End	Height	Area	Area %
1	0.973	1.087	1.453	334.85	2332.21	100

--- End Of Report ---

Qualitative Analysis Report

**Data Filename**	6O.d	**Sample Name**	6O
**Sample Type**	Sample	**Position**	P1-B6
**Instrument Name**	Instrument 1	**User Name**	
**Acq Method**	20241027 scan +.m	**Acquired Time**	10/28/2024 6:42:38 PM
**IRM Calibration Status**	Success	**DA Method**	01.m
**Comment**			

Sample Group **Info.**

Acquisition SW 6200 series TOF/6500 series
Version Q-TOF B.05.01 (B5125.1)

User Chromatograms

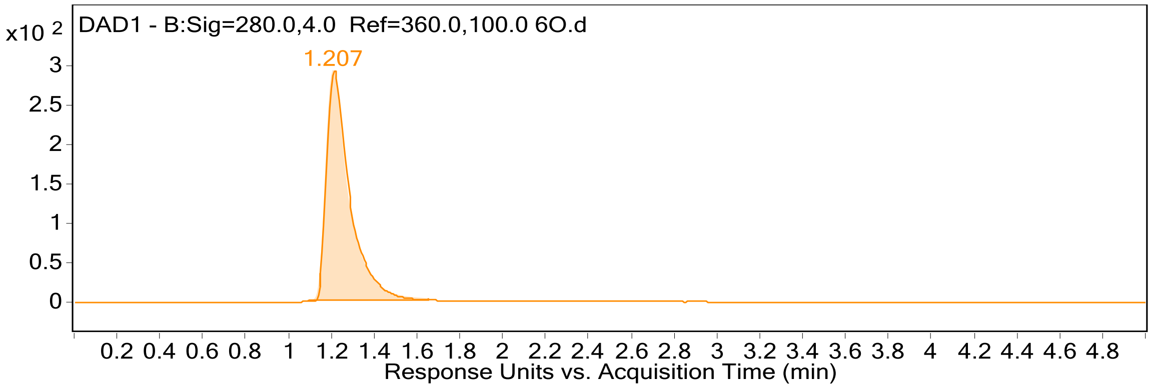

Integration Peak List

Peak	Start	RT	End	Height	Area	Area %
1	1.093	1.207	1.653	291.23	2246.56	100

--- End Of Report ---

Qualitative Analysis Report

**Data Filename**	6P.d	**Sample Name**	6P
**Sample Type**	Sample	**Position**	P1-B7
**Instrument Name**	Instrument 1	**User Name**	
**Acq Method**	20241027 scan +.m	**Acquired Time**	10/28/2024 6:48:36 PM
**IRM Calibration Status**	Success	**DA Method**	01.m
**Comment**			

Sample Group **Info.**

Acquisition SW Version 6200 series TOF/6500 series Q-TOF B.05.01 (B5125.1)

User Chromatograms

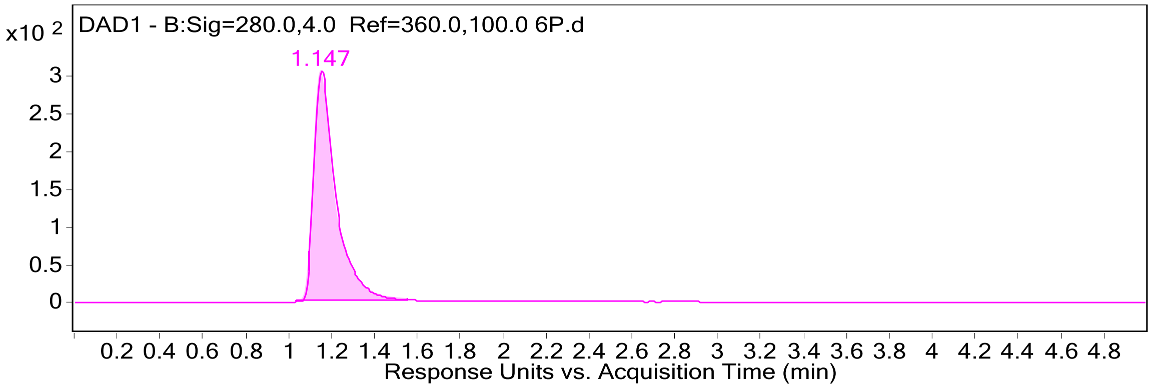

Integration Peak List

Peak	Start	RT	End	Height	Area	Area %
1	1.033	1.147	1.553	305.77	2228.21	100

--- End Of Report ---
